# Radioisotopes demonstrate changes in global atmospheric circulation possibly caused by global warming

**DOI:** 10.1038/s41598-020-66541-5

**Published:** 2020-07-01

**Authors:** Lucrezia Terzi, Gerhard Wotawa, Michael Schoeppner, Martin Kalinowski, Paul R. J. Saey, Philipp Steinmann, Lan Luan, Paul W. Staten

**Affiliations:** 1 Belgian Nuclear Research Centre (SCK•CEN), Mol, Belgium; 2 Technische Universität Wien, Atominstitut, Austria; 3 Zentralanstalt für Meteorologie und Geodynamik (ZAMG), Vienna, Austria; 4 Provisional Technical Secretariat, Preparatory Commission for the Nuclear-Test-Ban Treaty Organization, International Data Centre, Vienna, Austria; 5 University of Natural Resources and Life Sciences, Institute of Safety/Security and Risk Sciences, Vienna, Austria; 6 Federal Office of Public Health (BAG), Bern, Switzerland; 7 Indiana University Bloomington, Bloomington, Indiana, USA

**Keywords:** Climate sciences, Atmospheric science, Atmospheric dynamics, Environmental sciences, Environmental chemistry, Environmental monitoring

## Abstract

In this paper, we present a new method to study global atmospheric processes and their changes during the last decade. A cosmogenic radionuclide measured at ground-level, beryllium-7, is utilized as a proxy to study atmospheric dynamics. Beryllium-7 has two advantages: First, this radionuclide, primarily created in the lower stratosphere, attaches to aerosols that are transported downwards to the troposphere and travel around the globe with the general atmospheric circulation. By monitoring these particles, we can provide a global, simple, and sustainable way to track processes such as multi-annual variation of the troposphere, tropopause heightening, position and speed of atmospheric interface zones, as well as the poleward movement and stalling patterns of jet streams. Second, beryllium-7 is a product of cosmic rays which are themselves directly linked to solar activity and the earth magnetic field. This study shows whether beryllium-7 observed concentration changes are correlated with such natural processes or independent of them. Our work confirms that major changes in the atmospheric circulation are currently ongoing, even though timeseries are too short to make climatological assessments. We provide solid evidence of significant and progressive changes of the global atmospheric circulation as well as modifications of tropopause heights over the past decade. As the last decade happened to be the warmest on record, this analysis also indicates that the observed changes are, at least to some extent, attributable to global warming.

## Introduction

The increase of global average temperatures and of the frequency of severe weather conditions such as floods, storms and droughts are directly attributable to the anthropogenic emissions of greenhouse gases^1–14^. The rapid growth of greenhouse gas concentrations in the atmosphere is leading to significant changes in global atmospheric circulation patterns, from poleward movements and stalling patterns (blocking) of jet streams, to multi-annual variation of the troposphere and global tropopause heightening at mid-latitudes^1,10,14–24^.

The global circulation is weakening and this is linked to extra-tropic forcing^6^, meaning that warming is enhanced at the mid-latitudes (strong emissions of CO_2_) and the polar region (melting of ice shield) compared with the tropics. Simulations show that injection of greenhouse gases has proven to be much more effective when delivered at the mid latitudes because they decrease the temperature differences between the tropical and extra-tropical regions^2,10,12^ and therefore induce a slowdown of the general circulation. However, these changes in global circulation are rather complex and cannot be monitored as easily as changes in temperature, precipitation, and surface solar radiation^25^.

It is certainly common sense that beryllium-7 concentrations are not suitable to directly measure atmospheric circulation. They are a useful proxy to observe the annual and inter-annual dynamics of atmospheric circulation cells.

The causal relationship between tropopause and beryllium-7 is mainly reported in Delaygue *et al*.^26^ where we see a clear link between beryllium-7 isolines and tropopause height: the higher the troposphere, the larger the region (tropospheric column) with higher formation of beryllium-7.

Beryllium-7 alone cannot distinguish between stratosphere and troposphere production, but variation in air mixing and advection may have an effect on the observed trends. These uncertainties need to be taken into account. Analysis on the ratio between beryllium-7 and sodium-22 ratio can shed light on such processes and therefore needs to be further developed.

One example of the complexity is the North Atlantic region, where a recent cooling trend of sea-surface temperatures caused by a weakening of the Atlantic thermohaline circulation leads to a circulation amplification and increase of severe storms over western Europe in wintertime.

The weakening of the circulation and the decreasing temperature difference between polar region and tropics is not demonstrated by this study, but taken from scientific literature and the IPCC AR5 report^25^.

Regarding the mechanism of weakening of the tropical circulation, the decrease in temperature gradient could decelerate the extratropical circulation through baroclinic process but not directly relates the tropical circulation^27^.

The effect of melting ice may well be much more important compared with the effects of concentration differences of CO_2_ between emission regions in the Northern Hemisphere’s mid-latitudes and the global background, which are on the order of 10–20 ppm on a background of 400 ppm^28^. We cannot measure this directly based on isotopes. What we see and describe is the systematic changes and trends of beryllium-7 concentrations during the last 1–2 decades, presumably caused by gradual changes in circulation patterns^19,29^.

Global atmospheric processes which are expected to change due to global warming can be indirectly observed by monitoring cosmogenic radionuclides. In this study, measurements of the ground-level activity concentrations are used as a proxy of vertical exchange and circulation patterns^26,30–37^.

The analyses presented below address how the time series of radionuclide concentrations at ground level are modified by five processes, namely (a) cosmic rays, (b) variations of troposphere heights, (c) the poleward shift of the seasonal turning point of the Hadley-Ferrel interface zone, (d) the slowing down of atmospheric cell movements, and (e) a tendency towards static weather conditions. The reference to static weather conditions in this paper refers to the indication provided by beryllium-7 data that, during boreal summer, the tropical cell moves poleward, covering larger areas of the hemisphere. The westerly circulation band (referred to as Ferrel Cell in the manuscript), where the weather is subject to higher variability, exercises less and less influence.

Poleward shift not only refers to the northward movement of the Hadley cell in the Northern Hemisphere (or southward in the Southern Hemisphere) but also the poleward movement of tropical and polar jet streams and the global Rossby wave^9,10,12,38^. In the last decade. This movement of the tropical Hadley cell in northern hemispheric spring and summer has enhanced (within tropopause multi-annual variability) leading to a widening of the tropical belt. This poleward movement of circulation cells as well as the widening of the tropical belt is also described in IPCC AR5^25^.

**Figure 1:**
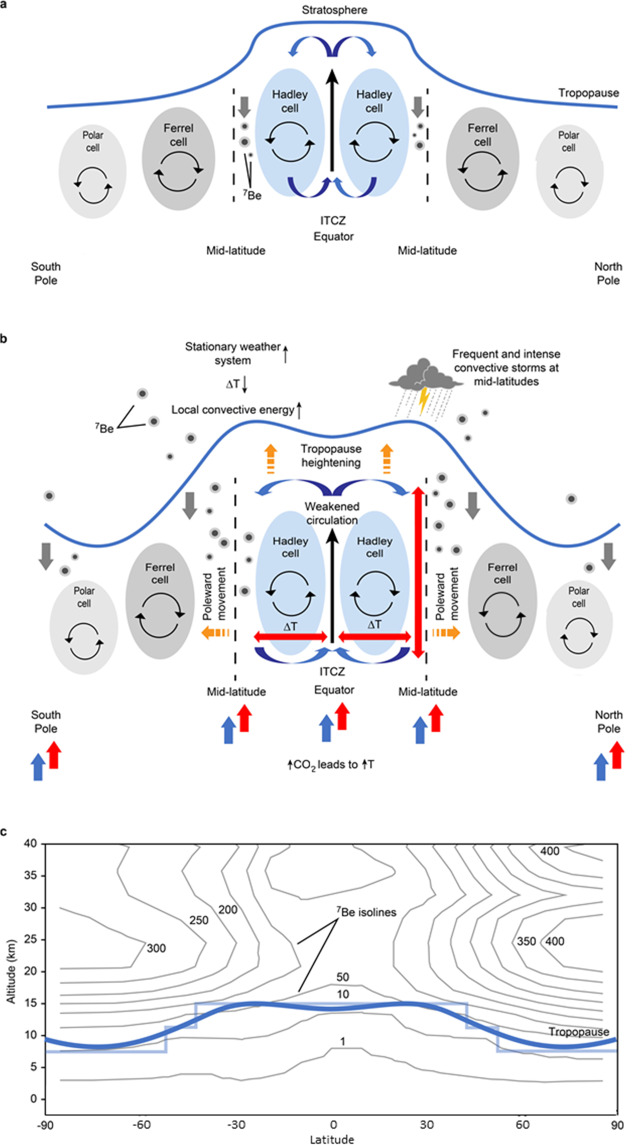
(**a**) Basic atmospheric cell circulation diagram of beryllium-7 being transported to the surface through stratosphere–troposphere exchange in the mid-latitudes (HFIZ) and polar latitudes (PFCZ). (**b**) Global warming caused by increasing CO_2_ concentrations trigger the following changes in the global circulation: (1) tropopause height increase, (2) poleward extension of the Hadley cell, and (3) weakening of the atmospheric circulation in the mid-latitudes due to the decreasing temperature difference between the equator and the mid-latitudes. Poleward extension and weakened circulation extend the area where cosmogenic radionuclides are detected and increase the period of radionuclide detection during summer months. In the mid-latitudes, weaker circulation and increased vertical temperature differences due to the rising of the tropopause have two effects: stationary weather system and increased convective energy, resulting in more frequent and stronger convective storms. (**a**,**b**) are simplified with respect to the STE (Stratosphere-Troposphere exchange), as we do not represent tropopause folding (or duplication) in spring (Northern Hemisphere) and its role in the STE^43^. See Figure S7 for flowchart representing step-by-step the process shown in (**a**,**b**). (**c**) The beryllium-7 concentration profile from Delaygue *et al*.^26^ shows the relationship between tropopause height (blue line) and beryllium-7 concentrations, indicated by isolines^26^.

For mid-latitude stations, this means that tropopause heights during summer time have increased. While the intertropical convergence zone (ITCZ) and the Polar-Ferrel convergence zone (PFCZ) exhibit an upward air flux, see Fig. 1(a,b), the Hadley-Ferrel Interface zone (HFIZ) exhibits the strongest stratosphere–troposphere exchange (STE) with a net downward flux of air bringing beryllium-7 to the surface, thereby allowing the tracking of its position and latitudinal progression^26,30,39^.

HFIZ is the downward branch of the Hadley cell where the highest level of beryllium-7 activity concentrations reaches the ground surface and leaves a clear signature of the HFIZ position (Figure S2)^30^. The position and speed of cells refers to the seasonal northward and southward movement of the atmospheric circulation cells, especially the tropical cell, following the sun from June solstice to December solstice, and the speed of this movement, respectively. While the astronomical speed is always the same, the true propagation speed shows inter-annual variability, indicated, for example, by different starting and ending dates of the Monsoon circulation in various parts of the world.

The analysis of the beryllium-7 time series provides important information on all four (b, c, d, e) aspects of global atmospheric circulations mentioned above. With this simple but previously unconsidered method, it is possible to track the atmospheric processes that are predicted to be modified as a consequence of global warming trends. Results from previous studies based on global reanalysis datasets such as ERA-Interim, JRA-55, and MERRA are available to monitor global circulation^40^.

Using radioisotope monitoring data as proxy for circulation anomalies is not preferable compared with direct observations but useful to complement such studies.

The model presented in Fig. 1 is simplified. Ferrel circulation is indirect^41,42^ and HFIZ is the downward branch of the Hadley cell. However, in the interface zone between the Hadley cell and the westerlies most of the downward mixing of beryllium-7 takes place. This is generated by the downward branch of the Hadley cell, and in particular by the subtropical jet (STJ) that mix stratospheric air from the lower mid-latitude troposphere into the significantly higher tropical troposphere (troposphere folding). In the tropical zone, the generally sinking air motion makes it much more likely that air reaches ground level and thus the monitoring stations, compared with the polar front jet (PFJ) where the beryllium-rich air is frequently only reaching the mid-troposphere. So, the STJ in combination with the large-scale subsidence within the downward branch of the Hadley cell is the region where the highest beryllium-7 concentrations are measured.

As reported in IPCC AR5^25^, there is high significance that the tropopause height increased in the subtropics and in northern high latitudes. Due to the broadening of the tropical belt, especially during summertime, the area affected by the higher subtropical tropopause also increases. This is visible in the beryllium-7 data. Decreasing tropopause height trends in other latitudes are less certain.

### Production and transport of cosmogenic radioisotopes

Beryllium-7 with a half-live of 53.2 days is particularly suitable as an atmospheric tracer^43,44^. This isotope is produced by the spallation of oxygen and nitrogen due to cosmic radiation in the upper troposphere and lower stratosphere (UTLS) region. Once created, the radionuclides attach to aerosols and are subject to atmospheric circulation until they decay. Currently, atmospheric concentration data can be readily obtained from ground level measurements by utilising the monitoring results from an existing global network of radionuclide stations^45^ (currently 71 in operations). These stations are part of the International Monitoring System (IMS) established and maintained by the Provisional Technical Secretariat of the Comprehensive Nuclear-Test-Ban Treaty Organization (CTBTO)^45^ located in Vienna, Austria. Air samples are continuously collected for 24 hours and then analysed for environmental radioactivity.

Datasets currently available from 70 global IMS monitoring stations cover up to the last 20 years and can therefore be used to analyse atmospheric characteristics and their changes for this time period^30,31,36^.

The continuous monitoring of cosmogenic beryllium-7 at these stations shows in the long-term a regular annual cycle, but with significant daily variations that amount to a factor of two or more. There are multiple causes for the variability of beryllium-7 activity concentrations in ground-level air^46–48^. Two main processes determine the abundance of these isotopes at lower altitudes: (1) the production rate in the UTLS region, and (2) the subsequent transport to the surface. At lower altitudes during horizontal transport, the beryllium-7 concentration may be diminished by dilution and rainout. There is a clear dependency between the transport processes investigated in this study and some modified atmospheric circulation patterns during the last decade^34^.

### Production through Cosmic Rays

The production of cosmogenic radioisotopes in the UTLS region is governed by incoming cosmic rays^35,46,48–51^. The incoming flux of cosmic rays is not constant but modulated by the approximately 11-year cycle of solar activity^52^. An increased solar activity as indicated by a higher number of sunspots is accompanied by strong magnetic fields that retain charged particles. Thus, the flux of cosmic rays reaching the Earth, is anti-correlated with the number of sunspots^52^.

Since an increased influx of cosmic rays leads to a proportionally increased spallation activity, an analysis of the radionuclide concentrations at ground level with focus on atmospheric circulation must include a correction against these variations in the production. In a comparison of the annual averages of ^7^Be concentrations with the annual average of cosmic rays, only 18 out of the 62 stations studied in this paper show a significant positive correlation (α = 0.05) for the years 2004 to 2018. The other stations show weak positive (37 stations) or even negative (7 stations) correlations not significant at the α = 0.05 level. At these stations the correlation is overprinted by atmospheric processes and due to the mixing of old and fresh air masses during transport^34,35^.

**Figure 2:**
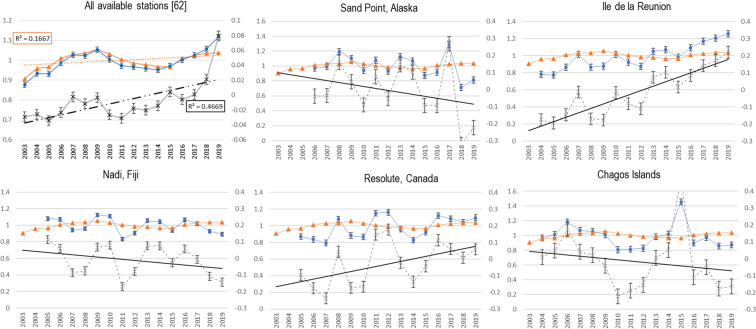
Examples of the correlation between yearly averages of beryllium-7 and cosmic rays (orange line). Stations with different trend are shown. First plot on the left displays all beryllium-7 normalized yearly average concentrations (blue line) which is the result of all 62 stations records. The global analysis clearly indicates the influence of cosmic rays; therefore, data needed to be corrected (grey line, secondary axis) to assess the weight of atmospheric circulation processes such as tropopause breathing, tropopause height variation etc. which are visible at each location. Cosmic rays cycle dataset (IGY detector) is taken from http://cosray.unibe.ch/ (2019). Normalization means each absolute value is divided by the overall average. Cosmic correction is based on the subtraction of normalised cosmic dataset to the normalised Beryllium-7 dataset. Error bars represent standard error. Supplementary files include the complete set of stations information and charts.

After linear correction for the cosmic ray variations, the remaining variability reflects the influence of atmospheric processes and long-term trends thereof, see Fig. 2.

Looking at the corrected time series in Fig. 2, two effects are highlighted: the troposphere multi annual variation^17^ (up and down movement of grey dotted line) and the overall trend towards a heightening or lowering of the tropopause (continuous black line).

Tropopause instability show different frequencies and patterns depending on the station location. As mentioned by Hakuba *et al*.^17^ the troposphere multi annual variation is influenced by seasonal, inter-annual, multi-annual variations associated with Brewer Dobson Circulation, Quasi Biannual Oscillation (QBO), El Nino Southern Oscillation (ENSO), etc.

Besides the 11-year solar activity cycle, a systematic decrease of the Earth’s magnetic field strength and a corresponding increase in cosmogenic radiation is being observed over the last 150 years^53^. This effect accounts for a systematic increase of only +0.4% in the beryllium-7 production over a decade^54,55^ which is taken into account by the above described correction for the measured cosmic ray flux.

### Transport through atmospheric processes

The second process, which is the transport of beryllium-7 from the UTLS region to the surface, is mainly governed by the systematic vertical downward movement taking place within the Hadley-Ferrel Interface Zone (HFIZ)^30,36,43^. As shown in Fig. 1(a,b), the HFIZ features the downward movement of air masses, while the tropical and polar interface zones feature convergence and upward movements. The HFIZ follows an annual cyclic latitudinal movement due to the poleward expansion of the Hadley cell during summer seasons in the respective Hemispheres^56^.

Since the isotopes are attaching to particles, the effective radionuclide downward transport is also dependent on the stratospheric aerosol loading^26^. The aerosol load is variable depending on external factors; e.g., it can increase by more than two orders of magnitude following large volcanic eruptions. The day-to-day variations were smoothened out by the postprocessing of the data, as described in the next section. Longer time scales, as in the focus of this work, are more robust against such fluctuations.

Taking all these dependencies and variations into account, the observed trends in the cosmogenic radionuclide concentrations measured at ground level can be used as proxies to investigate changes in the global tropospheric circulation possibly triggered by climate change effects^25^.

### Concentrations of cosmogenic isotopes at ground level

The measurements of beryllium-7 activity concentrations exhibit significant daily and weekly fluctuations^44,47,57,58^. Time series were post-processed with a moving average of 120 days to smooth out these fluctuations and normalized to make data more comparable (supplementary file Dataset1).

**Figure 3:**
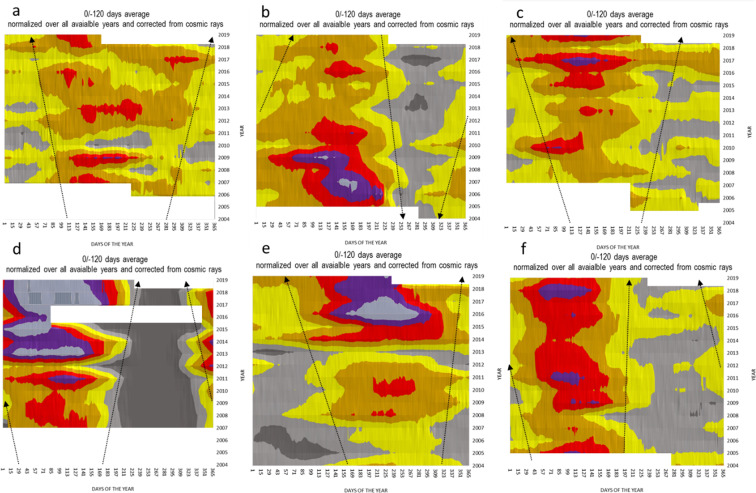
Time series of the annual beryllium-7 concentration time series over the years at selected monitoring stations. Most long-term data sets of beryllium-7 concentration, like a, c, d, e, and f (Canada, Hawaii, Japan, Mongolia, Tristan da Cunha respectively), exhibited over the last two decades a broadening of the beryllium-7 season; i.e., the time period in which the beryllium-7 concentrations were above average. Some stations, like b in Florida and others in Zalesovo, Russia, and Rio de Janeiro, Brazil (not shown here), showed an opposing trend. Others like Cook Island and Guam (Pacific Islands located at low latitudes below 20 degrees) did not show much of a significant change over the past 16 years with a standard deviation of 5 to 6% for the whole time series. (supplementary file Table S1). The black arrows indicate the trends; they are only for visual guidance. White areas indicate periods of n/a data.

Concentration data from 70 radionuclide monitoring stations were analysed. Some time series go back as far as 2000; other stations were later established, but only stations (62 stations) with at least seven years of data starting from 2003 were considered for this study (Table S1). In total, over 270,000 radionuclide samples were selected. Most of the samples analysed contained between 1000 and 9500 µBq/m³ of beryllium-7. Figure 3 shows time series of the averaged and normalised concentration data for the selected stations, with the orange and darker bandwidths indicating above-average beryllium-7 concentrations. Normalisation was obtained by dividing absolute values with the overall timeseries average. Stations exhibit phases of above-average concentrations followed by phases of below-average concentrations, resulting in a sinusoidal time series with a period of one year. The time series from stations in opposing hemispheres are phase-shifted by six months due to the simultaneous extension and contraction of the Northern and Southern Hemisphere Hadley cells^31,36^. This annual variation pattern is particularly visible in mid-latitudes where the stations are strongly affected by the HFIZ movement (Figure S2). For a given monitoring location in the mid-latitudes, the concentration rises when the HFIZ approaches from the equator and falls when the HFIZ retreats towards the equator. This effect is even visible at monitoring locations not directly below the HFIZ pathway due to the horizontal transport of air masses at lower altitudes (supplementary file surface charts).

Stations located in mid-latitudes, like. Canada and Japan, showed a clear pattern, namely a gradual extension of the high beryllium-7 season (Fig. 3). The annual period with above-average concentrations starts earlier and ends later in the season. Similar patterns were found especially along the sub-tropical zones in both hemispheres.

The station in Resolute, Canada, showed lower levels of beryllium-7 and the transit time to reach the yearly maxima increased over the years, which indicates that the atmospheric circulation and trade winds are weakening. Another example, the station in Stockholm, Sweden, showed a 40% net increase over the past three years as well as 54 additional days with concentrations above average. In general, most stations showed a similar increase in the beryllium-7 concentrations.

### Changes in the tropopause height

**Figure 4:**
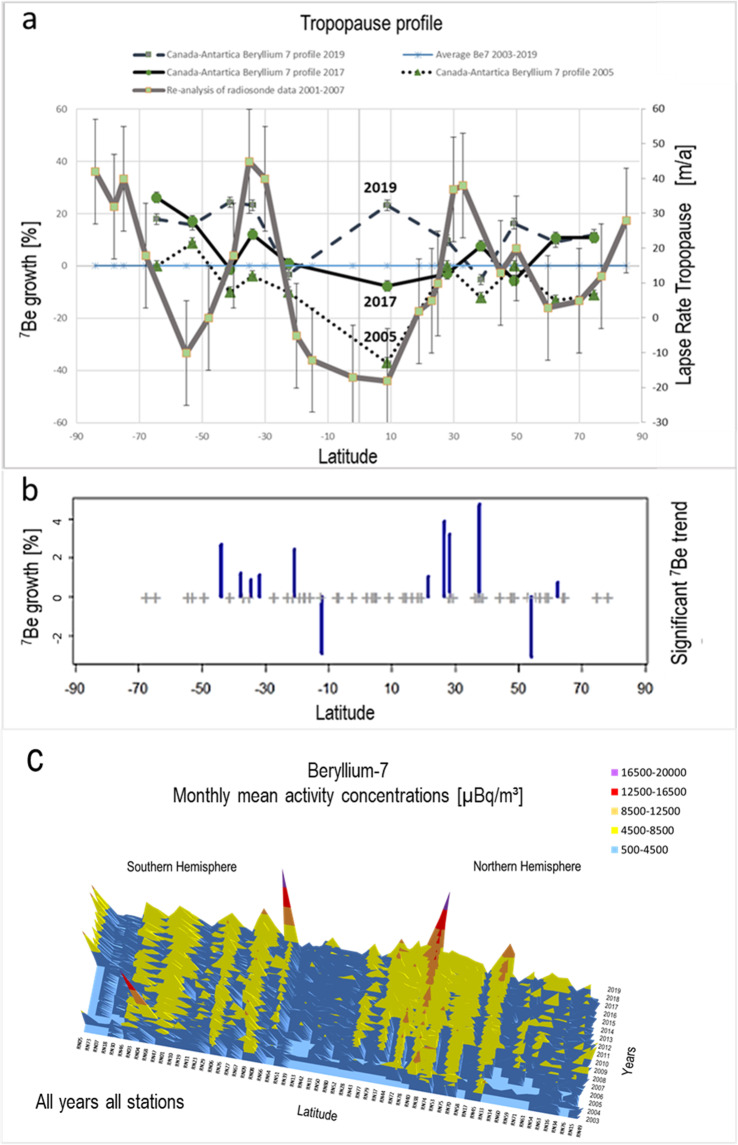
(**a**) The relative increase/decrease of beryllium-7 concentrations for varying latitudes correlate with re-analysis measurements of the tropopause height trends. The beryllium-7 data was based on measurements from 2003 to 2019; the re-analysis data was based on aggregated measurements from 2001 to 2007 (Schmidt *et al*.)^20^. The applied error margin was 2% based on data uncertainties for the beryllium-7 concentration; for the re-analysis of the radiosonde data, it was ±15 m/a based on the aggregation of data in different latitudinal resolutions of 5°–10° (Schmidt *et al*.)^20^. The 2019 beryllium-7 profile is especially interesting because of its uncommon increase at the equator, possibly indicating a change in the tropopause profile and a connection to why hurricane Dorian (August 2019) was so powerful and slow at the same time. (**b**) Annual beryllium-7 growth for stations with significant (α = 0.05) trends (2004–2018). Plus signs indicate the position of all stations included in this study. (**c**) beryllium-7 monthly mean from all stations for all available years.

The tropopause is the thermally stable boundary layer between the troposphere and the stratosphere. The tropopause height decreases from about 17 km in the equator region to about 7 km in the polar region. Observations have demonstrated that the tropopause height has been changing over the recent years, due to climate change processes^5,22,59^. Between 2001 and 2007, a height increase of 20–50 meters per year (Fig. 4) has been measured at around 30–40 degrees latitude and the poles, while a decline of 10–30 meters per year has been measured around the equator and 60 degrees latitude^20,59–61^. However, these observations, obtained using reanalysis data, were not evenly distributed globally, were conducted for a limited time and are therefore subject to significant uncertainty.

The beryllium-7 concentrations rise with altitude in the troposphere and then increase sharply above the tropopause^26,32^. Therefore, an increase in the tropopause height integrates areas of enhanced beryllium-7 concentrations into the troposphere. As vertical mixing within the troposphere is much stronger compared with troposphere-stratosphere exchange, this causes an increase of the amount of beryllium-7 being transported to the surface, see Fig. 1c.

Figure 4a displays the troposphere height trends measured with reanalysis data^20^ in comparison with the growth rate of the beryllium-7 concentrations at the ground level for different latitudes. The relative growth and decline of a latitudinal profile for beryllium-7 (2017) and re-analysis (2001–2007) datasets shows a correlation of 0.8, indicating a causal relationship between the beryllium-7 trends and the tropopause height changes.

Tropopause height, is defined by a thermodynamic gradient. The tropopause height increases if the critical temperature lapse rate moves to higher altitudes. Though this is accompanied by some air mass movement, the production of beryllium-7 is fairly robust against these changes and mainly depends on the height from ground level.

Tropopause trends presented here are in agreement with the work of Santer *et al*.^59^. A global interpolation of beryllium-7 concentration from 2003 to 2019 as troposphere multi annual variation or tropopause instability is available as supplementary file.

The datasets from re-analysis data and beryllium-7 are from different periods as data from 2001 to 2007 are not available for all the stations needed to build the latitudinal profile shown in Fig. 4a.

Figure 4b shows the beryllium-7 growth rate of all stations with a significant (α = 0.05) linear trend over the past 7–14 years compared to re-analysis data^20^. The highest increases are found at mid-latitudes corresponding with the locations of tropopause heightening. Thirteen out of 62 stations show a significant (α = 0.05) linear correlation between beryllium-7 and calendar year. Eleven of these stations show a positive correlation with the steepest increases of 7Be being observed at Okinawa, Azores, and Midway Islands. Two stations (Cocos Island and Zalesovo) show a significant negative correlation. Beryllium-7 trends from all stations, all available years is shown in Fig. 4c.

Global warming is also associated with decreasing lower-tropospheric temperature differences between the equator and the polar region (enhanced polar warming), which may cause a weakening of the geostrophic (westerly) circulation. Furthermore, it affects the poleward movement of the HFIZ. While the former may lead to more stable weather patterns, such as the blocking situations discussed below, the latter extends the time period as well as geographical area where high beryllium-7 concentrations are observed. The growth is greater in the Southern Hemisphere and lesser in the Northern Hemisphere, as indicated by the data. In general, the global beryllium-7 data correlated with re-analysis results.

Therefore, global beryllium-7 monitoring provides an opportunity to support the tropopause height monitoring efforts. More information on the role of intensified STE associated with this process can be expected from sodium-22/beryllium-7 ratios. The 19-year time series from Switzerland shows an overall positive trend (i.e. increasing contribution of stratospheric air), however, with relatively high year to year variability (Figure S2).

**Figure 5:**
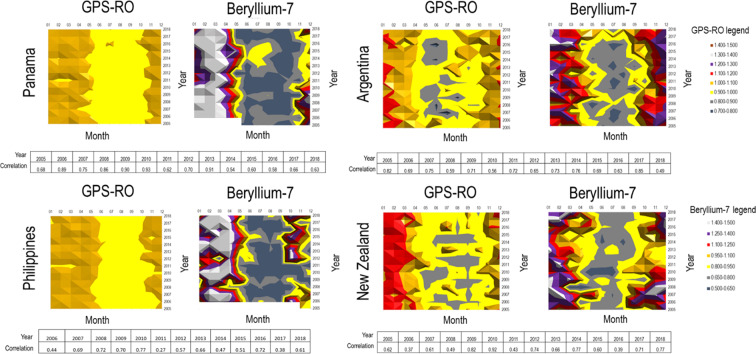
Shows examples of IMS station locations from all regions of the worlds where we compared tropopause profile with beryllium-7 data. Tropopause profile is built on monthly average records of GPS-RO [m] (left plot) and beryllium 7[µBq/m³] (right plot). GPS-RO data have the same GPS coordinates as IMS stations locations. GPS-RO are retrieved temperature profiles generated by satellites. All available years are plotted. The coloured scale is based on normalised data obtained by dividing absolute values with the overall timeseries average. White areas indicate periods of n/a data.

Figure 5 shows tropopause height calculated from gridded monthly global positioning system radio occultation (GPS-RO) data^62,63^ versus the beryllium-7 concentrations at the same location (equal coordinates). The post processing applied to create the chart is mean annual records. In both cases GPS-RO and beryllium-7 data are not smoothed additionally. Each location shows a high degree of correlation between the 2 parameters confirming connections between tropopause and beryllium-7.

Standard deviation is low at the mid latitudes and increases at the equator and at the very high latitudes, those areas where beryllium-7 is most affected by wash out. Years with lower correlation values need to be further investigated as possibly associated to additional drivers such as advection or stratospheric tropospheric exchange.

### The slowdown and poleward movement of the Ferrel cell

Changes of atmospheric cell progression are also visible through isotopic ratio changes over time. As seasons have characteristic isotopic ratios due to the associated position of the circulation zones, isotopic ratios of sodium-22 and beryllium-7 can also indicate the circulation trends^64^. Sodium-22 has similar properties as beryllium-7 except that is formed by spallation with Argon but has a significantly longer half-life (2.6 years). Figure S3 displays how much the radioisotope ratio seasonal gradient (^22^Na versus ^7^Be) varies over time, meaning that a seasonal peak is expression of fast transition of the atmospheric cell convergence zone over the point of measurement (IMS Station). A flattening of the ratio indicates a slowdown of the transition.

The resulting graphs (Figure S3) with the squared ratio of sodium-22 and beryllium-7 depicted a slow velocity of the isotopic ratio gradient over time, suggesting a weakening of the circulation.

The time series of the beryllium-7 concentrations (and sodium-22 for figure S3) were clearly affected by the changing seasonal movement of the HFIZ. Along with the increasing height of the tropopause, the Hadley cell expands not only vertically but also in the latitudinal direction, accompanied by a possible slowing down of the Ferrel circulation. Zhang and Song provide evidence that reduction of surface pressure gradient support predictions of weakening of vertical circulation in a warmer climate, including the weakening of trade winds^65^. Over the years, this effect was reflected in the time series by a prolongation of the periods with high isotope concentrations (supplementary file Table S3). Most of the locations followed this pattern, as indicated by the black dotted lines in Fig. 3.

The ongoing extension of the high beryllium-7 season is, therefore, highly likely directly associated with longer and warmer summers. The Hadley cell is susceptible to temperature effects as it is driven, like the polar cells, by the thermal circulation of warm and cold air and the creation of high- and low-pressure areas. The poleward extension of the HFIZ movement, (together with a widening of the tropical circulation, poleward shift of the tropospheric jet streams) can be linked to global temperature increase issues^4,7,8,22,46,65^.

### Static weather situations

**Figure 6:**
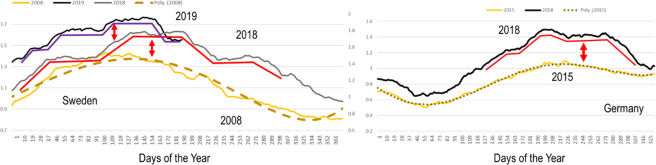
Left: The station in Stockholm, Sweden (see extended data file for station details), is a good example for beryllium-7 growth, extension of the warmer period, and plateau features, with a transition from a rather smooth, sinusoidal curve (2008, continuous yellow line with dotted sine trend line) to steps and plateaus (2018, continuous grey line and 2019, continuous black line). Right: Freiburg, Germany, showed similar patterns (red continuous line). The weather in Stockholm during May and June 2018 which correspond to the middle plateau feature (left plot) was persistently hot and sunny with peaks of 29 degrees Celsius.

Comparing the annual curves of beryllium-7 at a given station indicated that the observed shape of the curves has been significantly changing over the years. While in the early years of the available data sets—i.e., the 2000s—the curves showed rather smooth and steady increases and decreases, closely resembling an idealized sine curve, the more recent data showed plateaus where the beryllium-7 concentration stayed roughly constant for a few days or weeks per year (see Fig. 6).

The beryllium timeseries in both Sweden and Germany show the transition from sinusoidal (dotted line) to plateau (continuous red - purple line). In terms of HFIZ movement the sinusoidal curve indicates a smooth progression northward in summer and southward in winter. Once the timeseries flattens out into a plateau pattern we can translate it as stalling of the HFIZ.

Figure 6 shows two important effects: Heightening of the tropopause with higher concentrations in 2018 and 2019 with respect to previous years (Year 2008-Sweden, Year 2015-Germany), and change in the sinusoidal progression of the HFIZ. The tilting of the Earth axis makes the progression of the HFIZ follow a sinusoidal function (Year 2008, Fig. 6). With decreasing temperature differences between the equator and mid-latitudes, the circulation slows down, and we see higher frequencies of stalling situations, plateaus, or step features where the interface zone seems to pause its latitudinal movement^6,10,65,66^. These stalling conditions facilitate static weather patterns which correlate with the general trade wind deceleration^10,12,14^ and the increase of convective storms at mid-latitudes over the past few years^1,2,9,10,13^.

A warmer and higher tropopause along with a weaker westerly circulation would cause more tropical weather during summertime as well as more steady weather situations. Longer episodes of large-scale atmospheric blocking and high-pressure areas with low pressure gradients have been frequently observed globally over the last few years.

Atmospheric blocking is a phenomenon that leads to a static weather pattern lasting from one to several weeks, often associated with extreme weather events - heat waves in summer and cold waves in winter. Frequently, the onset of a block starts with the rapid poleward transport of subtropical air. Weather and climate models have a tendency to underpredict blocks and to underestimate their duration^3^. It is so far not well established whether blocks are becoming more or less frequent under global warming conditions, although regional increases in blocking conditions have been identified^25^.

The fraction of economic losses caused by severe convective storms in Europe with respect to all-natural hazards combined went from 26% between 1989 and 2017 to almost 40% between 2003 and 2017^11^. The trend towards persistent static weather patterns is in agreement with the observations (plateau features) at the German and Swedish stations (Fig. 6).

A majority of the stations showed an extended high-beryllium-7 season, as shown by examples in Fig. 6. By calculating how much time it takes to reach its yearly maximum at each station (which is the peak of beryllium-7 concentration per year when HFIZ is closest), it is possible to check whether the progression speed of the convergence zone has changed. Between 2003 and 2019, the progression clearly slowed down. The average of the beryllium-7 growth and extension period of growth for the past three years in all the stations indicated a prolongation of the summer season by around 1 month and a generalised deceleration (slower transit time) of circulation, producing a delay of 3 weeks to reach the beryllium-7 maximum worldwide (Figure S3). Figure S4 shows the increase in the number of days required per year to reach the maximum threshold from a baseline of the yearly mean. The trend is positive which indicates a weakening of cell progression and therefore suggest a slowing down of the general circulation.

**Figure 7:**
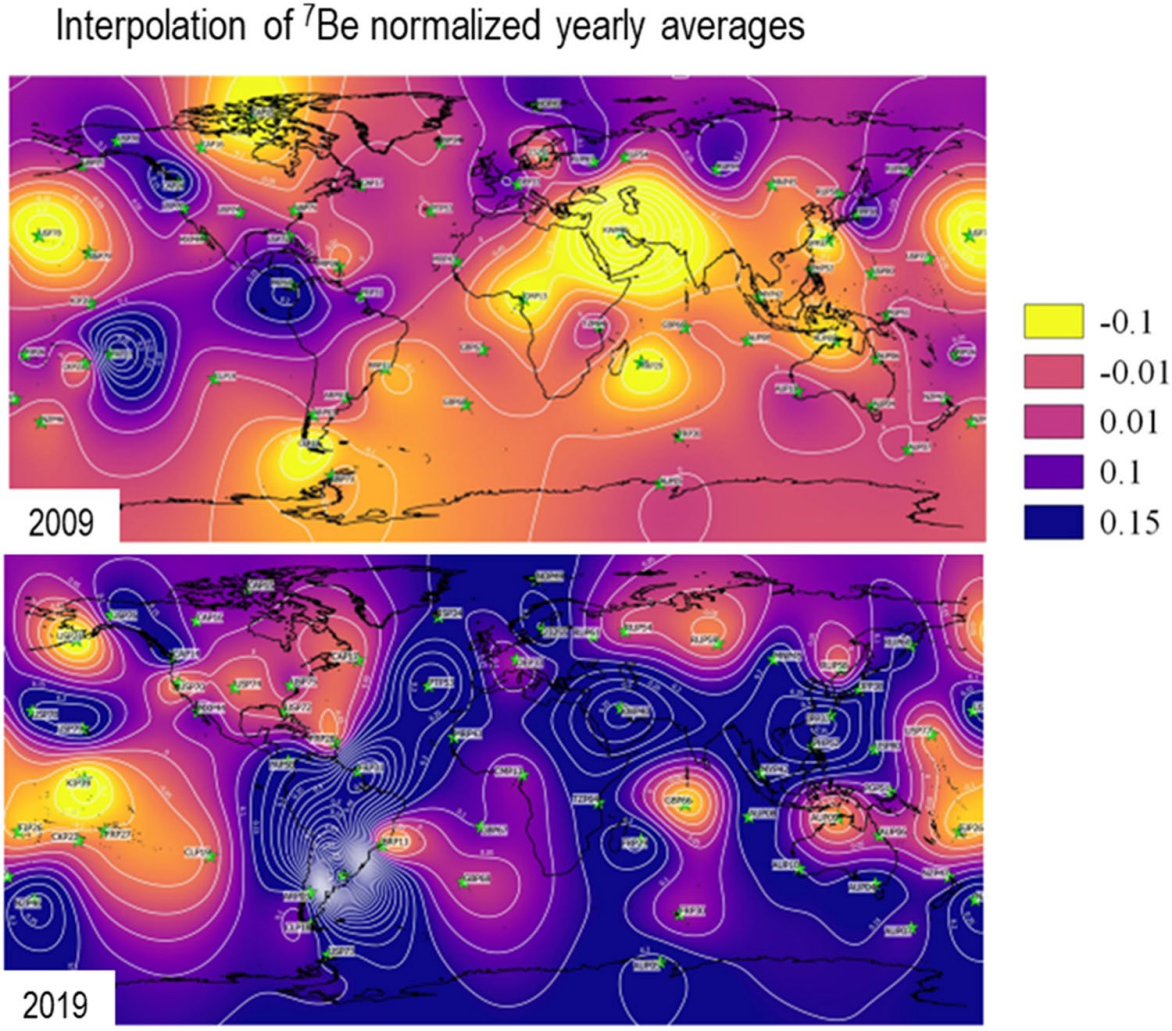
Yearly normalized averages of beryllium-7 data for 2009 and 2019 corrected from cosmic rays. The interpolation does not contain meteorological information and is only to help visualize the global increase of beryllium-7 concentration in the last 10 years. Interpolation is based on multilevel b-spline with cell grid size of 1 degree using beryllium-7 values from 62 IMS stations (green stars). Software used to produce Figure 7 QGIS Development Team (2019). QGIS Geographic Information System. Open Source Geospatial Foundation Project. http://qgis.osgeo.org.

As discussed above, the trends of increasing beryllium-7 concentrations (Fig. 7), extended periods of high concentrations and growing transit times can be best explained by changes in large-scale atmospheric circulation patterns, possibly associated with the continued increase in greenhouse gas concentrations^27,28^.

All years are available in the supplementary file but we chose to select 2 figures with 10 years difference to highlight how much the concentration increased over time. Moreover, both plots correspond to solar maxima where we should have the minimum levels of beryllium-7 concentration. The maps do not have any meteorological information rather they show only interpolation of beryllium-7 values.

Initial analysis on GPS RO scale allowed to build a preliminary comparative scale between the temperature and increase of beryllium-7 as proxy. The comparative scale built on the ratio of absolute monthly tropopause height versus absolute monthly beryllium-7 values. The average ratio is between 3 and 6: lower (~3) at the mid latitudes and highest (~6) at the equator where the height of tropopause is the highest and the wash out effect is stronger (Fig. 5).

In fact, if beryllium-7 observation data would be assimilated, weather models with integrated simulation of beryllium-7 production could assess the precision of this monitoring method. Furthermore, longer time series are needed to better distinguish between decadal-scale and longer-time cyclic circulation variations and the systematic effect of global warming.

### Summary and conclusions

Radioisotopes like beryllium-7 and sodium-22 are produced by the incoming cosmic radiation in the UTLS region. Their concentrations at ground level depend on the tropopause height and prevailing atmospheric circulation and are therefore a function of the measurement location, mostly the station latitude, and the time of the year. The activity concentrations are significantly influenced by systematic changes in the atmospheric circulation. Such ground-level concentration data can thus yield invaluable insights into key atmospheric processes, which are assumed/predicted to be affected by global warming.

Beryllium-7 data measured over the last 15–20 years confirm that major changes in the atmospheric circulation are currently ongoing. Even though timeseries are too short to make climatological assessments, these changes may well be triggered by progressive global warming. Natural causes to explain the observed beryllium-7 variations such as solar flux or the strength of the Earth’s magnetic field are relevant but act on either a different order of magnitude or at different time scales and thus cannot explain the trend we observe.

This new monitoring method using beryllium-7 as a proxy for global atmospheric circulation supports ongoing research related to changes in tropopause heights as well as global circulation patterns, which includes some topics marked as “known with lower confidence” in the latest report of the Intergovernmental Panel on Climate Change (IPCC)^25^. Although data sets cover less than two decades, our method provides some indication on how strongly global warming may influence the atmospheric circulation worldwide already today. This may also indicate that the warming trend is more severe and rapid than many climate projections show, and that we cannot exclude that weather extremes predicted to occur between 2050 and 2100 already materialize during the next few decades.

## Supplementary information

Extended Data File.

Supplementary Dataset.

Supplementary Information: Beryllium-7 maps.

Supplementary Movie 2.

Supplementary Information: Beryllium-7 trends.

Supplementary Movie 1.

Supplementary Information Guide.

## Data Availability

The data that were used for the current study and that support its findings are not publicly available, but access through the virtual Data Exploration Centre (vDEC) can be granted by the CTBTO via a cost-free confidentiality agreement. The application for vDEC access to data can be submitted for approval through a simple web form at https://www.ctbto.org/specials/vdec/.
