# Supplementary material for: Radioisotopes demonstrate changes in global atmospheric circulation possibly caused by global warming
Source: Sci Rep. 2020 Jul 1;10:10695. doi: 10.1038/s41598-020-66541-5 (PMC7329870; doi:10.1038/s41598-020-66541-5)
Supplement: Supplementary file 1 — Extended Data File. [file 41598_2020_66541_MOESM1_ESM.pdf]

Extended Data

Title: Radioisotopes demonstrate changes in global atmospheric circulation possibly caused by global warming

Authors: Lucrezia Terzi[a,b*], Gerhard Wotawa[c], Michael Schoeppner[d,e], Martin Kalinowski[d], Paul R.J. Saey[b],Philipp Steinmann[f], Lan Luan[g] and Paul W. Staten[g].

Affiliations: [a]Belgian Nuclear Research Centre (SCK•CEN), Mol, Belgium; [b]Technische Universität Wien, Atominstitut, Austria; [c]Zentralanstalt für Meteorologie und Geodynamik (ZAMG), Vienna, Austria, [d]Provisional Technical Secretariat, Preparatory Commission for the Nuclear-Test-Ban Treaty Organization, International Data Centre, Vienna, Austria; [e]Institute of Safety/Security and Risk Sciences, Vienna, Austria; [f]Federal Office of Public Health (BAG), Bern, Switzerland; [g]Indiana University Bloomington, Bloomington, Indiana, USA.

***Corresponding Author:**
Lucrezia Terzi
Ph: +43 6644553833
e-mail: lucrezia.terzi@sckcen.be
[a] SCK•CEN, Belgium Nuclear Research Centre,
Boeretang 200, 2400 Mol, Belgium.
[b] Technische Universität Wien, Atominstitut,
Stadionallee 2, 1020 Wien, Austria.

Supplementary Tables

***Table S1*. International Monitoring System Stations (www.CTBTO.org)**

A

Total n. of stations	62
Total n. of samples	268752
Average timeseries length (n. of years)	12
Minimum timeseries length (n. of years)	7
Maximum timeseries length (n. of years)	16
Outliers filtered out (single spikes above factor of 2 over overall average). Filtered data averaged over 0/-120 days and normalized over overall average (2003-2019).	

B

Station code	Treaty number	State	Location	Latitude	Longitude	N. of samples from 2003-2019 (5967 days)
ARP01	RN01	Argentina	Buenos Aires	-34.54	-58.47	5784
ARP03	RN03	Argentina	Bariloche	-41.15	-71.16	4563
AUP04	RN04	Australia	Melbourne, VIC	-37.73	145.10	5776
AUP05	RN05	Australia	Mawson, Antarctica	-67.60	62.87	2387
AUP06	RN06	Australia	Townsville, QLD	-19.25	146.77	5476
AUP07	RN07	Australia	Macquarie Island	-54.50	158.95	2673
AUP08	RN08	Australia	Cocos Islands	-12.19	96.83	5342
AUP09	RN09	Australia	Darwin, NT	-12.43	130.89	5291
AUP10	RN10	Australia	Perth, WA	-31.93	115.98	5410
BRP11	RN11	Brazil	Rio de Janeiro	-22.99	-43.42	5405
CMP13	RN13	Cameroon	Edea	3.78	10.15	3450
CAP14	RN14	Canada	Sidney	48.65	-123.45	3381
CAP15	RN15	Canada	Resolute, NU	74.71	-94.97	4089
CAP16	RN16	Canada	Yellowknife, N.W.T.	62.48	-114.47	5668
CAP17	RN17	Canada	St. John's N.L.	47.59	-52.74	4592
CLP18	RN18	Chile	Punta Arenas	-53.14	-70.88	4685
CLP19	RN19	Chile	Hanga Roa, Easter Island	-27.13	-109.35	3582
CKP23	RN23	Cook Islands	Rarotonga	-21.20	-159.81	5467
FJP26	RN26	Fiji	Nadi	-17.76	177.45	4775
FRP27	RN27	France	Papeete, Tahiti	-17.57	-149.57	4410
FRP28	RN28	France	Pointe-à-Pitre, Guadeloupe	16.26	-61.53	3844
FRP29	RN29	France	Réunion	-20.91	55.59	4575
FRP30	RN30	France	Port-aux-Français, Kerguelen	-49.35	70.26	4170
FRP31	RN31	France	Kourou, French Guiana	5.17	-52.69	4453
DEP33	RN33	Germany	Schauinsland/Freiburg	47.92	7.91	4144
ISP34	RN34	Iceland	Reykjavik	64.13	-21.90	4185
JPP37	RN37	Japan	Okinawa	26.50	127.90	4342
JPP38	RN38	Japan	Takasaki, Gunma	36.30	139.08	4683
KIP39	RN39	Kiribati	Kiritimati	2.01	-157.39	3200
KWP40	RN40	Kuwait	Kuwait City	29.34	47.91	3718
MYP42	RN42	Malaysia	Tanah Rata	4.48	101.37	3572
MRP43	RN43	Mauritania	Nouakchott	18.14	-15.92	4206

MXP44	RN44	Mexico	Guerrero Negro, Baja California	27.96	-114.06	2654
MNP45	RN45	Mongolia	Ulaanbaatar	47.89	106.33	5288
NZP46	RN46	New Zealand	Chatham Island	-43.82	-176.48	4827
NZP47	RN47	New Zealand	Kaitaia	-35.07	173.29	5614
NOP49	RN49	Norway	Spitsbergen	78.23	15.39	4215
PAP50	RN50	Panama	Panama City	8.98	-79.53	4863
PGP51	RN51	Papua New Guinea	Kavieng, New Ireland	-2.58	150.81	3902
PHP52	RN52	Philippines	Tanay	14.58	121.37	4706
PTP53	RN53	Portugal	Ponta Delgada, São Miguel, Azores	37.74	-25.70	2422
RUP54	RN54	Russian Federation	Kirov	58.59	49.41	2961
RUP58	RN58	Russian Federation	Ussuriysk	44.15	132.00	3277
RUP59	RN59	Russian Federation	Zalesovo	53.94	84.79	3315
RUP60	RN60	Russian Federation	Petropavlovsk-Kamchatskiy	53.05	158.78	3472
RUP61	RN61	Russian Federation	Dubna	56.74	37.25	3840
SEP63_	RN63	Sweden	Stockholm	59.41	17.95	4814
TZP64	RN64	United Republic of Tanzania	Dar es Salaam	-6.78	39.20	4003
GBP66	RN66	United Kingdom of Great Britain and Northern Ireland	BIOT/Chagos Archipelago	-7.28	72.37	4290
GBP67	RN67	United Kingdom of Great Britain and Northern Ireland	St. Helena	-15.94	-5.67	3769
GBP68	RN68	United Kingdom of Great Britain and Northern Ireland	Tristan da Cunha	-37.07	-12.31	4974
USP70	RN70	United States of America	Sacramento, CA	38.67	-121.36	5030
USP71	RN71	United States of America	Sand Point, AK	55.34	-160.49	4361
USP72	RN72	United States of America	Melbourne, FL	28.10	-80.65	5060
USP73	RN73	United States of America	Palmer Station	-64.77	-64.05	4553
USP74	RN74	United States of America	Ashland, KS	37.17	-99.77	5319
USP75	RN75	United States of America	Charlottesville, VA	38.00	-78.40	5324
USP76	RN76	United States of America	Salchaket, AK	64.67	-147.10	4449
USP77	RN77	United States of America	Wake Island	19.29	166.61	3882
USP78	RN78	United States of America	Midway Islands	28.22	-177.37	3722
USP79	RN79	United States of America	Oahu, HI	21.52	-157.99	4721
USP80	RN80	United States of America	Upi, Guam	13.57	144.93	3827

***Table S2*. For each station the ^{7}Be % of yearly growth versus the overall average is calculated (the growth is based on 1.001 increase of the normalized averaged timeseries).**

% values are rounded to zero decimals. Dataset is based on 120 days averaged normalized ^{7}Be concentration corrected from cosmic rays.

Treaty number	2003	2004	2005	2006	2007	2008	2009	2010	2011	2012	2013	2014	2015	2016	2017	2018	2019
RN01	-16.68	-8.53	-0.32	-0.71	-5.06	-4.00	1.21	-3.48	-2.75	-2.62	3.90	-3.00	2.76	5.29	9.20	5.89	18.91
RN03		-18.18	-6.14	10.17	20.95		-2.48	-0.85	-3.55	2.98	-10.13	-14.54	0.03	3.63	-3.86	1.34	20.62
RN04	-15.95	-8.59	-13.42	-11.41	2.01	-1.76	0.76	10.98	-1.76	-3.53	2.89	0.06	-0.51	8.08	9.41	6.88	15.85
RN05											-4.46	-4.75	-8.62	-5.40	-5.44	10.12	18.55
RN06	11.71	2.68	2.41	0.03	-3.43	-8.59	-2.45	-6.60	-21.72	-10.12	-4.34	-0.48	2.47	12.86	15.59	11.24	-1.24
RN07										-2.88	-6.10	0.81	4.28	-3.30	-1.70	-1.06	9.95
RN08	50.12	49.60	19.69	7.29	0.19	-11.33	-6.20	-9.29	-6.36	-20.46	-25.38	-12.76	-4.39	-26.61	-19.15	2.19	12.86
RN09	18.45	-2.69	1.90	10.11	0.36	17.05	-13.60	-0.03	-19.02	-5.19	-0.65	0.49	1.99	0.30	-1.39	3.85	-11.94
RN10	-22.13	-21.03	-11.23	-8.20	0.88	1.05	5.05	13.64	5.39	7.76	2.10	1.37	4.07	3.24	4.93	0.45	12.66
RN11	18.66	-0.85	-6.59	6.84	0.66	8.63	-8.74	4.42	-3.20	-2.30	-0.71	-3.02	2.52	-1.08	-1.91	-5.80	-7.54
RN13						5.66	-15.52	7.09	15.33	-8.96	-3.72	3.49		-10.67	3.70	-1.74	5.34
RN14							20.37	-1.78	-16.78	-1.29	-13.17	2.03	11.70	-5.59	-7.37	-1.38	13.25
RN15			-6.77	-12.87	-21.80	7.91	-14.74	-15.03	17.04	20.62	1.46	-12.05	0.51	15.03	8.97	3.18	8.56
RN16	-15.09	-11.56	-9.67	2.52	3.77	-0.61	-5.03	10.79	2.38	4.41	-1.15	-1.69	1.36	5.28	7.92	1.42	4.95
RN17				-0.83	3.11	-6.20	4.98	-15.45	-6.79	6.88	4.14	2.47	1.42	3.30	0.80	6.23	-4.06
RN18	-9.43	-10.34	11.95		-13.03	-23.22	-21.01	-18.10	14.21	22.98	-10.46	-8.48	8.41	10.76	14.12	20.39	11.26
RN19	-11.64		-23.58	-8.34	-3.89				21.86	28.74	19.97	-7.33	0.09	5.12	-4.08	-9.61	-7.31
RN23	5.87	-7.16	0.66	-0.64	-0.21	-5.15	-1.78	2.72	4.37	-0.08	1.27	2.95	3.52	8.63	1.22	-6.71	-9.48
RN26			12.65	7.76	-7.03	-6.08	8.13	8.02	-17.07	-7.09	9.49	7.79	0.48	7.22	0.11	-10.45	-13.94
RN27	-7.68	-13.22	-20.29	13.27	18.78	33.50	34.40			17.23	-8.89	-9.87	-8.58	-8.76	-10.55	-13.22	-16.13
RN28			-7.38	3.23	13.74	8.51	-3.39		-13.59	-11.72	-0.02	7.07	2.72	0.28	-4.07	9.07	-4.46
RN29		-17.59	-17.58	-11.66	1.07	-15.04	-15.94	-2.13	-8.13	-9.53	10.21	11.32	5.78	10.76	16.10	18.66	23.70
RN30				6.98	-9.24	3.04	-0.24	-11.48	4.51	-0.42	-12.52		6.89	6.32	-2.46	4.92	3.70
RN31			-14.60	-1.00	-7.88	26.10	10.77	-13.94	-21.74	-17.85	-3.82	6.84	20.69	-13.72	-32.25	14.21	48.17
RN33					2.15	10.75	11.07	9.17	-4.41	-8.57	-18.02	-3.94	-10.82	-1.35	3.62	7.31	3.04
RN34	-10.41	10.08			-11.48	7.17		13.28	13.31	9.97	-4.65	-17.47	-13.21	-3.70	4.34	-10.23	13.00
RN37					-18.91	-14.66	-15.79	-18.94	-6.37	-22.84	8.56	4.27	-10.16	23.34	23.13	17.48	30.89
RN38	-3.13	-0.19	13.85	-6.83	1.17	16.31	13.23	-21.99	-10.95	-13.73	6.30	0.75	-7.06	-3.57	2.53	-3.48	16.79
RN39					24.06	-15.99	5.01	-6.22	-9.44	-6.15	48.94	-7.11	-16.67		2.41	5.12	-23.94
RN40		32.16	-1.71	-27.81	-7.40	-28.61	-46.47	-45.48	-6.98	15.15	-23.92		28.35	16.17	31.76	23.20	41.61
RN42							-7.28	-12.10	-9.31	-9.29	-1.10	1.91	18.80	19.26	-16.69	-4.27	20.07
RN43				-0.14	9.52	7.19	3.37	7.86	10.63	22.74	-17.22	-23.16	-21.94	-14.28	-3.77	4.30	14.90
RN44									-10.51	3.70	-10.25	-0.87	9.18	-1.52	-5.70	10.52	5.45

RN45	-16.28	-8.67	-23.79	-18.56	-18.39	0.44	-0.54	-0.69	1.80	-6.93	-20.47	15.24	9.92	28.14	14.84	18.04	25.91
RN46	-20.14	-3.24	6.06	-5.44	3.96	6.50	-3.24	-8.53	-8.45	7.54	5.49	0.74	-2.83	9.54	-3.84	-0.56	16.45
RN47	-22.43	-7.55	2.09	6.82	13.71	8.41	3.10	-11.82	-1.47	-2.62	1.97	-5.93	-0.97	3.32	3.35	2.82	7.21
RN49	-8.48	37.11			-3.66	3.81	11.25	8.44	-32.92	-15.61	7.84	-5.59	4.25	-11.49	-4.29	-5.60	14.93
RN50			-32.53	17.97	3.75	6.23	24.51	-26.02	-6.22	-8.91	2.48	5.42	10.62	2.63	-9.82	-10.08	19.98
RN51	11.15	4.64	22.71	-24.44	-11.66	3.28		26.93	-0.11	-14.41	-22.15	-19.16	30.13	-15.80	-3.27	7.95	4.19
RN52				17.24	11.28	-25.26	-1.57	-50.78	-16.28	-5.55	12.98	14.60	28.73	-2.08	-6.12	-9.29	32.09
RN53									-7.77	-17.17	-12.57	-13.89	-6.40	1.52	-1.74	29.50	28.52
RN54						-15.39			5.60	3.10	17.02	-4.56	0.24	8.16	-16.08	4.12	-2.21
RN58								1.69	-1.65	-9.47	-1.52	4.50	4.24	0.74	10.95	-3.60	-5.89
RN59					3.79	6.81	18.71	11.72	14.67	19.19	-3.53		-17.04	-11.04	-19.03	-21.31	-2.94
RN60						-12.78	8.49	12.09	-2.72	11.15	0.17	-21.28	-1.87	-4.29	-17.49	15.38	13.16
RN61							14.93	28.14	5.45	-6.39	0.90	5.05	-12.33	-20.73	-35.54	13.28	7.23
RN63	-5.73	-4.05	-5.49		2.25	-1.35	-4.05	1.26	-0.89	-6.44	-4.99	5.43	-4.53	-9.46	-2.30	18.01	22.35
RN64					-12.90	3.32	4.34	-1.61	-0.96	1.92	-4.24	-7.70	9.28	-1.05	-6.91	2.20	14.32
RN66		1.00	7.22	19.30	6.85	4.63	-2.23	-23.69	-19.13	-14.02	3.38	6.30	54.08	-8.71	-3.41	-16.55	-15.02
RN67					-9.52	2.52	0.48	19.62	11.34	-2.33	-0.19	-11.97	0.39	2.93	-11.18	-10.12	8.01
RN68		-2.11	24.99	7.06	-5.80	-15.41	-4.07	-3.94	3.23	-0.67	-4.96	-7.66	-0.45	1.63	2.71	5.06	0.40
RN70		-1.55	-9.64		3.36	10.04	9.25	-12.17	-13.52	8.32	-2.26	-0.26	11.44	7.27	3.49	-3.14	-10.63
RN71					-2.60	17.45	5.49	-9.00	7.50	-4.53	16.41	10.23	-6.23	-8.05	27.63	-32.18	-22.11
RN72		-12.06	3.03	10.70	13.00	7.38	8.50	2.86	5.05	-2.01	-9.04	-7.30	-4.81	-0.10	-10.12	-1.25	-3.84
RN73				14.86	5.82	-12.45	-7.33	-6.15	-16.56	-12.60	3.27	-1.39	2.95	-2.92	24.13	-6.19	14.56
RN74	-12.28	-2.74	-7.61	-3.65	2.42	1.35	5.46	-6.27	0.41	14.63	3.11	0.36	6.94	-5.30	5.62	3.27	-5.71
RN75	-17.77	-11.44	-5.08	9.67	17.59	10.26	-0.18	-16.22	-9.18	10.37	4.45	10.05	1.92	-2.97	2.21	-5.96	2.30
RN76					35.07	-4.36	15.07	-5.11	-5.80	-17.04	-1.51	-0.10	-5.18	-21.26	-7.87	6.26	11.83
RN77						-10.44	-1.33	2.80	4.55	2.09	4.86	-3.56	7.39	2.76	0.12	0.69	-9.94
RN78							-22.13	-0.76	-14.31	-16.27	1.31	0.55	5.13	6.07	19.31	5.23	15.86
RN79			-7.14	-4.87	-10.41	-2.87	-6.99	3.16	-3.79	-4.03	2.57	-7.27	1.33	-0.88	20.99	-0.18	20.36
RN80						-8.89	-3.74	7.28	-10.70	-1.93	4.45	-7.01	10.96	0.67	0.36	-5.58	14.13

***Table S3*. N. of days above average during which ^{7}Be activity concentrations are above 1.001 absolute value (1.001 based on overall averaged normalised data timeseries). This period indicates the extended "warm period" for each year between 2003 and 2019. High levels of concentration are proxy for high Tropopause which is a proxy for warmer tropospheric temperatures. Dataset is based on 120 days averaged normalized ^{7}Be concentration corrected from cosmic rays.**

N. of days are rounded to zero decimals.

Treaty number	2003	2004	2005	2006	2007	2008	2009	2010	2011	2012	2013	2014	2015	2016	2017	2018	2019
RN01		-33.88	8.12	34.12	-19.88	-25.88	25.12	1.12	11.12	-13.88	28.12	-9.88	8.12	33.12	74.12	15.12	36.12
RN03		-51.41	14.59	98.59	45.59			30.59	49.59	34.59	-0.41	-23.41	38.59	75.59	20.59	38.59	40.59
RN04	-162.65	-14.65	-114.65	-31.65	34.35	-12.65	36.35	45.35	21.35	-3.65	11.35	14.35	42.35	37.35	65.35	13.35	18.35
RN05											15.76	64.76	76.76	82.76	82.76	150.76	148.76
RN06	-50.06	-7.06	27.94	-6.06	-6.06	-83.06	-15.06	-97.06	-90.06	-61.06	-14.06	9.94	44.94	192.94	105.94	121.94	-74.06
RN07										25.06	-28.94	110.06	195.06	61.06	57.06	82.06	155.06
RN08	-30.47	218.53	214.53	44.53	54.53	-104.47	28.53	-17.47	-22.47	-135.47	-140.47	5.53	0.53	-88.47	-98.47	34.53	36.53
RN09	-54.35	11.65	36.65	-1.35	17.65	109.65	-55.35	30.65	-52.35	7.65	-6.35	-0.35	27.65	-6.35	19.65	39.65	-124.35
RN10			-84.59	-78.59	14.41	33.41	37.41	69.41	112.41	37.41	14.41	1.41	40.41	8.41	77.41	14.41	17.41
RN11	-51.18	-47.18	-110.18	165.82	47.82	158.82	-35.18	117.82	2.82	-47.18	6.82	-38.18	117.82	-4.18	20.82	-145.18	
RN13						99.65	1.65	150.65	139.65	-8.35	53.65	-1.35		-35.35	121.65	26.65	23.65
RN14							-91.59	83.41		128.41	5.41	125.41	223.41	32.41	9.41	103.41	91.41
RN15			25.24	-124.76	-120.76	34.24	-14.76	-45.76	99.24	31.24	23.24	-27.76	82.24	118.24	71.24	92.24	12.24
RN16	-125.65	-36.65	-37.65	30.35	8.35	2.35	12.35	37.35	8.35	31.35	21.35	-8.65	1.35	41.35	30.35	10.35	-26.65
RN17				-92.24	76.76	-17.24	62.76	-131.24	-42.24	133.76	85.76	85.76	10.76	71.76	30.76	162.76	-35.24
RN18	-129.94	-104.94	70.06		-44.94				141.06	-72.94	-24.94	-24.94	108.06	195.06	95.06	228.06	113.06
RN19				5.12	60.12				-18.88	235.12	79.12	-57.88	108.12	118.12	92.12	-46.88	1.12
RN23	-63.76	-48.76	51.24	15.24	11.24	-24.76	-0.76	-26.76	-33.76	5.24	6.24	59.24	34.24	132.24	41.24	-23.76	-133.76
RN26			33.88	110.88	5.88	-8.12	47.88	74.88	-33.12	5.88	88.88	78.88	65.88	52.88	0.88	-79.12	-148.12
RN27	-103.24	-57.24	-78.24	242.76	244.76	244.76	-109.24			68.76	6.76	-70.24	-6.24	-11.24	17.76	-31.24	-118.24
RN28			-7.41	47.59	168.59	140.59	-8.41		-61.41	-42.41	-118.41	10.59	42.59	92.59	6.59	116.59	-23.41
RN29			-126.59	-81.59	12.41	-91.59	-112.59	-88.59	-124.59		75.41	223.41	45.41	212.41	191.41	157.41	114.41
RN30				-55.00	-11.00	117.00	61.00		107.00	65.00			126.00	73.00	-10.00	120.00	25.00
RN31				-26.76		226.24	102.24	-3.76	-76.76	-85.76	24.24	52.24	174.24	27.24		94.24	96.24
RN33					32.12	164.12	81.12	62.12	4.12	39.12	-83.88	25.12	-4.88	47.12	62.12	77.12	18.12
RN34		3.12				-22.88		163.12	121.12	130.12	29.12	-95.88	-56.88	33.12	140.12	-8.88	59.12
RN37					20.53	31.53	-1.47	26.53	51.53	-29.47	74.53	98.53	7.53	-28.47	100.53	111.53	70.53
RN38		-106.29	91.71	-103.29	80.71	-65.29	97.71		-32.29	-109.29	71.71	54.71	-1.29	35.71	111.71	13.71	102.71
RN39					-1.24	35.76	84.76	113.76	63.76	57.76	-37.24	25.76			53.76	124.76	-34.24
RN40		37.35	51.35		-92.65				1.35	113.35			-0.65	91.35	230.35	173.35	71.35
RN42							-55.65	-52.65	19.35	47.35	114.35	44.35	220.35	174.35		107.35	92.35
RN43				-117.18	183.82	114.82	89.82	125.82	191.82	131.82				-123.18	-25.18	129.82	108.82
RN44									-37.00	112.00	-61.00	84.00	87.00	82.00	50.00	172.00	79.00

RN45		-63.88				36.12	60.12	10.12	73.12	-21.88		189.12	69.12	117.12	89.12	139.12	92.12
RN46		-1.29	-117.29	-52.29	59.71	74.71	24.71	-23.29	-53.29	36.71	21.71	31.71	24.71	54.71	5.71	17.71	40.71
RN47		-36.76	13.24	74.24	87.24	30.24	-2.76	-24.76	-26.76	-5.76	2.24	-30.76	-4.76	59.24	-0.76	32.24	-5.76
RN49	-81.24	-97.24			35.76	64.76	88.76	38.76		3.76	60.76	5.76	101.76	4.76	24.76	27.76	75.76
RN50				76.71	37.71	53.71	30.71	-62.29	33.71	-13.29	40.71	49.71	75.71	28.71	5.71	-5.29	41.71
RN51	-9.00	43.00	-37.00	-47.00	15.00	-111.00		3.00	63.00	-7.00	-97.00	-50.00	225.00	-86.00	8.00	173.00	39.00
RN52				98.65	72.65	-123.35	-3.35		-1.35	-5.35	77.65	79.65	149.65	72.65	20.65	6.65	68.65
RN53									40.12		25.12	-1.88	94.12	208.12	120.12	33.12	173.12
RN54						-64.82			55.18	103.18	2.18	90.18	90.18	112.18	48.18	102.18	34.18
RN58								87.29	60.29	-22.71	35.29	109.29	175.29	60.29	178.29	78.29	-35.71
RN59					-61.82	103.18	93.18	115.18	116.18	112.18	17.18			31.18	-4.82	3.18	20.18
RN60							93.35	92.35	80.35	134.35	79.35	-79.65	30.35	49.35	-88.65	101.35	117.35
RN61							104.82	121.82	75.82	58.82	72.82	102.82	5.82	0.82		129.82	20.82
RN63	-99.65	22.35	-21.65		16.35	-4.65	10.35	8.35	42.35	6.35	22.35	77.35	1.35	-20.65	19.35	73.35	-6.65
RN64						98.65	144.65	69.65	26.65	31.65	-17.35	-2.35	190.65	28.65	44.65	-50.35	-38.35
RN66		-101.00	28.00	-10.00	82.00	109.00	79.00				131.00	112.00	218.00	-32.00	8.00		
RN67					-81.76	25.24	-45.76	241.24	182.24	32.24	56.24	-32.76	43.24	117.24	-39.76	-63.76	61.24
RN68		-127.24	144.76	59.76	13.76	-56.24	6.76	-12.24	44.76	-4.24	8.76	-9.24	8.76	6.76	37.76	28.76	-12.24
RN70	-155.82	-31.82	-78.82	-115.82	79.18	172.18	39.18	-131.82	-130.82	177.18	-12.82	26.18	130.18	83.18	113.18	-45.82	-116.82
RN71					52.88	136.88	65.88	62.88	62.88	65.88	125.88	166.88	-63.12	-57.12	76.88		
RN72			62.18	99.18	112.18	97.18	47.18	48.18	66.18	24.18	-33.82	-78.82	-55.82	5.18	-40.82	21.18	-65.82
RN73				54.29	145.29	-63.71	-28.71	13.29		-44.71	44.29	52.29	84.29	-25.71	233.29	-7.71	70.29
RN74		31.88	-124.12	-14.12	-15.12	42.88	72.88	-35.12	27.88	193.88	12.88	-18.12	78.88	-86.12	85.88	3.88	-92.12
RN75		-82.35	-71.35	94.65	139.65	81.65	-0.35	-144.35	-98.35	84.65	25.65	94.65	43.65	-14.35	40.65	-38.35	1.65
RN76					110.29	27.29	102.29	21.29	49.29	-1.71	46.29	31.29	17.29	-93.71	39.29	107.29	14.29
RN77						-68.71	39.29	92.29	94.29	80.29	106.29	10.29	79.29	59.29	81.29	87.29	-92.71
RN78								81.12		-80.88	80.12	70.12	118.12	103.12	221.12	116.12	114.12
RN79				-78.06	-100.06	35.94	-29.06	54.94	4.94	13.94	39.94	-79.06	73.94	42.94	245.94	11.94	118.94
RN80						24.59	27.59	48.59	7.59	51.59	78.59	56.59	61.59	89.59	56.59	54.59	54.59

***Table S4.* Count the maximum consecutive n. of days per year per station that are required to transit from the yearly MEAN value (based on normalized ^{7}Be value) up to the yearly MAXIMA which is a variable threshold from year to year. The Maxima indicates when the convergence zone is closest to the station. The transit time between Mean and the Maxima indicate a time period of cell progression which is a proxy for Hadley cell circulation strengthening or weakening. The higher the number of days required to reach the maxima, the slower the cell progression, the weaker the circulation. N. of days are rounded to zero decimals. Dataset is based on 120 days averaged normalized ^{7}Be concentration corrected from cosmic rays.**

Treaty number	2003	2004	2005	2006	2007	2008	2009	2010	2011	2012	2013	2014	2015	2016	2017	2018	2019

RN01	54	177	182	208	170	159	188	184	186	177	183	170	169	187	184	159	138
RN03		140	168	189	134		2	167	194	165	168	179	172	177	167	169	131
RN04	44	175	199	199	191	161	199	169	196	184	164	185	215	177	187	159	151
RN05											82	158	148	173	159	143	134
RN06	64	125	172	149	166	145	153	129	129	146	176	170	190	148	185	184	90
RN07										155	167	147	179	186	161	169	134
RN08	61	176	120	133	178	75	190	136	168	183	172	207	158	162	204	159	128
RN09	67	189	172	151	176	193	186	194	190	185	164	167	173	148	192	165	115
RN10	51	202	165	169	171	189	155	153	238	181	167	155	170	162	198	171	127
RN11	61	125	219	173	201	196	172	224	181	178	170	165	209	164	203	160	138
RN13						170	194	224	94	136	194	89		147	177	148	108
RN14							4	179	146	207	162	163	226	183	208	196	120
RN15			175	17	99	155	195	168	135	103	150	198	223	168	174	218	117
RN16	54	185	161	186	158	164	183	164	165	181	182	156	159	191	171	160	125
RN17				68	187	203	156	198	195	233	200	212	143	158	164	204	114
RN18	52	162	163		112	184	167	164	182	63	135	193	182	167	168	165	133
RN19	38		82	167	160				28	193	136	107	188	159	196	198	114
RN23	57	174	209	179	173	178	164	97	66	167	140	190	180	169	196	189	125
RN26			148	224	187	163	172	177	160	190	165	206	214	155	153	180	130
RN27	69	169	121	134	160	108	4			99	169	203	186	138	176	180	96
RN28			163	158	150	247	217		119	127	18	67	160	241	157	187	114
RN29		45	177	108	149	145	214	71	120	210	187	140	143	156	175	190	102
RN30				24	181	203	166	75	200	171	125		184	170	162	174	123
RN31			147	95	31	159	175	177	150	175	167	152	175	164	144	159	122
RN33					156	190	192	159	144	205	144	232	210	184	180	188	139
RN34	47	63			111	58		171	143	153	145	137	203	153	207	146	129
RN37					195	193	197	218	189	206	186	218	156	49	189	204	153
RN38	5	13	120	46	191	18	162	192	163	186	128	171	175	195	186	165	110
RN39					43	169	137	203	155	146	25	121	1		123	165	110
RN40		91	182	75	95	187	234	16	156	170	58		67	179	190	160	99
RN42							93	202	195	164	229	139	166	156	194	226	136
RN43				24	189	193	149	246	201	159	150	192	167	163	195	193	98
RN44									67	181	37	204	63	180	179	196	139
RN45	60	204	182	194	188	193	218	170	223	180	227	229	151	178	152	137	121
RN46	39	153	24	139	196	193	177	182	236	170	150	176	178	149	159	176	141

RN47	44	178	167	216	191	175	154	206	168	167	154	170	166	211	155	185	145
RN49	47	11			179	170	196	139	99	175	160	163	206	137	154	159	162
RN50			31	167	166	176	119	97	181	164	170	174	190	158	160	161	121
RN51	39	149	87	143	176	5		92	178	153	114	76	126	146	132	170	147
RN52				125	193	192	133	29	161	165	179	182	141	204	167	173	146
RN53									139	203	168	130	205	196	159	44	120
RN54						28			125	166	59	180	171	180	194	173	117
RN58								170	188	142	158	191	207	150	152	205	134
RN59					27	178	154	143	183	176	131		17	164	150	197	121
RN60						8	141	143	200	142	182	179	153	181	180	137	119
RN61							169	186	165	179	170	193	161	167	193	209	112
RN63	69	194	135		146	144	185	151	191	182	200	153	186	187	181	191	130
RN64					112	181	196	208	167	130	201	149	159	177	203	54	66
RN66		16	109	55	136	198	215	35	23	192	228	99	151	120	181	167	139
RN67					81	146	79	152	188	170	194	196	186	207	100	142	112
RN68		46	156	145	180	145	166	154	174	136	164	186	153	144	168	161	127
RN70		133	123		202	116	135	205	138	203	172	183	180	186	149	139	71
RN71					177	148	180	201	176	209	196	175	212	194	178	212	149
RN72		32	213	144	144	193	188	168	213	194	191	215	223	162	213	194	128
RN73				83	195	107	160	201	177	168	170	191	169	139	176	186	125
RN74	62	212	207	181	143	200	204	234	196	161	170	154	207	202	159	157	110
RN75	62	148	203	191	201	185	158	248	170	170	157	189	193	189	186	148	157
RN76					145	156	149	163	177	166	165	148	161	192	170	214	112
RN77						162	162	190	163	175	207	167	155	167	210	201	142
RN78							85	191	176	175	182	175	185	191	165	145	134
RN79			49	85	182	187	185	157	188	161	144	208	191	184	155	156	132
RN80						196	160	157	203	180	196	201	140	211	180	188	146

Table S5. Tropopause profile figure 4 is based on the below data table featuring ^{7}Be ***% of growth over the overall average and re-analysis data (aggregation of different grid resolution as presented in figure 1 of Schmidt et al. 2008). The correlation with*** ^{7}***Be growth in 2017 is 0.81. Positive trend is in agreement with the re-analysis data. In 2019 the trend varies at the equator but we currently do not have re-analysis data of 2019 available. 2005, 2017, and 2019 are selected to show the tropopause growth trend.***

IMS Station	^{7}Be % of Growth versus average along the Canadian-Antarctica profile				Aggregation of re-analysis measurement (Schmidt et al. 2008)	
	Latitude	% (2005)	% (2017)	% (2019)	Latitude	mt/Yr (2001-2007)

RN73	-64.5		24.13	14.56	-75	40
RN18	-53.1	11.95	14.12	11.26	-68	18
RN03	-41.1	-6.14	-3.86	20.62	-55	-10
RN01	-34	-0.32	9.20	18.91	-35	45
RN11	-22.5	-6.59	-1.91	-7.54	-20	-5
RN50	8.9	-32.53	-9.82	19.98	9	-18
RN44	28		-5.70	5.45	19	2
RN70	38.7	-9.64	3.49	-10.63	30	37
RN14	49.3		-7.37	13.25	60	3
RN15	74.7	-6.77	8.97	8.56	85	28
Correlation between ^{7}Be % of growth and radio-sonde based data (2001-2007)		0.5	**0.8**	-0.1		

Supplementary Figures

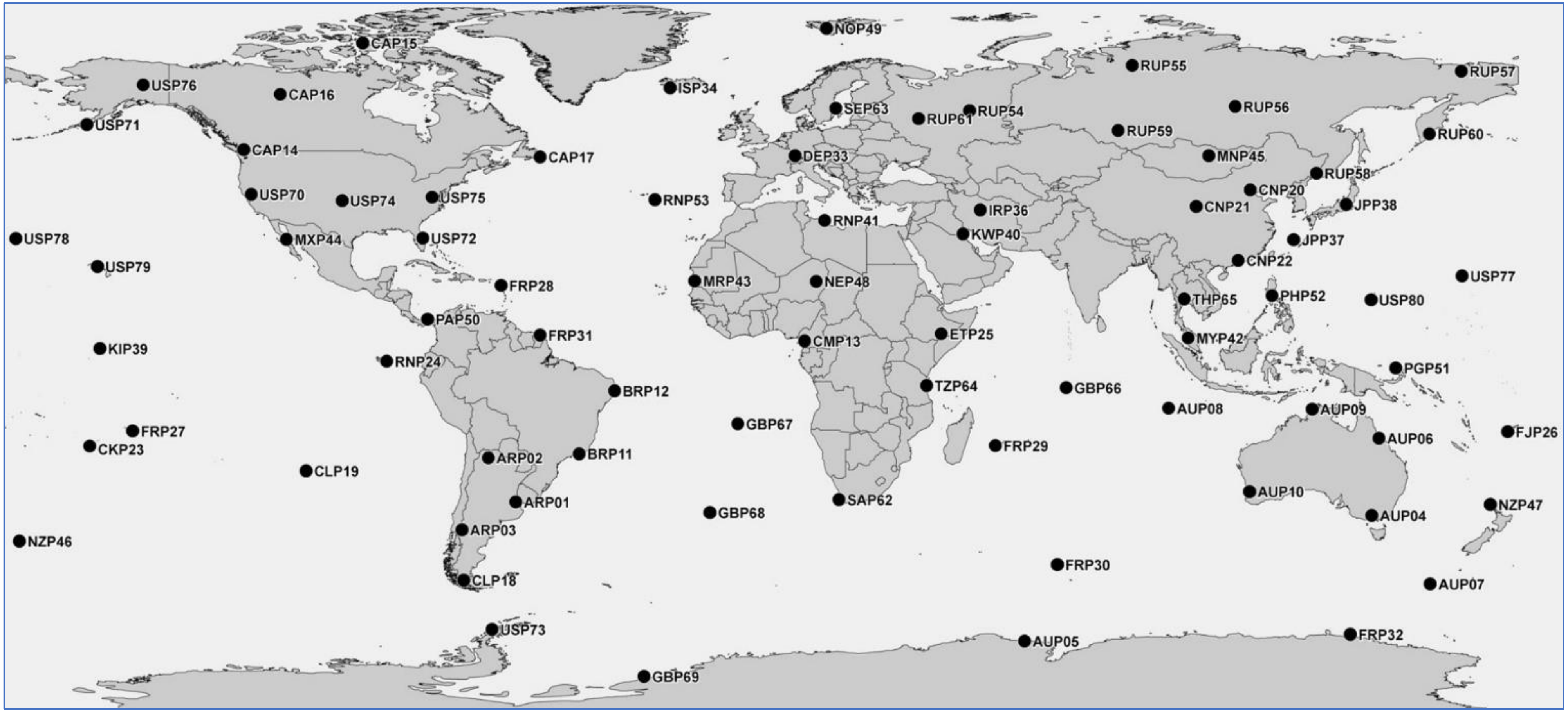

Figure S1. **The radionuclide component of the International Monitoring System consists of 80 stations (70 stations currently certified for operation) and aims at the detection of signature isotopes from nuclear explosions**. Software used to produce figure S1: QGIS Development Team (2019). QGIS Geographic Information System. Open Source Geospatial Foundation Project. http://qgis.osgeo.org.

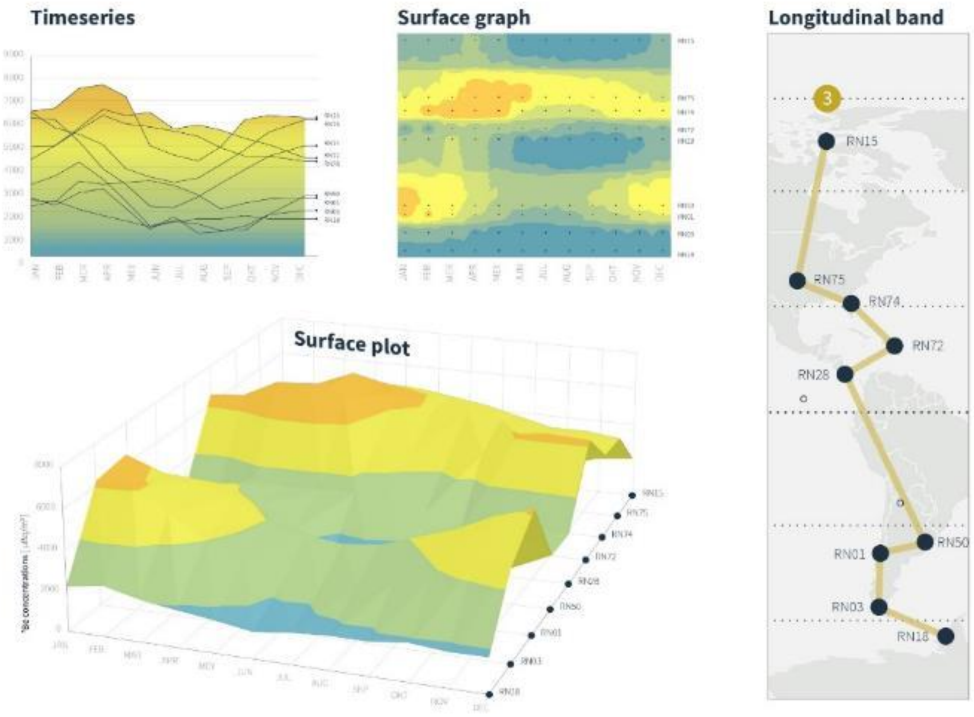

Figure S2. **Reconstruction of HFIZ or downward branch of the Hadley circulation through beryllium 7 is demonstrated in Terzi and Kalinowski (2017) [54].**

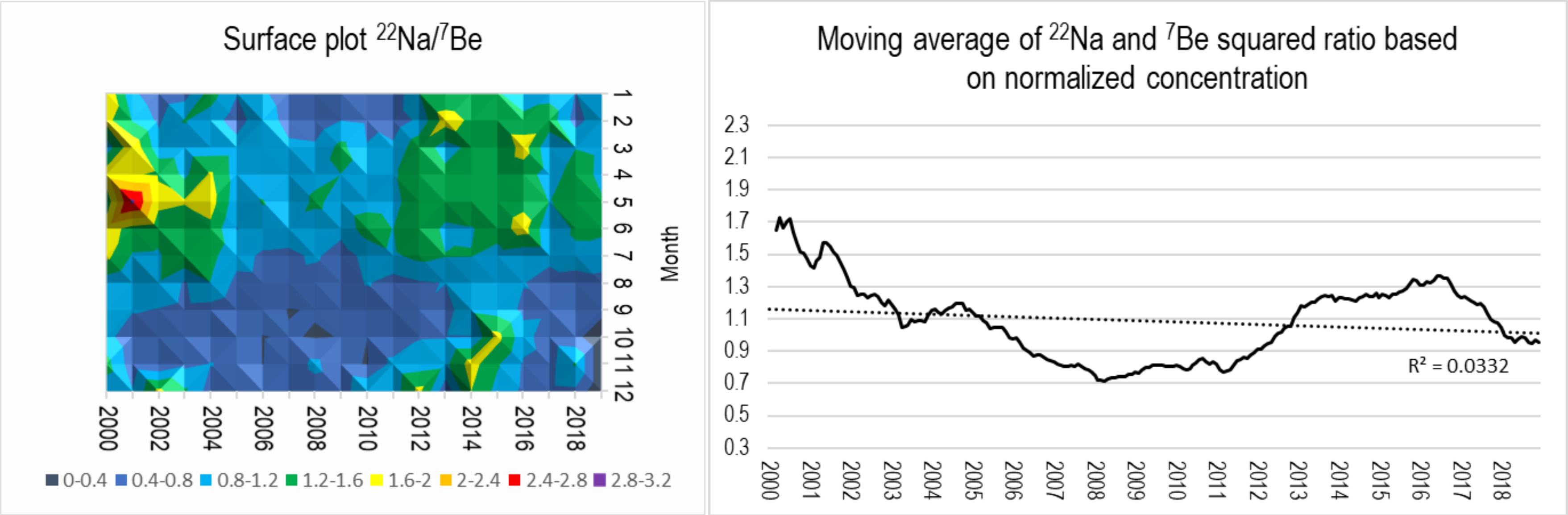

Figure S3. Change of atmospheric cells progression is also visible through isotopic ratio change over time. As seasons have characteristic isotopic ratio due to the position of the convergence zones over time, isotopic ratio of ^{22}Na and ^{7}Be can also indicate the circulation trend. In this case ratios are from Switzerland where the seasonal gradient of the isotopic ratio has decreased over time possibly indicating the slowing down of atmospheric cell. The surface charts above show the change of atmospheric cells progression visible through isotopic ratio change over time. The figure displays how much the radioisotope ratio seasonal gradient (^{22}Na versus ^{7}Be) varies over time, meaning that a seasonal peak is expression of fast transition of the atmospheric cell convergence zone over the point of measurement (IMS Station). In Switzerland, where the plotted data come from, that typically happen during pre-summer season, end of April beginning of June. A plane like feature of the seasonal profile indicate a slowing down of Hadley cell progression, meaning more time is needed to transit to a different ratio value. Between 2000 and 2018 a gradual transition from peak to plane is observed, possibly indicating a general weakening of the circulation over Switzerland. Right chart displays the same dataset where decreasing speed trend (linear trendline) is indicated by the dotted black line. Looking at the Na-22/Be-7 ratio provides a better indicator for the origin of air masses, since the ratio is very sensitive to the height of production with much lower values in the lower stratosphere than in the upper troposphere. Unfortunately, due to the much lower abundance of Na-22 (activity ratio of Be-7/Na-22 ratio is on the order of 10 to 4) no global Na-22 dataset is available comparable to the here discussed Be-7 data. Data have been provided by P. Steinmann from BAG Bern, Switzerland.

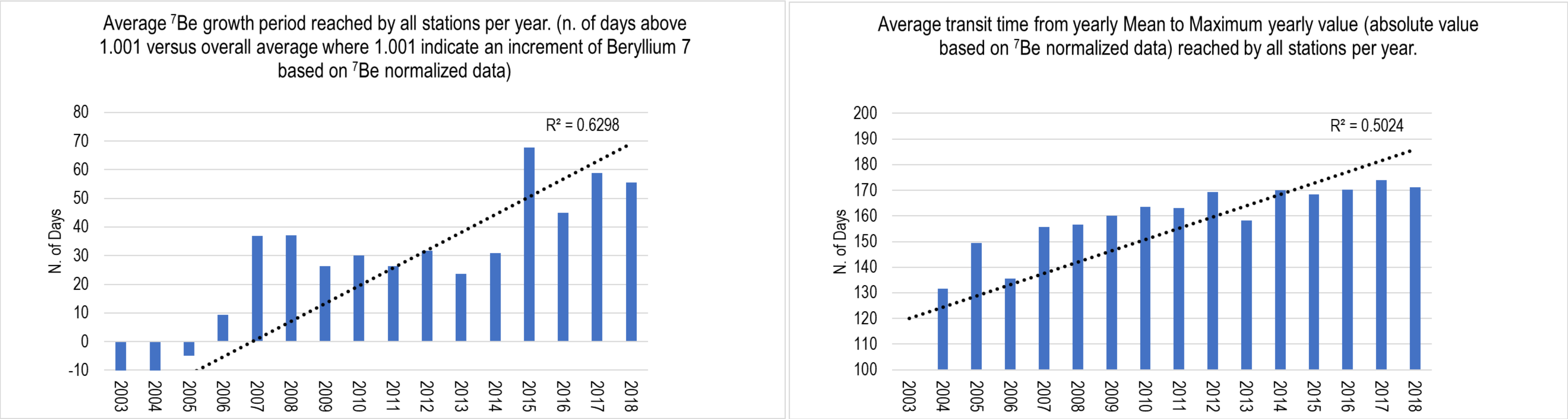

Figure S4. **The left chart shows the global (62 IMS stations considered, for details please see table S1) ^{7}Be growth versus average with a clear positive linear trend (black continuous line). The right chart shows, based on the same 62 stations, the increase in the n. of days required per year to reach the maximum threshold from a baseline of the yearly mean. The trend is positive which indicates a weakening of cell progression and therefore suggest a slowing down of the general circulation. Presented dataset has been corrected from cosmic rays.**

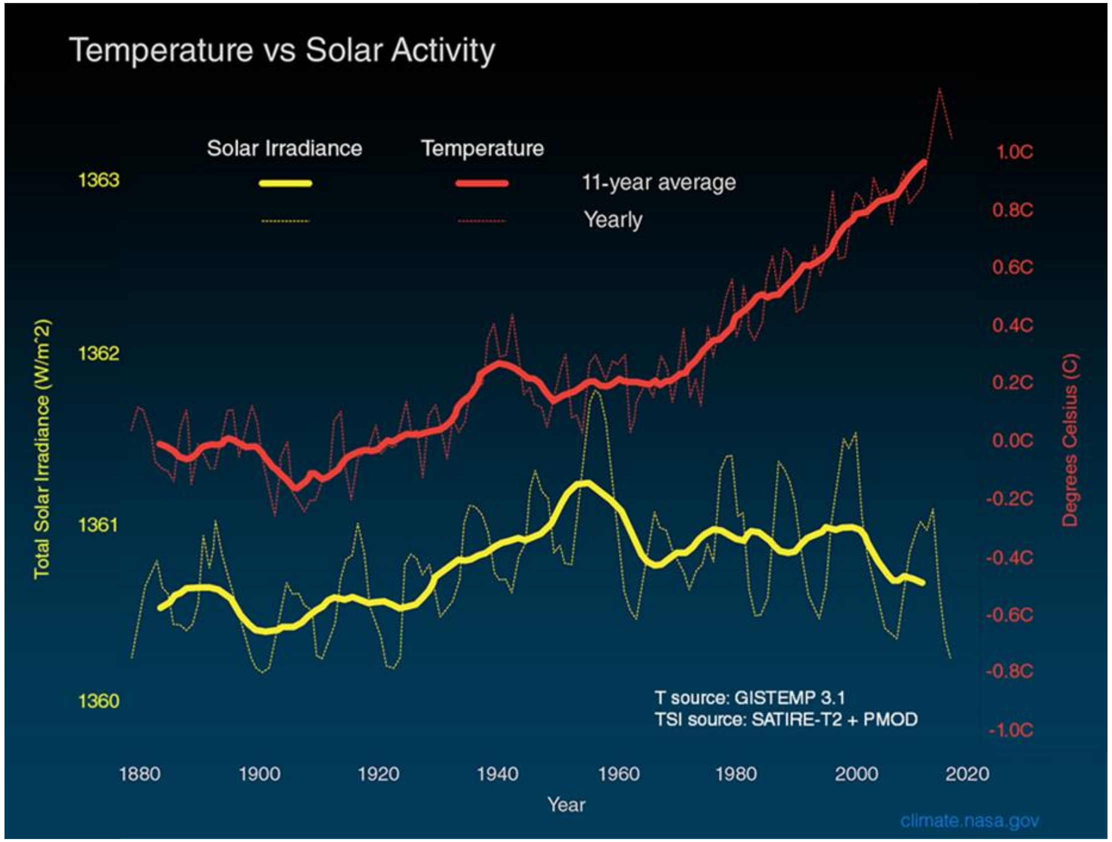

Figure S5. **Global surface temperature changes (red line) and Sun's energy that Earth receives (yellow line) in watts (units of energy) per square meter since 1880.The lighter/thinner lines show the yearly levels while the heavier/thicker lines show the 11-year average trends. Eleven-year averages are used to reduce the year-to-year natural noise in the data, making the underlying trends more obvious. The amount of solar energy that Earth receives has followed the Sun’s natural 11-year cycle of small ups and downs with no net increase since the 1950s. Over the same period, global temperature has risen markedly. It is therefore extremely unlikely that the Sun has caused the observed global temperature warming trend over the past half-century. Credit: NASA/JPL-Caltech [55].**

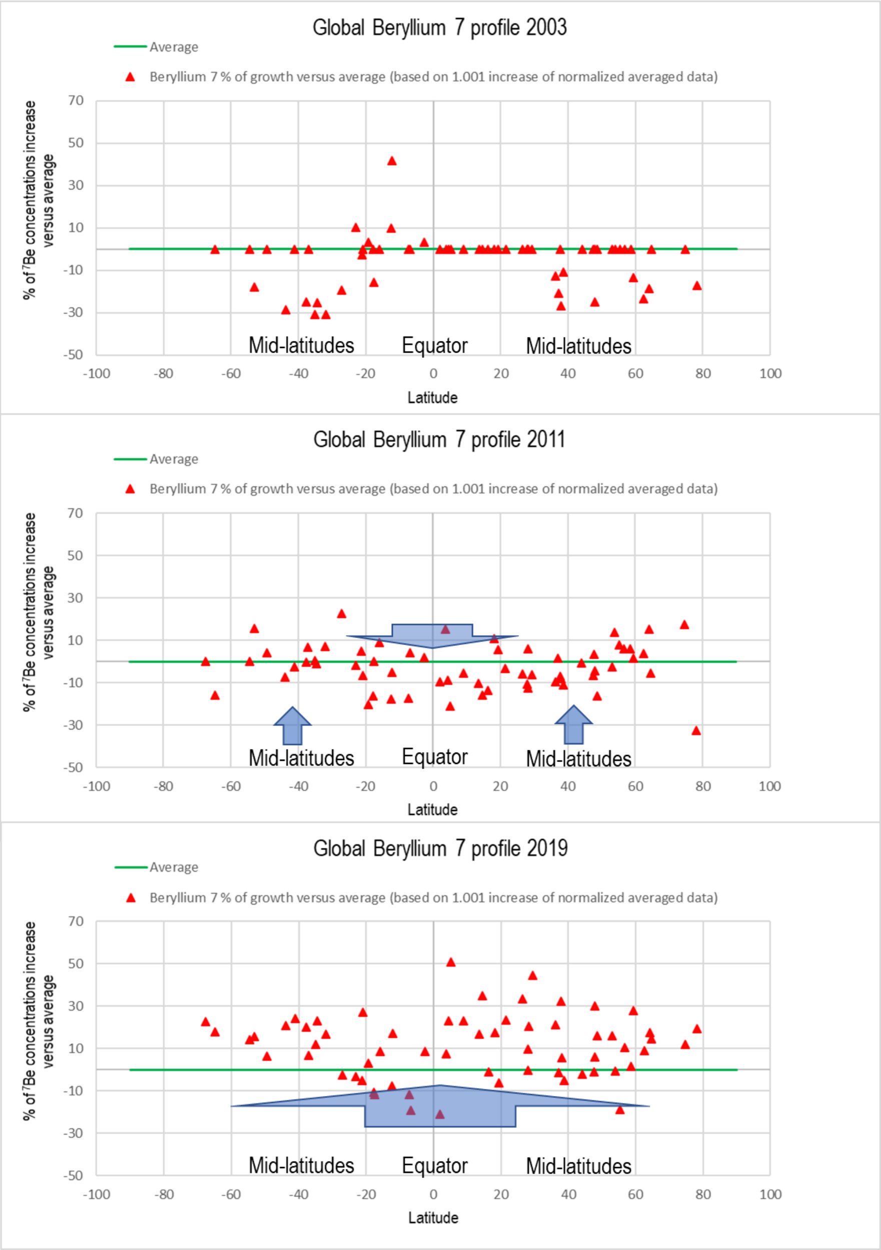

Figure S6. Supplementary information related to figure 4. Figure 5S display the 7Be growth trend at all 62 stations in 2003, 2011 and 2019. Displayed dataset has not been corrected from cosmic ray.

In 2003 the concentration at midlatitude is below average, in 2011 mid-latitudes concentrations rise above average while concentrations decrease at the equator. These results are consistent with re-analysis of radio-sonde measurement which show tropopause increase at the mid latitudes while a decrease along the ITCZ.

In 2019 the 7Be growth rate is increasing globally with the exception of a few locations.

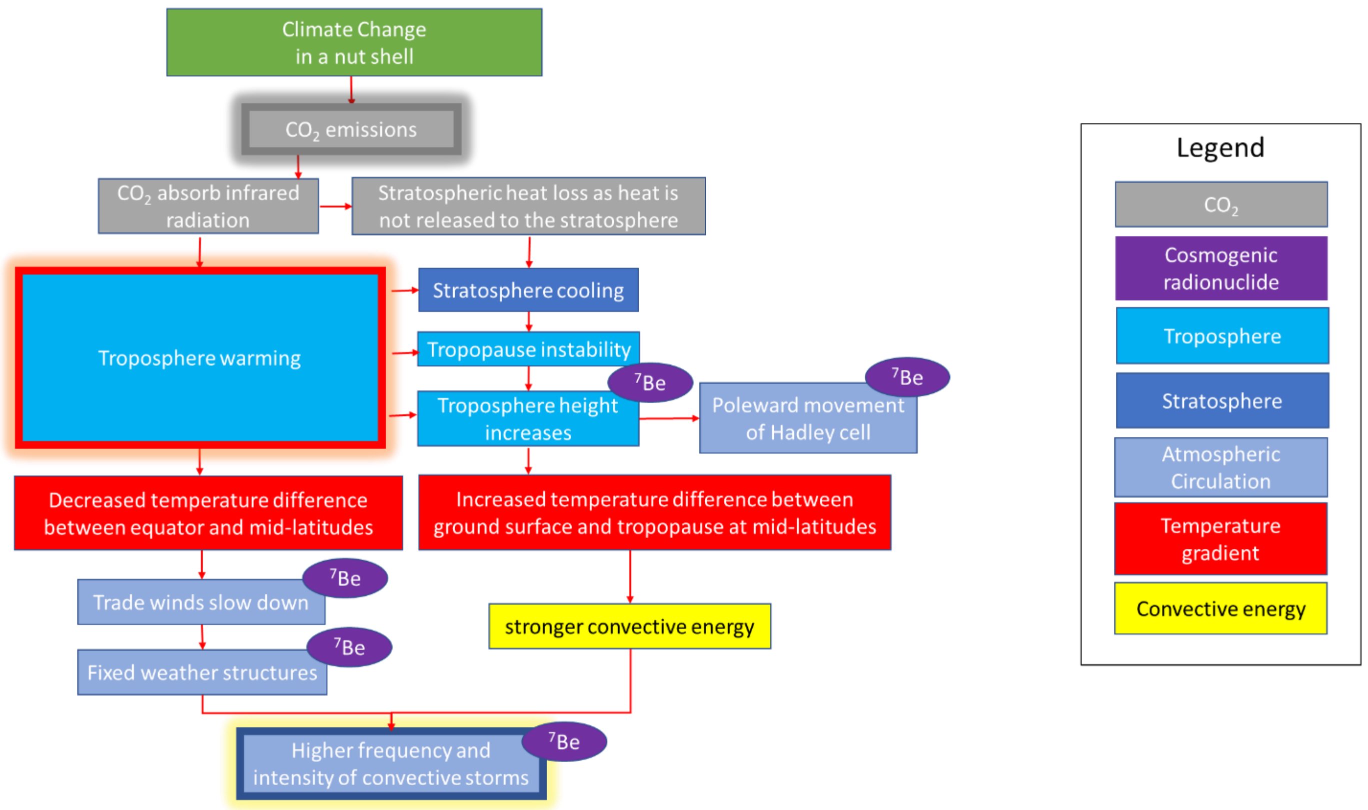

Figure S7. Flow chart showing the connection between CO_2 emissions to climate change patterns and the use of beryllium-7 as a proxy.

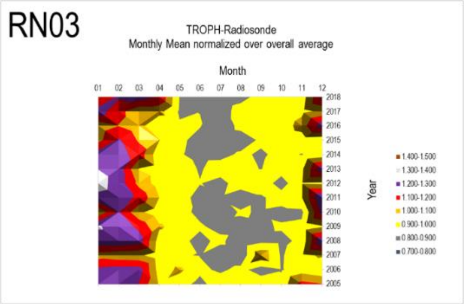

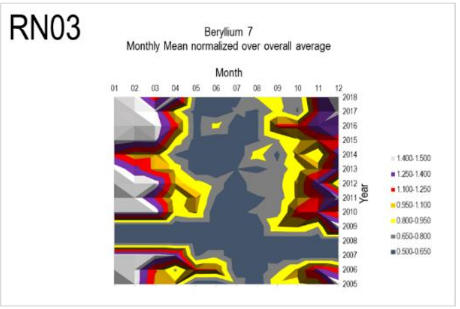

Year	2005	2006	2007	2008	2009	2010	2011	2012	2013	2014	2015	2016	2017	2018
Correlation	0.80	0.71	0.94	-	0.87	0.55	0.92	0.78	0.89	0.61	0.84	0.68	0.84	0.80

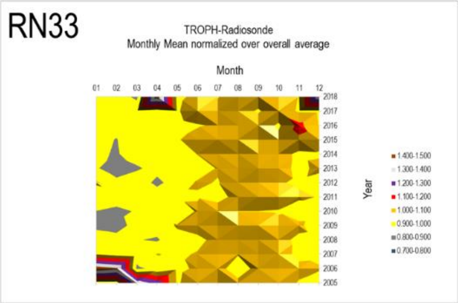

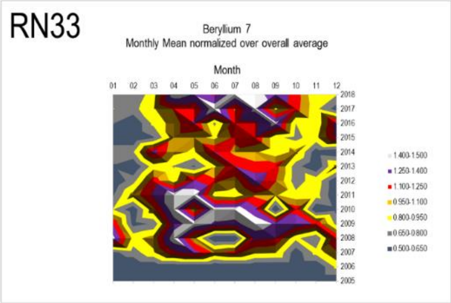

Year	2007	2008	2009	2010	2011	2012	2013	2014	2015	2016	2017	2018
Correlation	-0.11	-0.21	0.72	0.79	-0.09	0.17	0.64	0.14	0.78	0.39	0.70	0.23

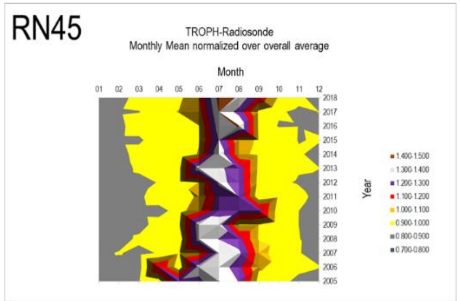

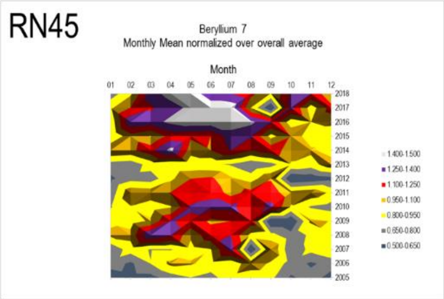

Year	2005	2006	2007	2008	2009	2010	2011	2012	2013	2014	2015	2016	2017	2018
Correlation	0.80	0.61	0.23	0.28	0.39	0.80	0.41	0.47	-0.24	0.44	0.72	0.57	0.47	0.19

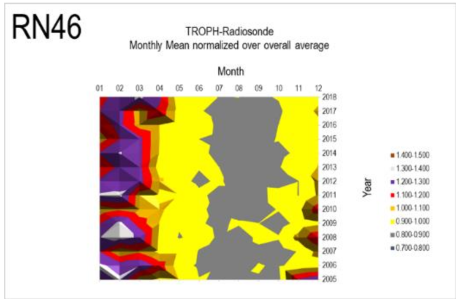

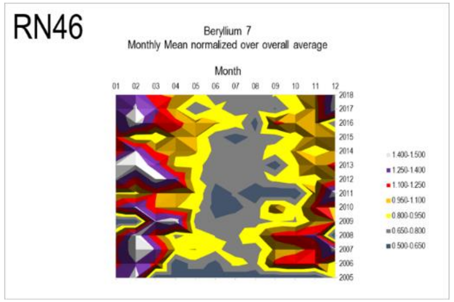

90

Year	2006	2007	2008	2009	2010	2011	2012	2013	2014	2015	2016	2017	2018
Correlation	0.39	0.67	0.57	0.82	0.63	0.13	0.89	0.88	0.88	0.23	0.76	0.71	0.62

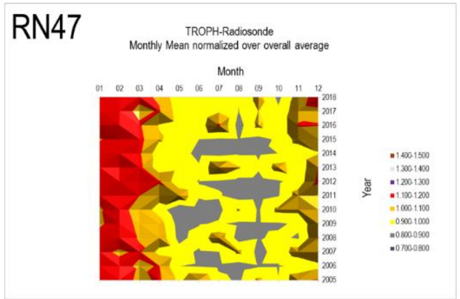

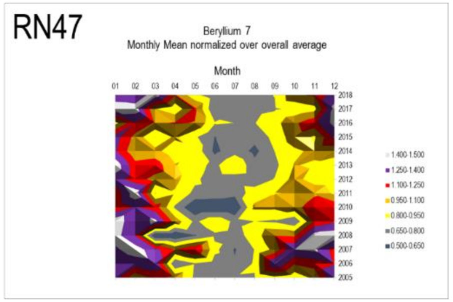

91

Year	2005	2006	2007	2008	2009	2010	2011	2012	2013	2014	2015	2016	2017	2018
Correlation	0.62	0.37	0.61	0.49	0.82	0.92	0.43	0.74	0.66	0.77	0.60	0.39	0.71	0.77

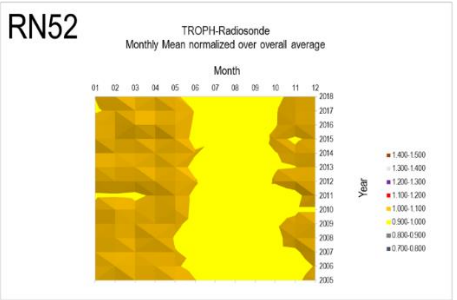

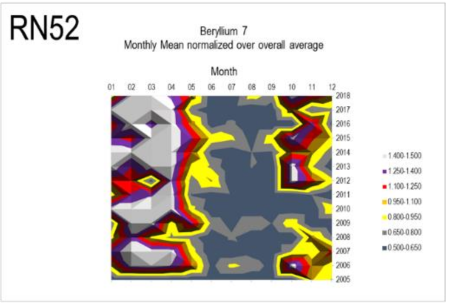

92

Year	2006	2007	2008	2009	2010	2011	2012	2013	2014	2015	2016	2017	2018
Correlation	0.44	0.69	0.72	0.70	0.77	0.27	0.57	0.66	0.47	0.51	0.72	0.38	0.61

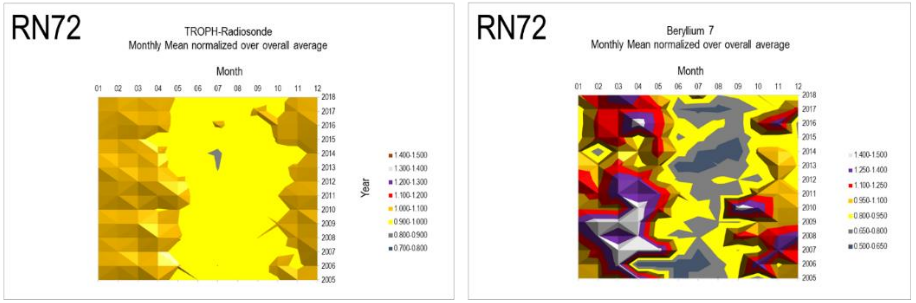

Year	2005	2006	2007	2008	2009	2010	2011	2012	2013	2014	2015	2016	2017	2018
Correlation	0.53	0.63	0.49	0.86	0.85	0.16	0.77	0.84	0.71	0.43	0.44	0.25	0.66	0.55

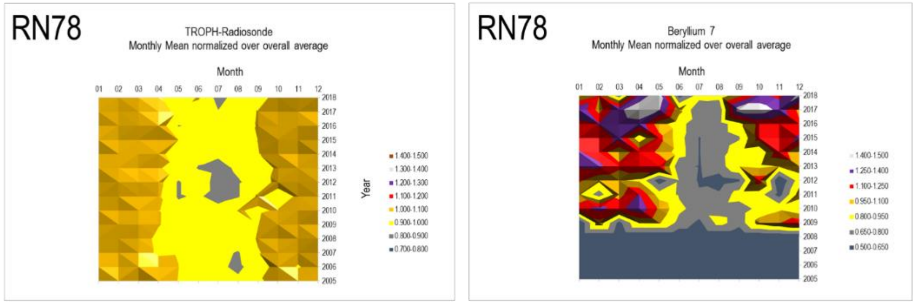

Year	2009	2010	2011	2012	2013	2014	2015	2016	2017	2018
Correlation	0.33	0.33	0.45	0.86	0.80	0.53	0.84	0.82	0.57	0.18

Figure S8 shows examples of IMS station locations from all regions of the worlds where we compared tropopause profile with be7 data. Tropopause profile is built with GPS-RO yearly average data (left plot) and be7 yearly averaged data (right plot). GPS-RO data GPS coordinates to IMS stations locations. GPS-RO are retrieved temperature profiles generated by satellites. All available years are plotted. The coloured scale is based on normalised data obtained by dividing absolute values with the overall timeseries average. The correlation between GPS-RO data and Be7 trend is highest at the mid latitudes, where we have correlation up to 0.8. About 1/3 of stations (about 20 stations) have a good correlation, 1/3 of station with low correlation and 1/3 with no correlation. We expected a higher n. of station with higher correlation rate but the low rate may be due to Be7 dataset which needs to be corrected for wash out effect. The correlation is lowest at equatorial and polar latitudes (maybe also related to wash out effect or the low transport) but also site specific due to local weather pattern therefore further research is needed. The comparative scale is built on the ratio of absolute monthly tropopause height (based on GPS-RO data) versus absolute monthly Be7 values. The average ratio is between 3 and 6, lower at the mid latitudes and highest (6) at the equator where the height of tropopause is the highest and the wash out effect is stronger. The uncertainty of the ratio value (standard deviation) is low at the mid latitudes (which is good) and increases at the equator and at the very high latitudes, those areas where be7 is most affected by wash out.

Supplementary Formulas

***Formula S1*. (1) Mean calculation and (2) normalized mean calculation used for data processing. Where x^i is the ^{7}Be activity concentration recorded each day [µBq/m³]. *N* is the number of days for which the mean is calculated: 0/-120 for the presented method. *M* is the calculated mean. $\overline{x}$ is the overall averaged activity concentration of ^{7}Be [µBq/m³] measured at each location: 2003-2019 for all listed IMS Station. *N(M)* is the calculated normalized mean.**

1 $$M = \frac{1}{n} * \sum_{i=1}^{n} x^i$$

2 $$N(M) = \frac{1}{\bar{x}} * \frac{1}{n} * \sum_{i=1}^{n} x^i$$
