# Supplementary material for: Radioisotopes demonstrate changes in global atmospheric circulation possibly caused by global warming
Source: Sci Rep. 2020 Jul 1;10:10695. doi: 10.1038/s41598-020-66541-5 (PMC7329870; doi:10.1038/s41598-020-66541-5)
Supplement: Supplementary file 3 — Supplementary Information: Beryllium-7 maps. [file 41598_2020_66541_MOESM3_ESM.pdf]

Title: Radioisotopes demonstrate changes in global atmospheric circulation possibly caused by global warming

Authors: Lucrezia Terzi[a,b*], Gerhard Wotawa[c], Michael Schoeppner[d,e], Martin Kalinowski[d], Paul R.J. Saey[b],Philipp Steinmann[f], Lan Luan[g] and Paul W. Staten[g].

Affiliations: [a]Belgian Nuclear Research Centre (SCK•CEN), Mol, Belgium; [b]Technische Universität Wien, Atominstitut, Austria; [c]Zentralanstalt für Meteorologie und Geodynamik (ZAMG), Vienna, Austria, [d]Provisional Technical Secretariat, Preparatory Commission for the Nuclear-Test-Ban Treaty Organization, International Data Centre, Vienna, Austria; [e]Institute of Safety/Security and Risk Sciences, Vienna, Austria; [f]Federal Office of Public Health (BAG), Bern, Switzerland; [g]Indiana University Bloomington, Bloomington, Indiana, USA.

***Corresponding Author:**
Lucrezia Terzi
Ph: +43 6644553833
e-mail: lucrezia.terzi@sckcen.be
[a] SCK•CEN, Belgium Nuclear Research Centre,
Boeretang 200, 2400 Mol, Belgium.
[b] Technische Universität Wien, Atominstitut,
Stadionallee 2, 1020 Wien, Austria.

^{7}Be normalized trend interpolated into a global map

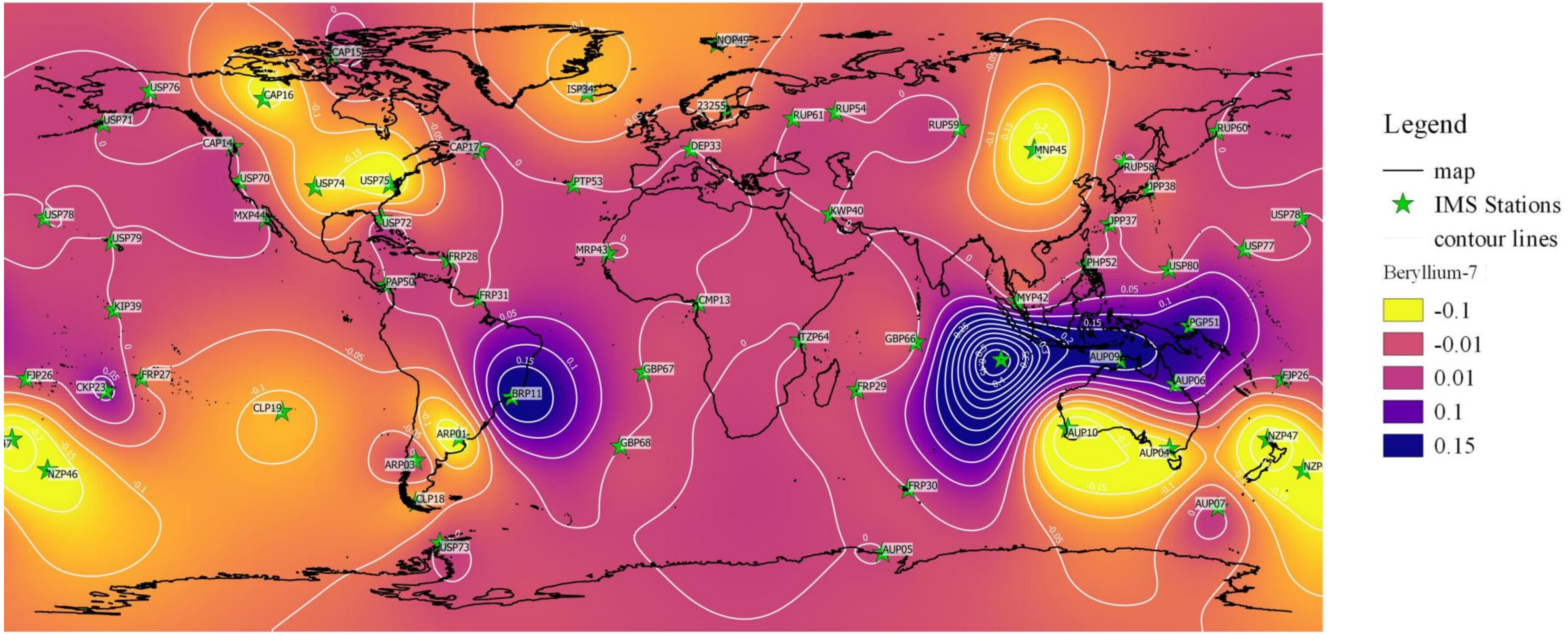

2003

Map executed with QGIS.
QGIS Development Team (2019). QGIS Geographic Information System. Open Source Geospatial Foundation Project. http://qgis.osgeo.org.

^{7}Be normalized trend interpolated into a global map

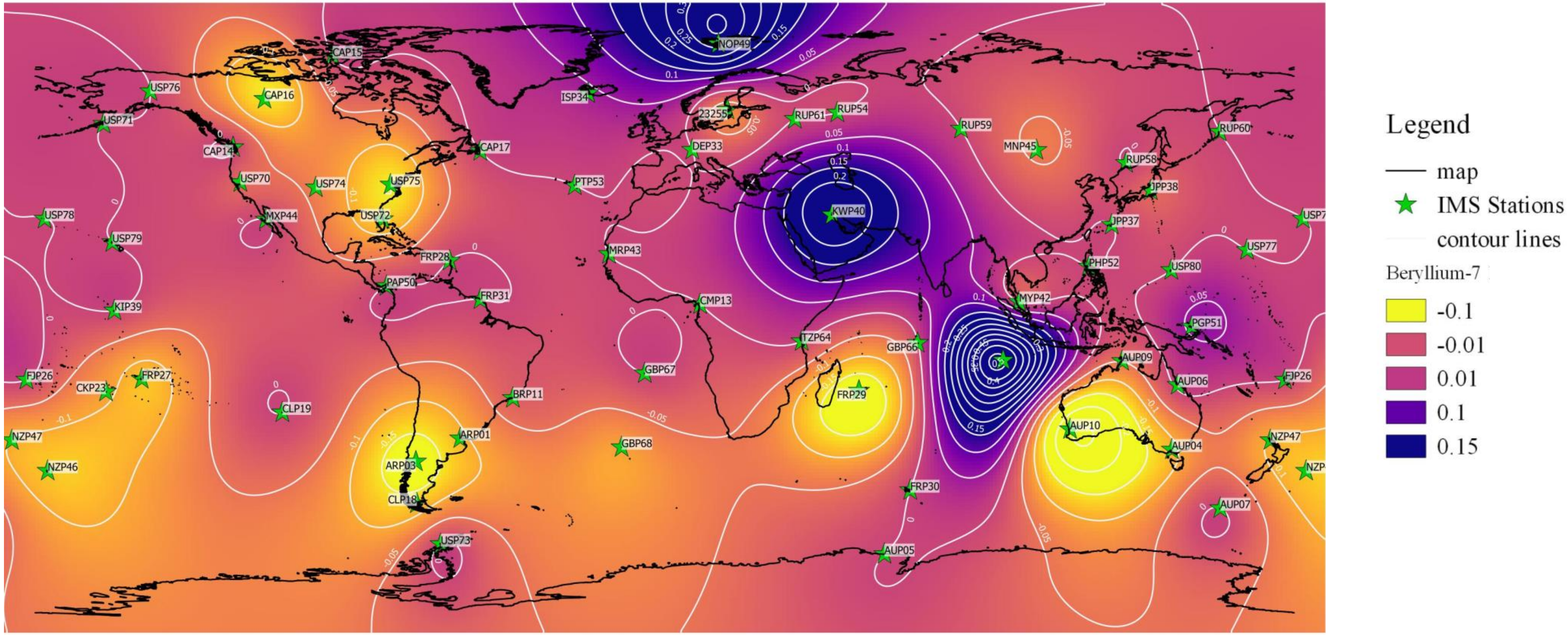

2004

Map executed with QGIS.
QGIS Development Team (2019). QGIS Geographic Information System. Open Source Geospatial Foundation Project. http://qgis.osgeo.org.

^{7}Be normalized trend interpolated into a global map

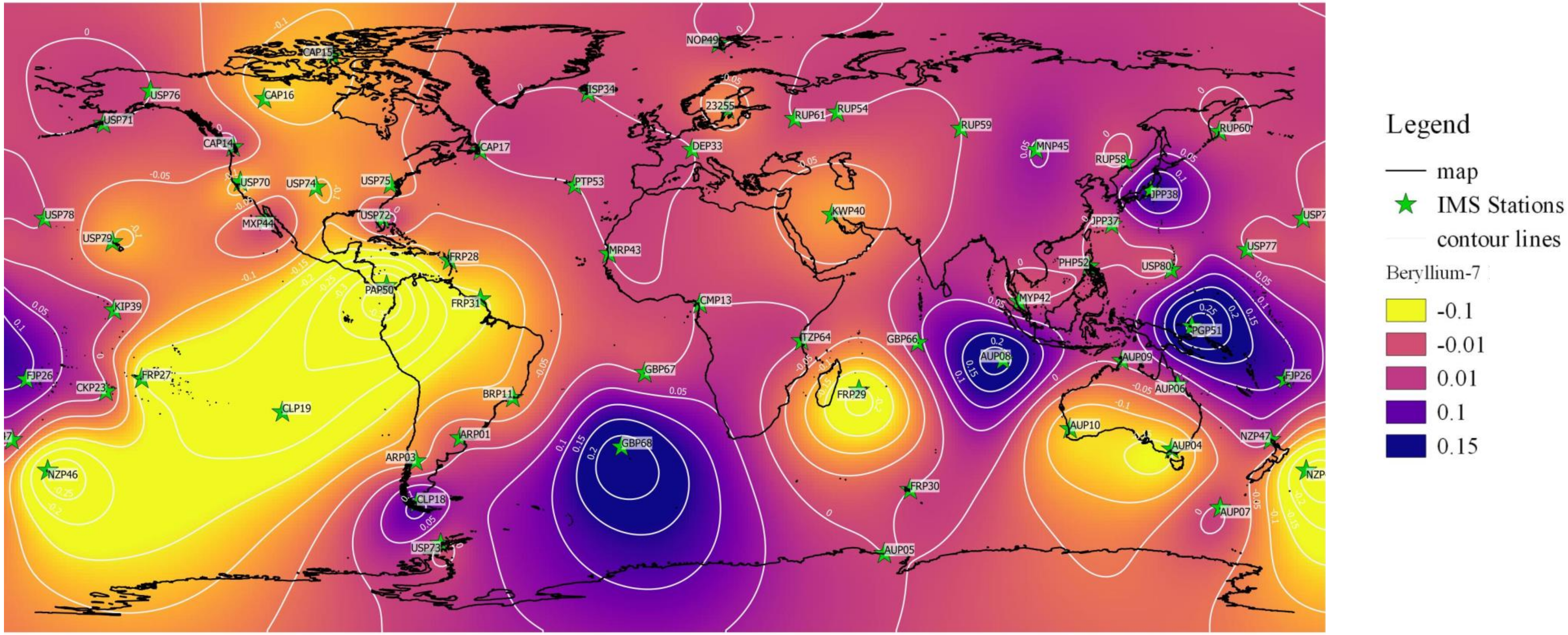

2005

Map executed with QGIS.
QGIS Development Team (2019). QGIS Geographic Information System. Open Source Geospatial Foundation Project. http://qgis.osgeo.org.

^{7}Be normalized trend interpolated into a global map

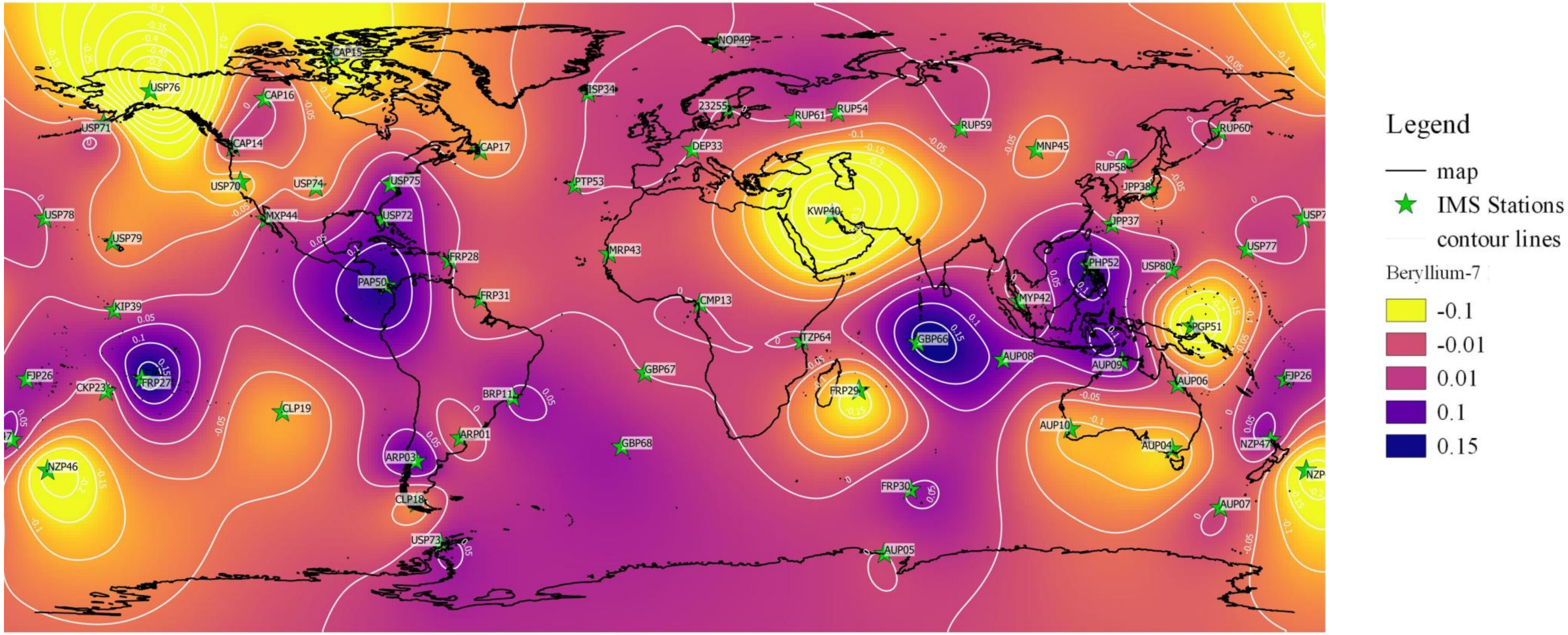

2006

Map executed with QGIS.
QGIS Development Team (2019). QGIS Geographic Information System. Open Source Geospatial Foundation Project. http://qgis.osgeo.org.

^{7}Be normalized trend interpolated into a global map

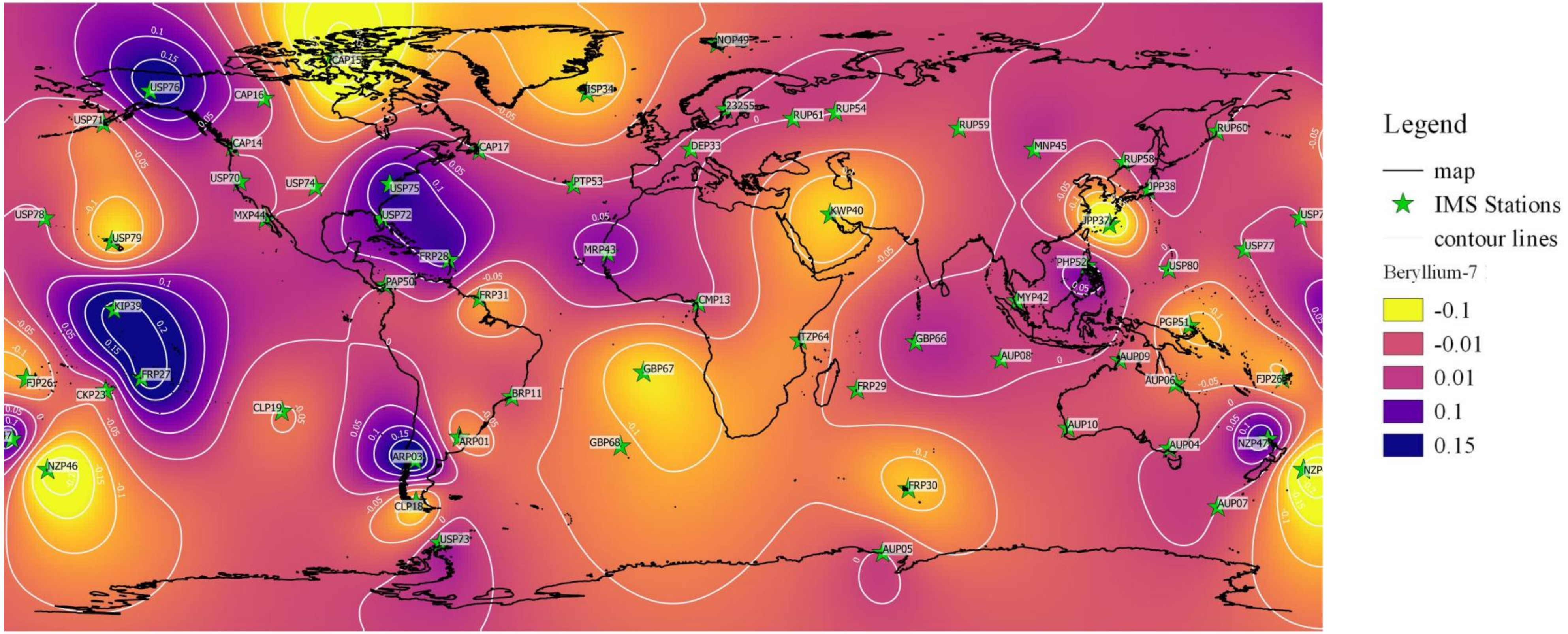

2007

Map executed with QGIS.
QGIS Development Team (2019). QGIS Geographic Information System. Open Source Geospatial Foundation Project. http://qgis.osgeo.org.

^{7}Be normalized trend interpolated into a global map

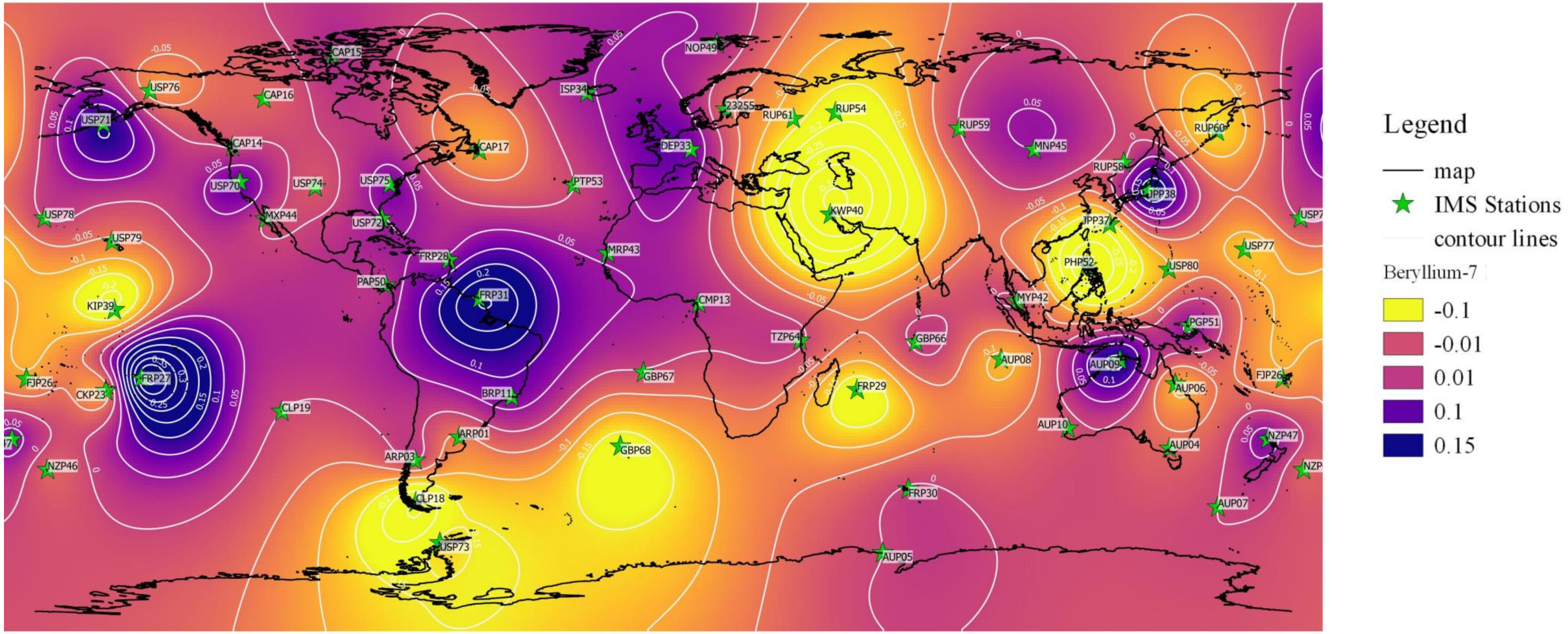

2008

Map executed with QGIS.
QGIS Development Team (2019). QGIS Geographic Information System. Open Source Geospatial Foundation Project. http://qgis.osgeo.org.

^{7}Be normalized trend interpolated into a global map

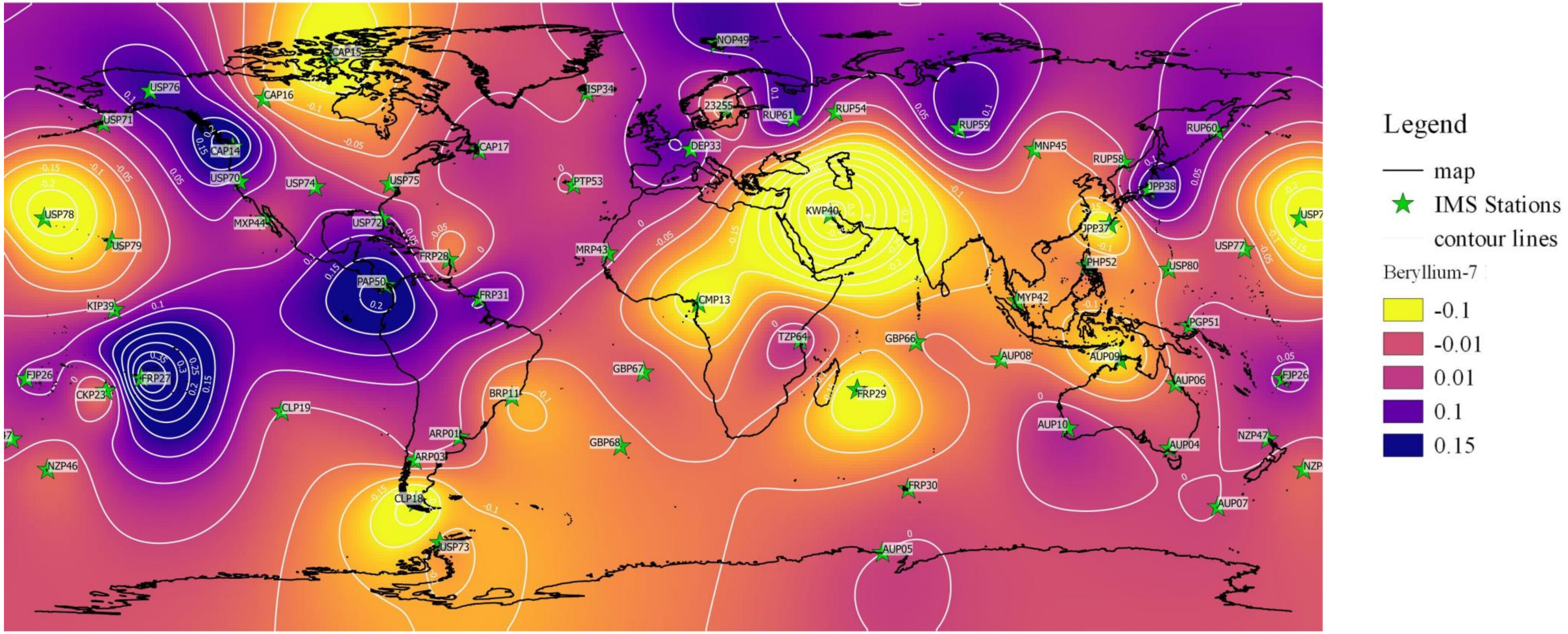

2009

Map executed with QGIS.
QGIS Development Team (2019). QGIS Geographic Information System. Open Source Geospatial Foundation Project. http://qgis.osgeo.org.

^{7}Be normalized trend interpolated into a global map

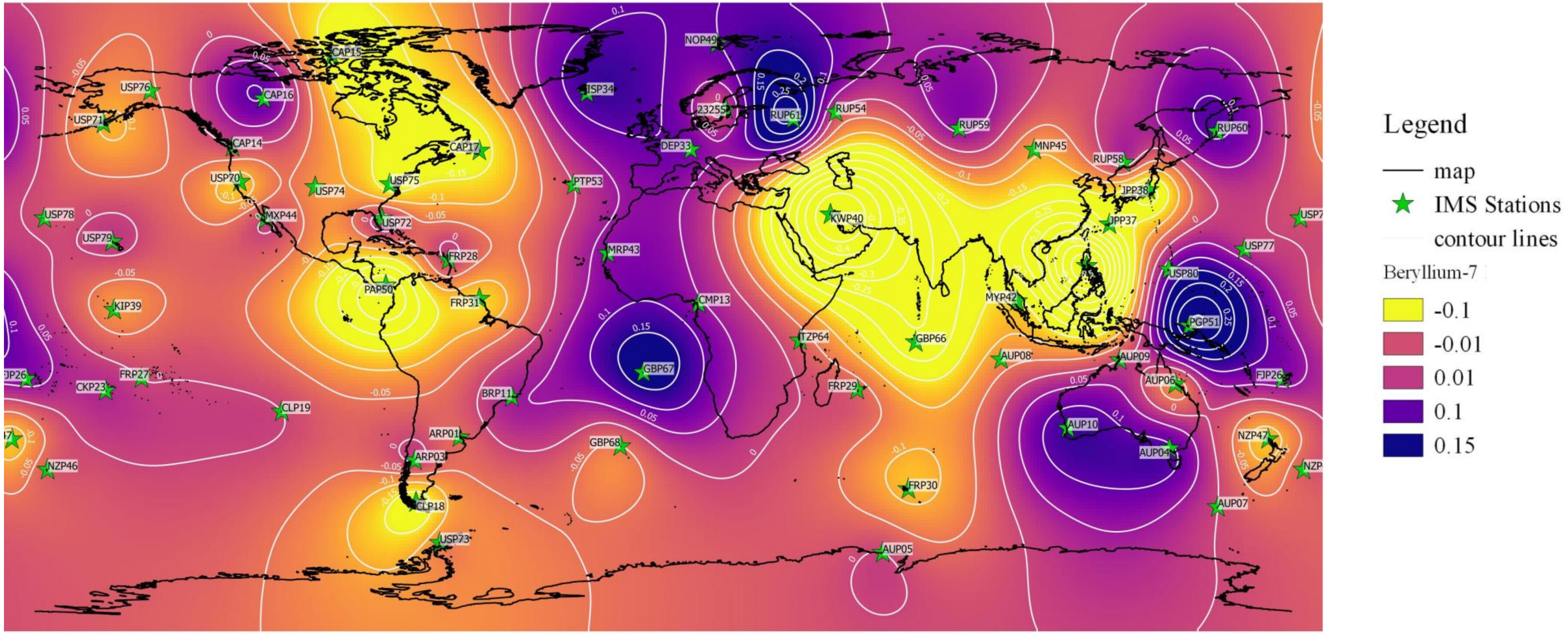

2010

Map executed with QGIS.
QGIS Development Team (2019). QGIS Geographic Information System. Open Source Geospatial Foundation Project. http://qgis.osgeo.org.

^{7}Be normalized trend interpolated into a global map

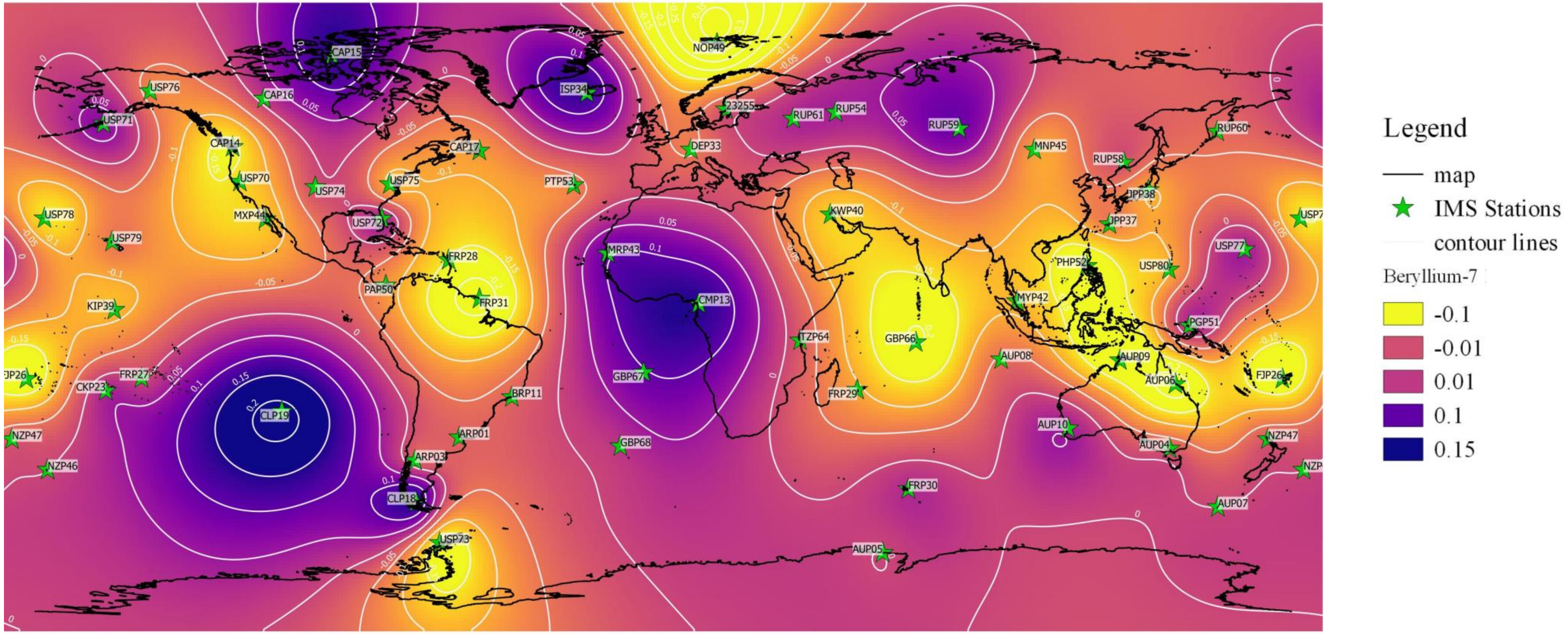

2011

Map executed with QGIS.
QGIS Development Team (2019). QGIS Geographic Information System. Open Source Geospatial Foundation Project. http://qgis.osgeo.org.

^{7}Be normalized trend interpolated into a global map

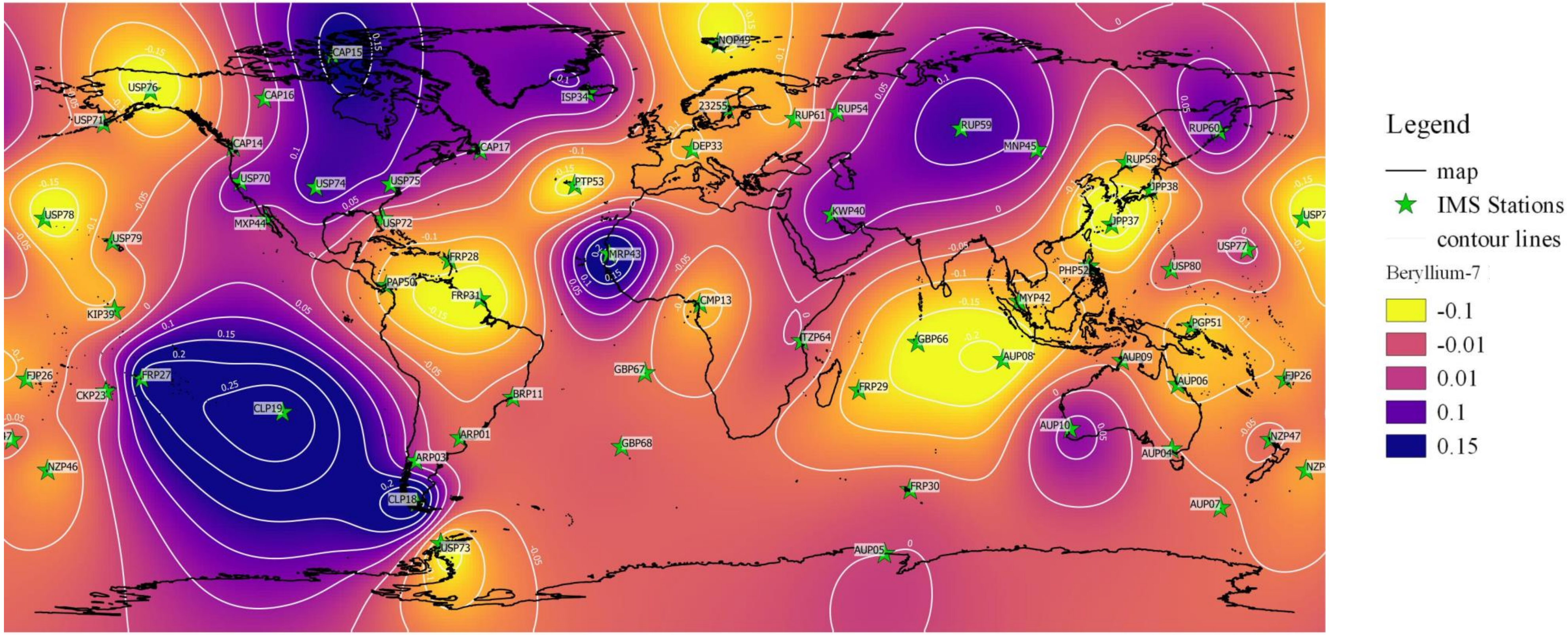

2012

Map executed with QGIS.
QGIS Development Team (2019). QGIS Geographic Information System. Open Source Geospatial Foundation Project. http://qgis.osgeo.org.

^{7}Be normalized trend interpolated into a global map

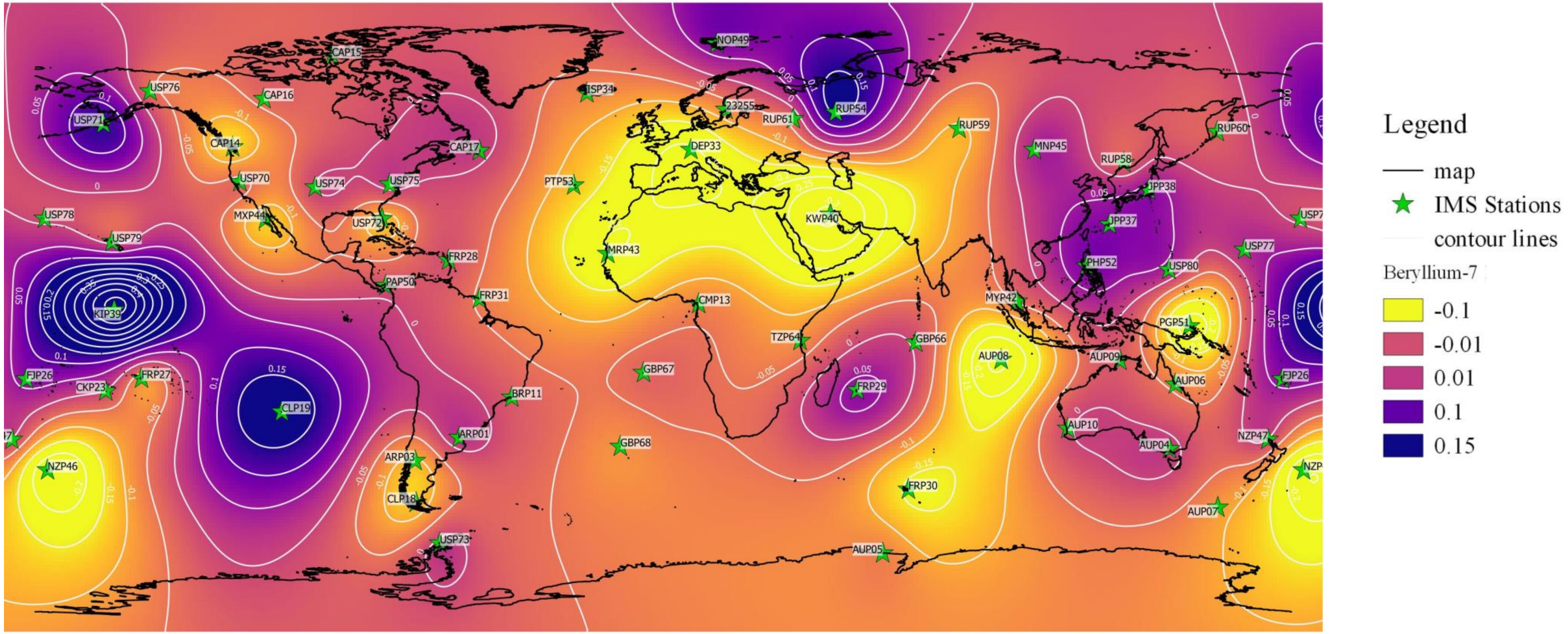

2013

Map executed with QGIS.
QGIS Development Team (2019). QGIS Geographic Information System. Open Source Geospatial Foundation Project. http://qgis.osgeo.org.

^{7}Be normalized trend interpolated into a global map

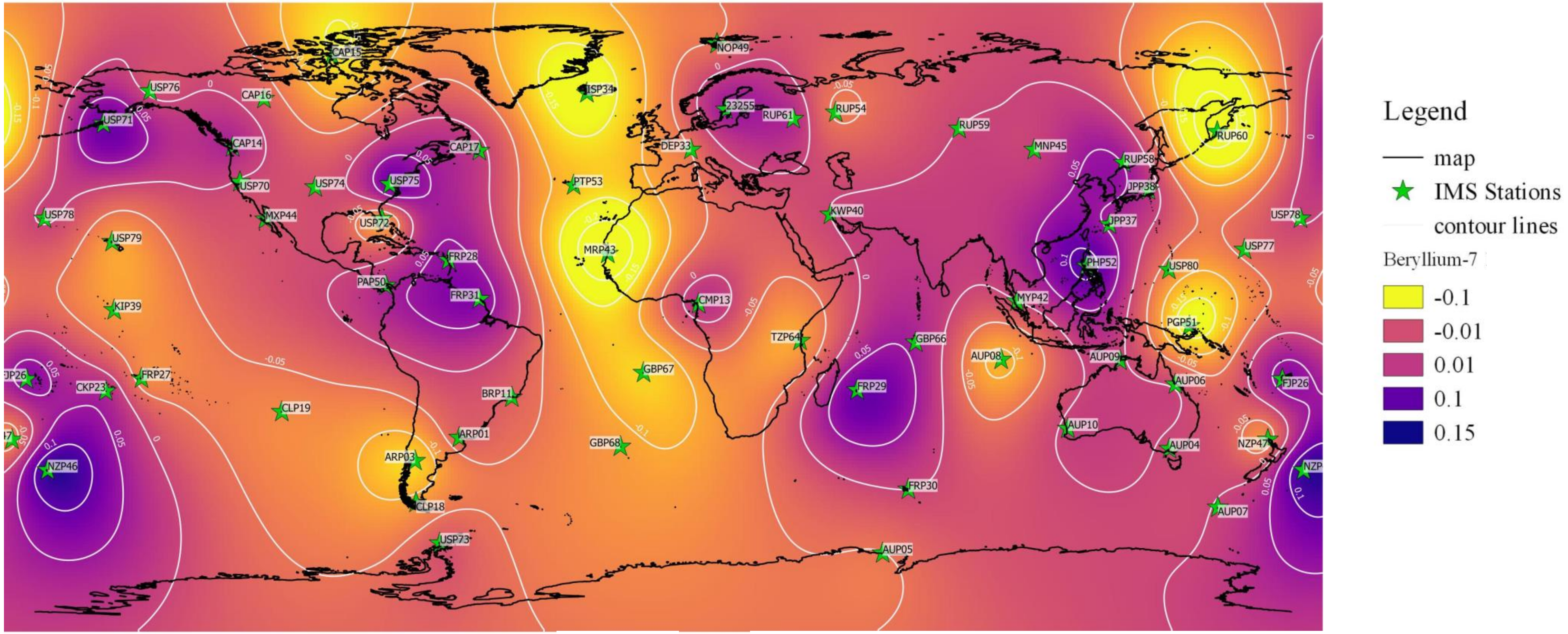

2014

Map executed with QGIS.
QGIS Development Team (2019). QGIS Geographic Information System. Open Source Geospatial Foundation Project. http://qgis.osgeo.org.

^{7}Be normalized trend interpolated into a global map

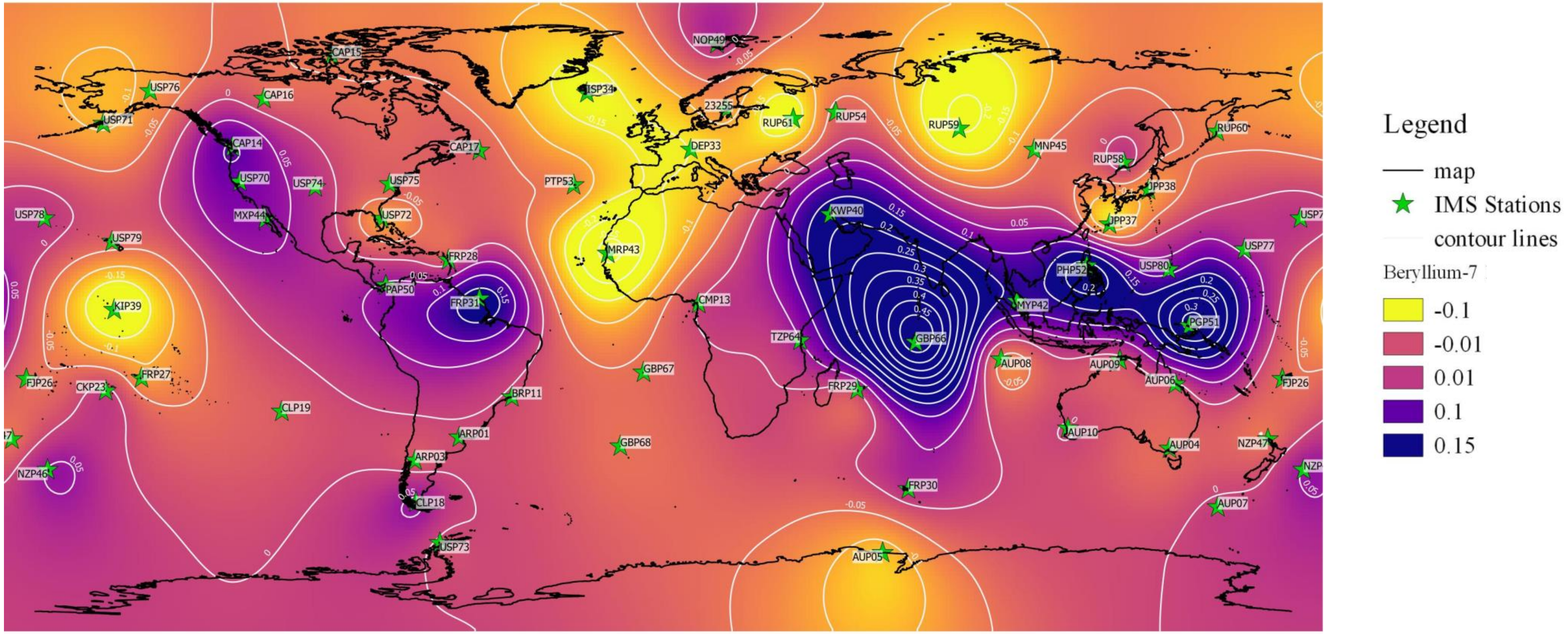

2015

Map executed with QGIS.
QGIS Development Team (2019). QGIS Geographic Information System. Open Source Geospatial Foundation Project. http://qgis.osgeo.org.

^{7}Be normalized trend interpolated into a global map

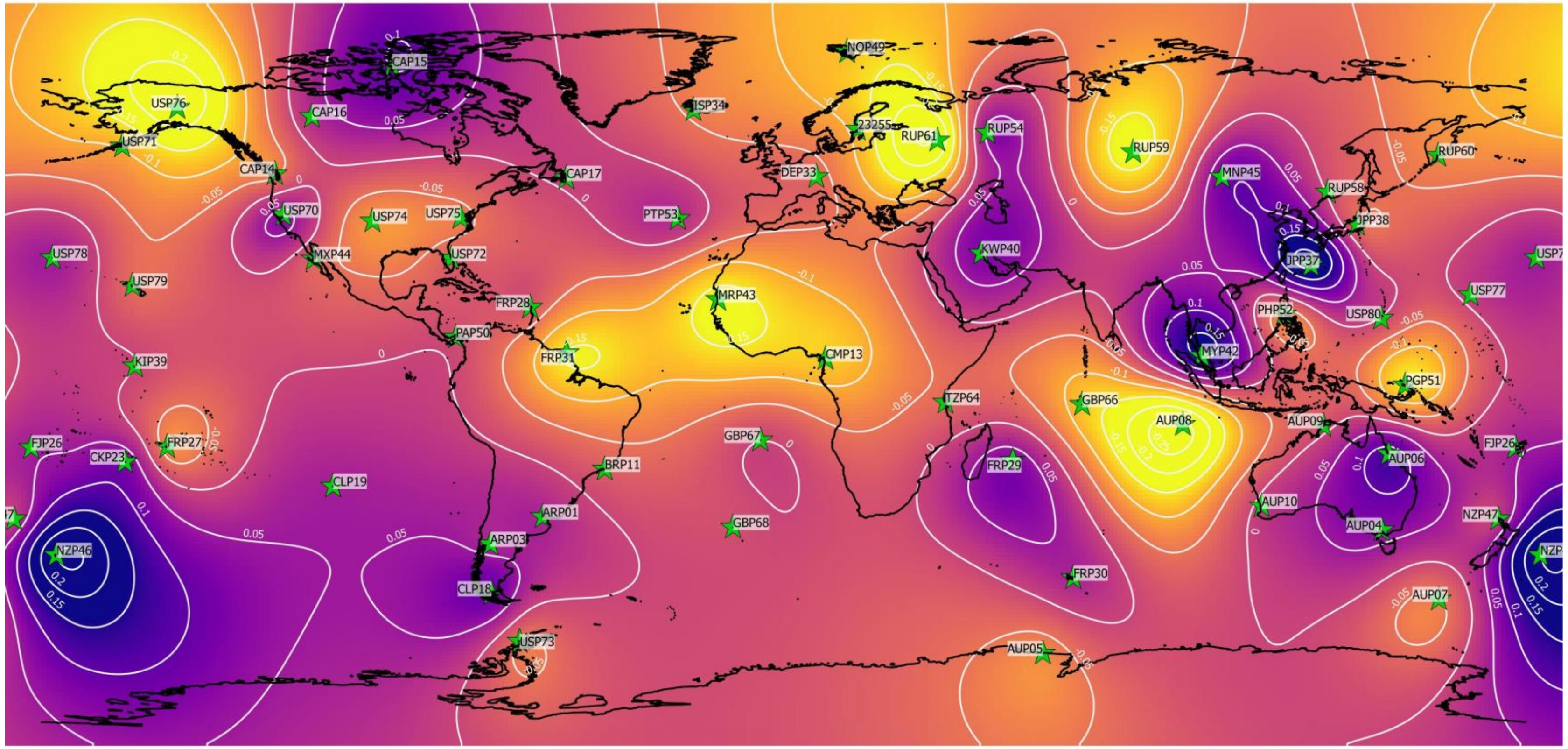

Legend
map
IMS Stations
contour lines
Beryllium-7
-0.1
-0.01
0.01
0.1
0.15

2016

Map executed with QGIS.
QGIS Development Team (2019). QGIS Geographic Information System. Open Source Geospatial Foundation Project. http://qgis.osgeo.org.

^{7}Be normalized trend interpolated into a global map

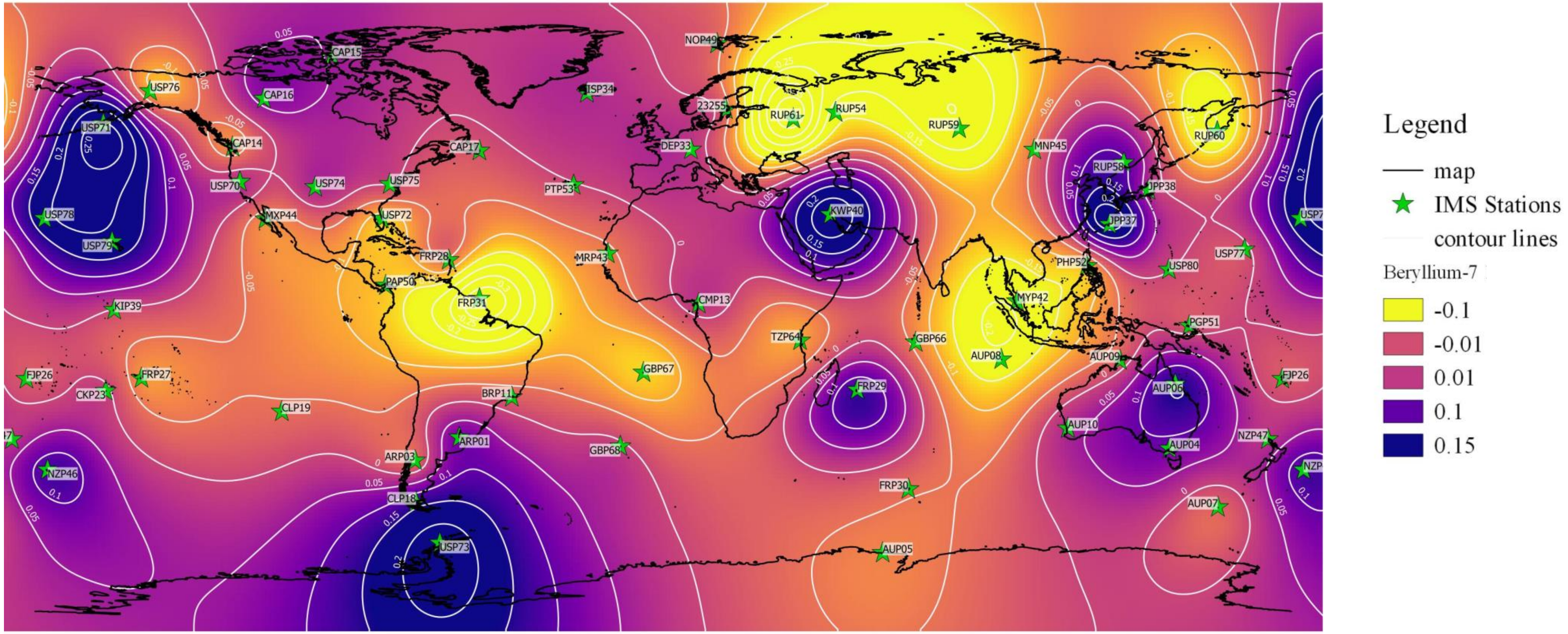

2017

Map executed with QGIS.
QGIS Development Team (2019). QGIS Geographic Information System. Open Source Geospatial Foundation Project. http://qgis.osgeo.org.

^{7}Be normalized trend interpolated into a global map

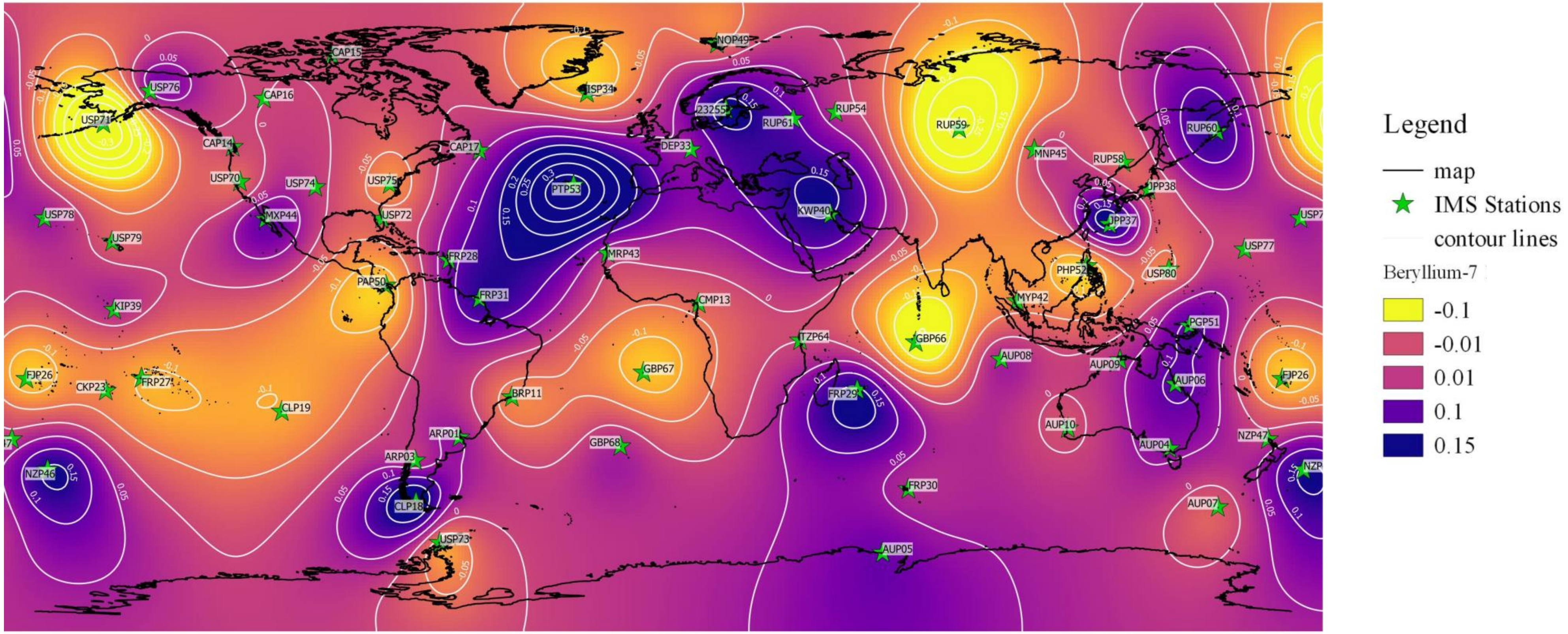

2018

Map executed with QGIS.
QGIS Development Team (2019). QGIS Geographic Information System. Open Source Geospatial Foundation Project. http://qgis.osgeo.org.

^{7}Be normalized trend interpolated into a global map

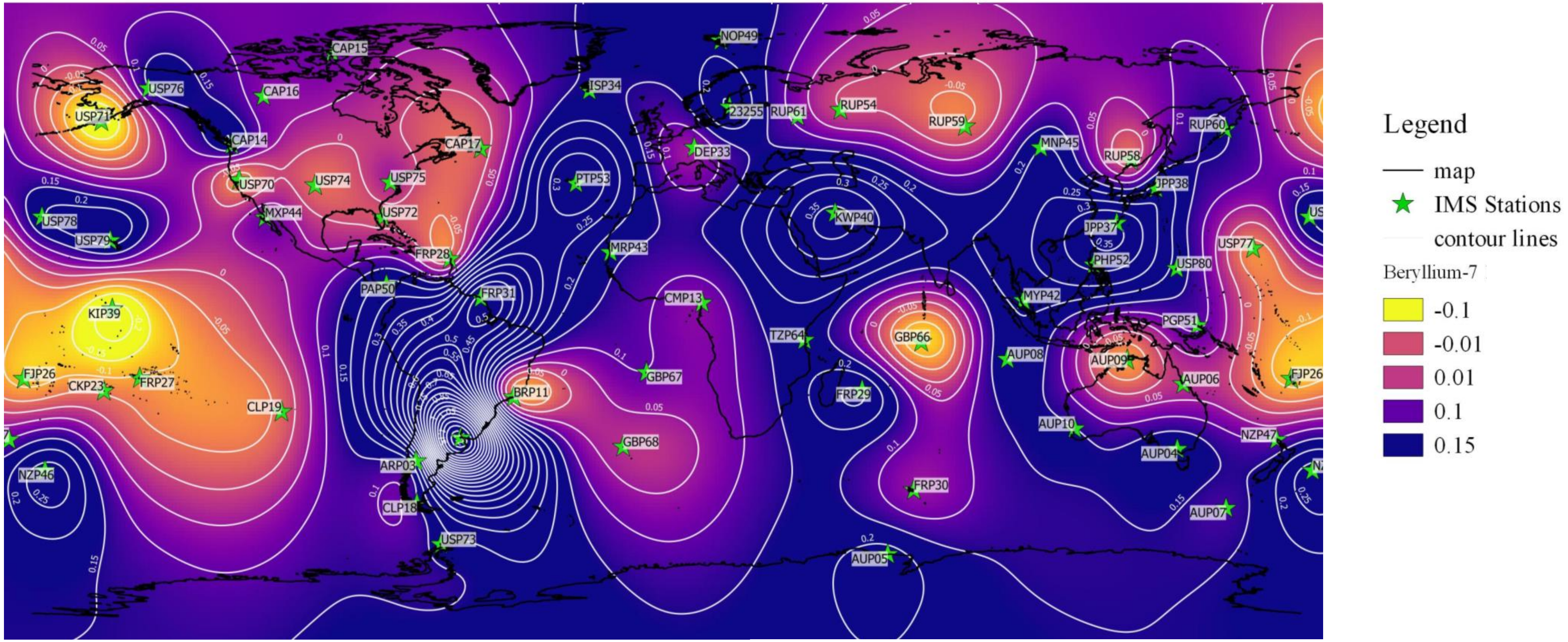

2019

Map executed with QGIS.
QGIS Development Team (2019). QGIS Geographic Information System. Open Source Geospatial Foundation Project. http://qgis.osgeo.org.

^{7}Be normalized trend interpolated into a global map

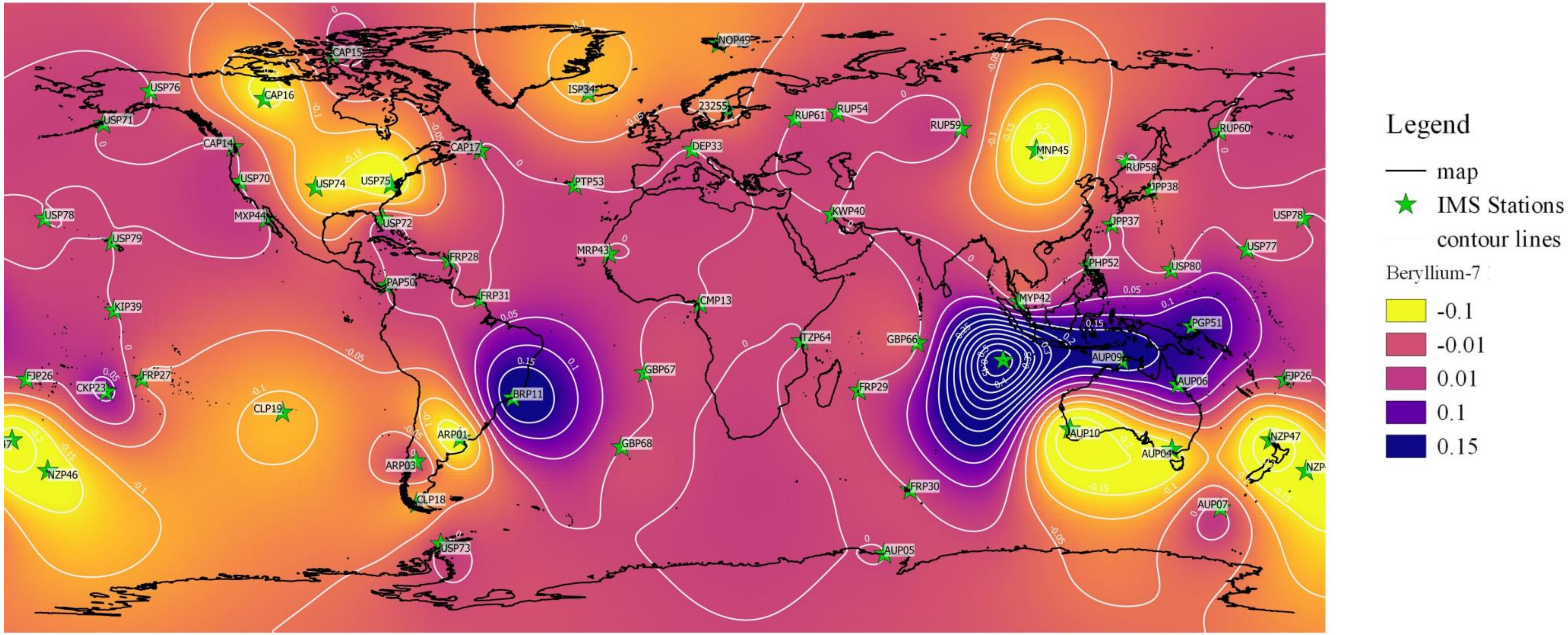

2003

Map executed with QGIS.
QGIS Development Team (2019). QGIS Geographic Information System. Open Source Geospatial Foundation Project. http://qgis.osgeo.org.

^{7}Be normalized trend interpolated into a global map

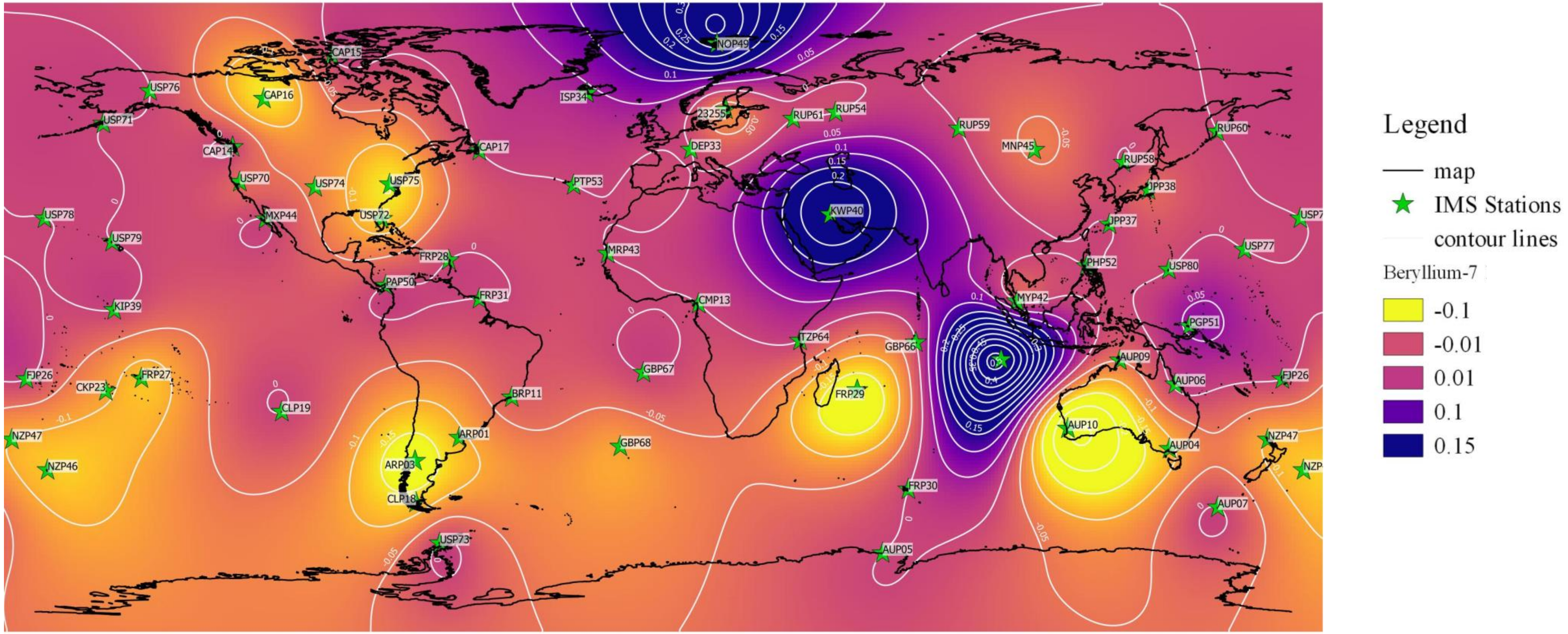

2004

Map executed with QGIS.
QGIS Development Team (2019). QGIS Geographic Information System. Open Source Geospatial Foundation Project. http://qgis.osgeo.org.

^{7}Be normalized trend interpolated into a global map

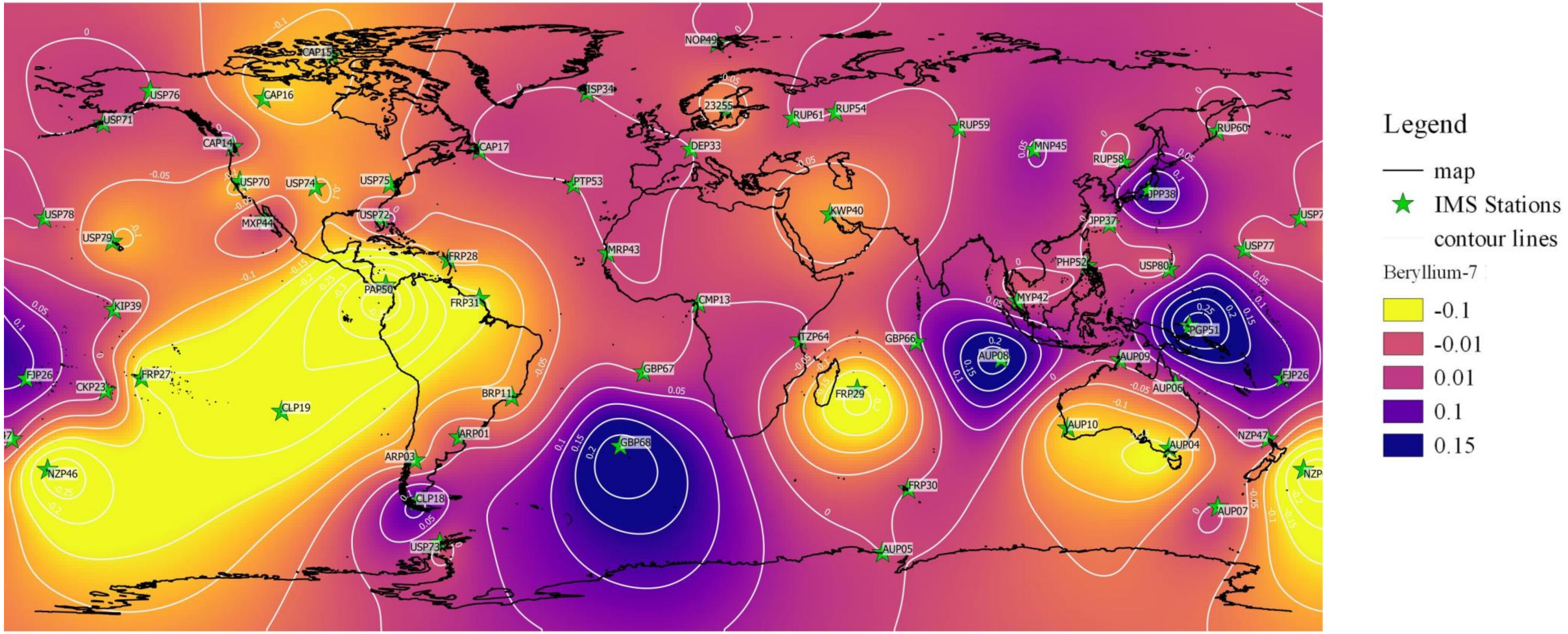

2005

Map executed with QGIS.
QGIS Development Team (2019). QGIS Geographic Information System. Open Source Geospatial Foundation Project. http://qgis.osgeo.org.

^{7}Be normalized trend interpolated into a global map

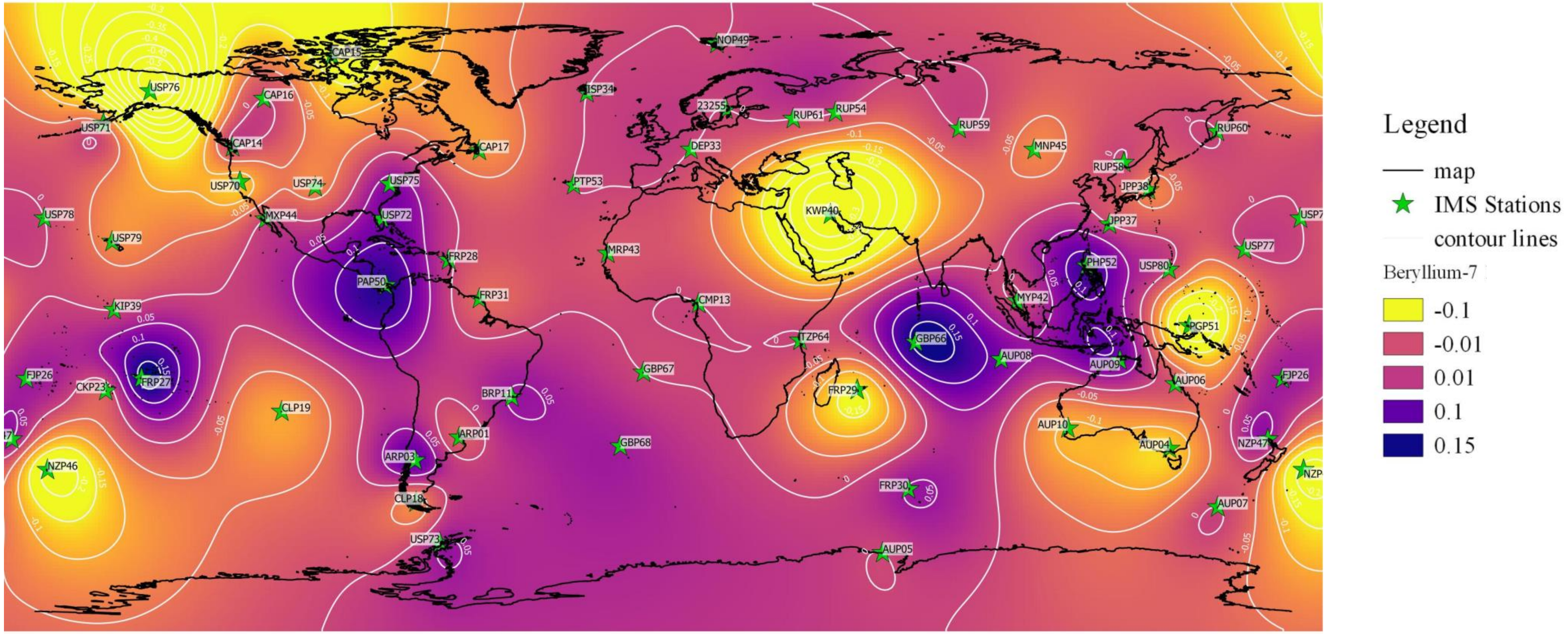

2006

Map executed with QGIS.
QGIS Development Team (2019). QGIS Geographic Information System. Open Source Geospatial Foundation Project. http://qgis.osgeo.org.

^{7}Be normalized trend interpolated into a global map

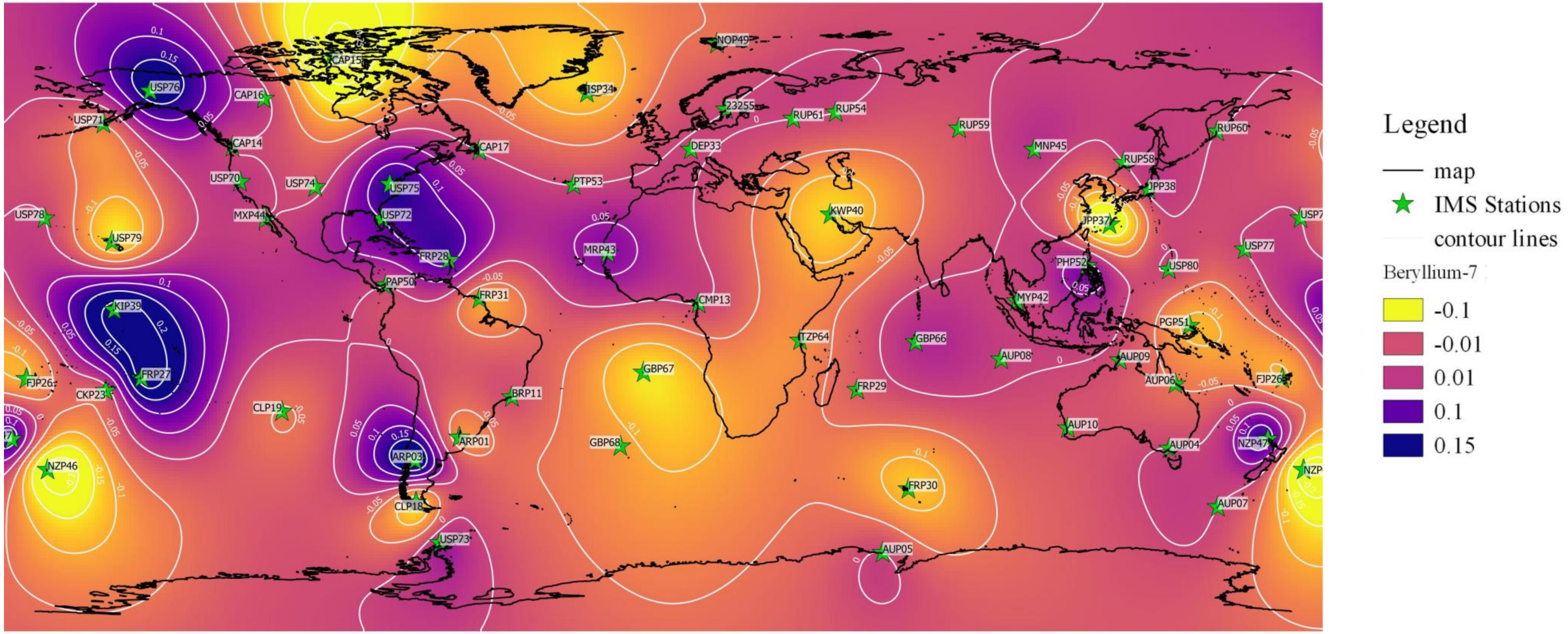

2007

Map executed with QGIS.
QGIS Development Team (2019). QGIS Geographic Information System. Open Source Geospatial Foundation Project. http://qgis.osgeo.org.

^{7}Be normalized trend interpolated into a global map

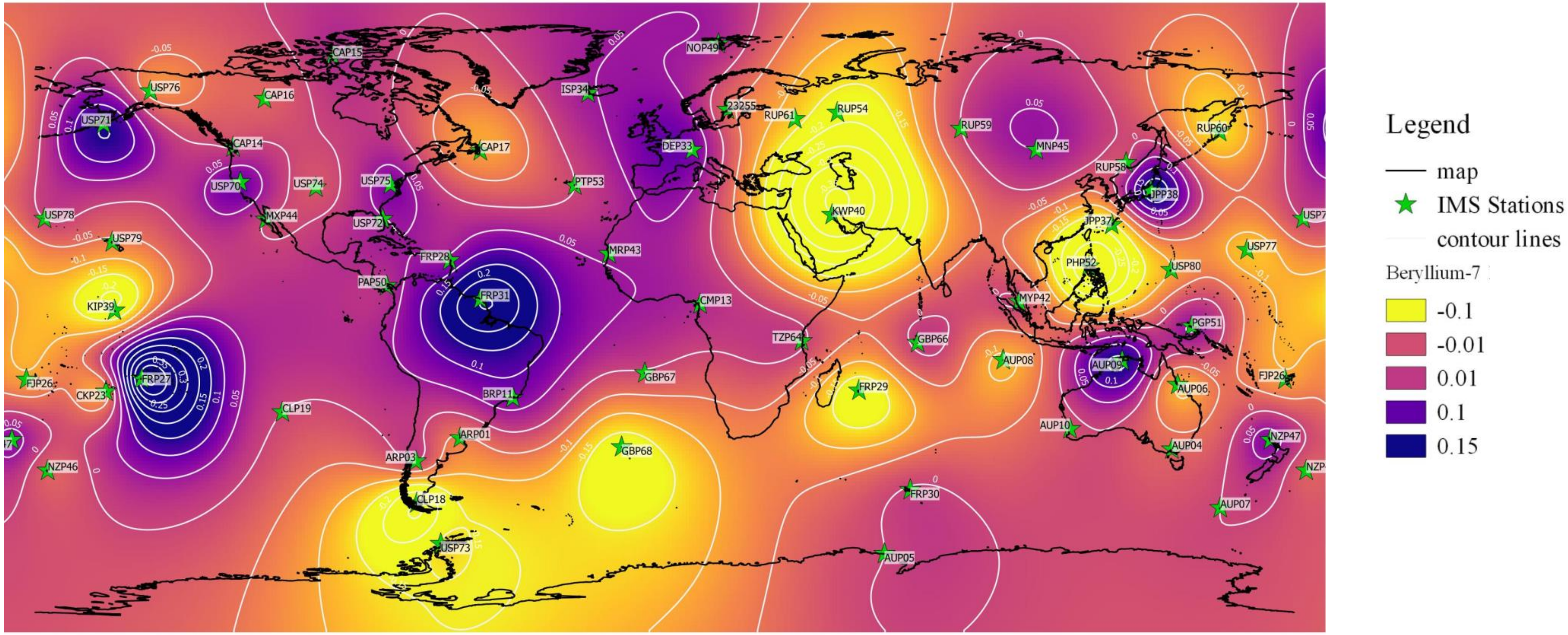

2008

Map executed with QGIS.
QGIS Development Team (2019). QGIS Geographic Information System. Open Source Geospatial Foundation Project. http://qgis.osgeo.org.

^{7}Be normalized trend interpolated into a global map

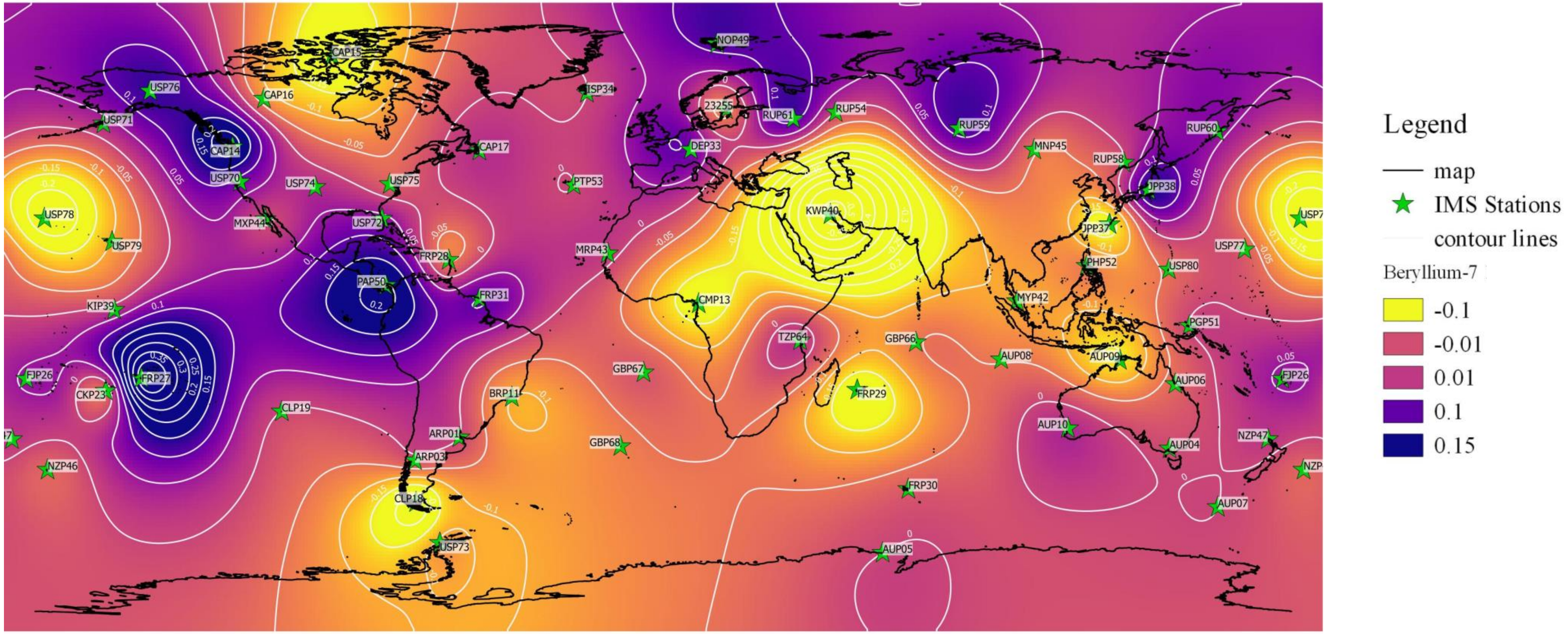

2009

Map executed with QGIS.
QGIS Development Team (2019). QGIS Geographic Information System. Open Source Geospatial Foundation Project. http://qgis.osgeo.org.

^{7}Be normalized trend interpolated into a global map

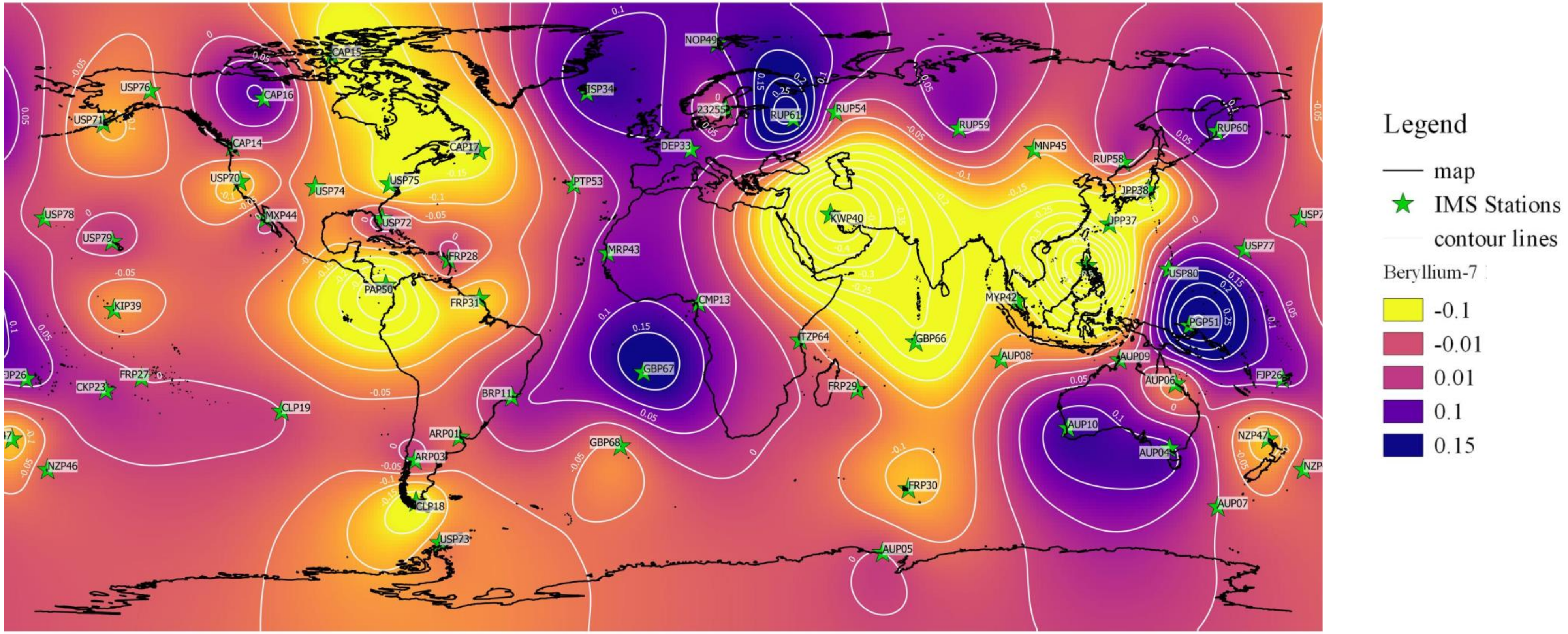

2010

Map executed with QGIS.
QGIS Development Team (2019). QGIS Geographic Information System. Open Source Geospatial Foundation Project. http://qgis.osgeo.org.

^{7}Be normalized trend interpolated into a global map

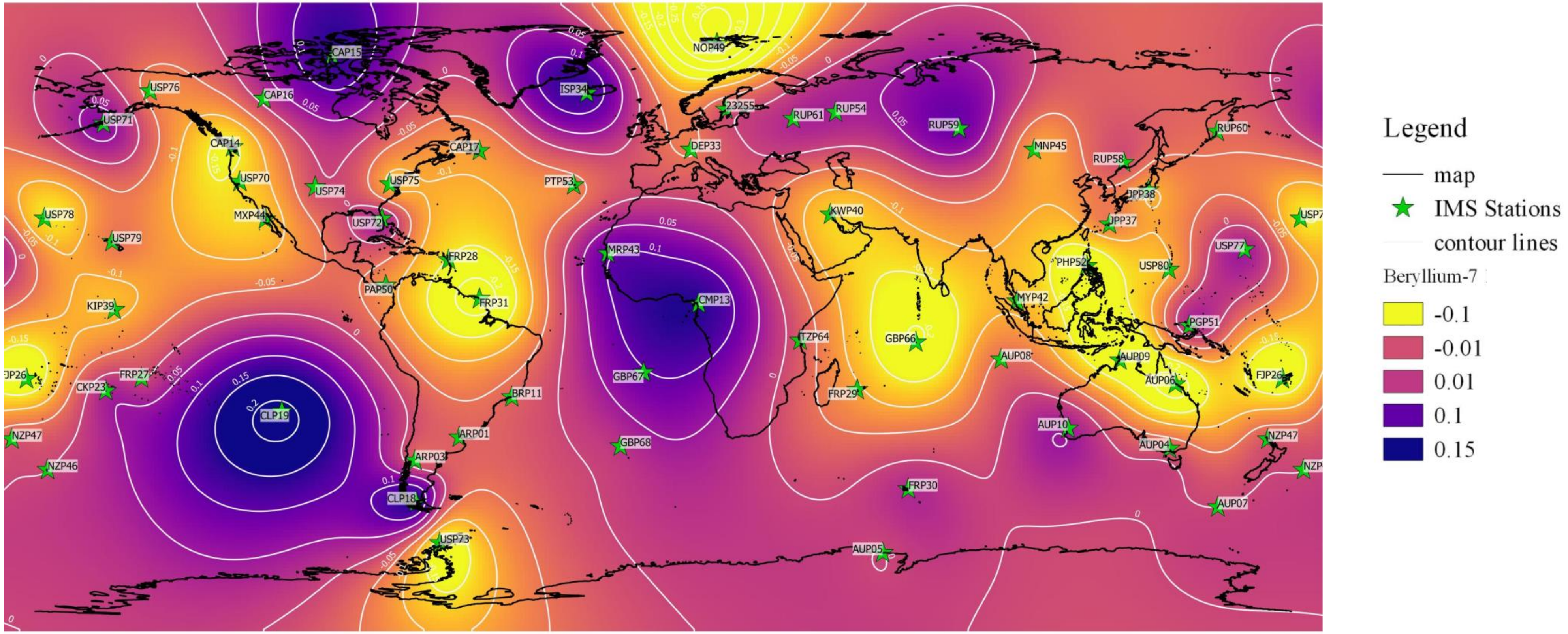

2011

Map executed with QGIS.
QGIS Development Team (2019). QGIS Geographic Information System. Open Source Geospatial Foundation Project. http://qgis.osgeo.org.

^{7}Be normalized trend interpolated into a global map

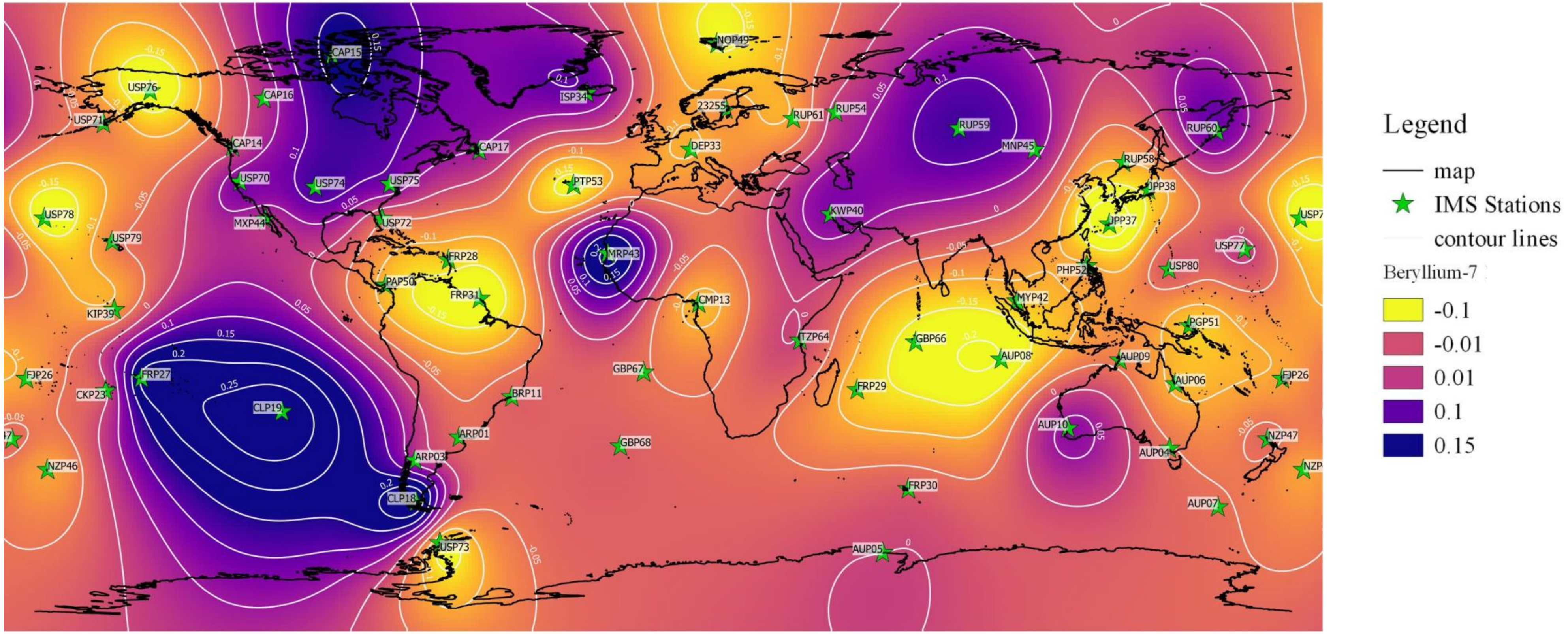

2012

Map executed with QGIS.
QGIS Development Team (2019). QGIS Geographic Information System. Open Source Geospatial Foundation Project. http://qgis.osgeo.org.

^{7}Be normalized trend interpolated into a global map

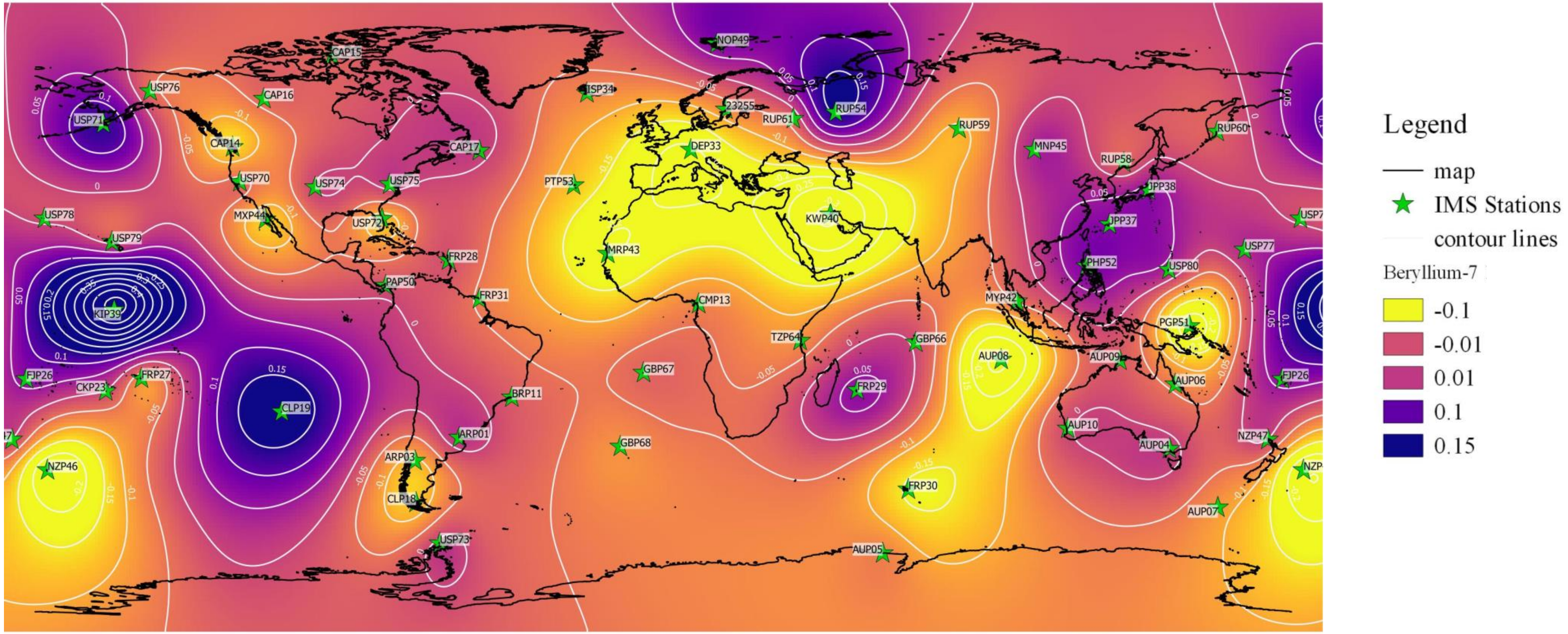

2013

Map executed with QGIS.
QGIS Development Team (2019). QGIS Geographic Information System. Open Source Geospatial Foundation Project. http://qgis.osgeo.org.

^{7}Be normalized trend interpolated into a global map

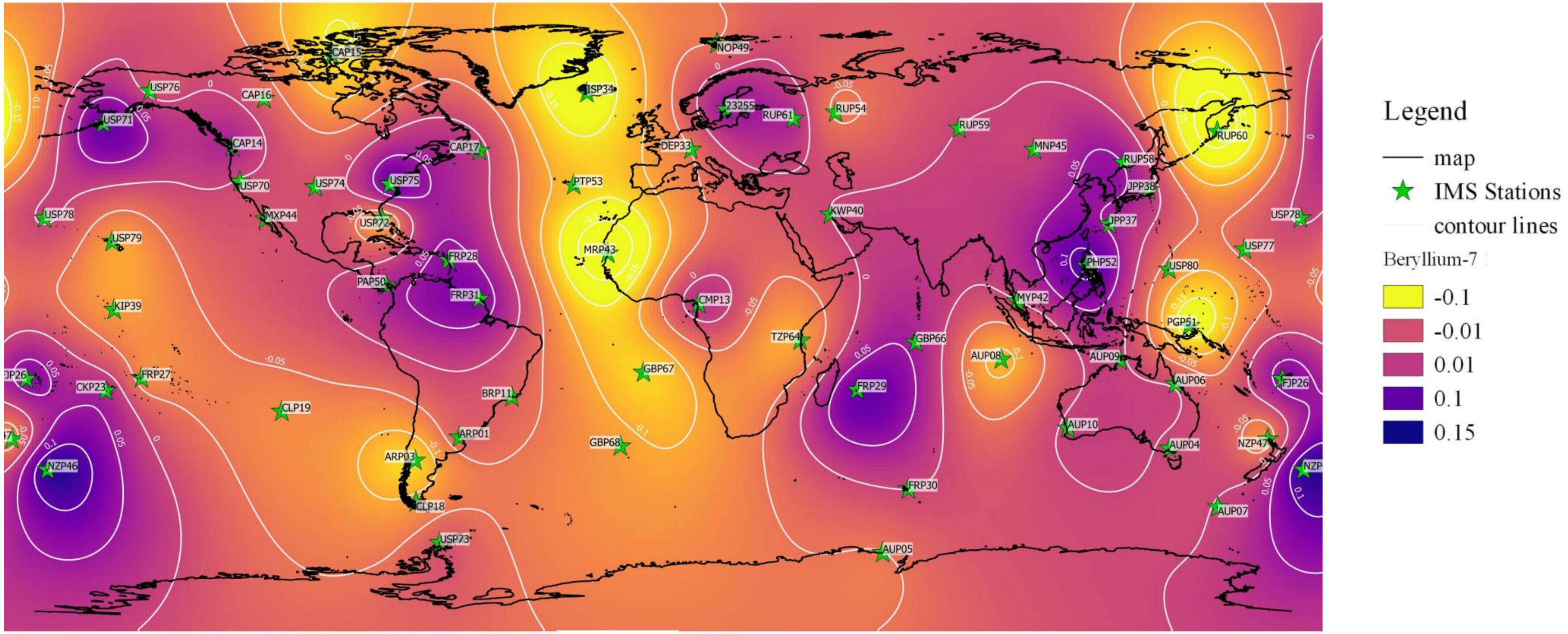

2014

Map executed with QGIS.
QGIS Development Team (2019). QGIS Geographic Information System. Open Source Geospatial Foundation Project. http://qgis.osgeo.org.

^{7}Be normalized trend interpolated into a global map

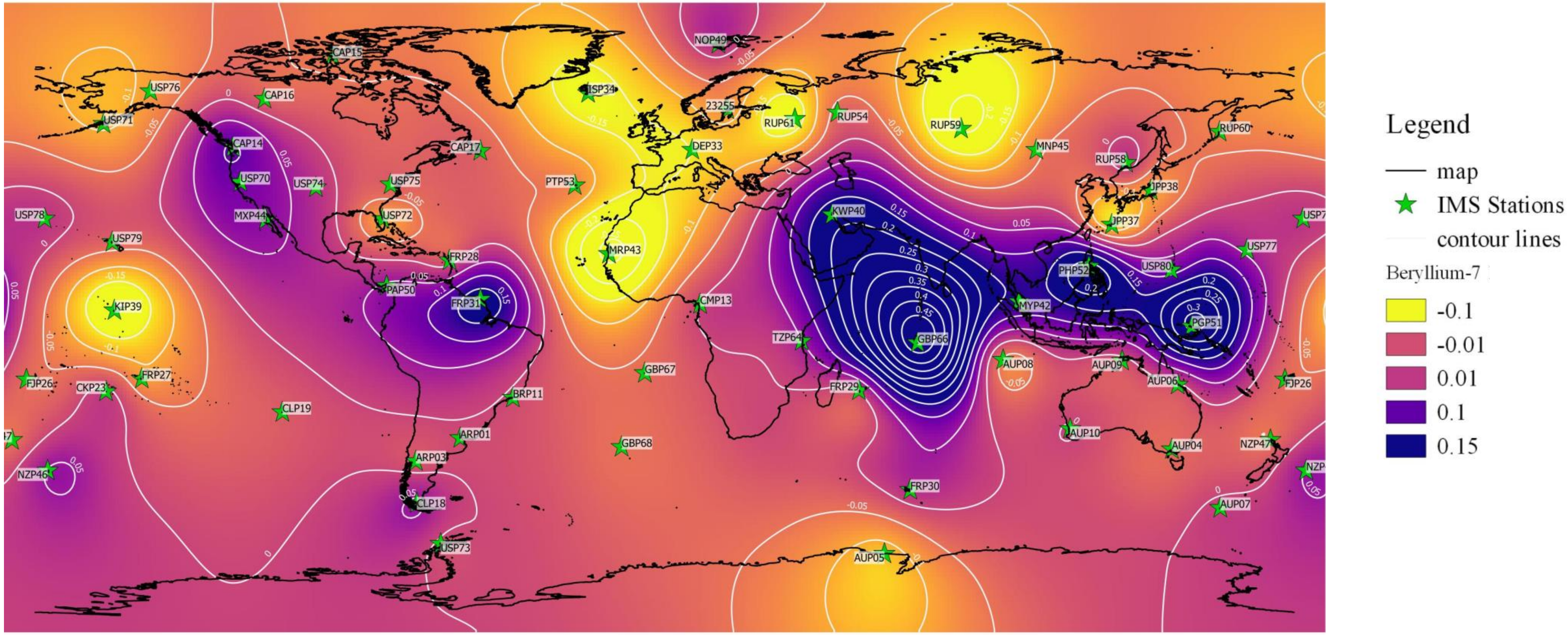

2015

Map executed with QGIS.
QGIS Development Team (2019). QGIS Geographic Information System. Open Source Geospatial Foundation Project. http://qgis.osgeo.org.

^{7}Be normalized trend interpolated into a global map

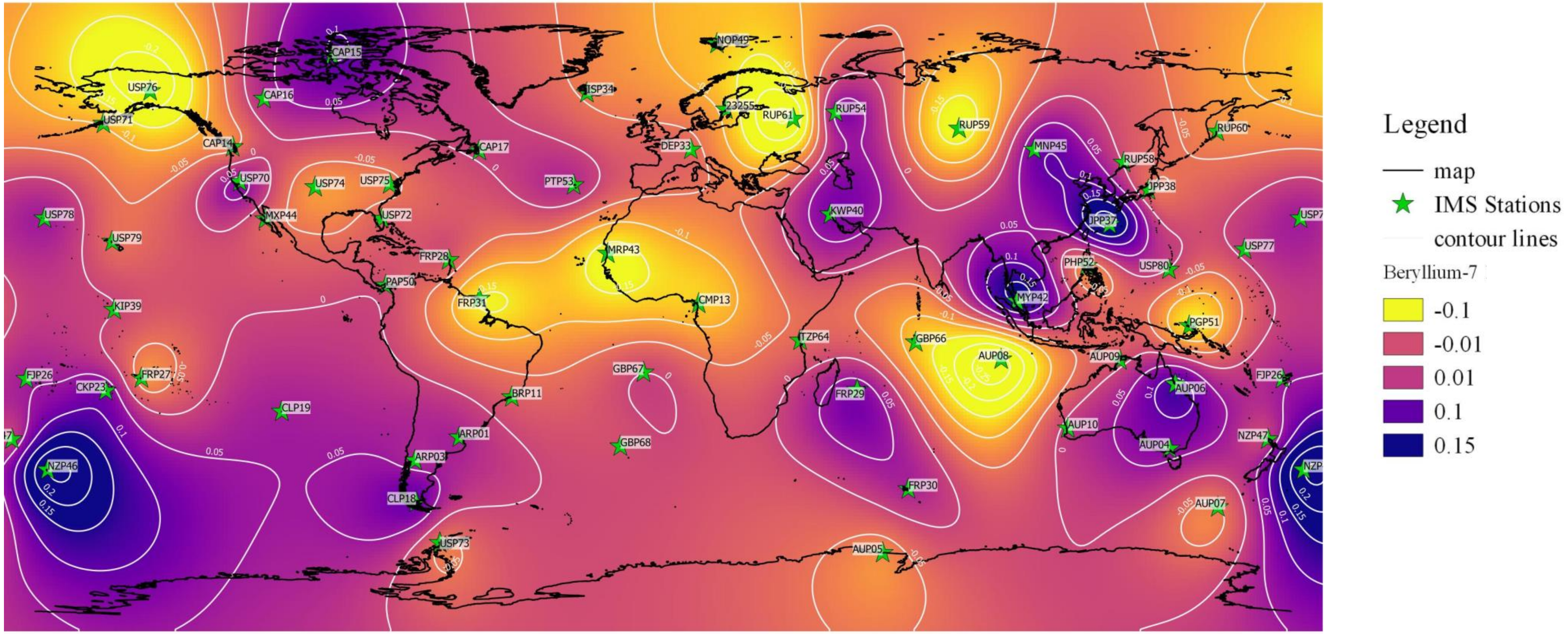

2016

Map executed with QGIS.
QGIS Development Team (2019). QGIS Geographic Information System. Open Source Geospatial Foundation Project. http://qgis.osgeo.org.

^{7}Be normalized trend interpolated into a global map

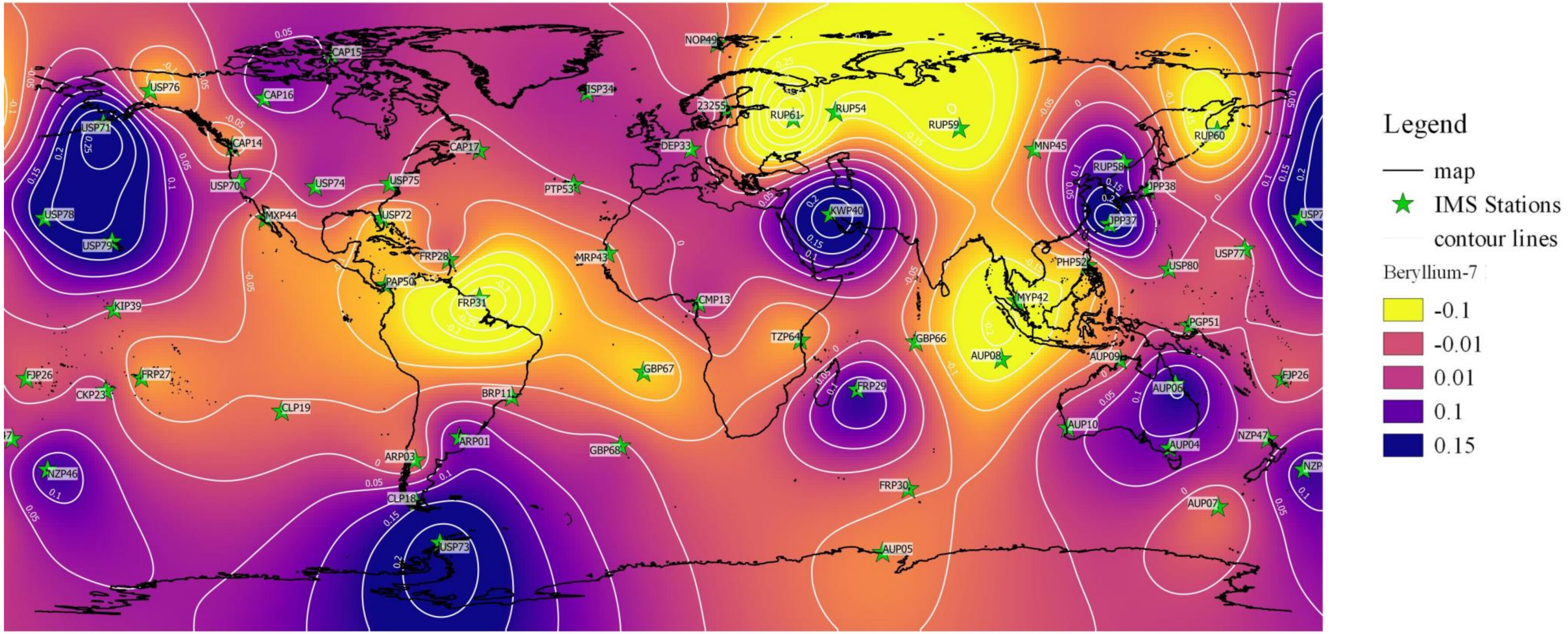

2017

Map executed with QGIS.
QGIS Development Team (2019). QGIS Geographic Information System. Open Source Geospatial Foundation Project. http://qgis.osgeo.org.

^{7}Be normalized trend interpolated into a global map

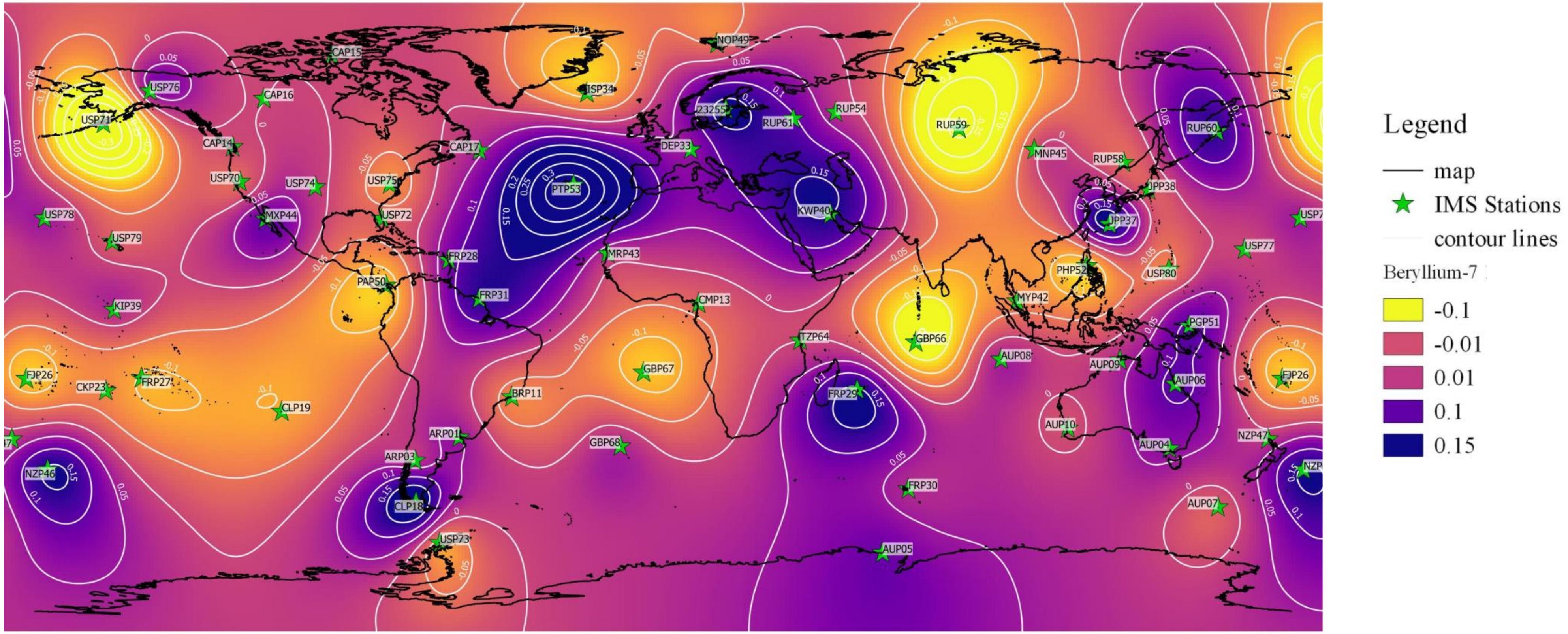

2018

Map executed with QGIS.
QGIS Development Team (2019). QGIS Geographic Information System. Open Source Geospatial Foundation Project. http://qgis.osgeo.org.

^{7}Be normalized trend interpolated into a global map

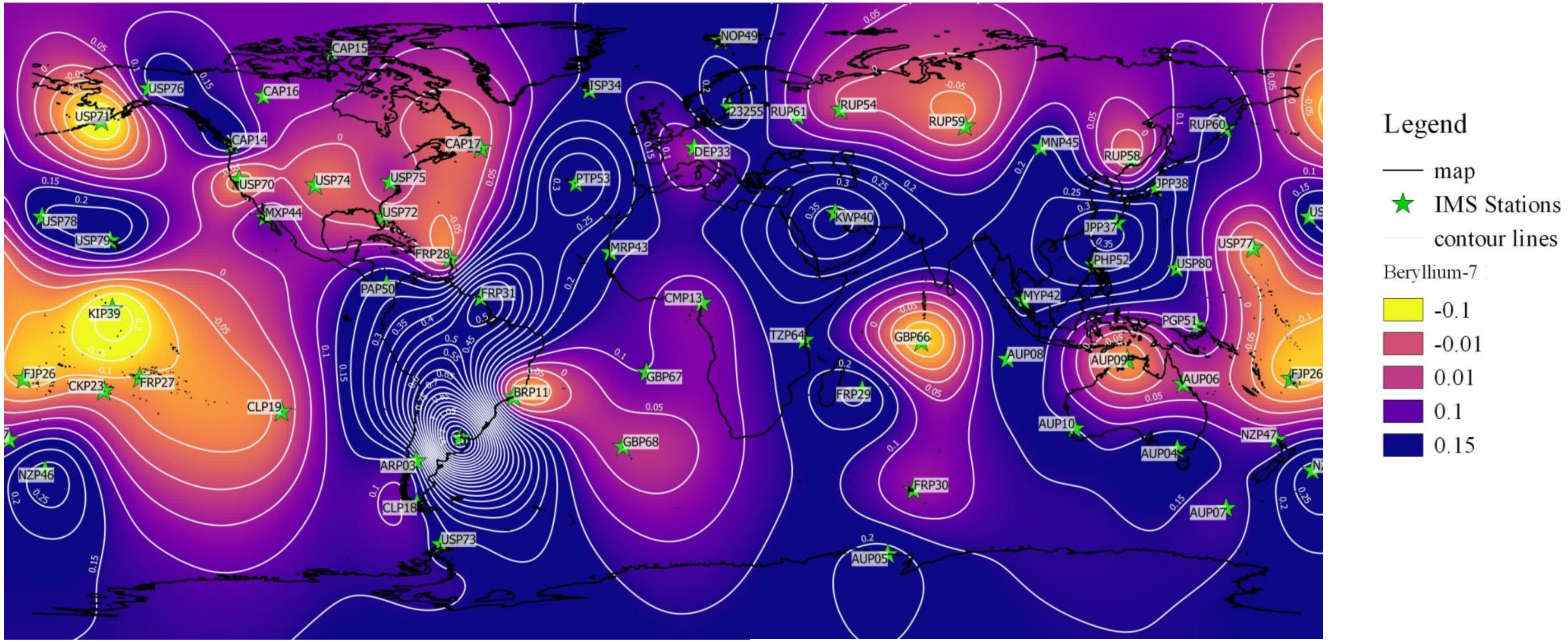

2019

Map executed with QGIS.
QGIS Development Team (2019). QGIS Geographic Information System. Open Source Geospatial Foundation Project. http://qgis.osgeo.org.

^{7}Be normalized trend interpolated into a global map

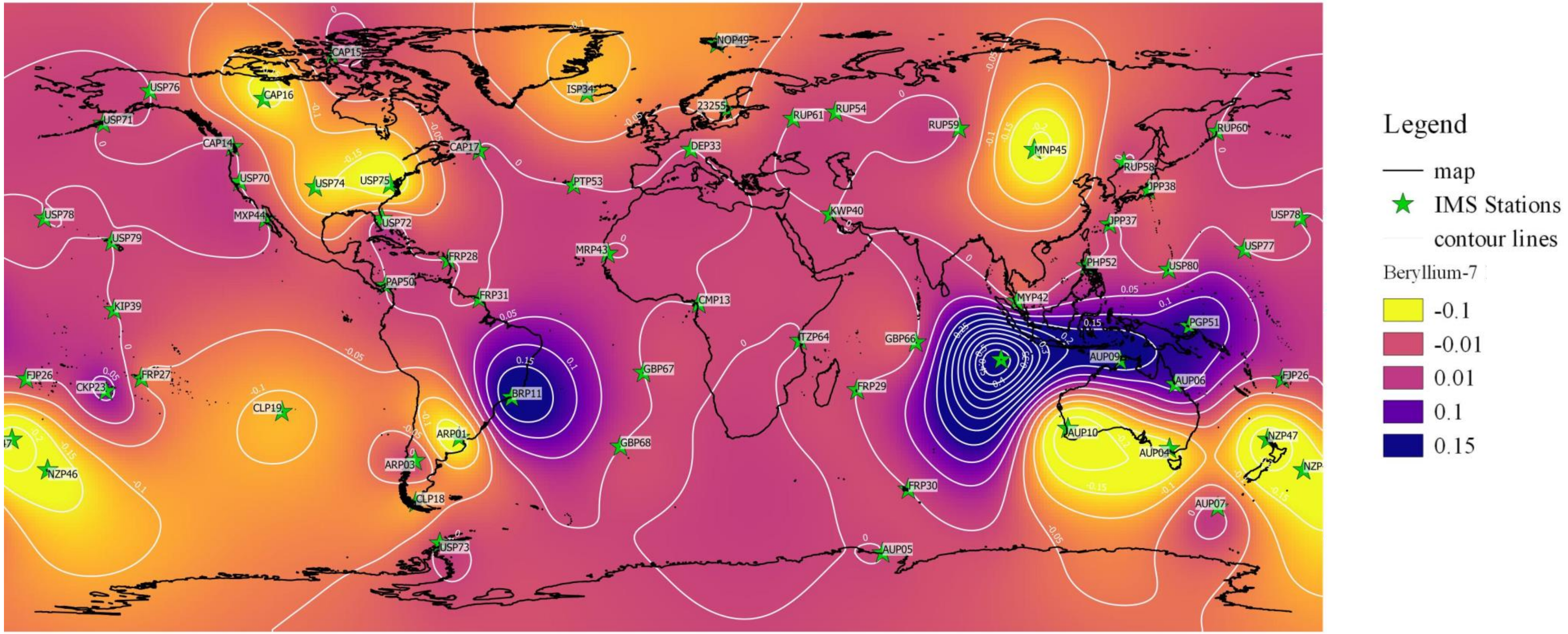

2003

Map executed with QGIS.
QGIS Development Team (2019). QGIS Geographic Information System. Open Source Geospatial Foundation Project. http://qgis.osgeo.org.

^{7}Be normalized trend interpolated into a global map

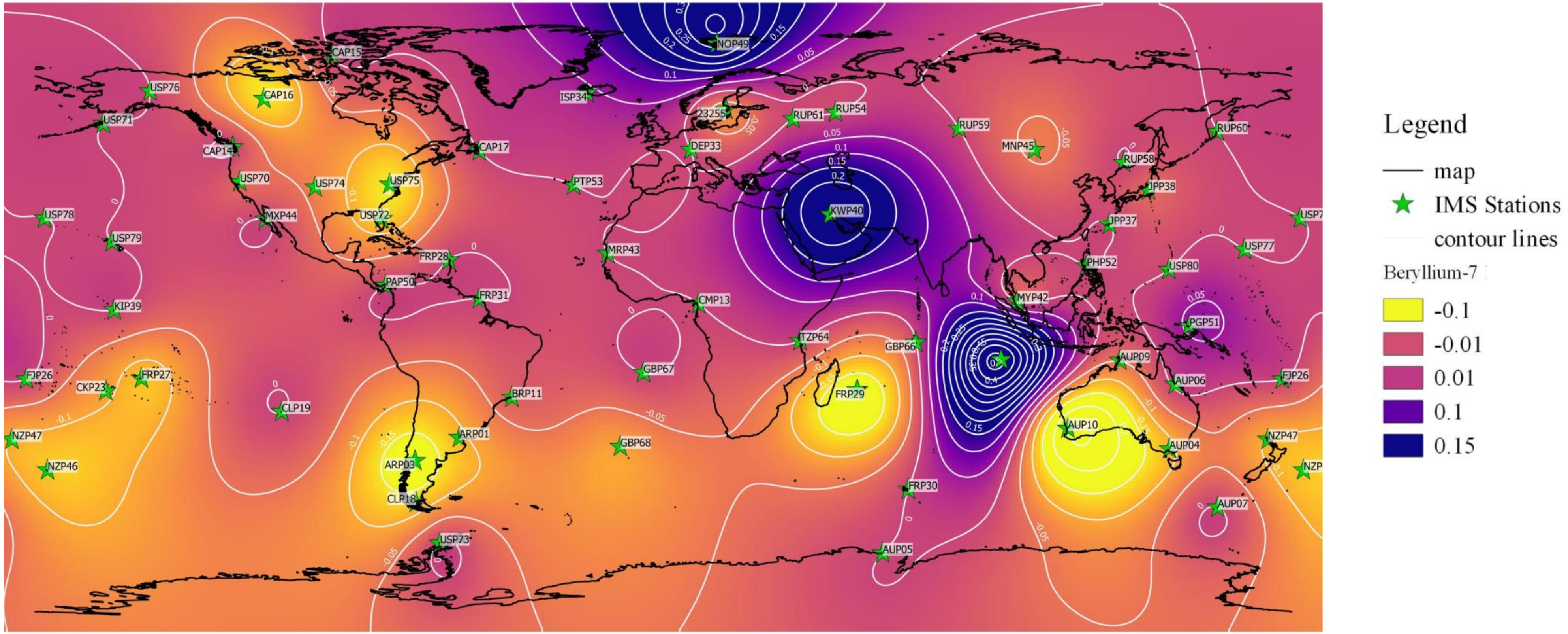

2004

Map executed with QGIS.
QGIS Development Team (2019). QGIS Geographic Information System. Open Source Geospatial Foundation Project. http://qgis.osgeo.org.

^{7}Be normalized trend interpolated into a global map

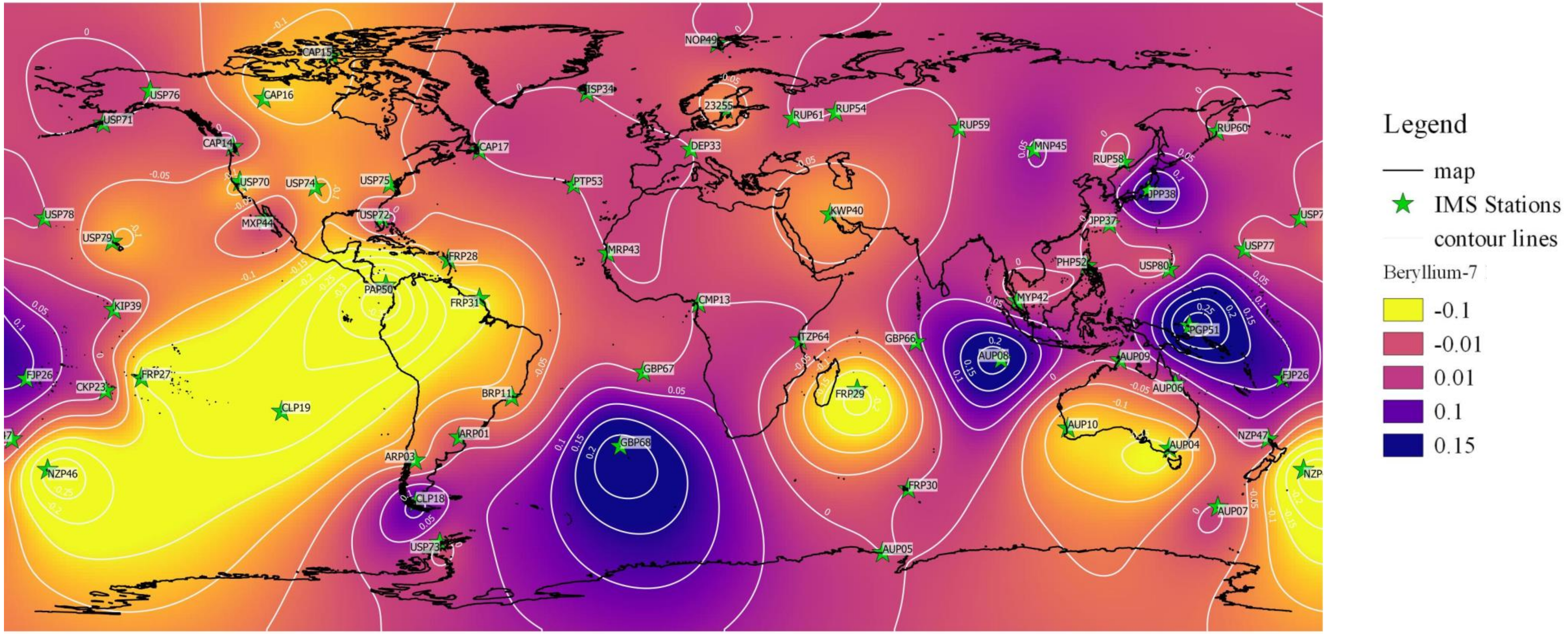

2005

Map executed with QGIS.
QGIS Development Team (2019). QGIS Geographic Information System. Open Source Geospatial Foundation Project. http://qgis.osgeo.org.

^{7}Be normalized trend interpolated into a global map

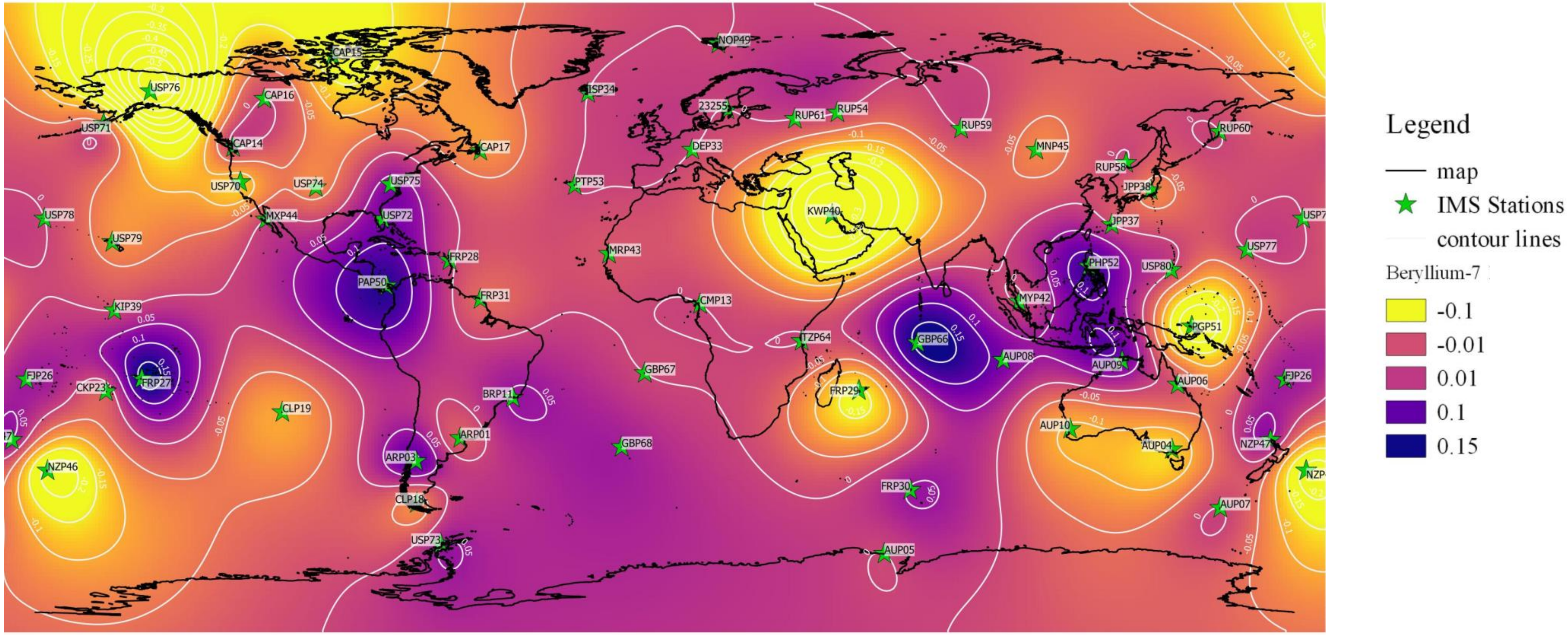

2006

Map executed with QGIS.
QGIS Development Team (2019). QGIS Geographic Information System. Open Source Geospatial Foundation Project. http://qgis.osgeo.org.

^{7}Be normalized trend interpolated into a global map

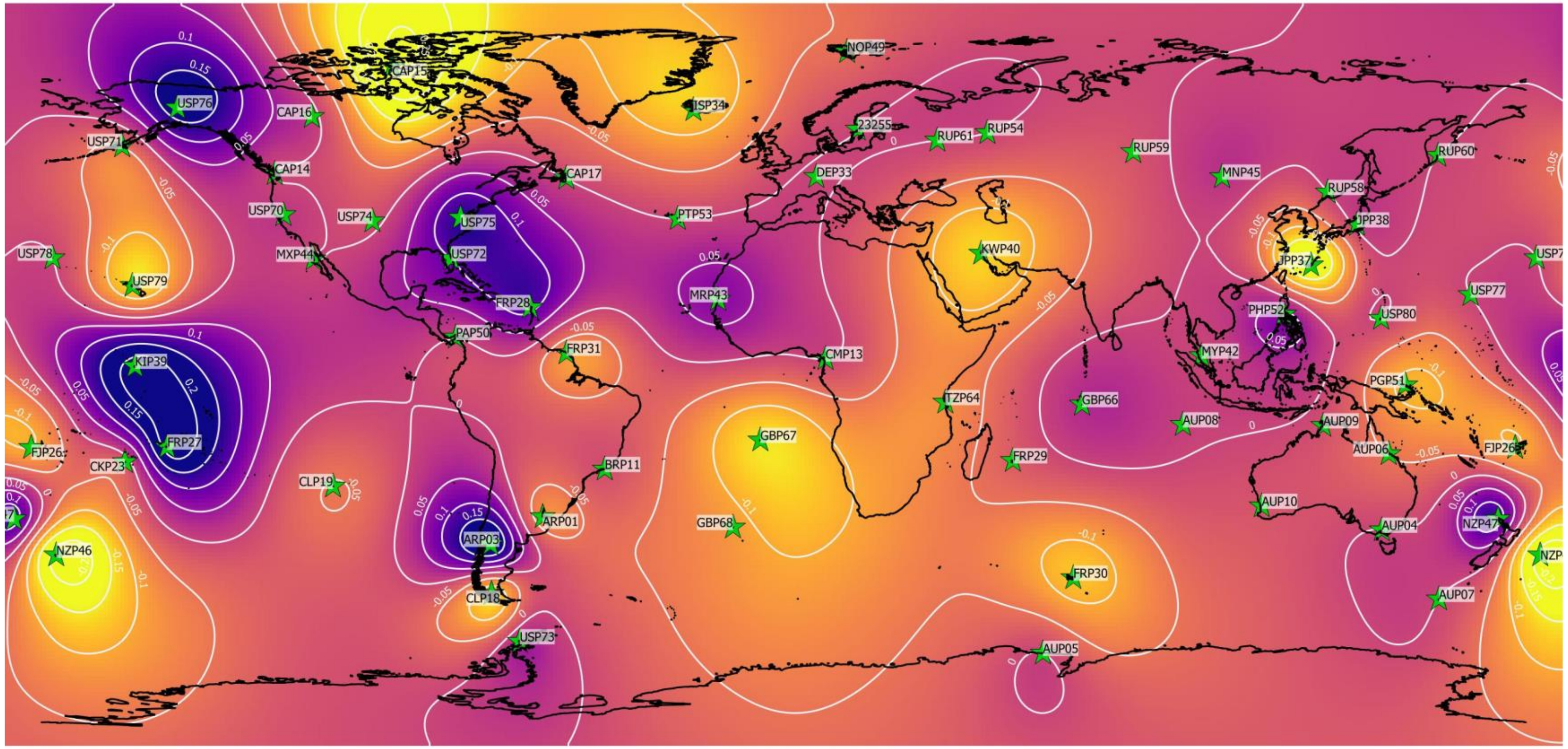

Legend

map

IMS Stations

contour lines

Beryllium-7

-0.1

-0.01

0.01

0.1

0.15

2007

Map executed with QGIS.
QGIS Development Team (2019). QGIS Geographic Information System. Open Source Geospatial Foundation Project. http://qgis.osgeo.org.

^{7}Be normalized trend interpolated into a global map

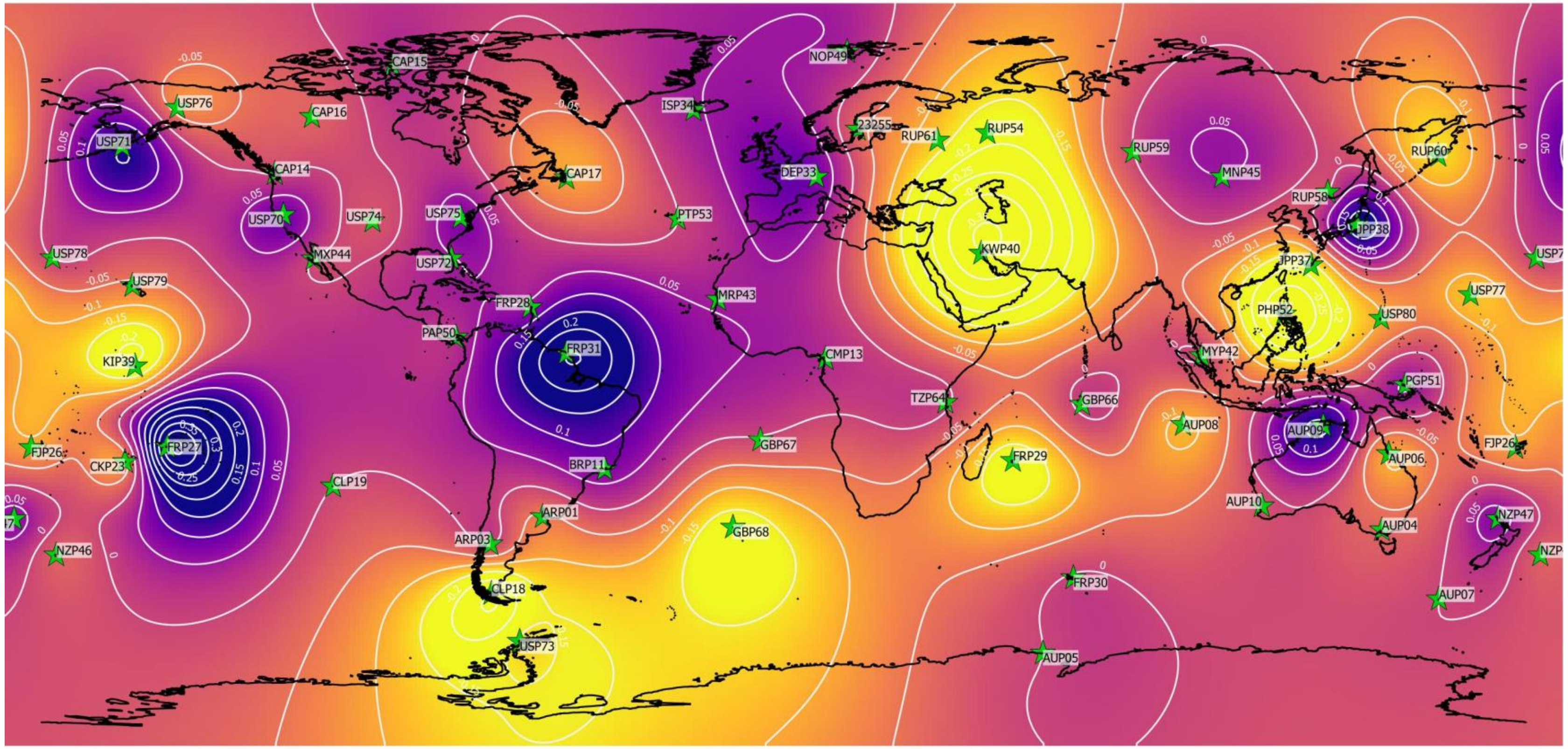

2008

Map executed with QGIS.
QGIS Development Team (2019). QGIS Geographic Information System. Open Source Geospatial Foundation Project. http://qgis.osgeo.org.

^{7}Be normalized trend interpolated into a global map

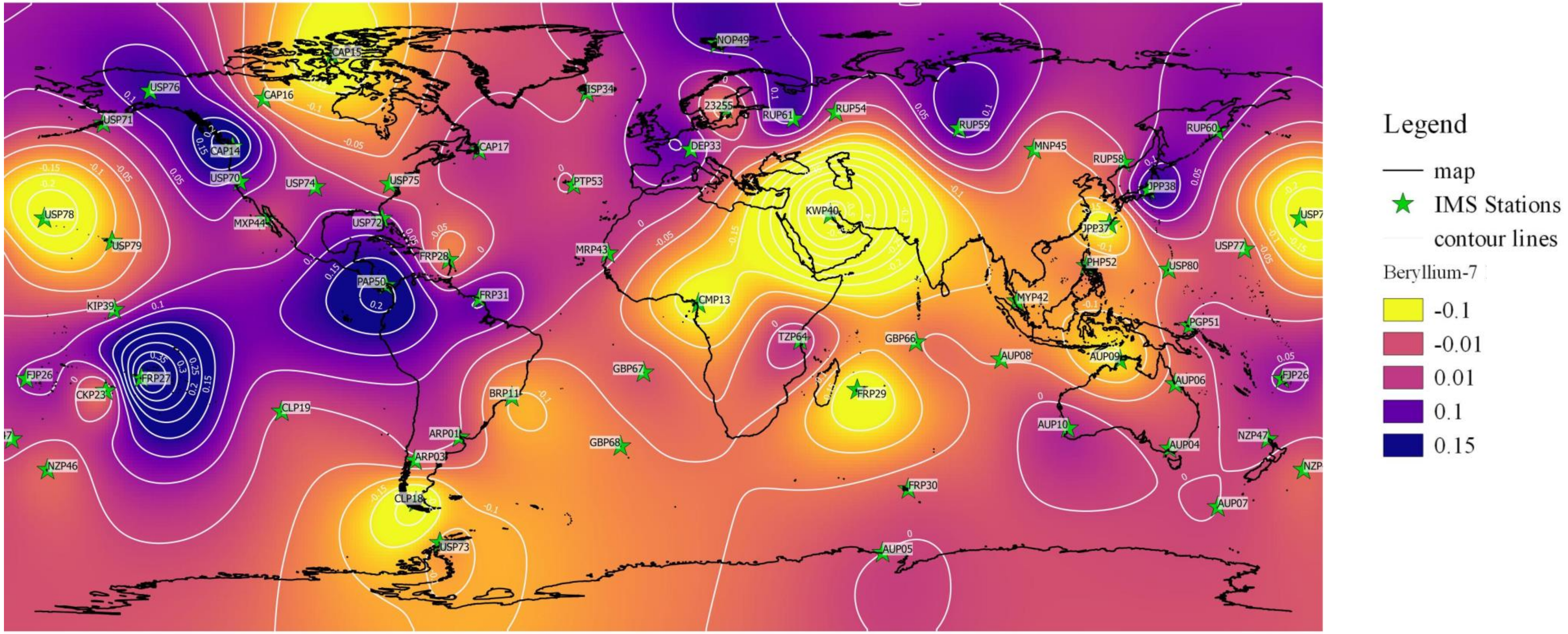

2009

Map executed with QGIS.
QGIS Development Team (2019). QGIS Geographic Information System. Open Source Geospatial Foundation Project. http://qgis.osgeo.org.

^{7}Be normalized trend interpolated into a global map

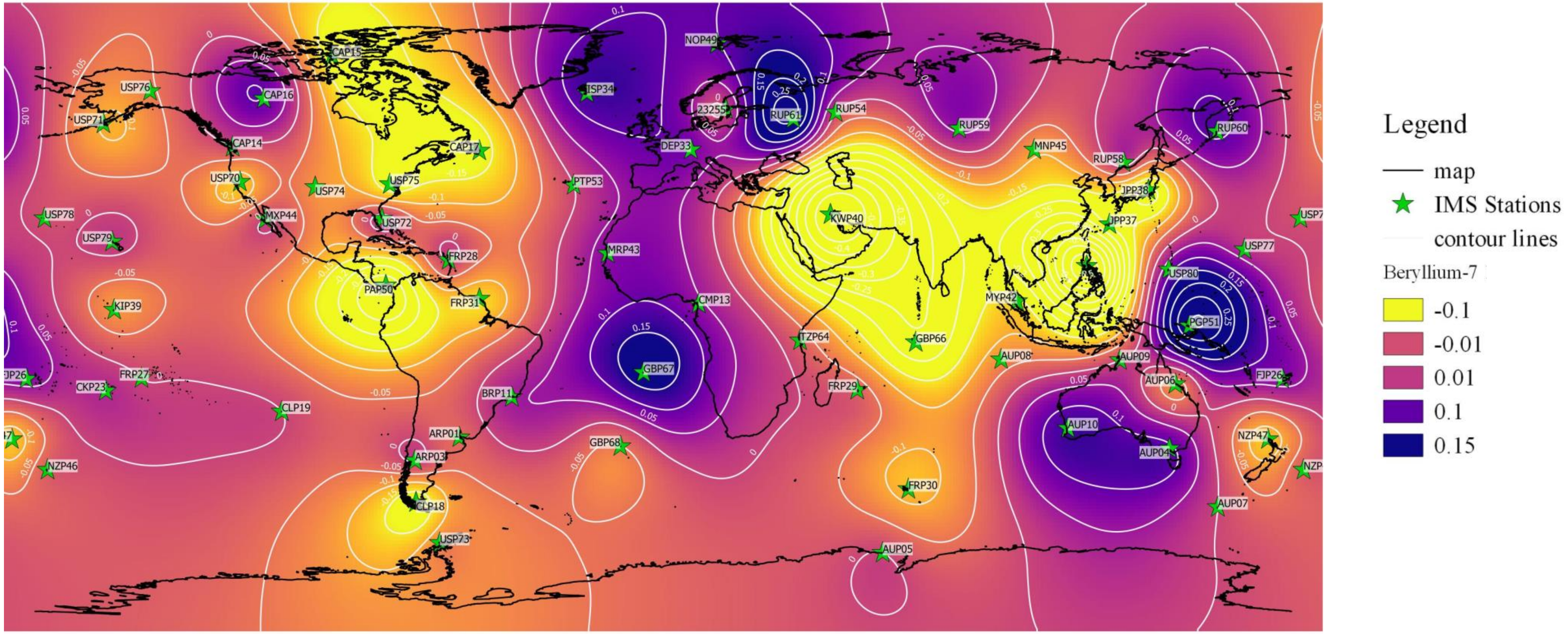

2010

Map executed with QGIS.
QGIS Development Team (2019). QGIS Geographic Information System. Open Source Geospatial Foundation Project. http://qgis.osgeo.org.

^{7}Be normalized trend interpolated into a global map

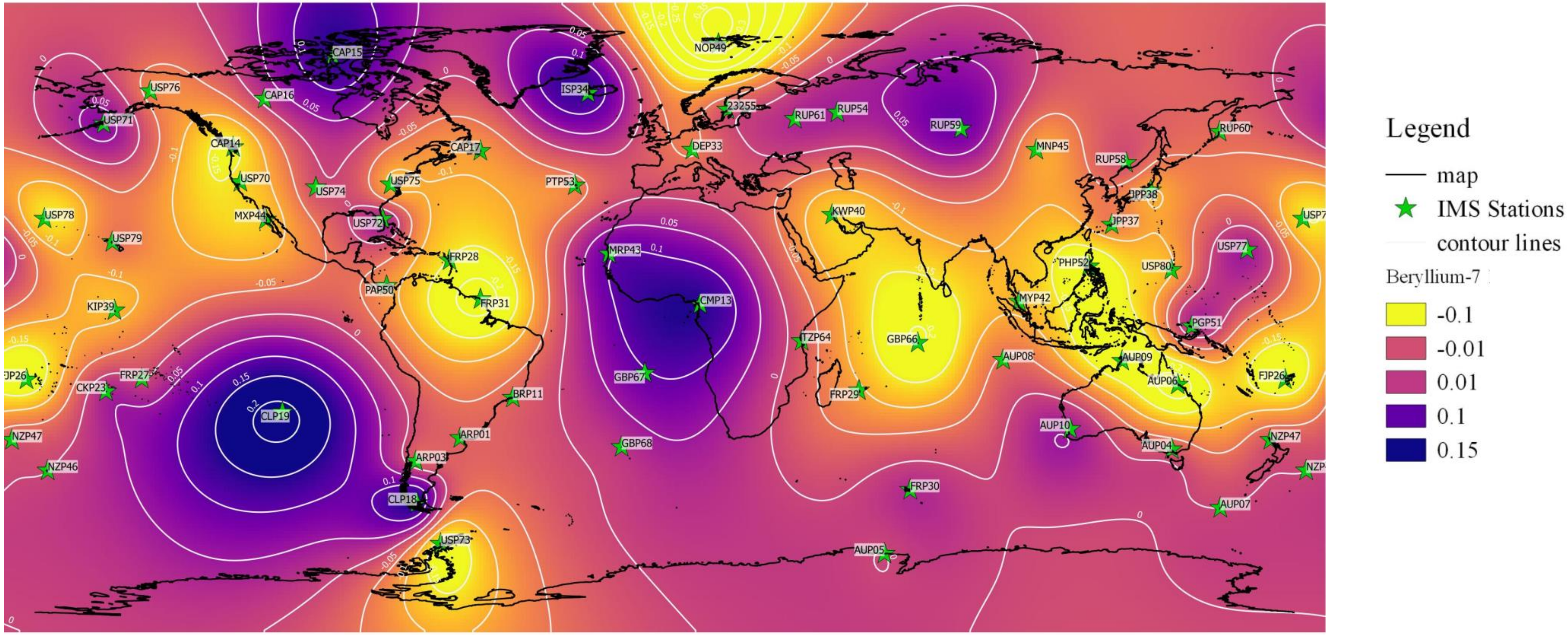

2011

Map executed with QGIS.
QGIS Development Team (2019). QGIS Geographic Information System. Open Source Geospatial Foundation Project. http://qgis.osgeo.org.

^{7}Be normalized trend interpolated into a global map

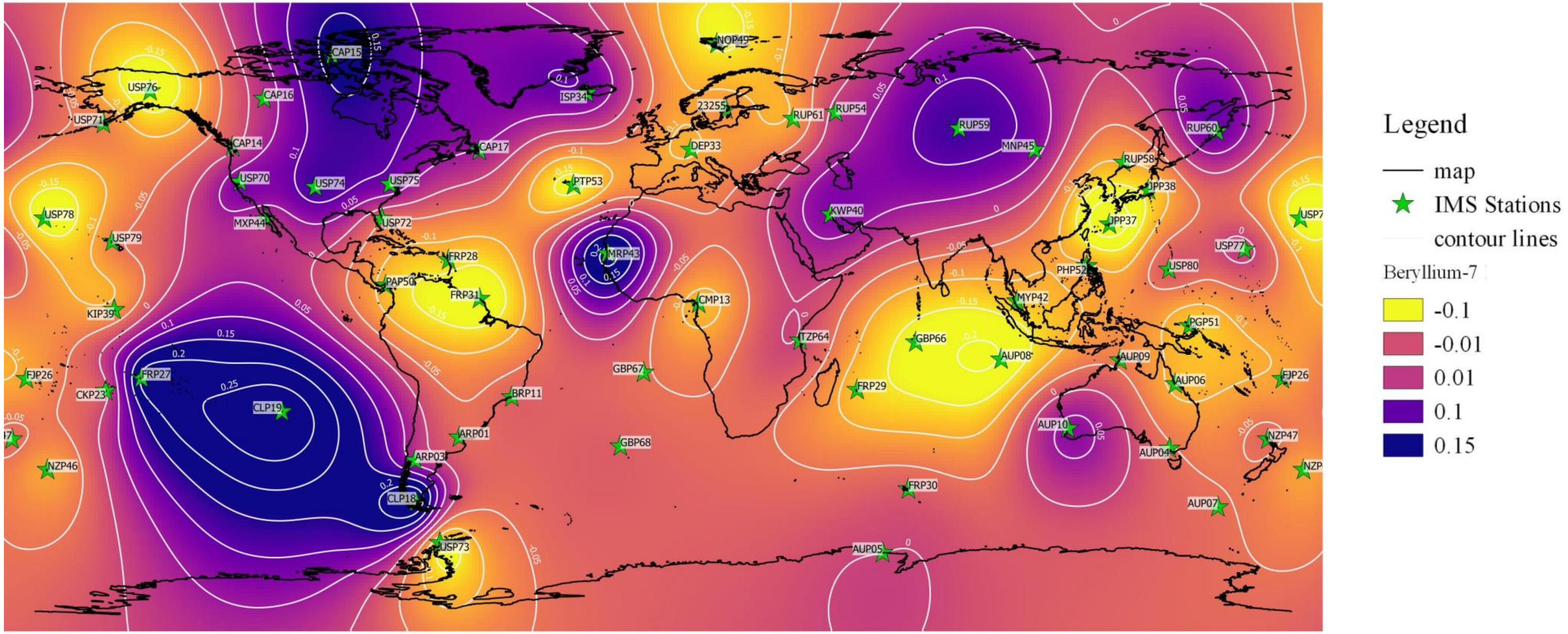

2012

Map executed with QGIS.
QGIS Development Team (2019). QGIS Geographic Information System. Open Source Geospatial Foundation Project. http://qgis.osgeo.org.

^{7}Be normalized trend interpolated into a global map

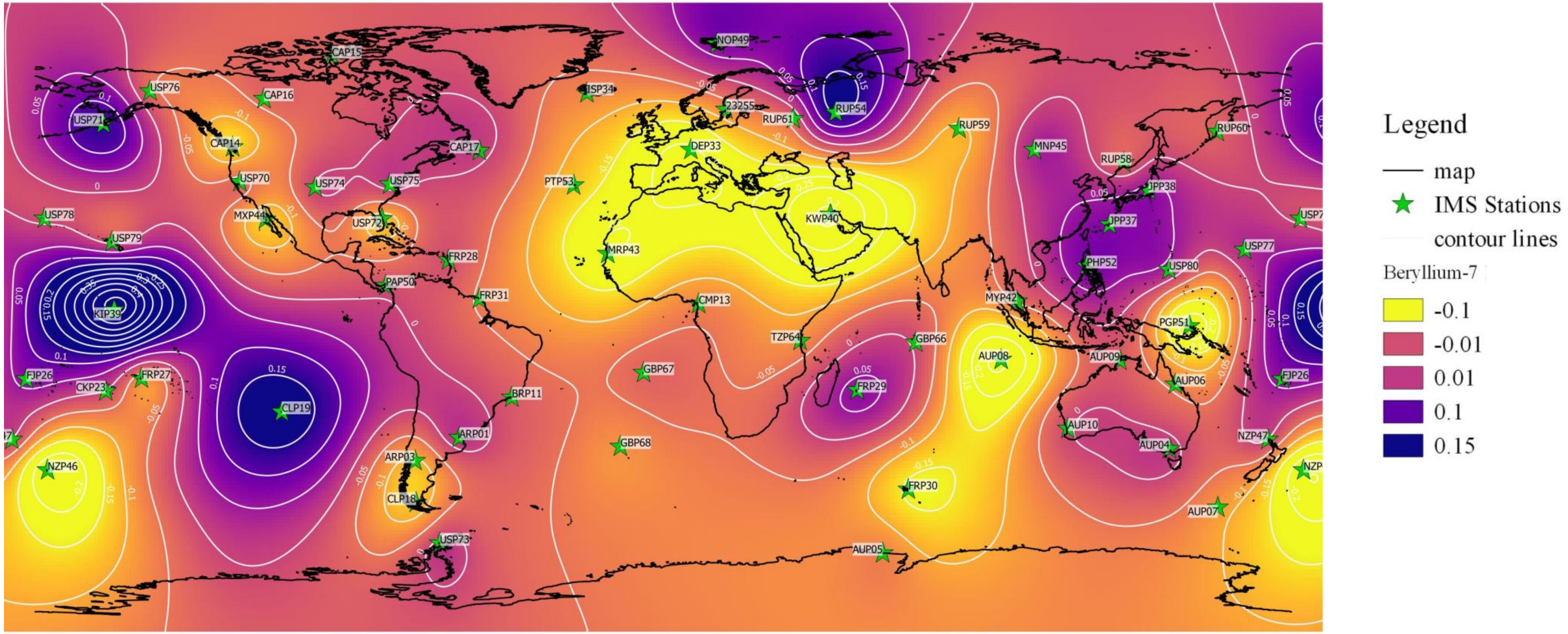

2013

Map executed with QGIS.
QGIS Development Team (2019). QGIS Geographic Information System. Open Source Geospatial Foundation Project. http://qgis.osgeo.org.

^{7}Be normalized trend interpolated into a global map

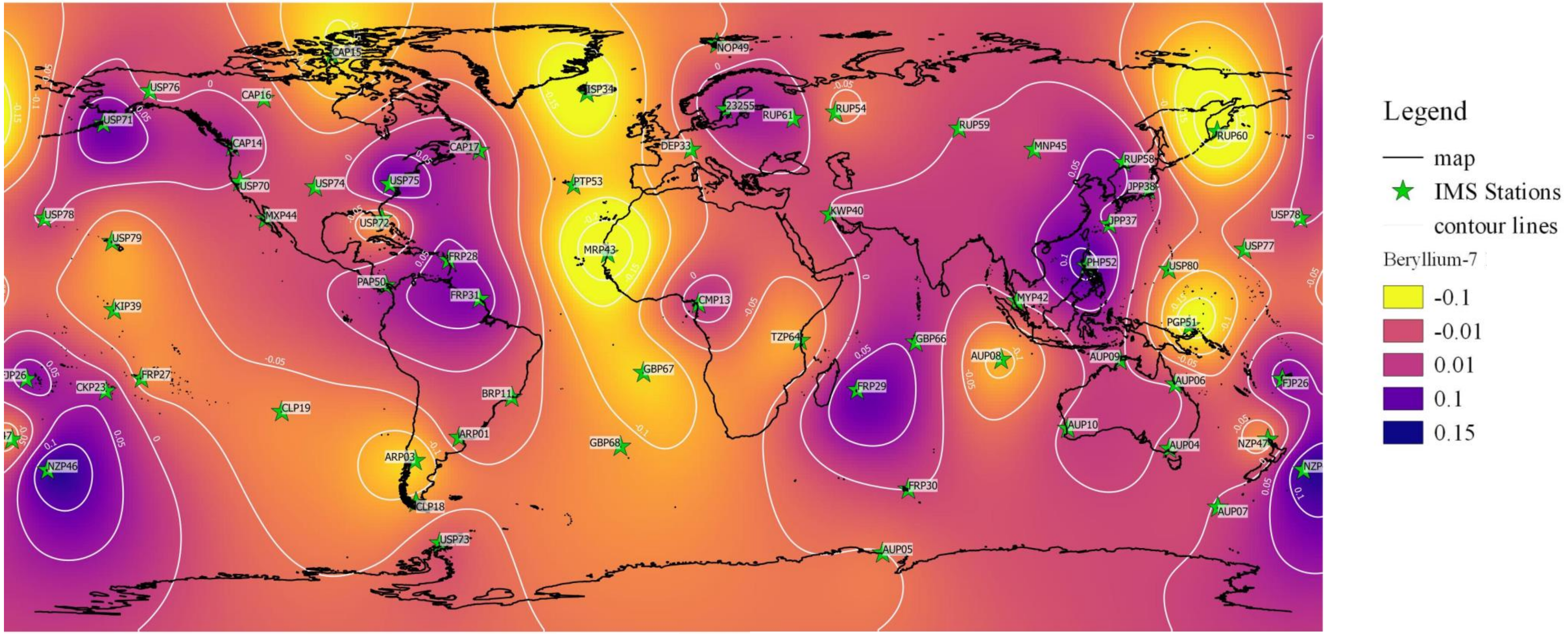

2014

Map executed with QGIS.
QGIS Development Team (2019). QGIS Geographic Information System. Open Source Geospatial Foundation Project. http://qgis.osgeo.org.

^{7}Be normalized trend interpolated into a global map

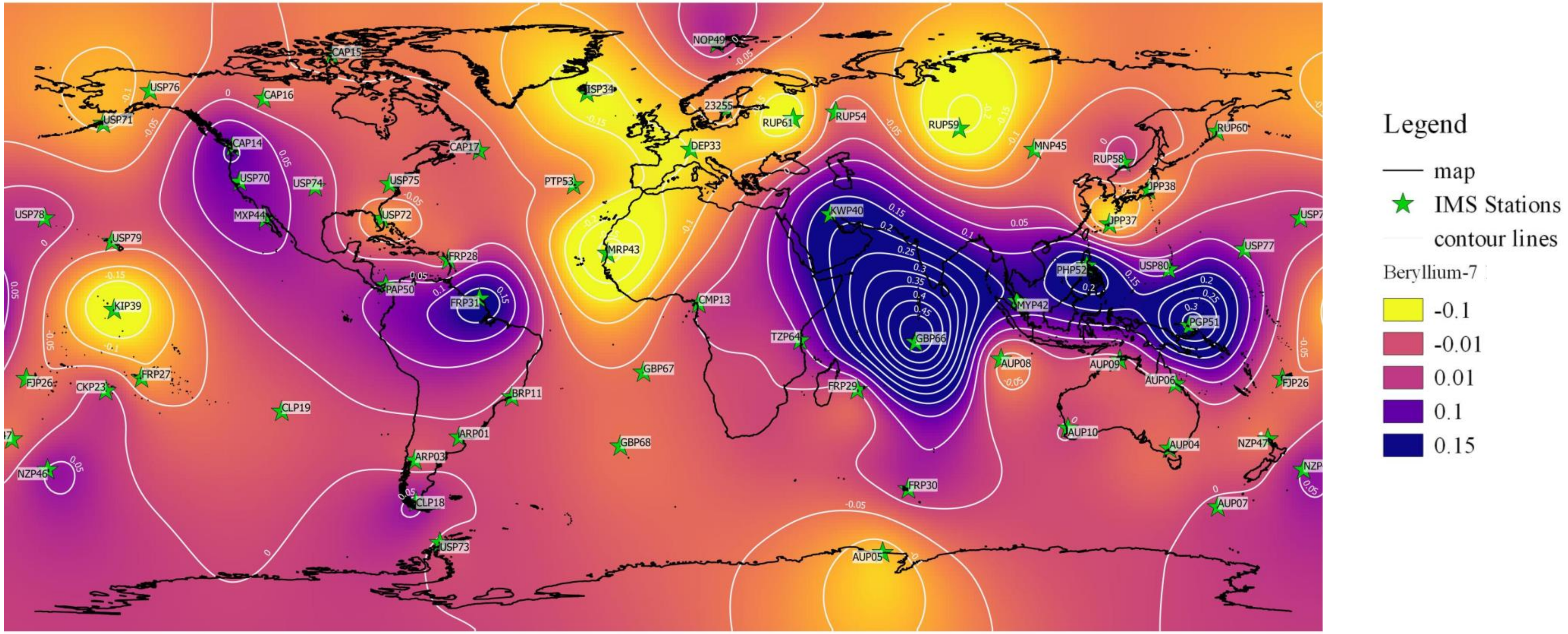

2015

Map executed with QGIS.
QGIS Development Team (2019). QGIS Geographic Information System. Open Source Geospatial Foundation Project. http://qgis.osgeo.org.

^{7}Be normalized trend interpolated into a global map

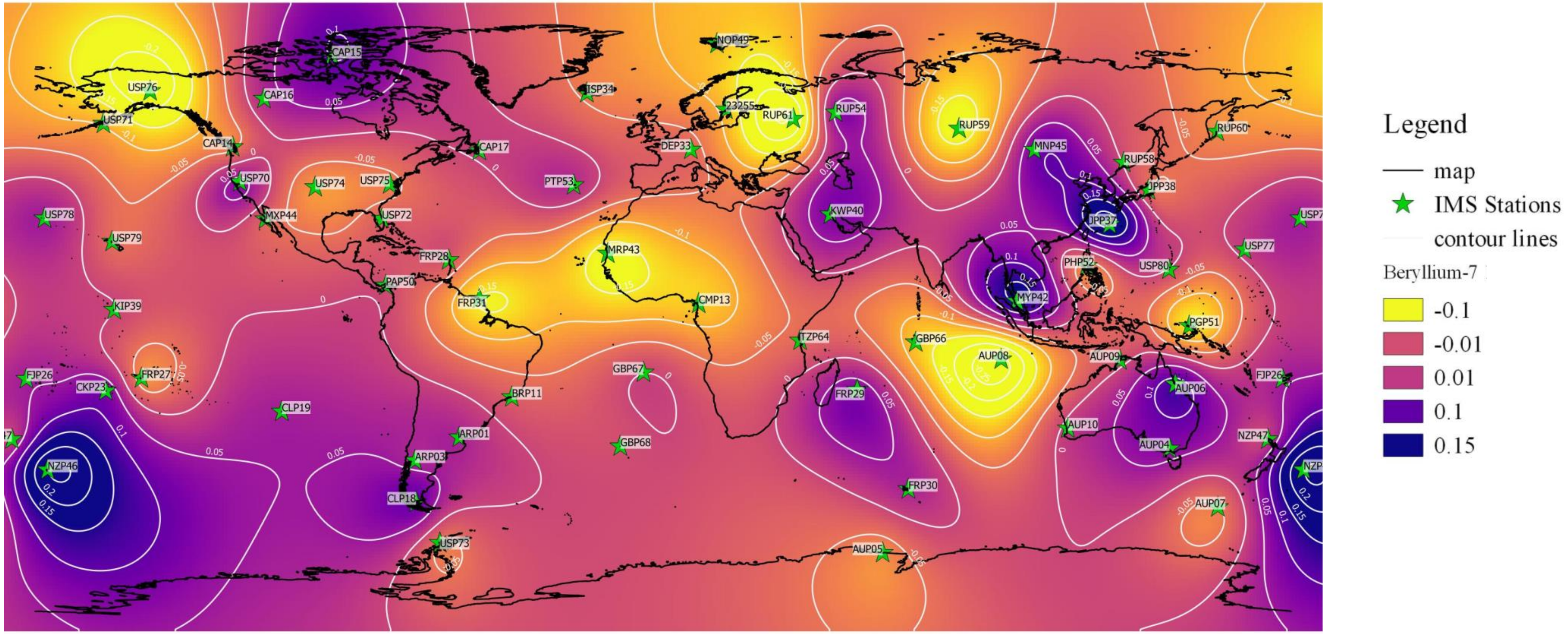

2016

Map executed with QGIS.
QGIS Development Team (2019). QGIS Geographic Information System. Open Source Geospatial Foundation Project. http://qgis.osgeo.org.

^{7}Be normalized trend interpolated into a global map

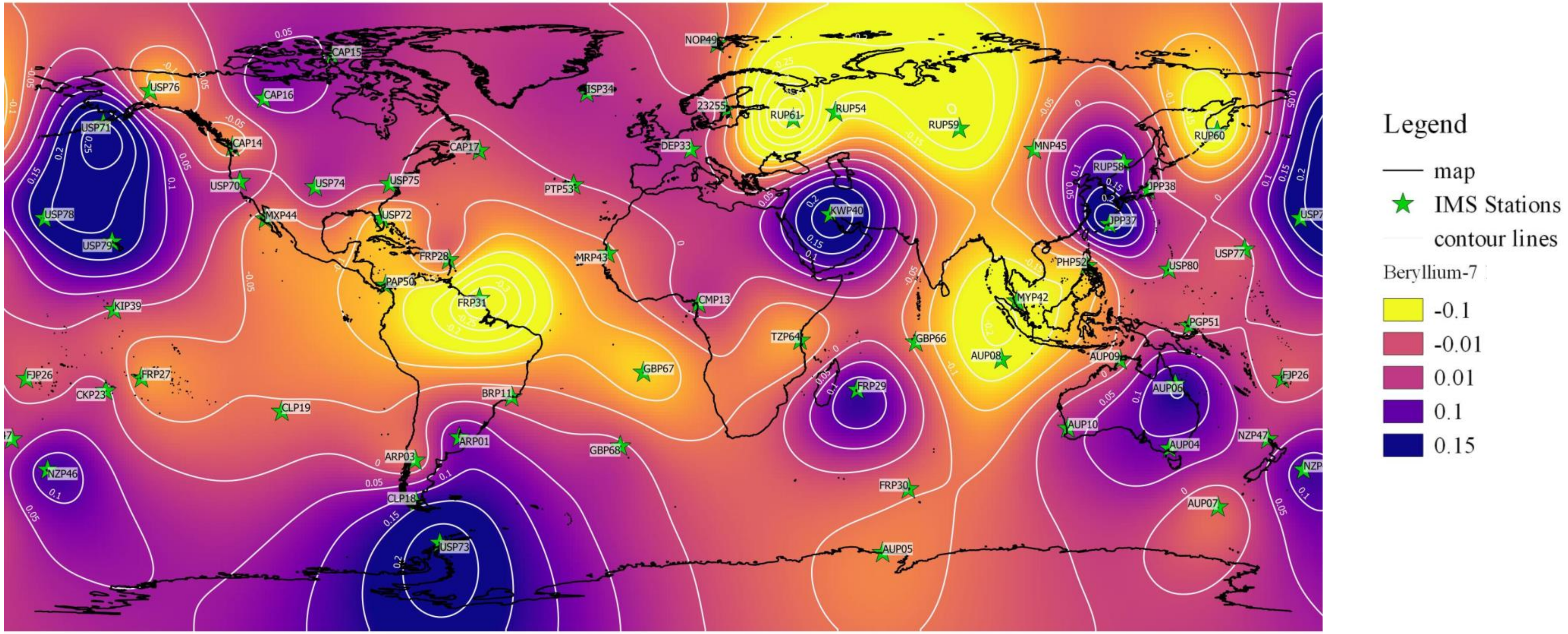

2017

Map executed with QGIS.
QGIS Development Team (2019). QGIS Geographic Information System. Open Source Geospatial Foundation Project. http://qgis.osgeo.org.

^{7}Be normalized trend interpolated into a global map

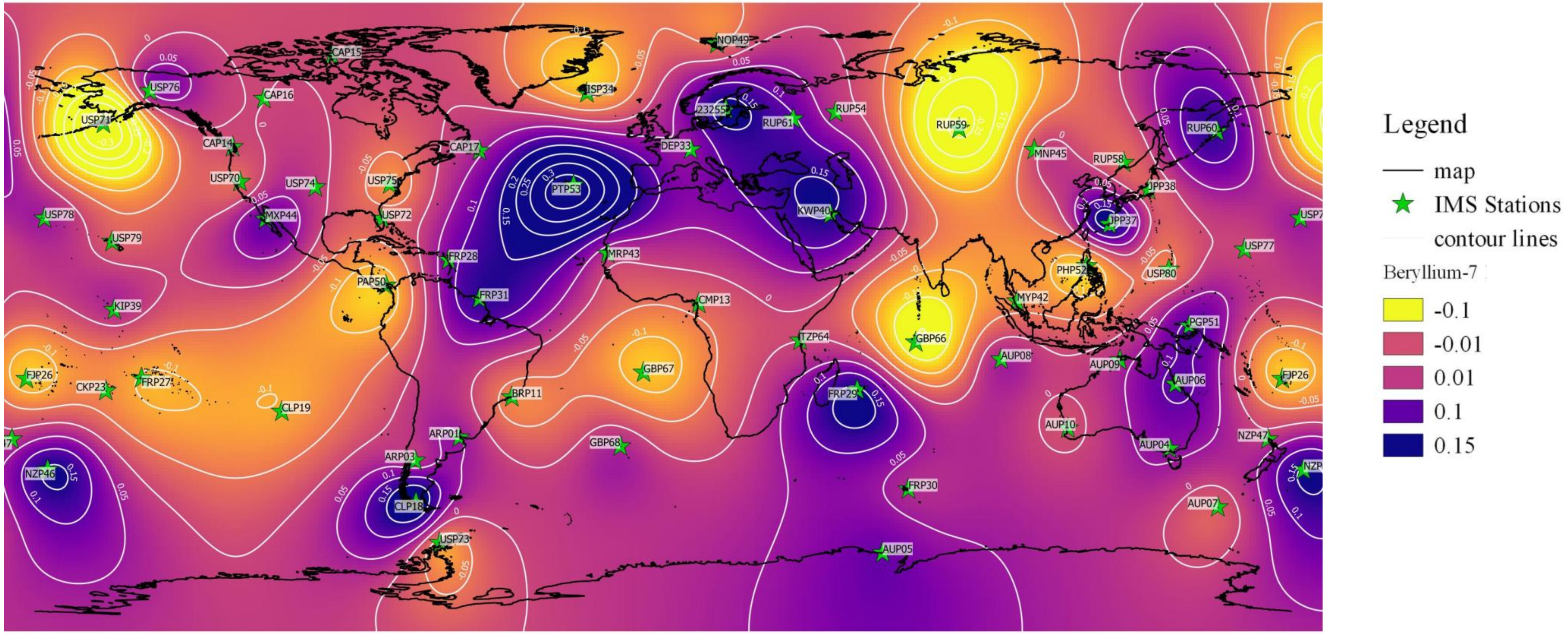

2018

Map executed with QGIS.
QGIS Development Team (2019). QGIS Geographic Information System. Open Source Geospatial Foundation Project. http://qgis.osgeo.org.

^{7}Be normalized trend interpolated into a global map

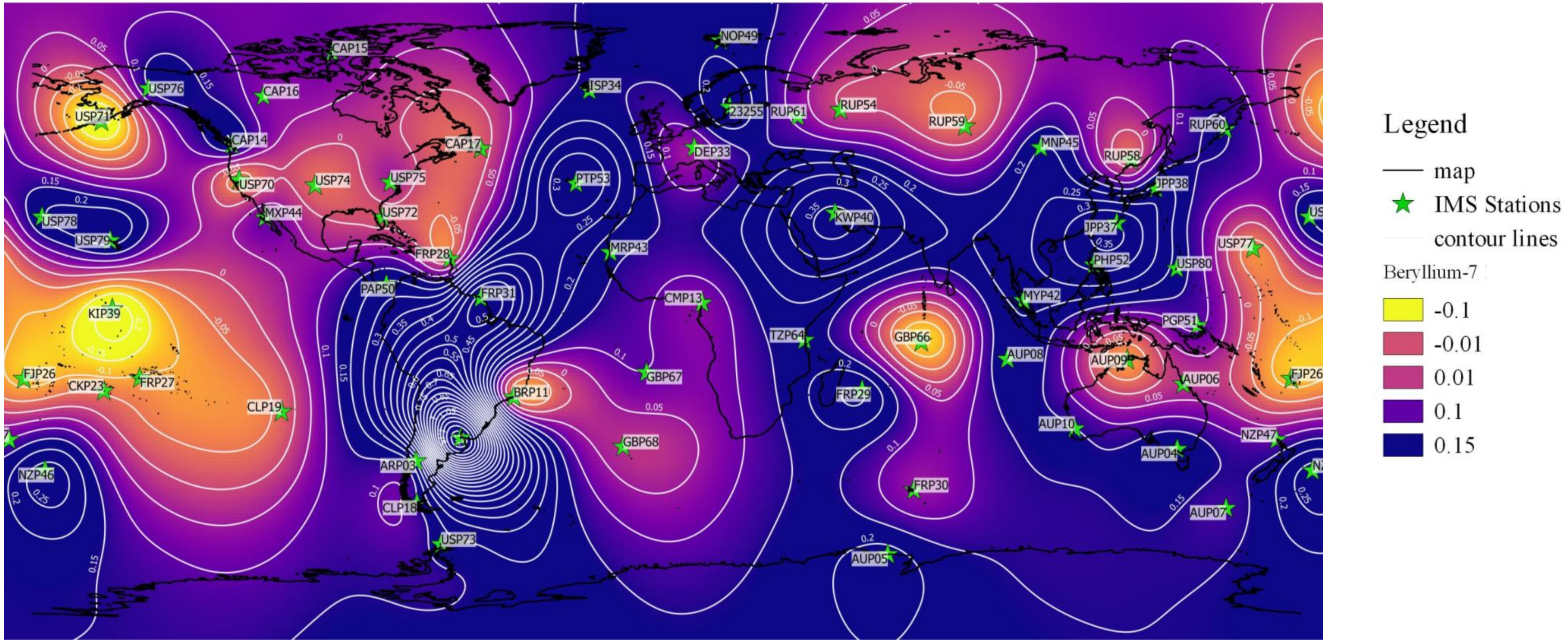

2019

Map executed with QGIS.
QGIS Development Team (2019). QGIS Geographic Information System. Open Source Geospatial Foundation Project. http://qgis.osgeo.org.

^7Be normalized trend interpolated into a global map

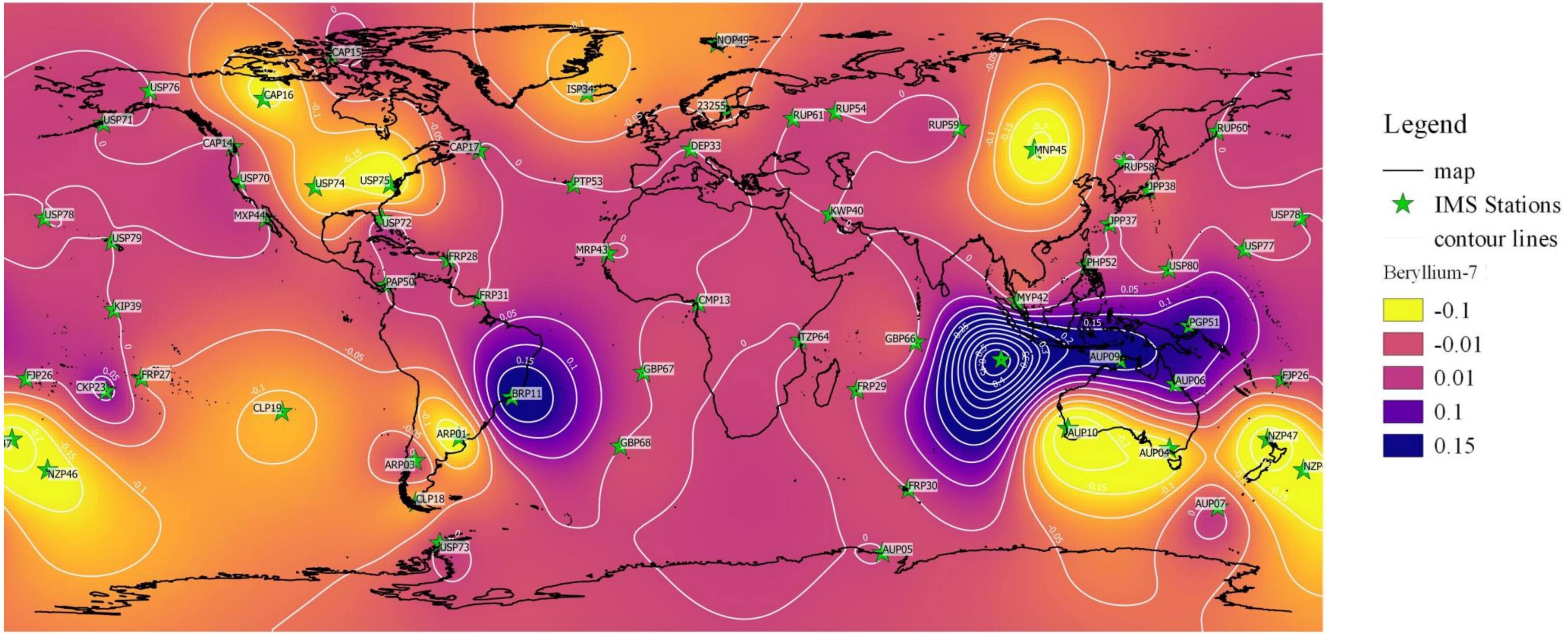

2003

Map executed with QGIS.
QGIS Development Team (2019). QGIS Geographic Information System. Open Source Geospatial Foundation Project. http://qgis.osgeo.org.

^{7}Be normalized trend interpolated into a global map

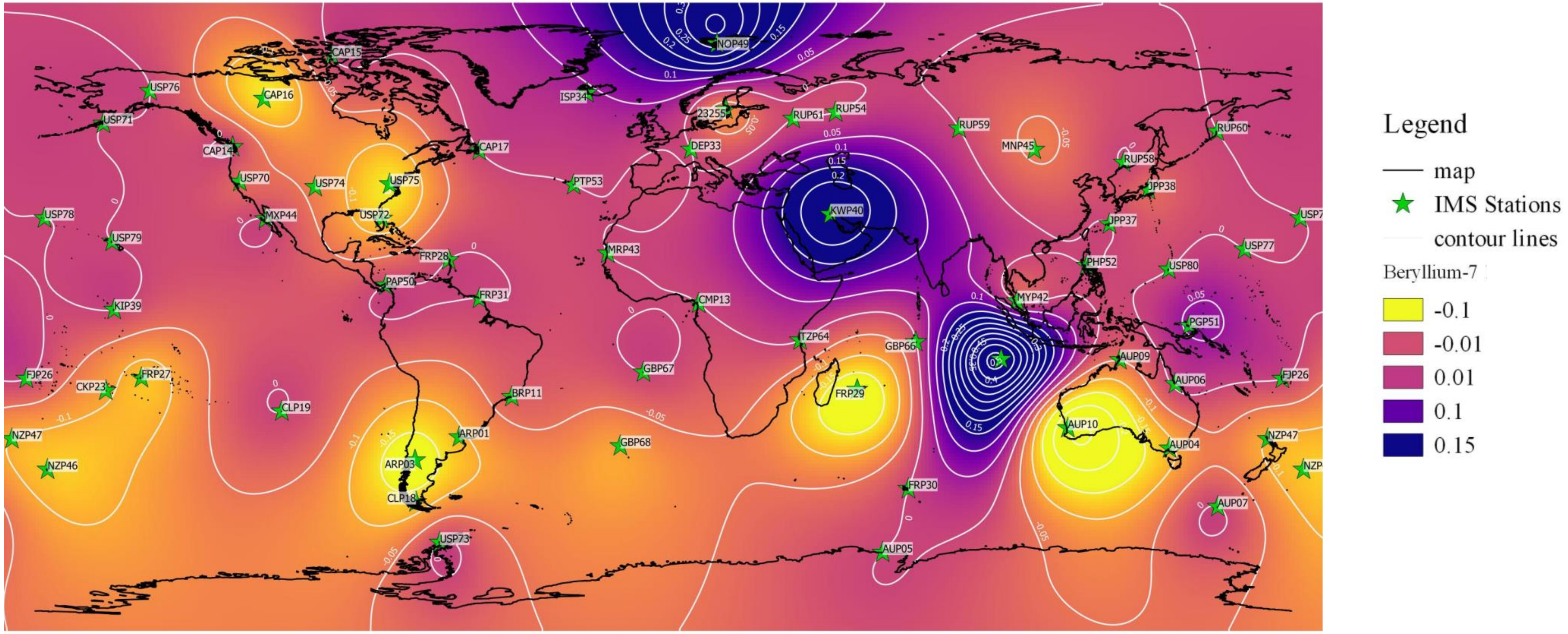

2004

Map executed with QGIS.
QGIS Development Team (2019). QGIS Geographic Information System. Open Source Geospatial Foundation Project. http://qgis.osgeo.org.

^{7}Be normalized trend interpolated into a global map

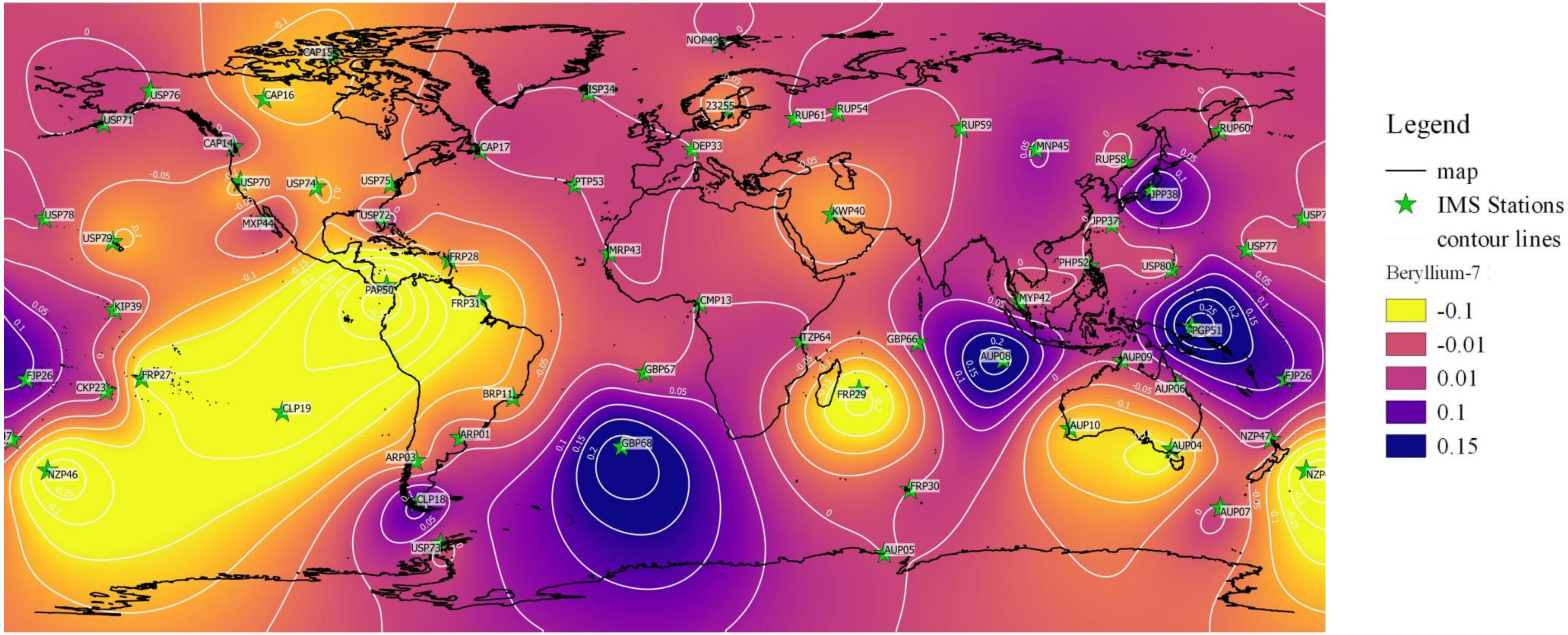

2005

Map executed with QGIS.
QGIS Development Team (2019). QGIS Geographic Information System. Open Source Geospatial Foundation Project. http://qgis.osgeo.org.

^{7}Be normalized trend interpolated into a global map

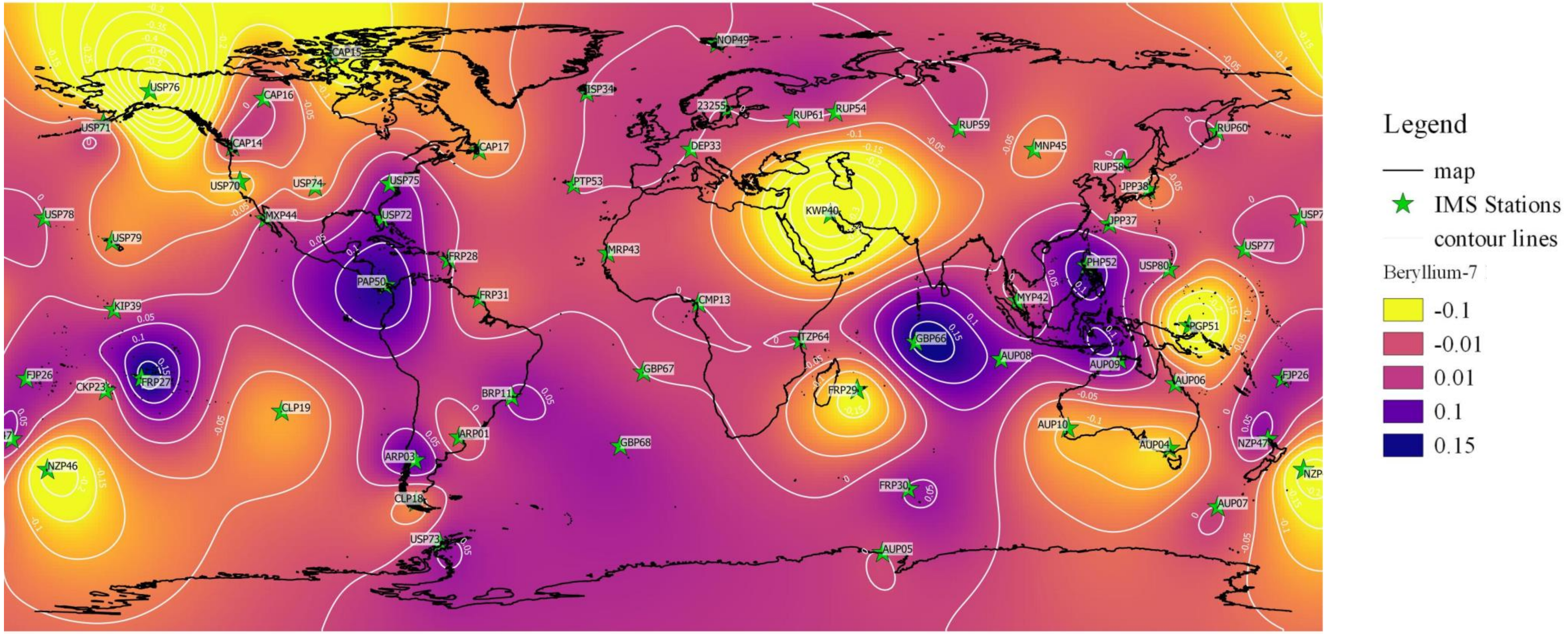

2006

Map executed with QGIS.
QGIS Development Team (2019). QGIS Geographic Information System. Open Source Geospatial Foundation Project. http://qgis.osgeo.org.

^{7}Be normalized trend interpolated into a global map

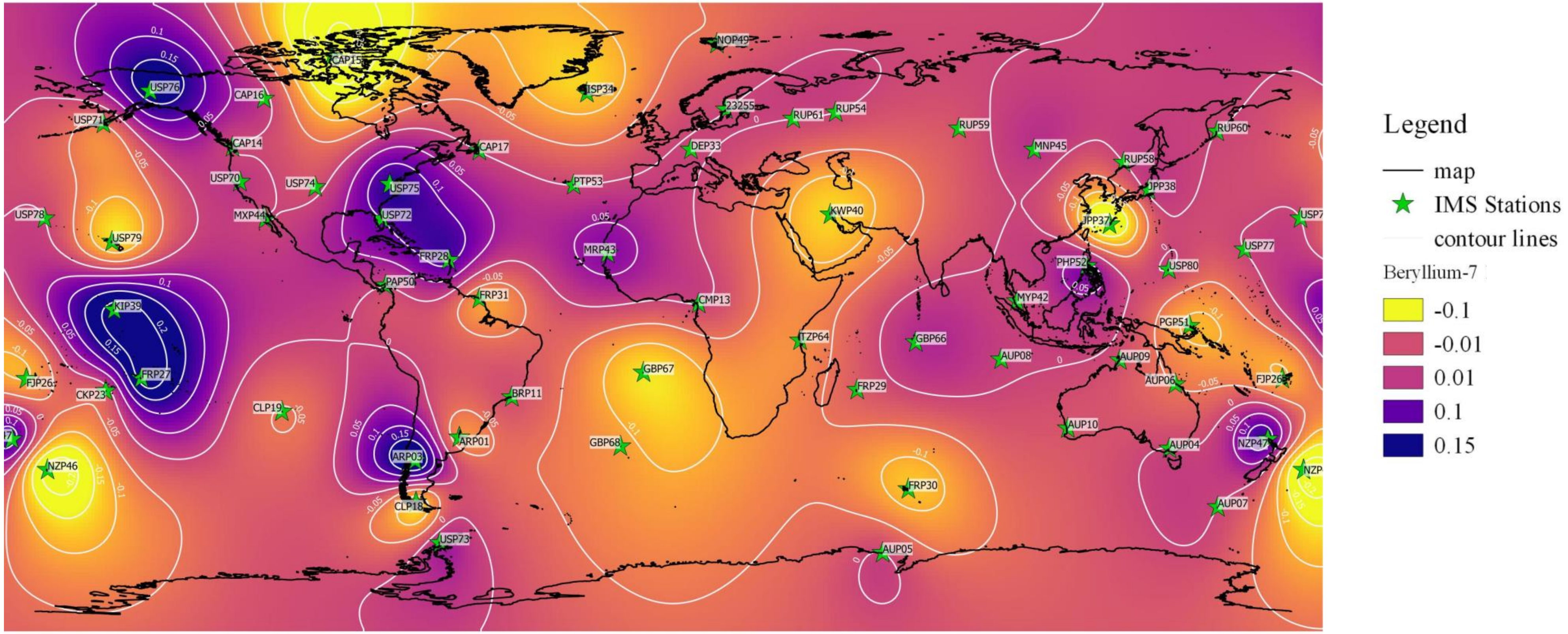

2007

Map executed with QGIS.
QGIS Development Team (2019). QGIS Geographic Information System. Open Source Geospatial Foundation Project. http://qgis.osgeo.org.

^{7}Be normalized trend interpolated into a global map

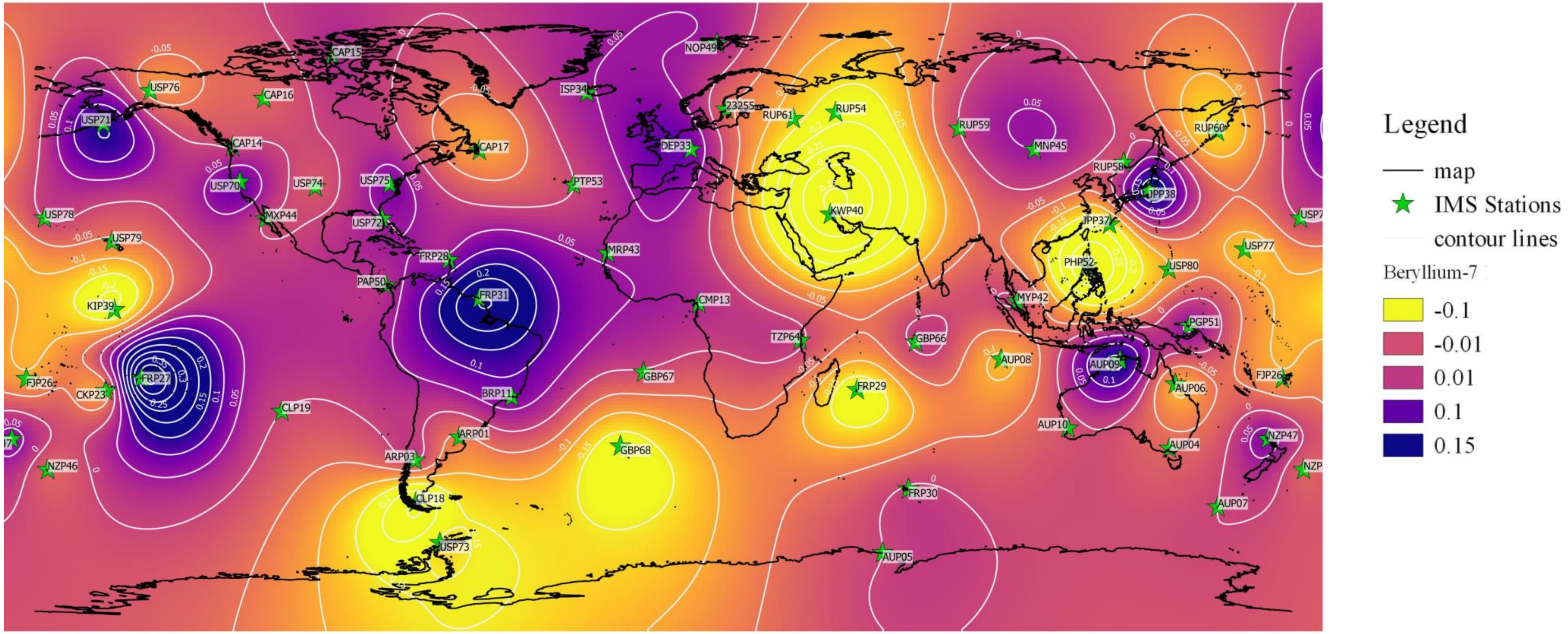

2008

Map executed with QGIS.
QGIS Development Team (2019). QGIS Geographic Information System. Open Source Geospatial Foundation Project. http://qgis.osgeo.org.

^{7}Be normalized trend interpolated into a global map

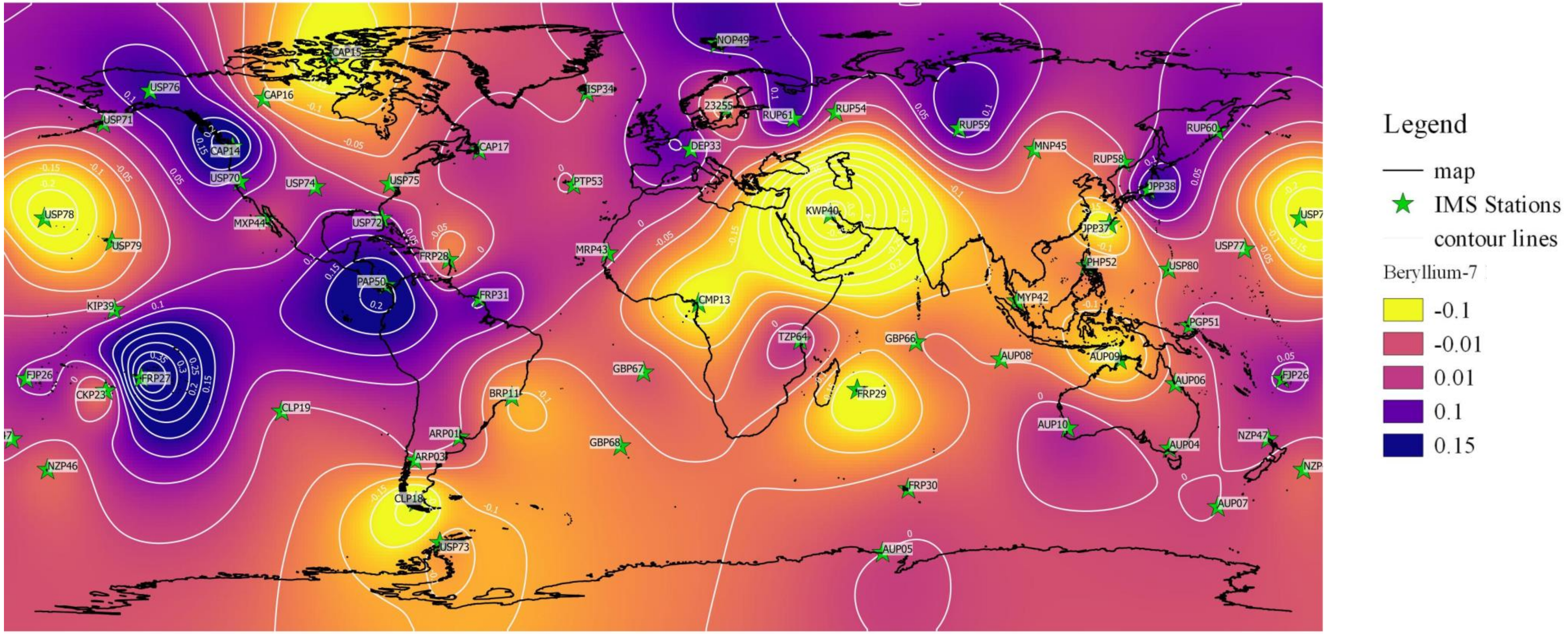

2009

Map executed with QGIS.
QGIS Development Team (2019). QGIS Geographic Information System. Open Source Geospatial Foundation Project. http://qgis.osgeo.org.

^{7}Be normalized trend interpolated into a global map

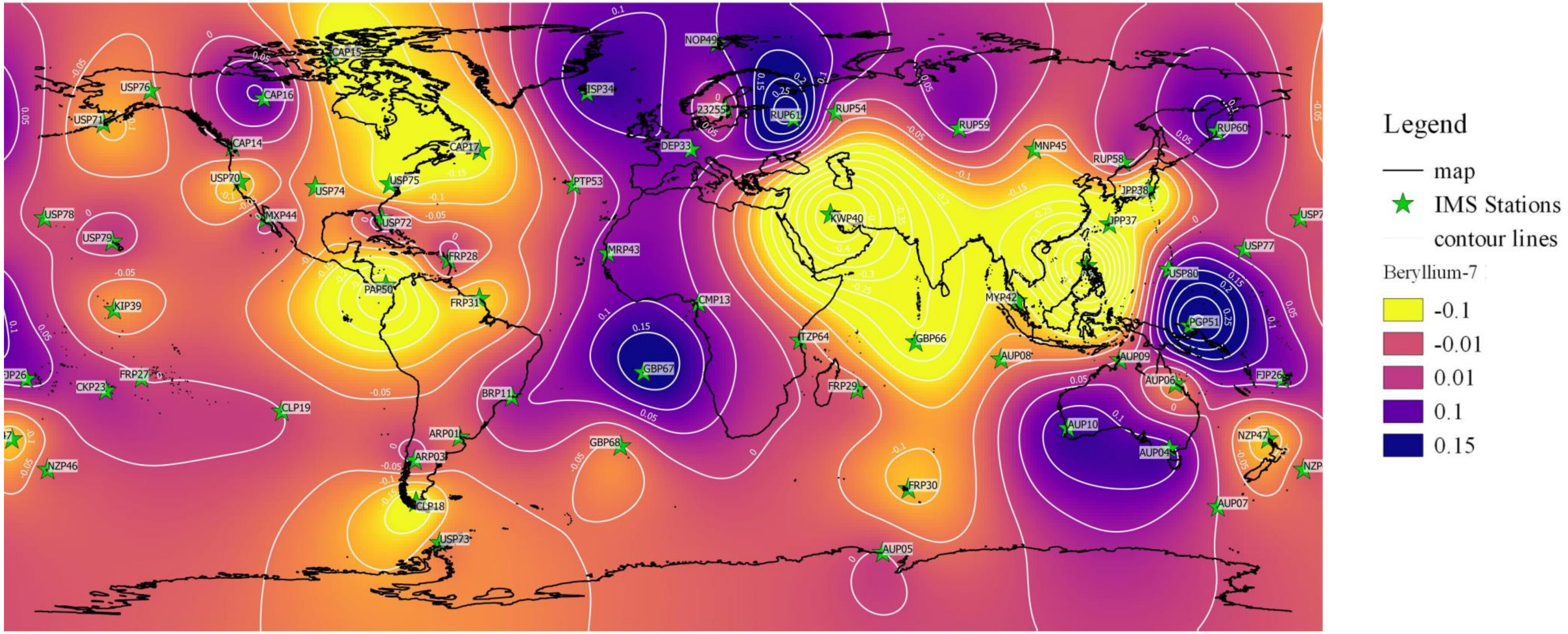

2010

Map executed with QGIS.
QGIS Development Team (2019). QGIS Geographic Information System. Open Source Geospatial Foundation Project. http://qgis.osgeo.org.

^{7}Be normalized trend interpolated into a global map

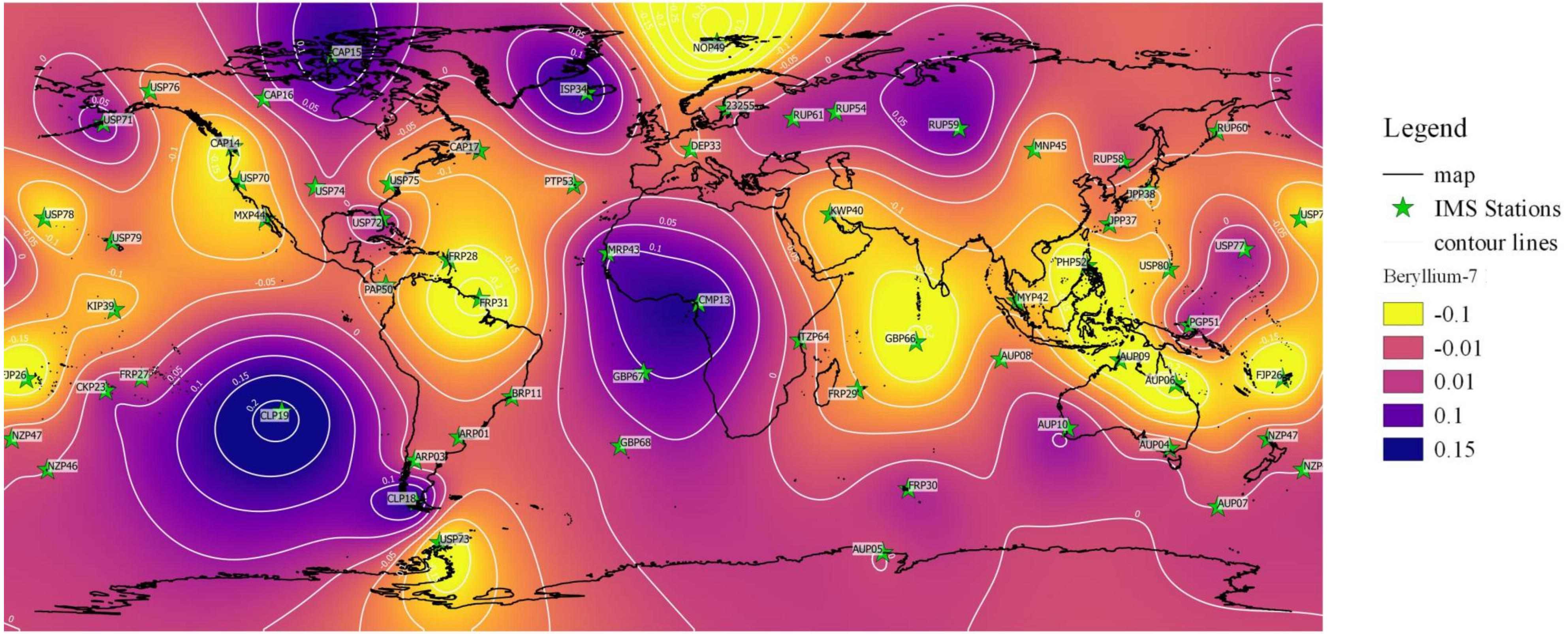

2011

Map executed with QGIS.
QGIS Development Team (2019). QGIS Geographic Information System. Open Source Geospatial Foundation Project. http://qgis.osgeo.org.

^{7}Be normalized trend interpolated into a global map

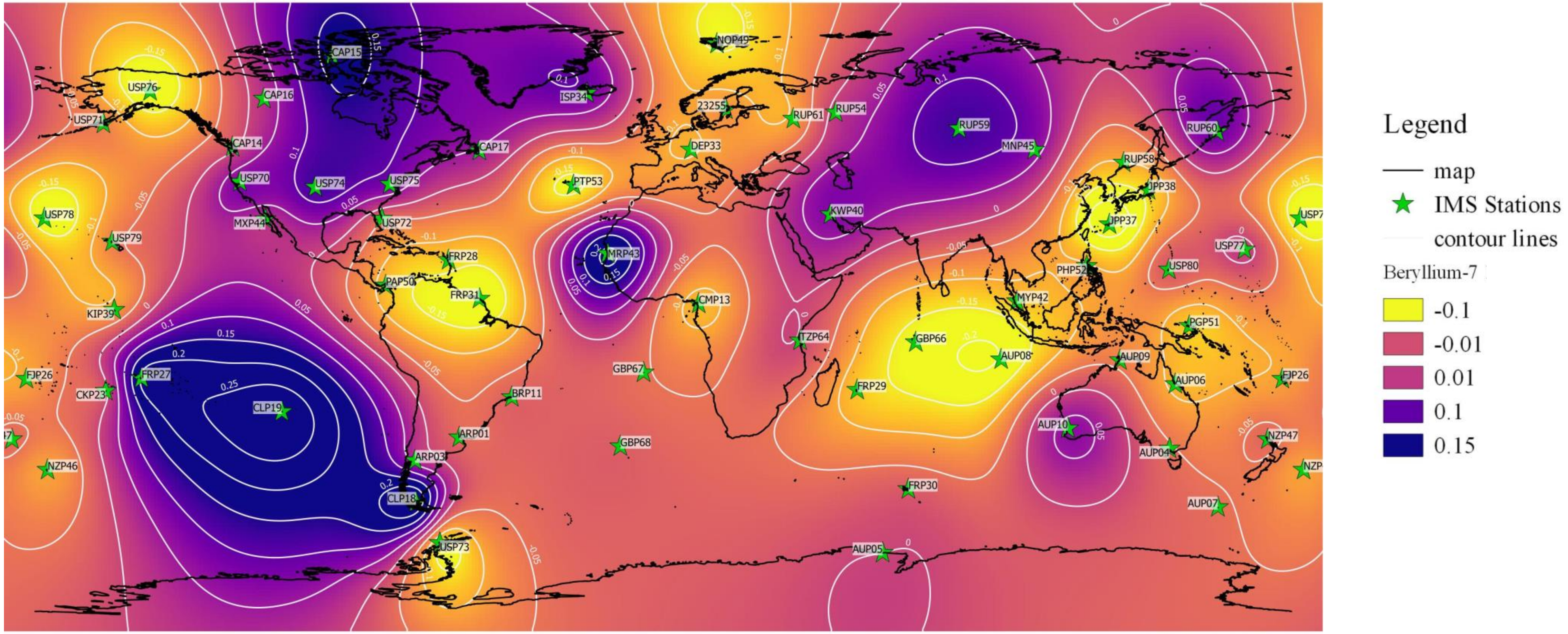

2012

Map executed with QGIS.
QGIS Development Team (2019). QGIS Geographic Information System. Open Source Geospatial Foundation Project. http://qgis.osgeo.org.

^{7}Be normalized trend interpolated into a global map

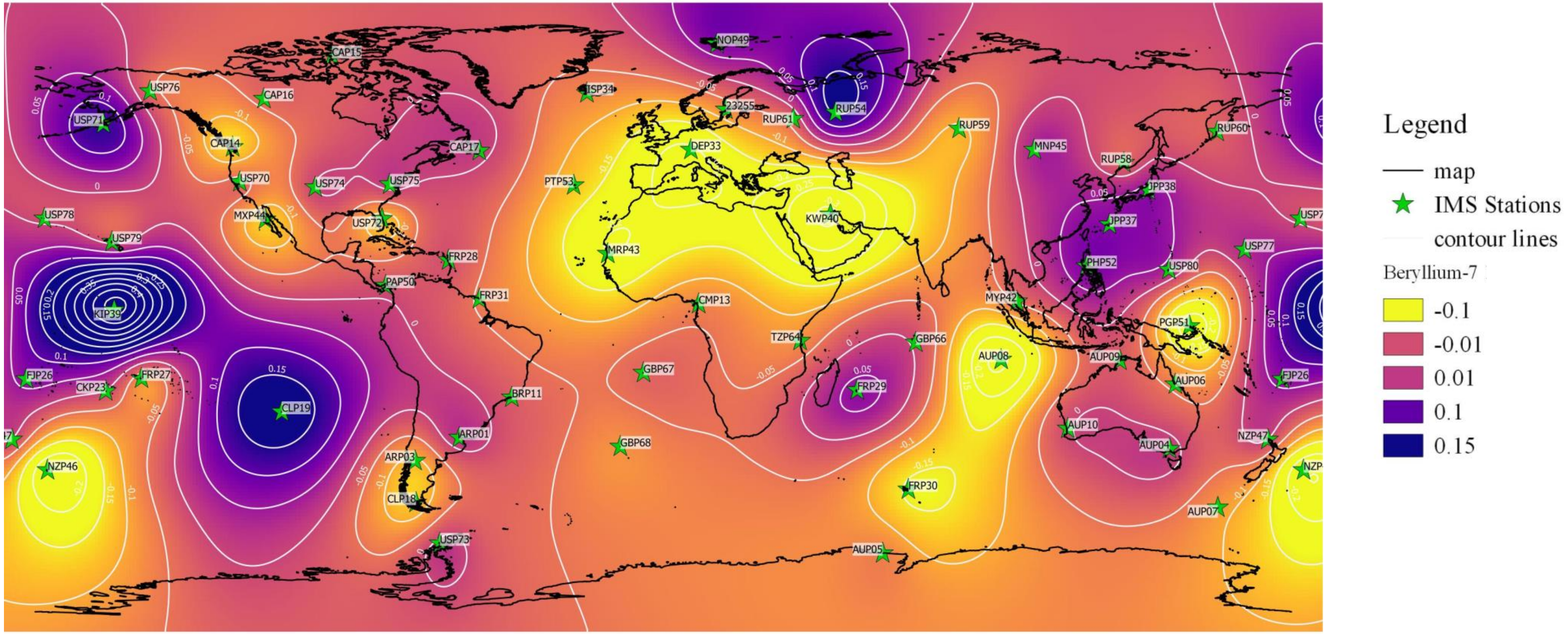

2013

Map executed with QGIS.
QGIS Development Team (2019). QGIS Geographic Information System. Open Source Geospatial Foundation Project. http://qgis.osgeo.org.

^{7}Be normalized trend interpolated into a global map

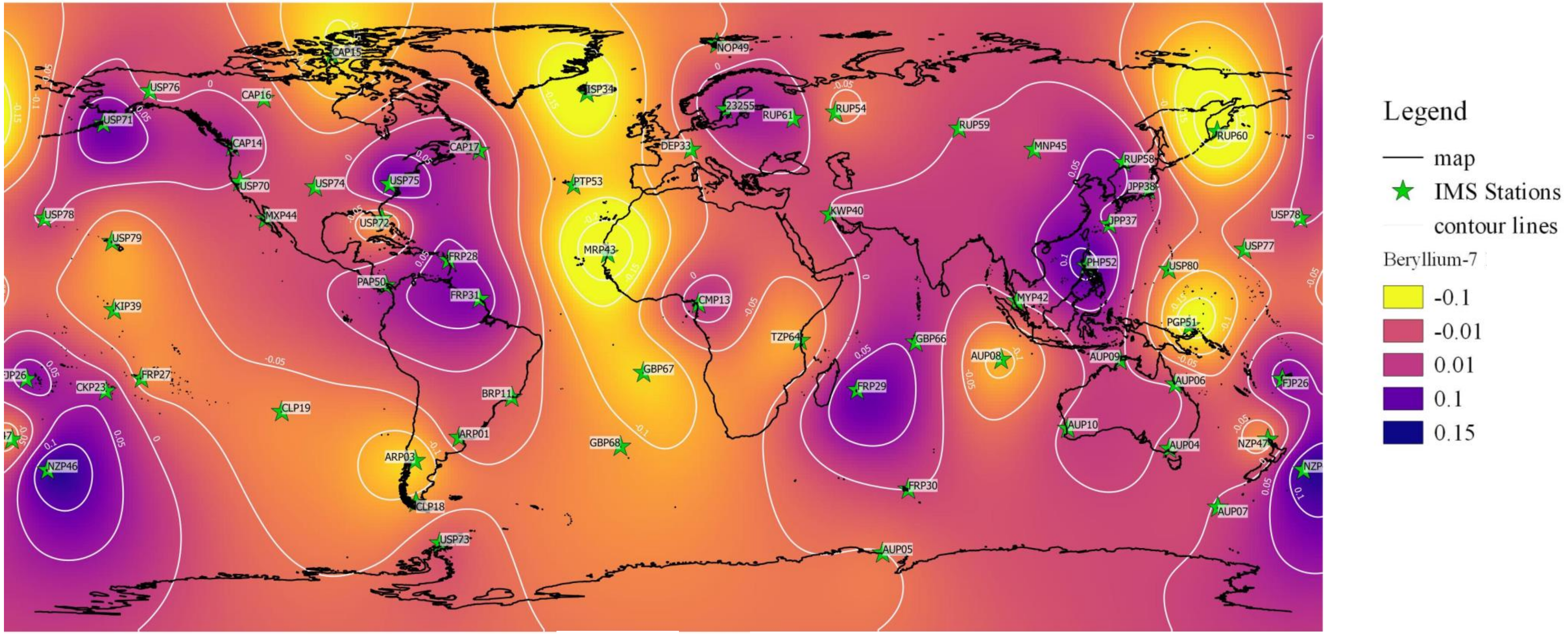

2014

Map executed with QGIS.
QGIS Development Team (2019). QGIS Geographic Information System. Open Source Geospatial Foundation Project. http://qgis.osgeo.org.

^{7}Be normalized trend interpolated into a global map

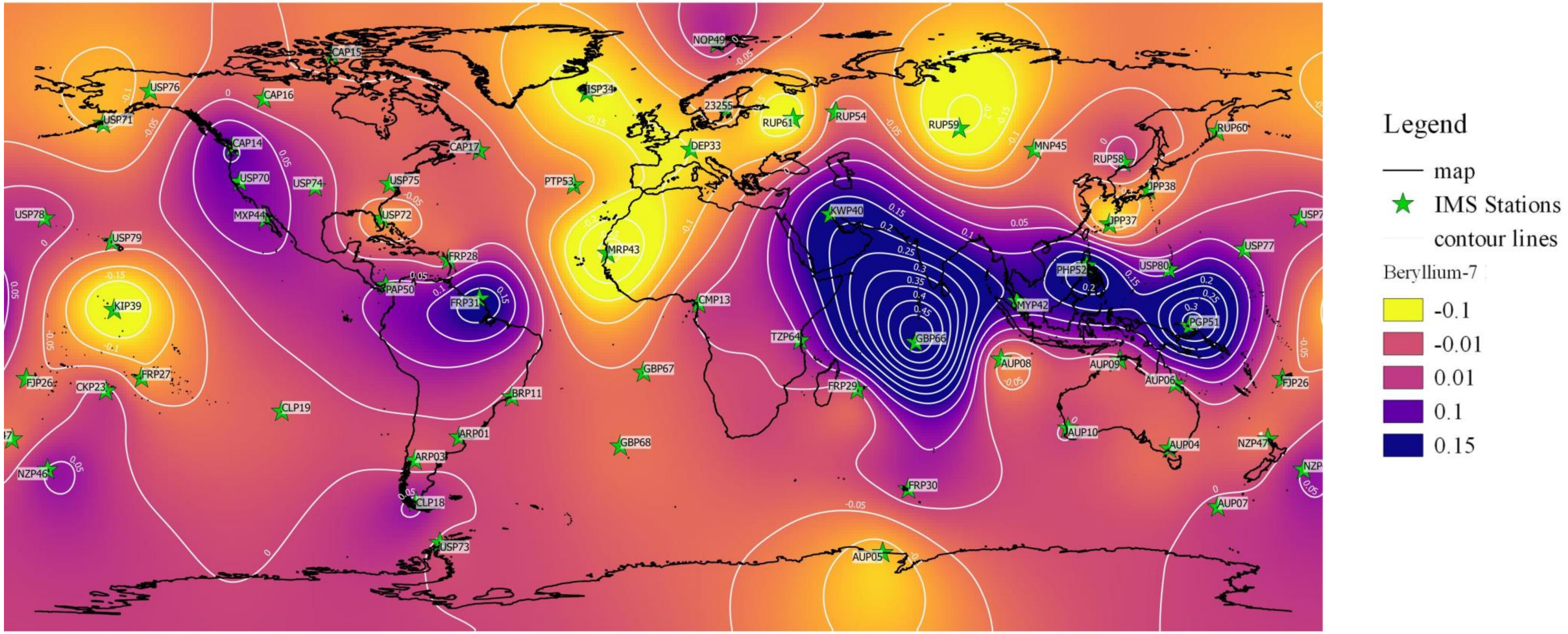

2015

Map executed with QGIS.
QGIS Development Team (2019). QGIS Geographic Information System. Open Source Geospatial Foundation Project. http://qgis.osgeo.org.

^{7}Be normalized trend interpolated into a global map

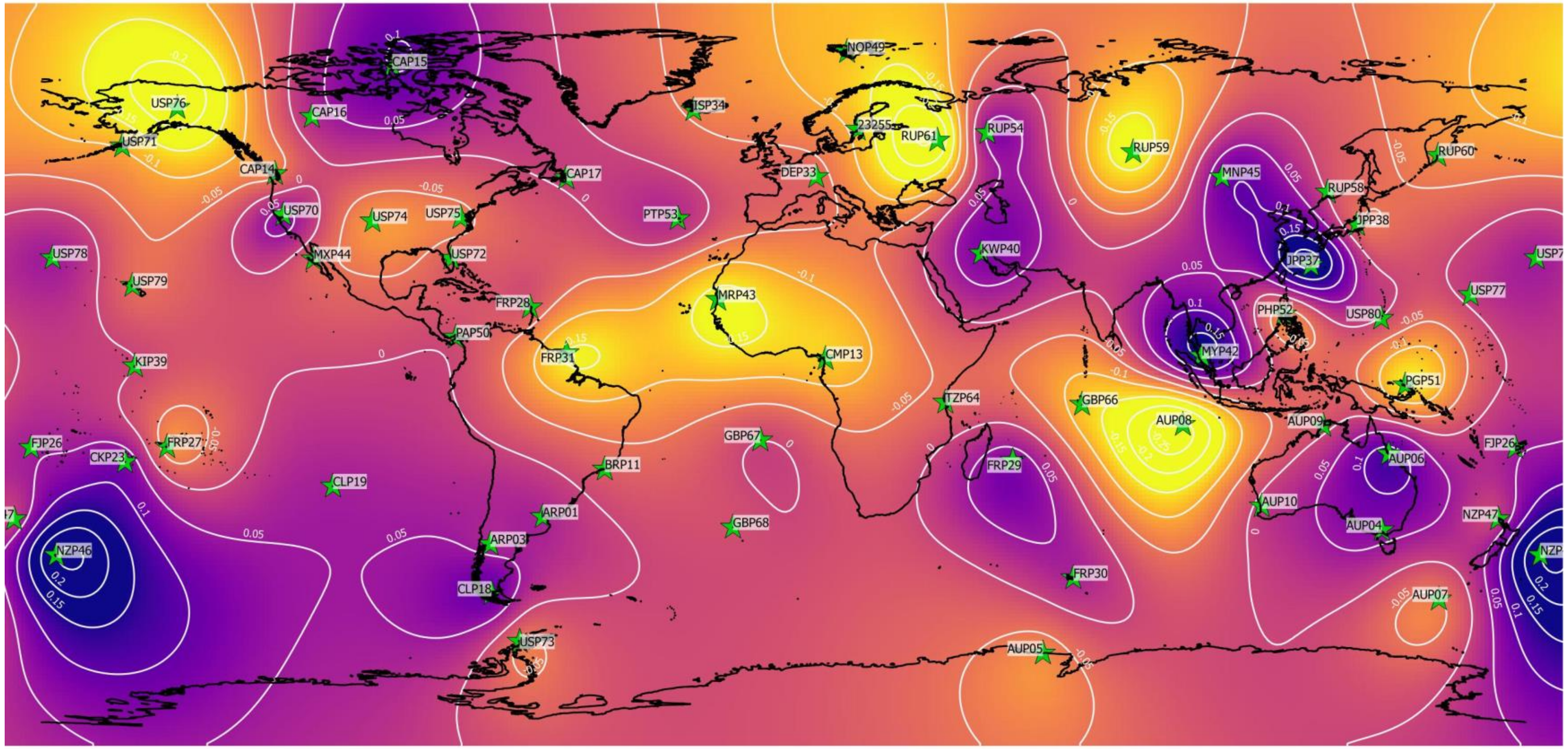

Legend

map

IMS Stations

contour lines

Beryllium-7

-0.1

-0.01

0.01

0.1

0.15

2016

Map executed with QGIS.
QGIS Development Team (2019). QGIS Geographic Information System. Open Source Geospatial Foundation Project. http://qgis.osgeo.org.

^{7}Be normalized trend interpolated into a global map

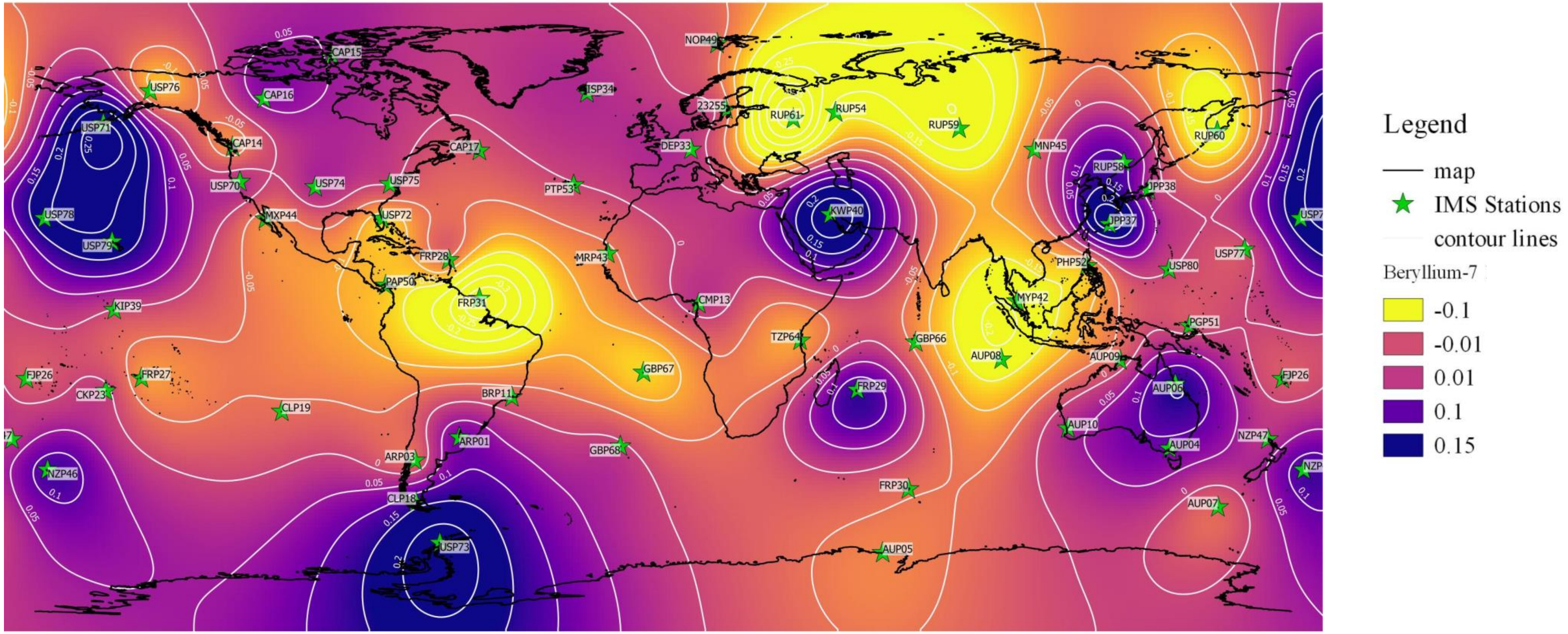

2017

Map executed with QGIS.
QGIS Development Team (2019). QGIS Geographic Information System. Open Source Geospatial Foundation Project. http://qgis.osgeo.org.

^{7}Be normalized trend interpolated into a global map

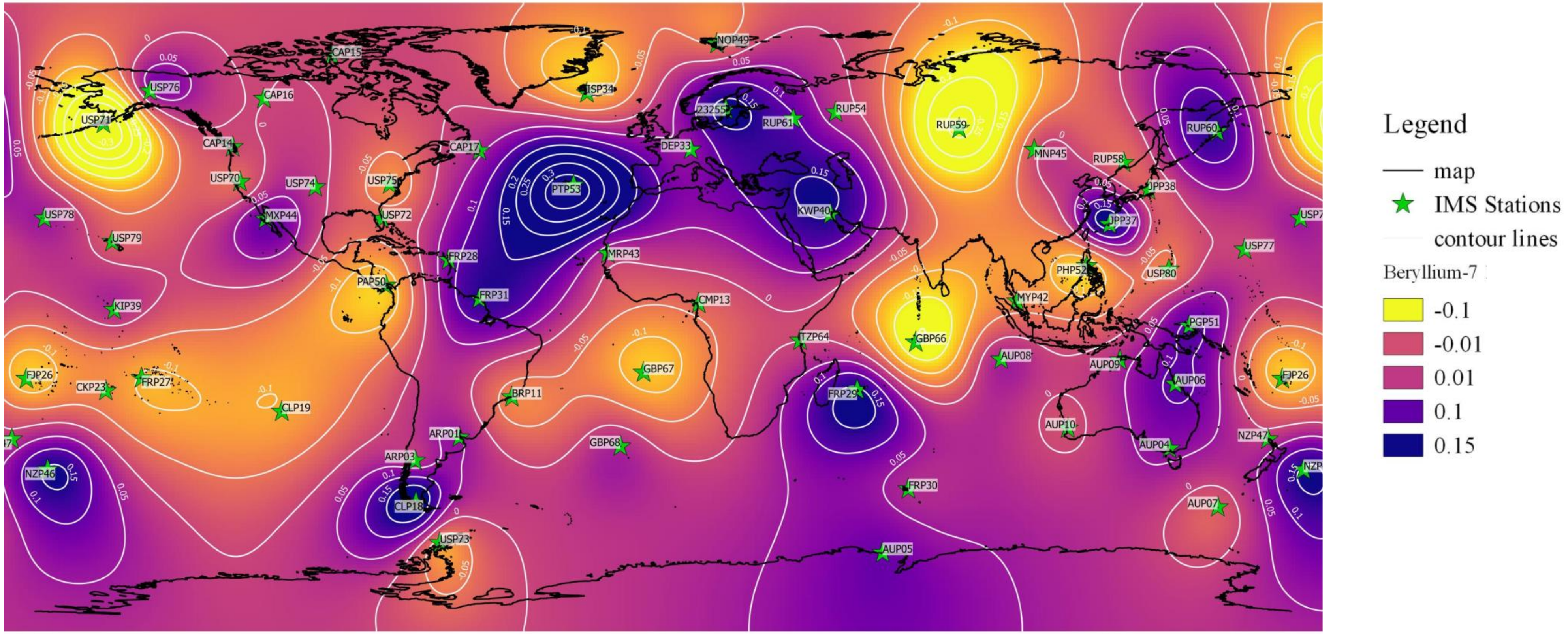

2018

Map executed with QGIS.
QGIS Development Team (2019). QGIS Geographic Information System. Open Source Geospatial Foundation Project. http://qgis.osgeo.org.

^{7}Be normalized trend interpolated into a global map

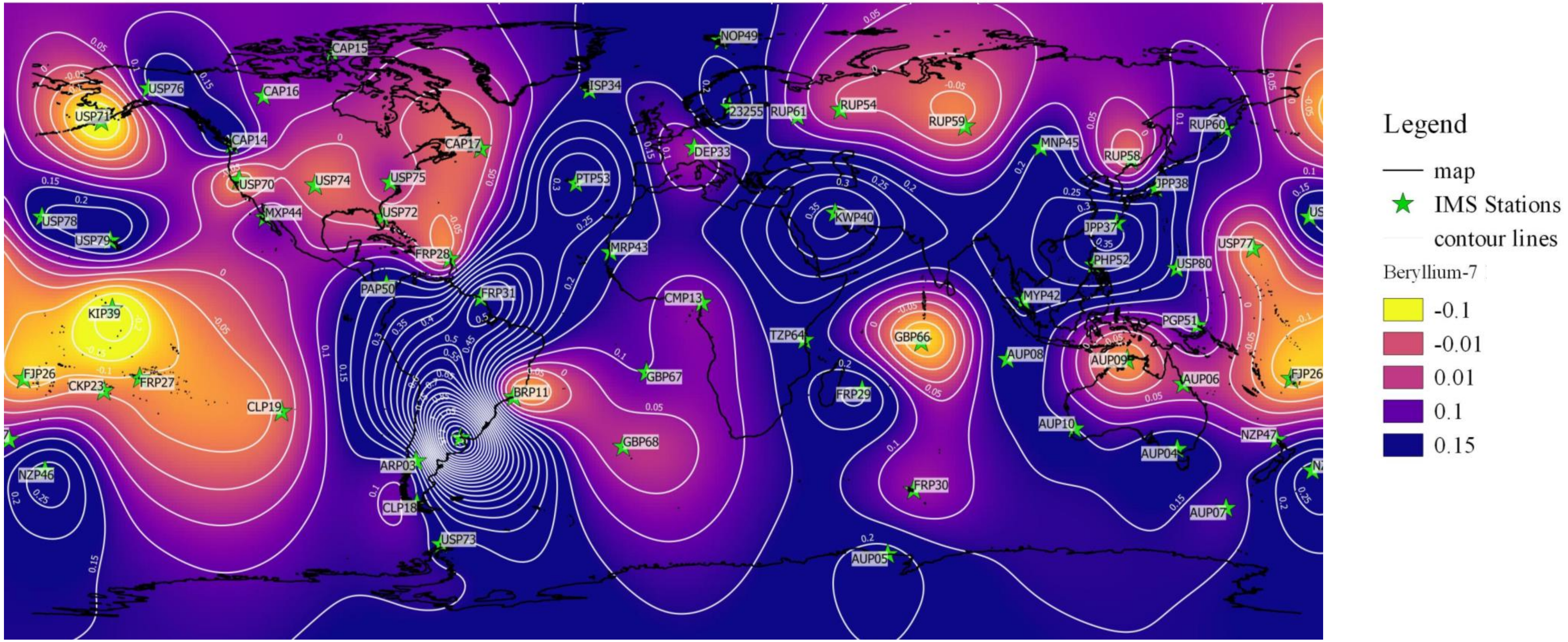

2019

Map executed with QGIS.
QGIS Development Team (2019). QGIS Geographic Information System. Open Source Geospatial Foundation Project. http://qgis.osgeo.org.
