# Supplementary material for: Radioisotopes demonstrate changes in global atmospheric circulation possibly caused by global warming
Source: Sci Rep. 2020 Jul 1;10:10695. doi: 10.1038/s41598-020-66541-5 (PMC7329870; doi:10.1038/s41598-020-66541-5)
Supplement: Supplementary file 5 — Supplementary Information: Beryllium-7 trends. [file 41598_2020_66541_MOESM5_ESM.pdf]

Title: Radioisotopes demonstrate changes in global atmospheric circulation possibly caused by global warming

Authors: Lucrezia Terzi[a,b*], Gerhard Wotawa[c], Michael Schoeppner[d,e], Martin Kalinowski[d], Paul R.J. Saey[b],Philipp Steinmann[f], Lan Luan[g] and Paul W. Staten[g].

Affiliations: [a]Belgian Nuclear Research Centre (SCK•CEN), Mol, Belgium; [b]Technische Universität Wien, Atominstitut, Austria; [c]Zentralanstalt für Meteorologie und Geodynamik (ZAMG), Vienna, Austria, [d]Provisional Technical Secretariat, Preparatory Commission for the Nuclear-Test-Ban Treaty Organization, International Data Centre, Vienna, Austria; [e]Institute of Safety/Security and Risk Sciences, Vienna, Austria; [f]Federal Office of Public Health (BAG), Bern, Switzerland; [g]Indiana University Bloomington, Bloomington, Indiana, USA.

***Corresponding Author:**
Lucrezia Terzi
Ph: +43 6644553833
e-mail: lucrezia.terzi@sckcen.be
[a] SCK•CEN, Belgium Nuclear Research Centre,
Boeretang 200, 2400 Mol, Belgium.
[b] Technische Universität Wien, Atominstitut,
Stadionallee 2, 1020 Wien, Austria.

RN01

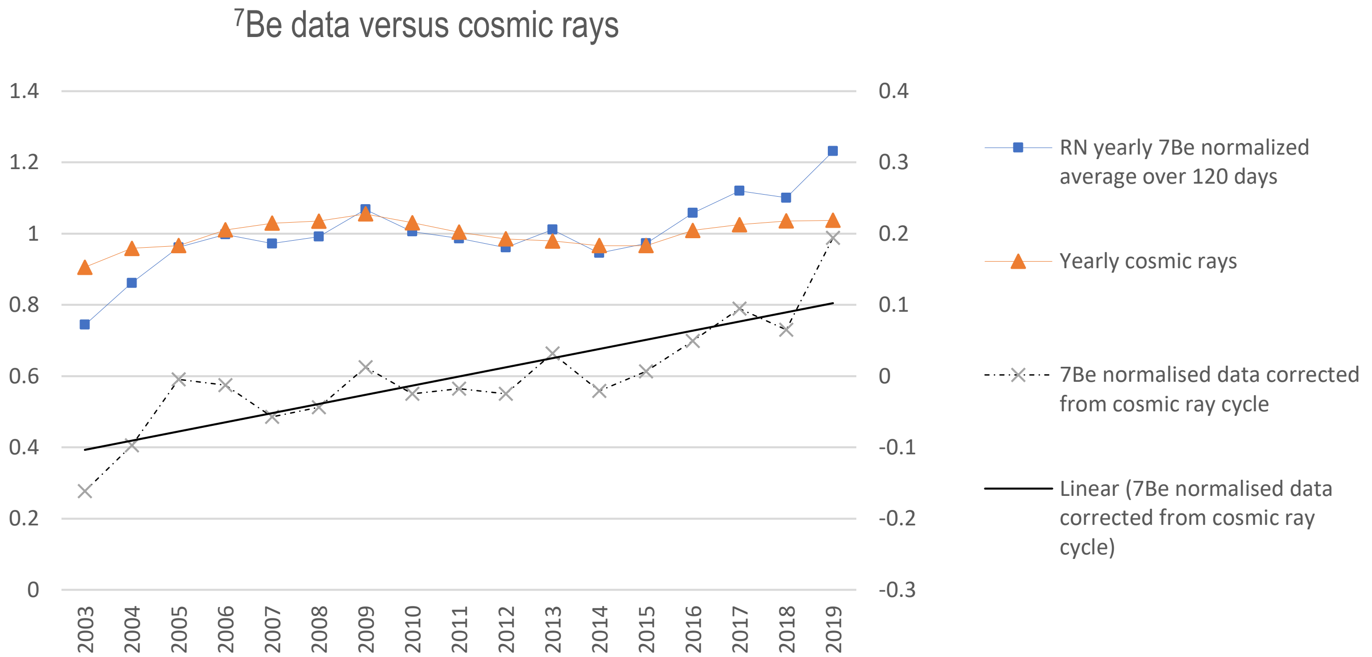

^{7}Be data versus cosmic rays
1.4
1.2
1
0.8
0.6
0.4
0.2
0
0.4
0.3
0.2
0.1
0
-0.1
-0.2
-0.3
2003
2004
2005
2006
2007
2008
2009
2010
2011
2012
2013
2014
2015
2016
2017
2018
2019
RN yearly 7Be normalized average over 120 days
Yearly cosmic rays
7Be normalised data corrected from cosmic ray cycle
Linear (7Be normalised data corrected from cosmic ray cycle)

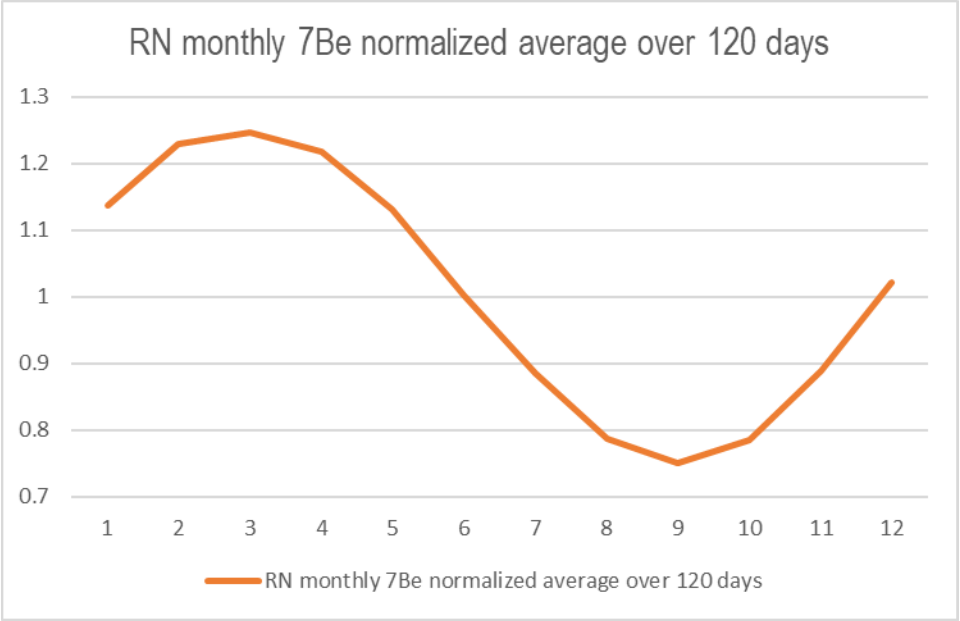

RN monthly 7Be normalized average over 120 days
1.3
1.2
1.1
1
0.9
0.8
0.7
1
2
3
4
5
6
7
8
9
10
11
12
RN monthly 7Be normalized average over 120 days

RN03

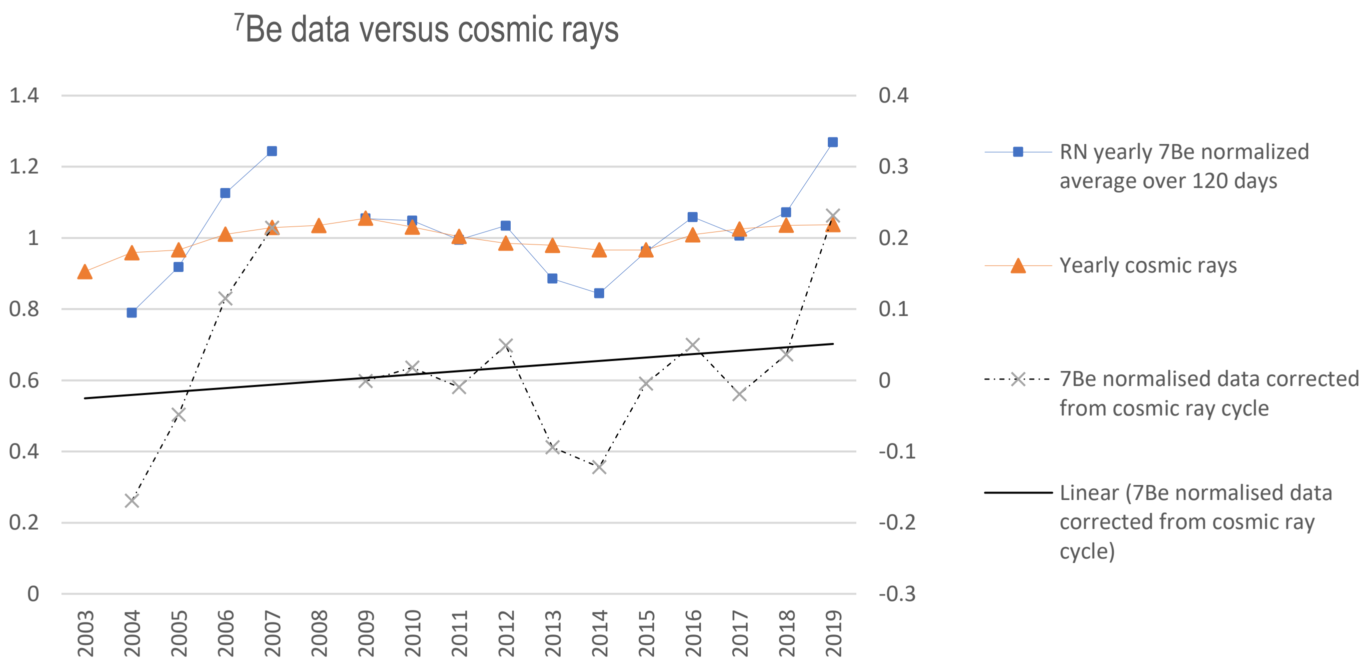

^{7}Be data versus cosmic rays
1.4
1.2
1
0.8
0.6
0.4
0.2
0
0.4
0.3
0.2
0.1
0
-0.1
-0.2
-0.3
2003 2004 2005 2006 2007 2008 2009 2010 2011 2012 2013 2014 2015 2016 2017 2018 2019
RN yearly 7Be normalized average over 120 days
Yearly cosmic rays
7Be normalised data corrected from cosmic ray cycle
Linear (7Be normalised data corrected from cosmic ray cycle)

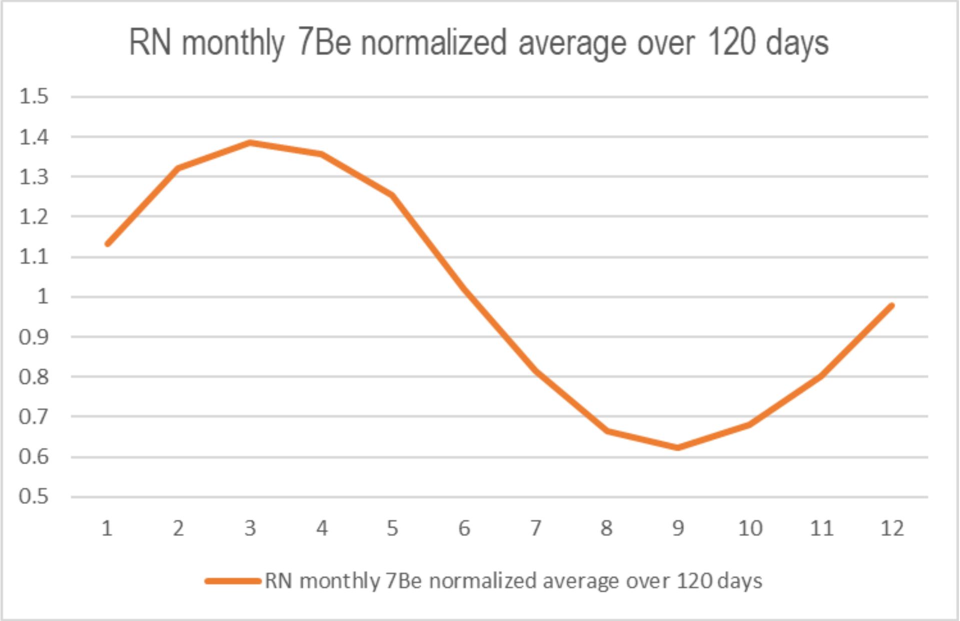

RN monthly 7Be normalized average over 120 days
1.5
1.4
1.3
1.2
1.1
1
0.9
0.8
0.7
0.6
0.5
1 2 3 4 5 6 7 8 9 10 11 12
RN monthly 7Be normalized average over 120 days

RN04

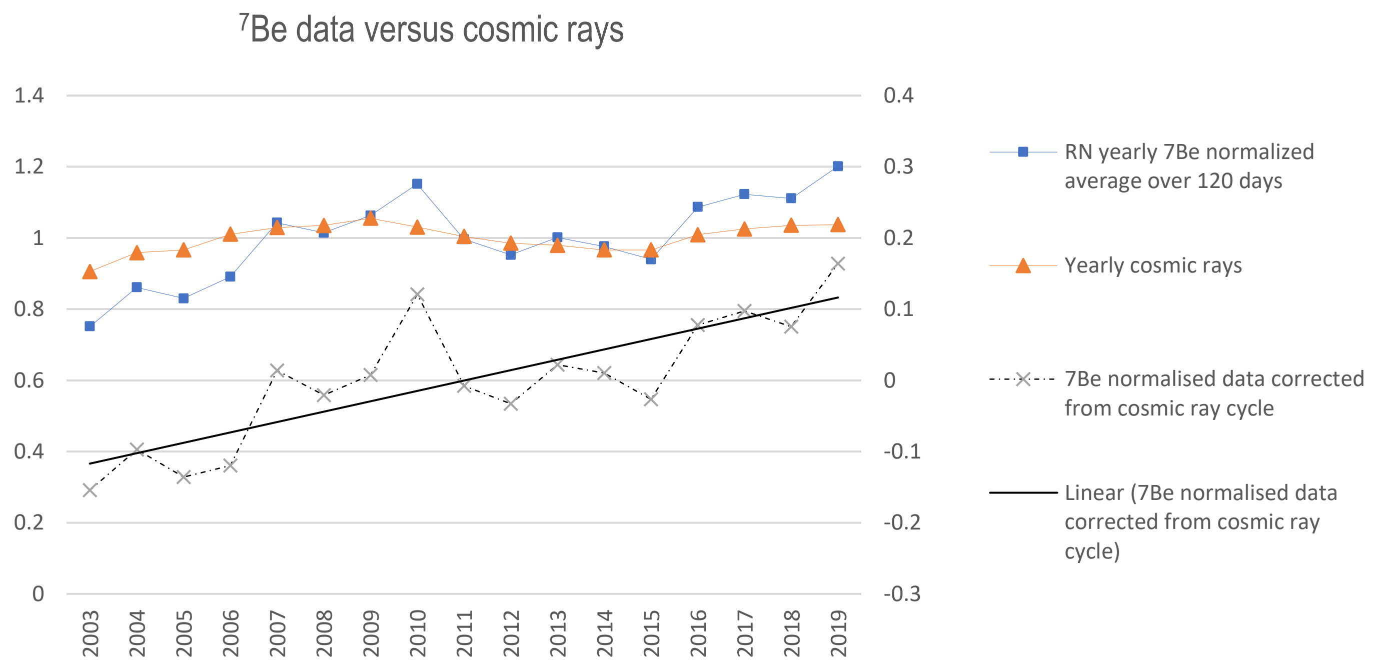

^{7}Be data versus cosmic rays
1.4
1.2
1
0.8
0.6
0.4
0.2
0
0.4
0.3
0.2
0.1
0
-0.1
-0.2
-0.3
2003
2004
2005
2006
2007
2008
2009
2010
2011
2012
2013
2014
2015
2016
2017
2018
2019
RN yearly 7Be normalized average over 120 days
Yearly cosmic rays
7Be normalised data corrected from cosmic ray cycle
Linear (7Be normalised data corrected from cosmic ray cycle)

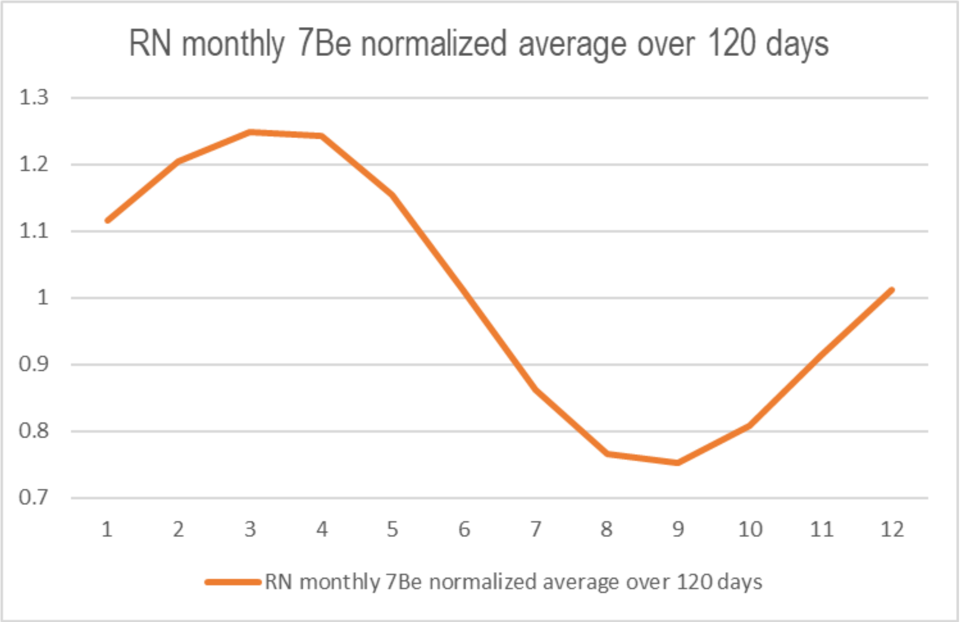

RN monthly 7Be normalized average over 120 days
1.3
1.2
1.1
1
0.9
0.8
0.7
1
2
3
4
5
6
7
8
9
10
11
12
RN monthly 7Be normalized average over 120 days

RN05

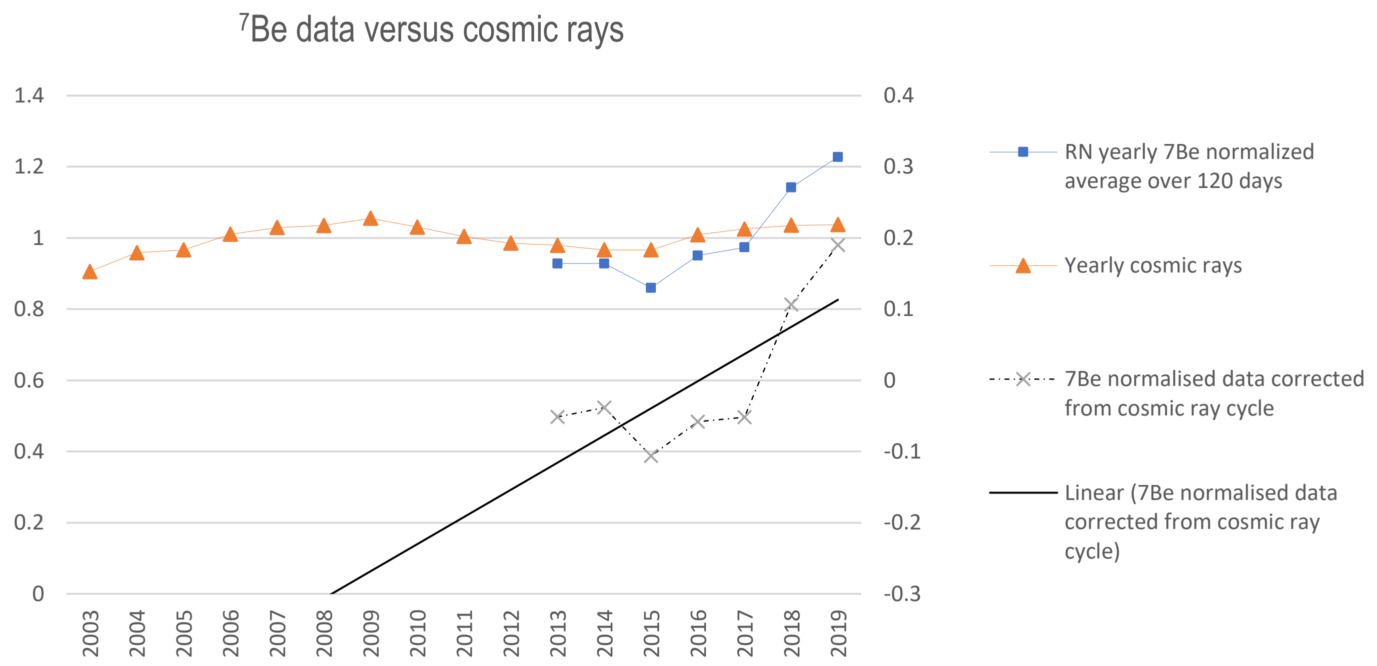

^{7}Be data versus cosmic rays
1.4
1.2
1
0.8
0.6
0.4
0.2
0
0.4
0.3
0.2
0.1
0
-0.1
-0.2
-0.3
2003
2004
2005
2006
2007
2008
2009
2010
2011
2012
2013
2014
2015
2016
2017
2018
2019
RN yearly 7Be normalized average over 120 days
Yearly cosmic rays
7Be normalised data corrected from cosmic ray cycle
Linear (7Be normalised data corrected from cosmic ray cycle)

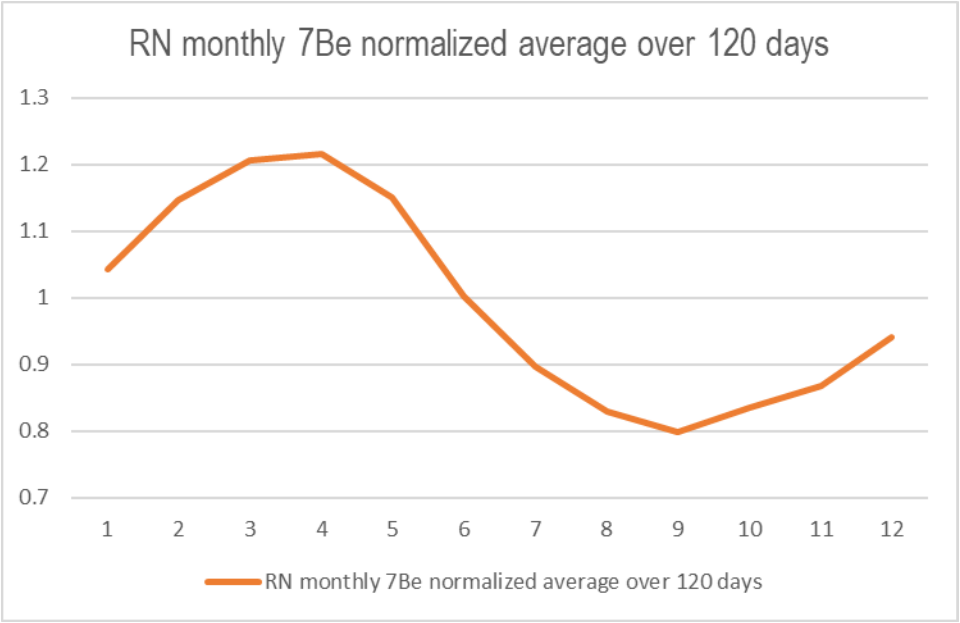

RN monthly 7Be normalized average over 120 days
1.3
1.2
1.1
1
0.9
0.8
0.7
1
2
3
4
5
6
7
8
9
10
11
12
RN monthly 7Be normalized average over 120 days

RN06

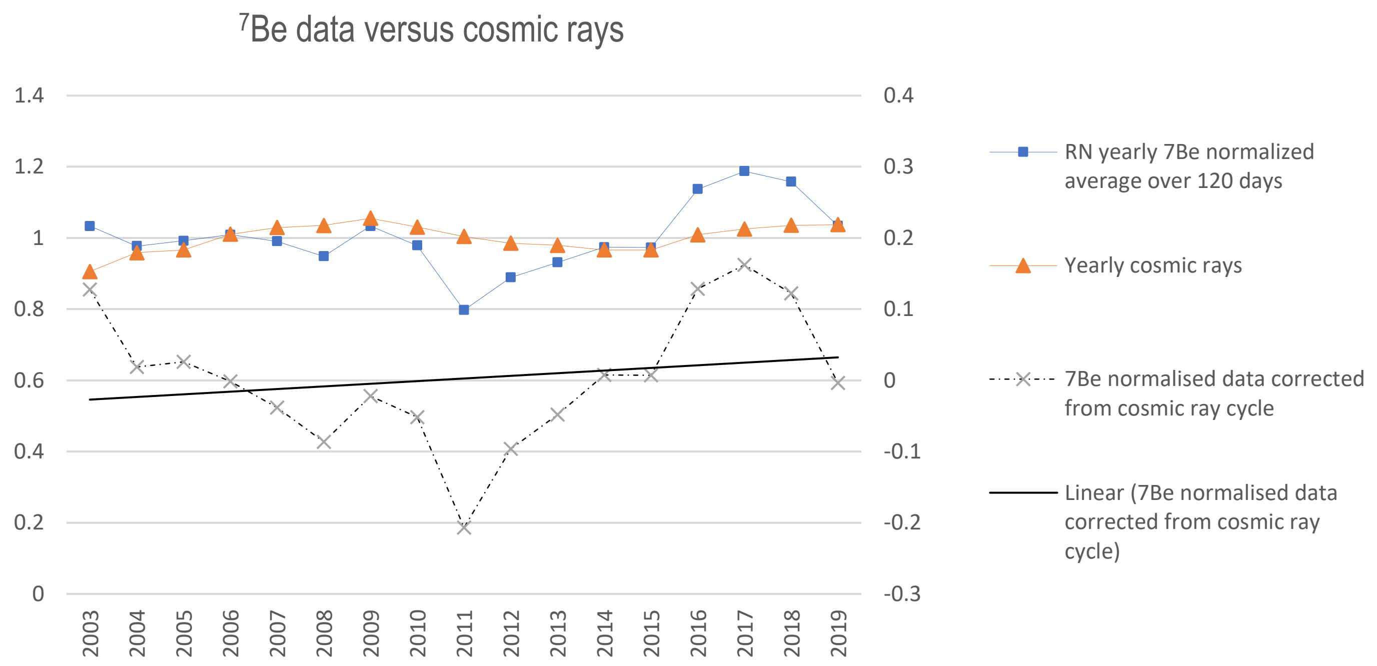
^{7}Be data versus cosmic rays
1.4
1.2
1
0.8
0.6
0.4
0.2
0
0.4
0.3
0.2
0.1
0
-0.1
-0.2
-0.3
2003
2004
2005
2006
2007
2008
2009
2010
2011
2012
2013
2014
2015
2016
2017
2018
2019
RN yearly 7Be normalized average over 120 days
Yearly cosmic rays
7Be normalised data corrected from cosmic ray cycle
Linear (7Be normalised data corrected from cosmic ray cycle)

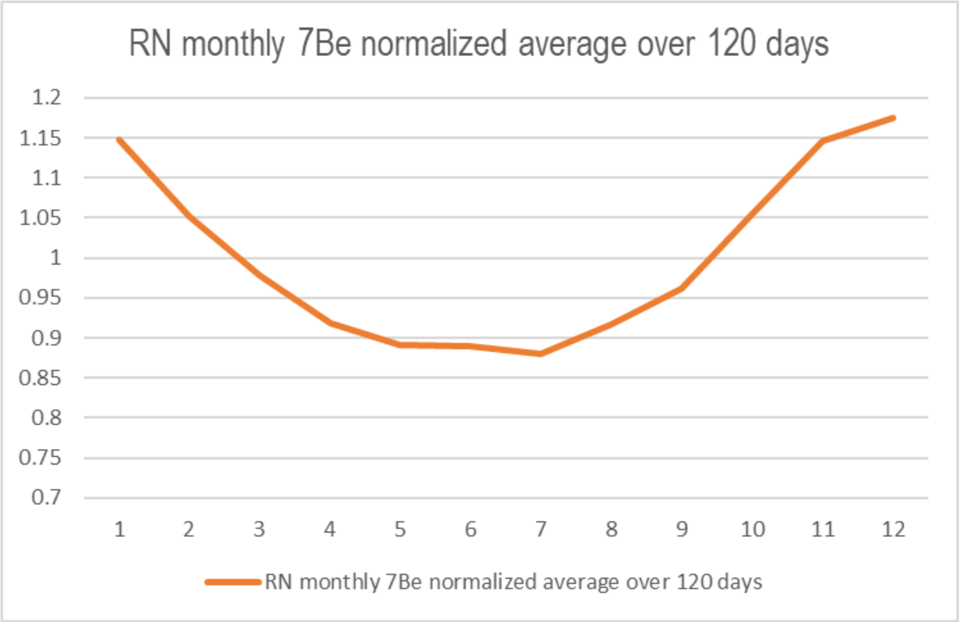
RN monthly 7Be normalized average over 120 days
1.2
1.15
1.1
1.05
1
0.95
0.9
0.85
0.8
0.75
0.7
1
2
3
4
5
6
7
8
9
10
11
12
RN monthly 7Be normalized average over 120 days

RN07

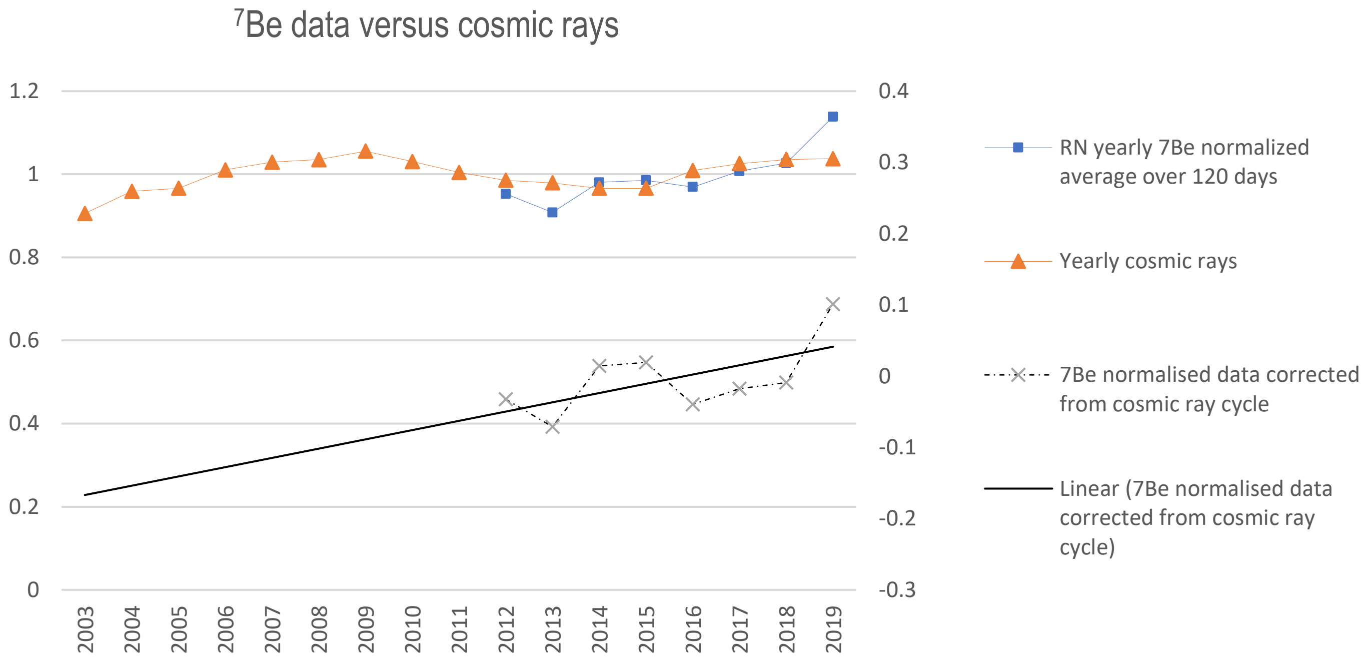
7Be data versus cosmic rays
1.2
1
0.8
0.6
0.4
0.2
0
0.4
0.3
0.2
0.1
0
-0.1
-0.2
-0.3
2003 2004 2005 2006 2007 2008 2009 2010 2011 2012 2013 2014 2015 2016 2017 2018 2019
RN yearly 7Be normalized average over 120 days
Yearly cosmic rays
7Be normalised data corrected from cosmic ray cycle
Linear (7Be normalised data corrected from cosmic ray cycle)

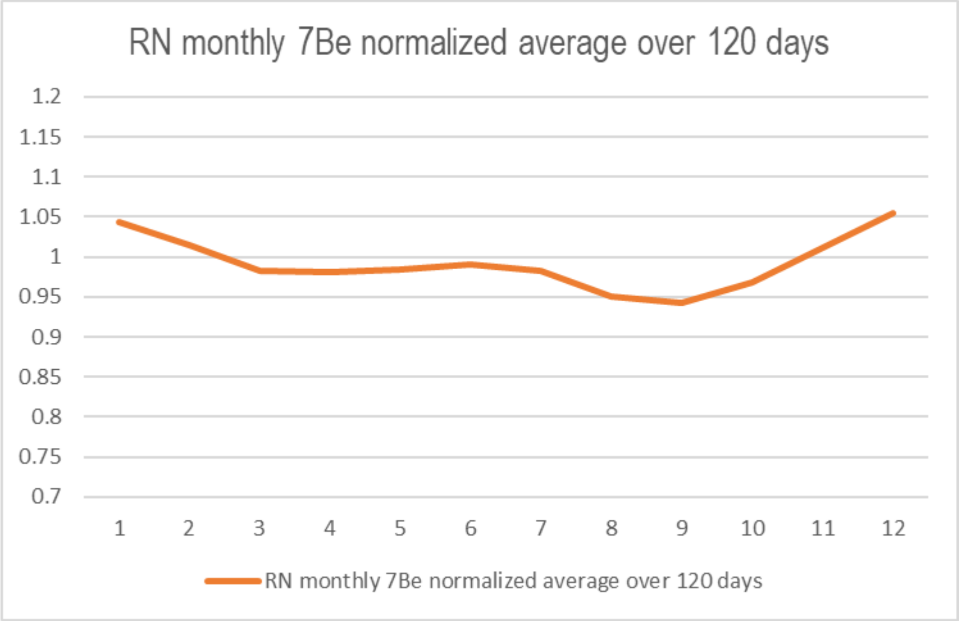
RN monthly 7Be normalized average over 120 days
1.2
1.15
1.1
1.05
1
0.95
0.9
0.85
0.8
0.75
0.7
1 2 3 4 5 6 7 8 9 10 11 12
RN monthly 7Be normalized average over 120 days

RN08

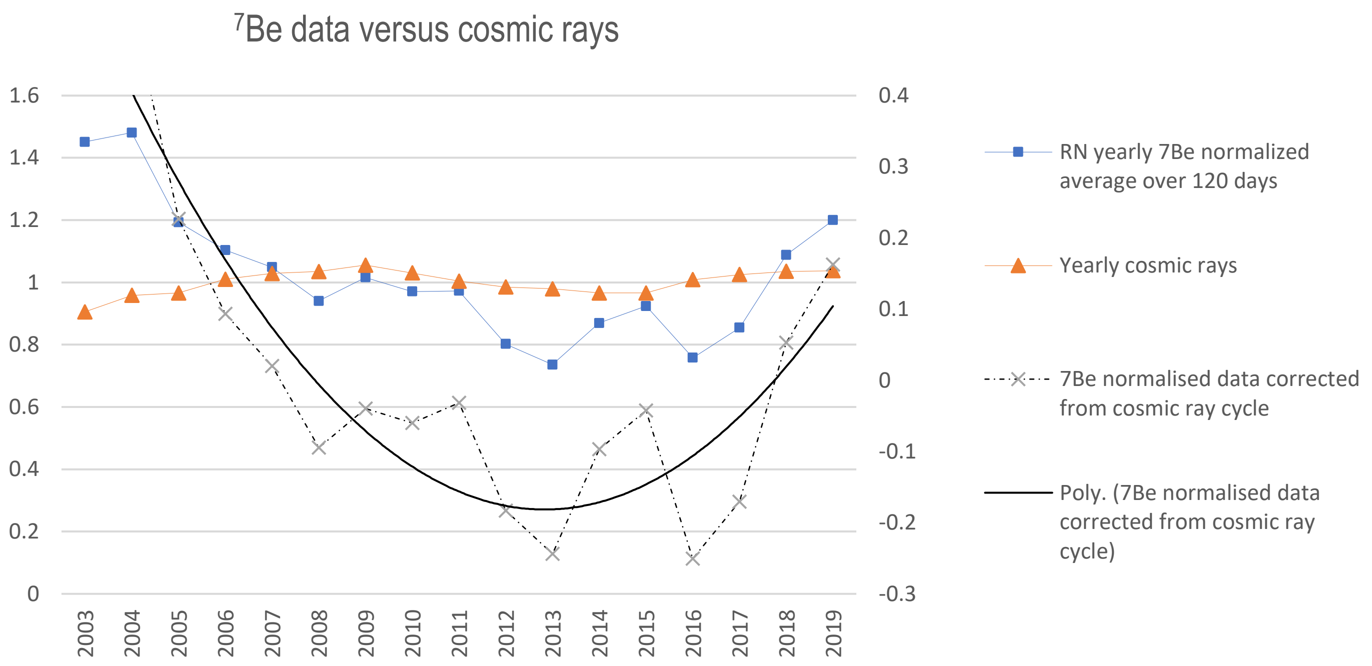

^{7}Be data versus cosmic rays
1.6
1.4
1.2
1
0.8
0.6
0.4
0.2
0
0.4
0.3
0.2
0.1
0
-0.1
-0.2
-0.3
2003
2004
2005
2006
2007
2008
2009
2010
2011
2012
2013
2014
2015
2016
2017
2018
2019
RN yearly 7Be normalized average over 120 days
Yearly cosmic rays
7Be normalised data corrected from cosmic ray cycle
Poly. (7Be normalised data corrected from cosmic ray cycle)

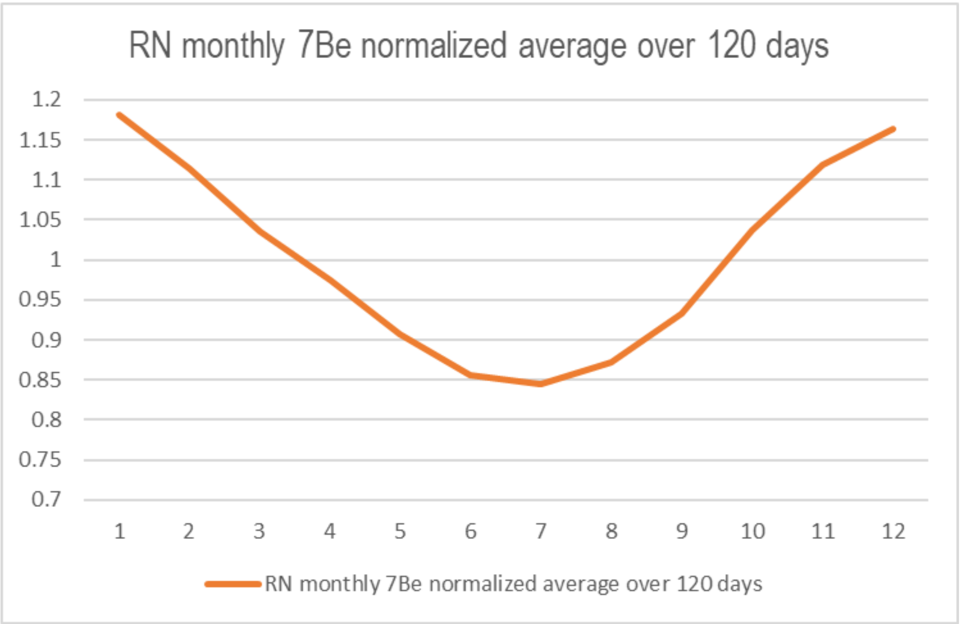

RN monthly 7Be normalized average over 120 days
1.2
1.15
1.1
1.05
1
0.95
0.9
0.85
0.8
0.75
0.7
1
2
3
4
5
6
7
8
9
10
11
12
RN monthly 7Be normalized average over 120 days

RN09

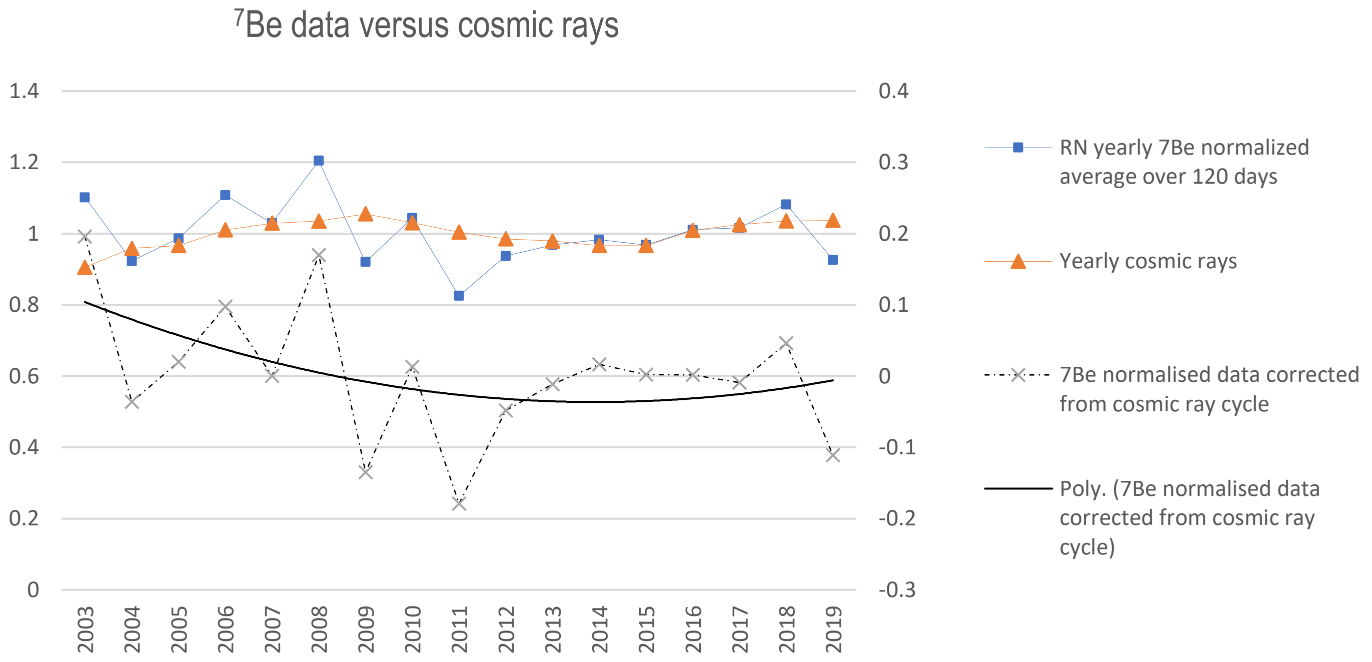

⁷Be data versus cosmic rays
1.4
1.2
1
0.8
0.6
0.4
0.2
0
0.4
0.3
0.2
0.1
0
-0.1
-0.2
-0.3
2003
2004
2005
2006
2007
2008
2009
2010
2011
2012
2013
2014
2015
2016
2017
2018
2019
RN yearly 7Be normalized average over 120 days
Yearly cosmic rays
7Be normalised data corrected from cosmic ray cycle
Poly. (7Be normalised data corrected from cosmic ray cycle)

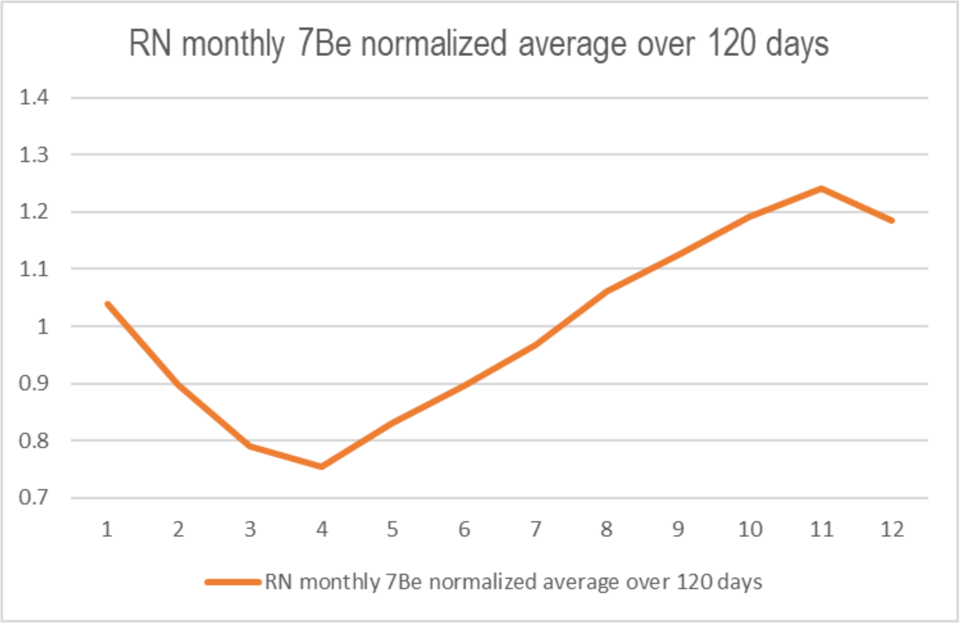

RN monthly 7Be normalized average over 120 days
1.4
1.3
1.2
1.1
1
0.9
0.8
0.7
1
2
3
4
5
6
7
8
9
10
11
12
RN monthly 7Be normalized average over 120 days

RN10

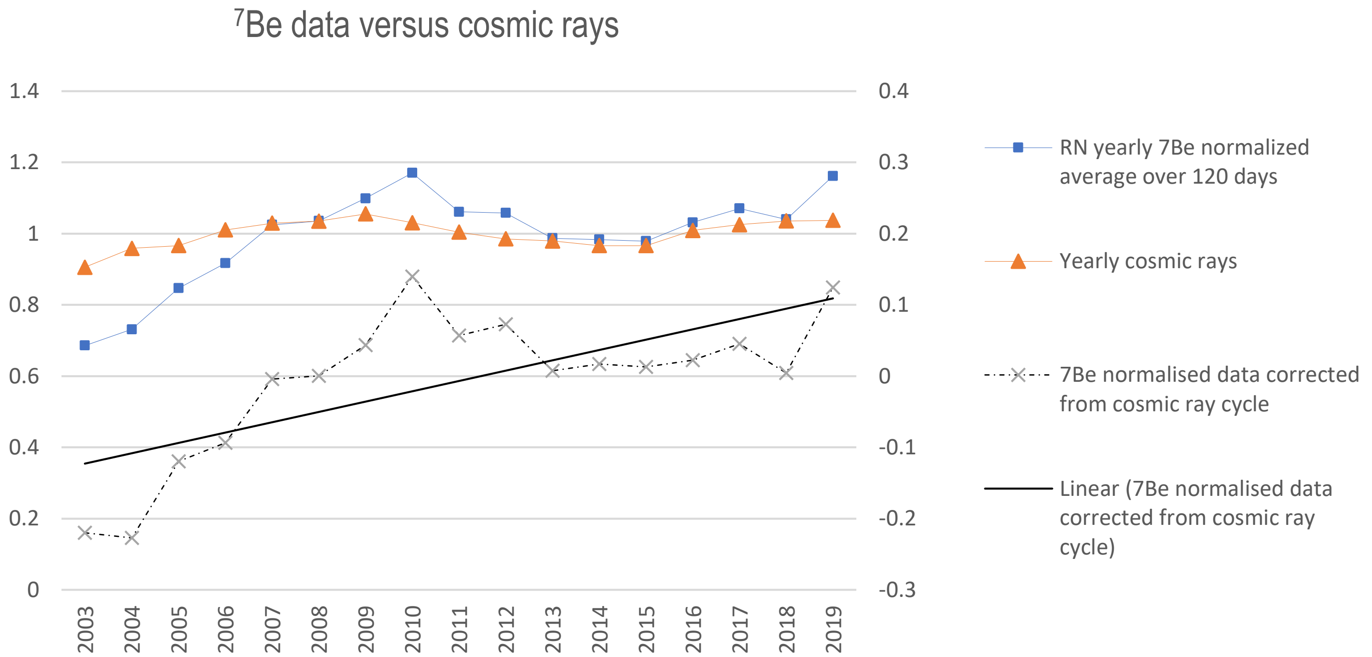

^{7}Be data versus cosmic rays
1.4
1.2
1
0.8
0.6
0.4
0.2
0
0.4
0.3
0.2
0.1
0
-0.1
-0.2
-0.3
2003
2004
2005
2006
2007
2008
2009
2010
2011
2012
2013
2014
2015
2016
2017
2018
2019
RN yearly 7Be normalized average over 120 days
Yearly cosmic rays
7Be normalised data corrected from cosmic ray cycle
Linear (7Be normalised data corrected from cosmic ray cycle)

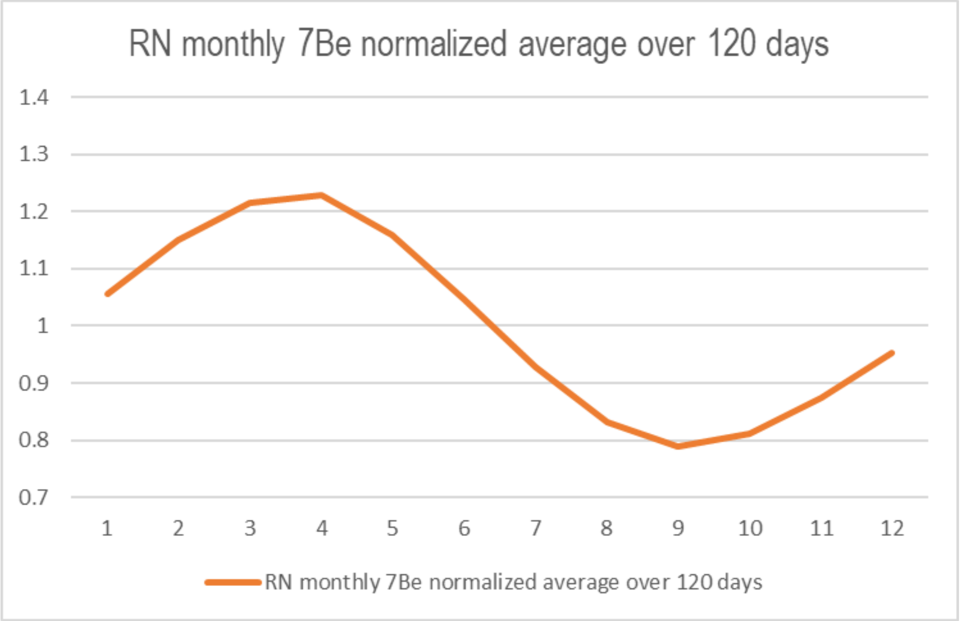

RN monthly 7Be normalized average over 120 days
1.4
1.3
1.2
1.1
1
0.9
0.8
0.7
1
2
3
4
5
6
7
8
9
10
11
12
RN monthly 7Be normalized average over 120 days

RN11

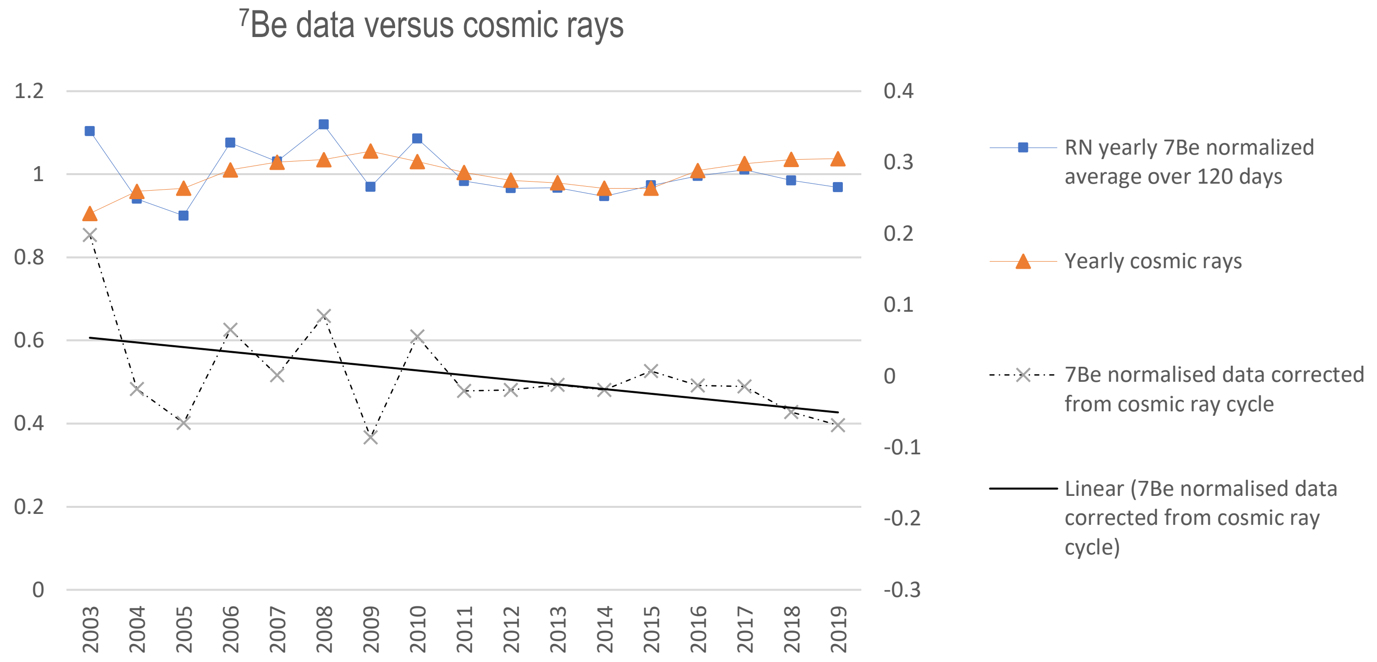

^{7}Be data versus cosmic rays
1.2
1
0.8
0.6
0.4
0.2
0
0.4
0.3
0.2
0.1
0
-0.1
-0.2
-0.3
2003
2004
2005
2006
2007
2008
2009
2010
2011
2012
2013
2014
2015
2016
2017
2018
2019
RN yearly 7Be normalized average over 120 days
Yearly cosmic rays
7Be normalised data corrected from cosmic ray cycle
Linear (7Be normalised data corrected from cosmic ray cycle)

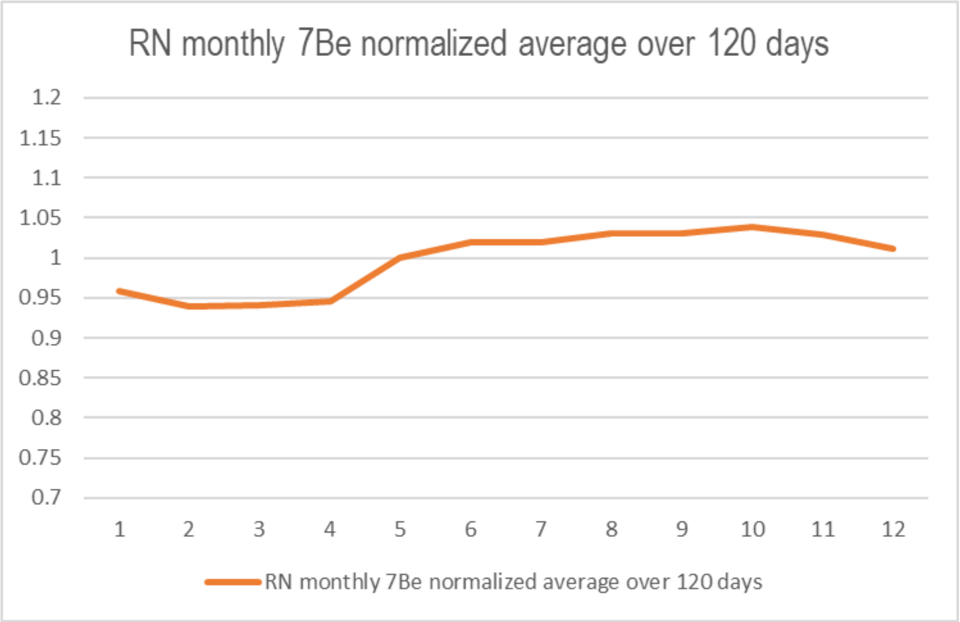

RN monthly 7Be normalized average over 120 days
1.2
1.15
1.1
1.05
1
0.95
0.9
0.85
0.8
0.75
0.7
1
2
3
4
5
6
7
8
9
10
11
12
RN monthly 7Be normalized average over 120 days

RN13

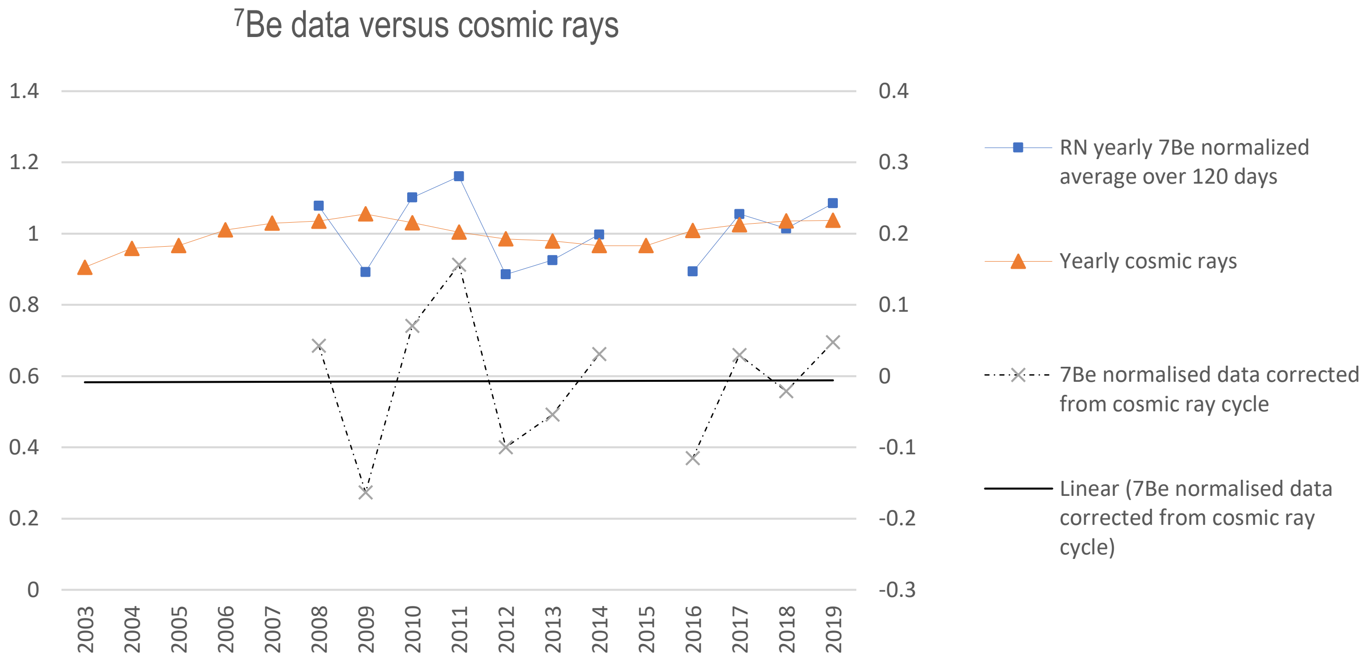

^{7}Be data versus cosmic rays
1.4
1.2
1
0.8
0.6
0.4
0.2
0
0.4
0.3
0.2
0.1
0
-0.1
-0.2
-0.3
2003
2004
2005
2006
2007
2008
2009
2010
2011
2012
2013
2014
2015
2016
2017
2018
2019
RN yearly 7Be normalized average over 120 days
Yearly cosmic rays
7Be normalised data corrected from cosmic ray cycle
Linear (7Be normalised data corrected from cosmic ray cycle)

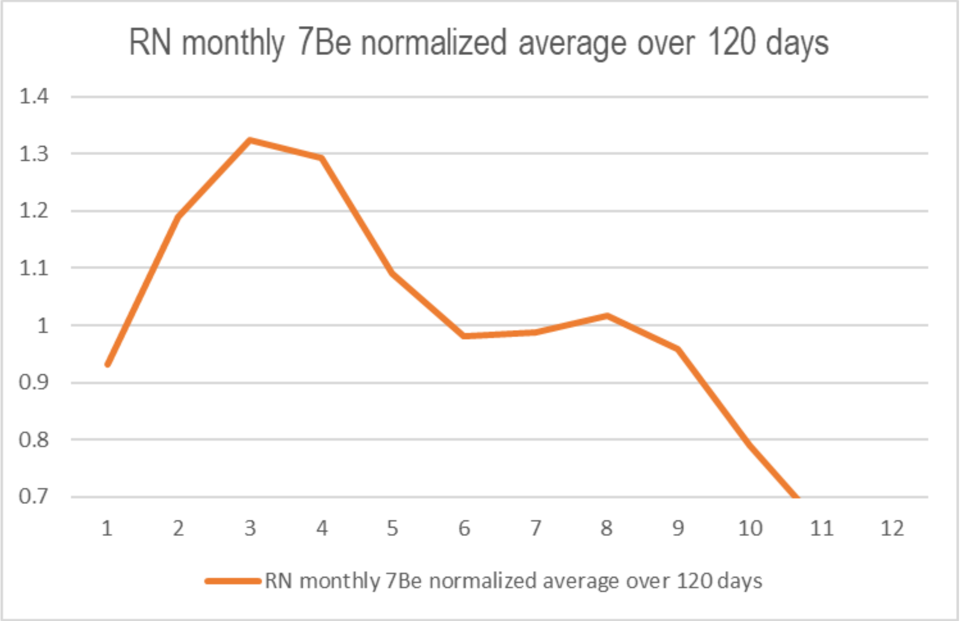

RN monthly 7Be normalized average over 120 days
1.4
1.3
1.2
1.1
1
0.9
0.8
0.7
1
2
3
4
5
6
7
8
9
10
11
12
RN monthly 7Be normalized average over 120 days

RN14

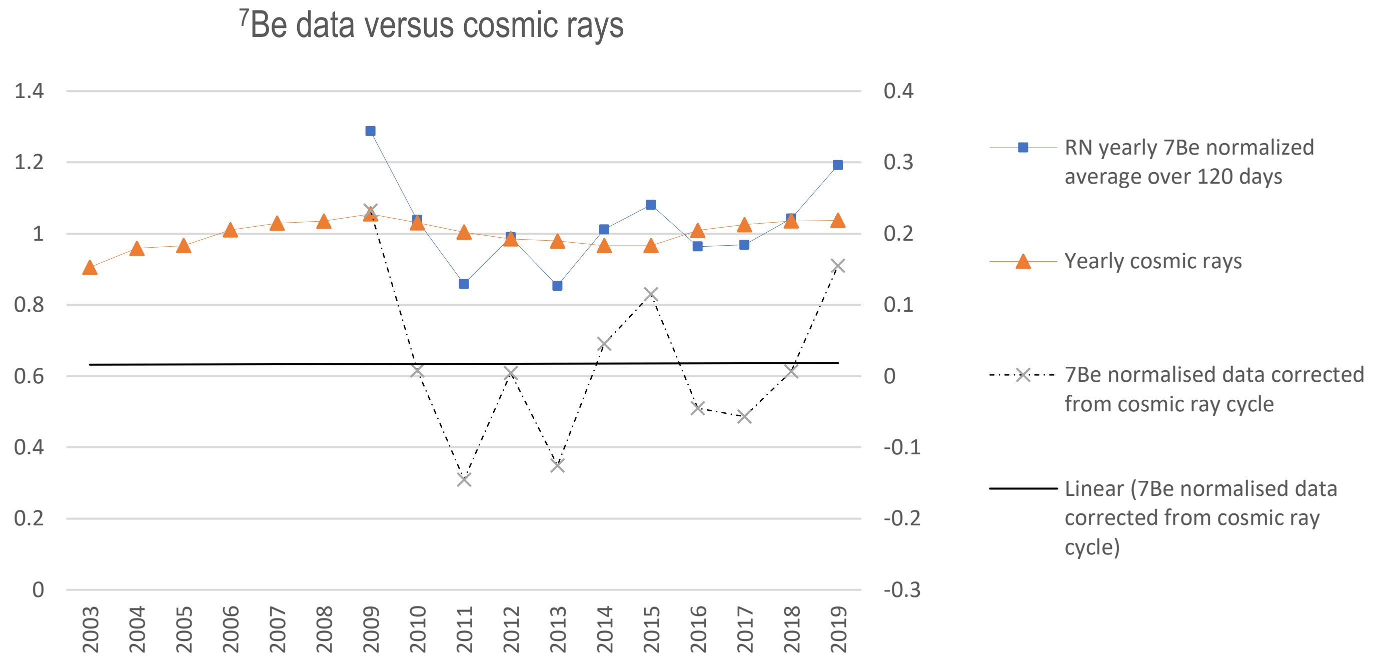

^{7}Be data versus cosmic rays
1.4
1.2
1
0.8
0.6
0.4
0.2
0
0.4
0.3
0.2
0.1
0
-0.1
-0.2
-0.3
2003
2004
2005
2006
2007
2008
2009
2010
2011
2012
2013
2014
2015
2016
2017
2018
2019
RN yearly 7Be normalized average over 120 days
Yearly cosmic rays
7Be normalised data corrected from cosmic ray cycle
Linear (7Be normalised data corrected from cosmic ray cycle)

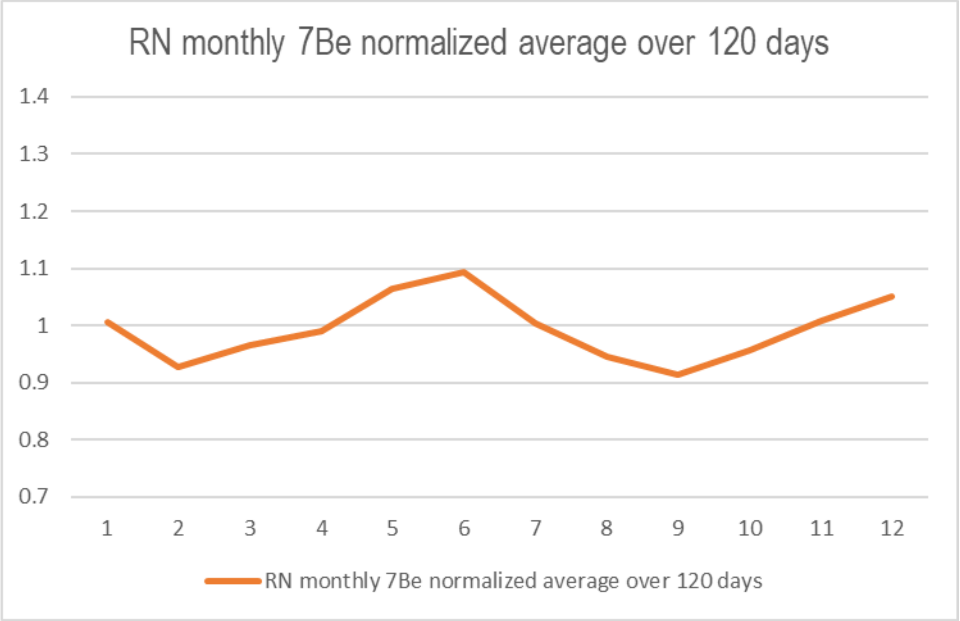

RN monthly 7Be normalized average over 120 days
1.4
1.3
1.2
1.1
1
0.9
0.8
0.7
1
2
3
4
5
6
7
8
9
10
11
12
RN monthly 7Be normalized average over 120 days

RN15

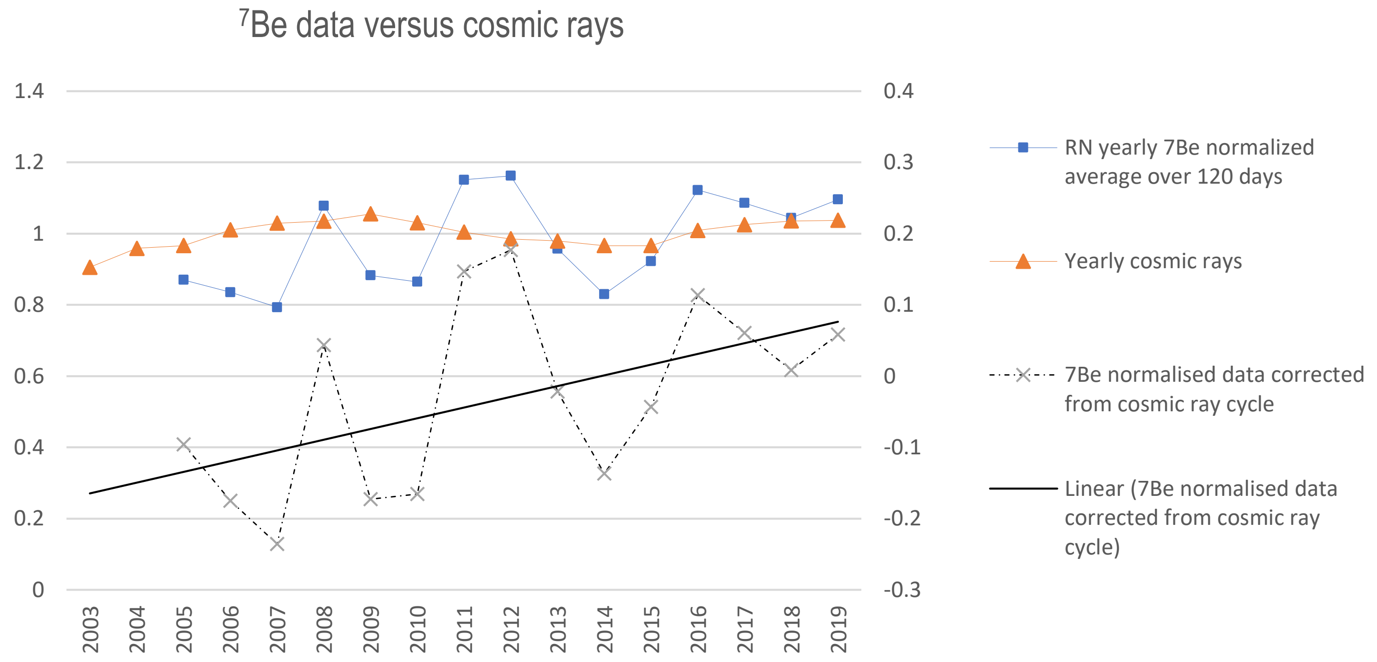

⁷Be data versus cosmic rays
1.4
1.2
1
0.8
0.6
0.4
0.2
0
0.4
0.3
0.2
0.1
0
-0.1
-0.2
-0.3
2003
2004
2005
2006
2007
2008
2009
2010
2011
2012
2013
2014
2015
2016
2017
2018
2019
RN yearly 7Be normalized average over 120 days
Yearly cosmic rays
7Be normalised data corrected from cosmic ray cycle
Linear (7Be normalised data corrected from cosmic ray cycle)

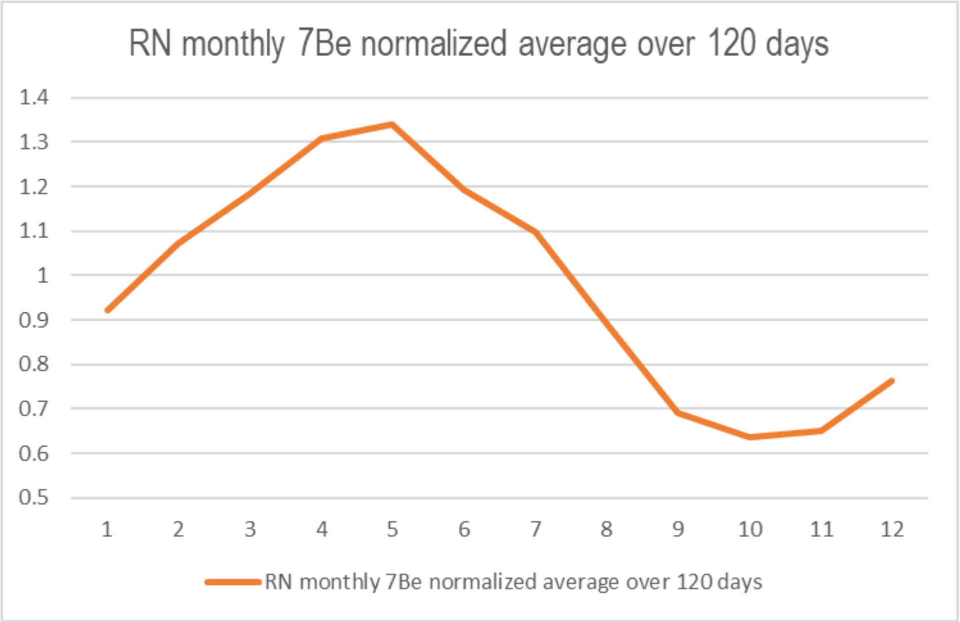

RN monthly 7Be normalized average over 120 days
1.4
1.3
1.2
1.1
1
0.9
0.8
0.7
0.6
0.5
1
2
3
4
5
6
7
8
9
10
11
12
RN monthly 7Be normalized average over 120 days

RN16

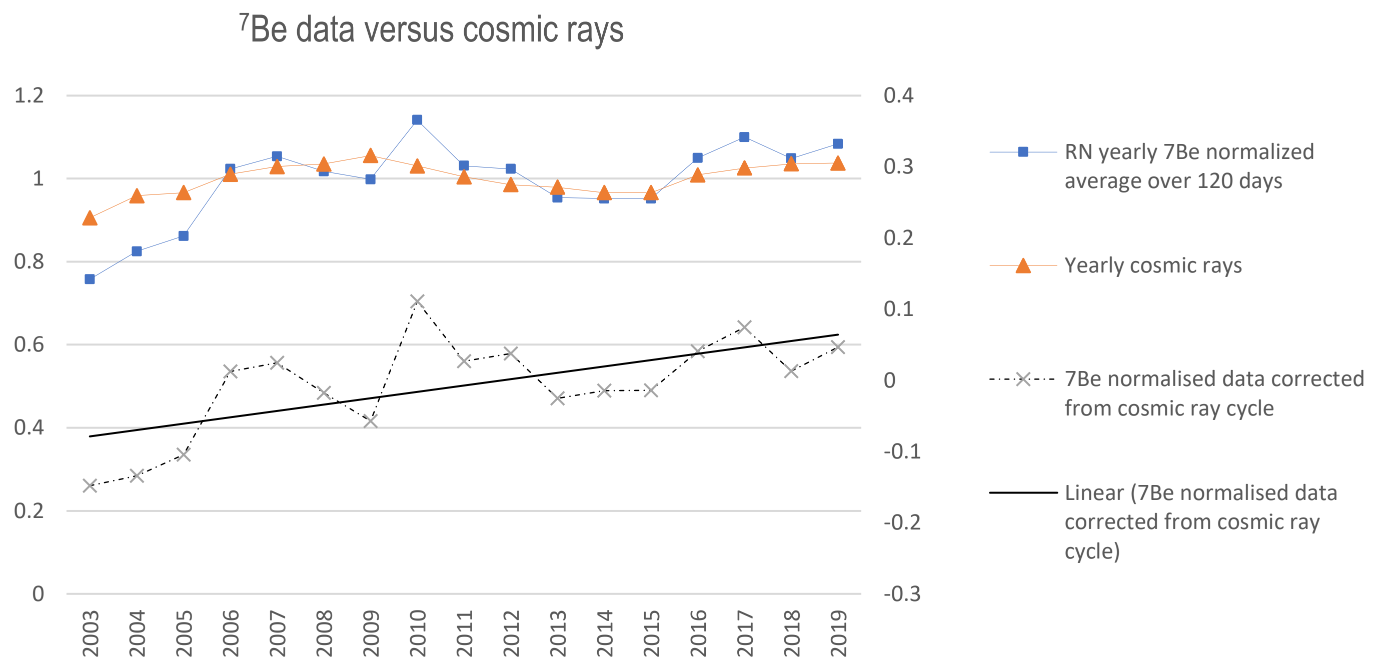

^{7}Be data versus cosmic rays
1.2
1
0.8
0.6
0.4
0.2
0
0.4
0.3
0.2
0.1
0
-0.1
-0.2
-0.3
2003
2004
2005
2006
2007
2008
2009
2010
2011
2012
2013
2014
2015
2016
2017
2018
2019
RN yearly 7Be normalized average over 120 days
Yearly cosmic rays
7Be normalised data corrected from cosmic ray cycle
Linear (7Be normalised data corrected from cosmic ray cycle)

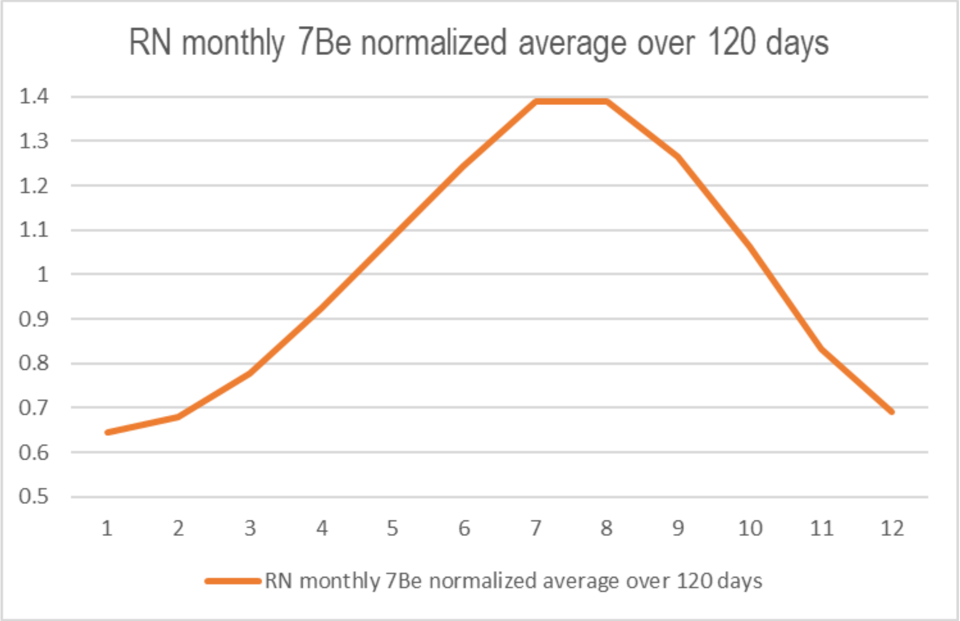

RN monthly 7Be normalized average over 120 days
1.4
1.3
1.2
1.1
1
0.9
0.8
0.7
0.6
0.5
1
2
3
4
5
6
7
8
9
10
11
12
RN monthly 7Be normalized average over 120 days

RN17

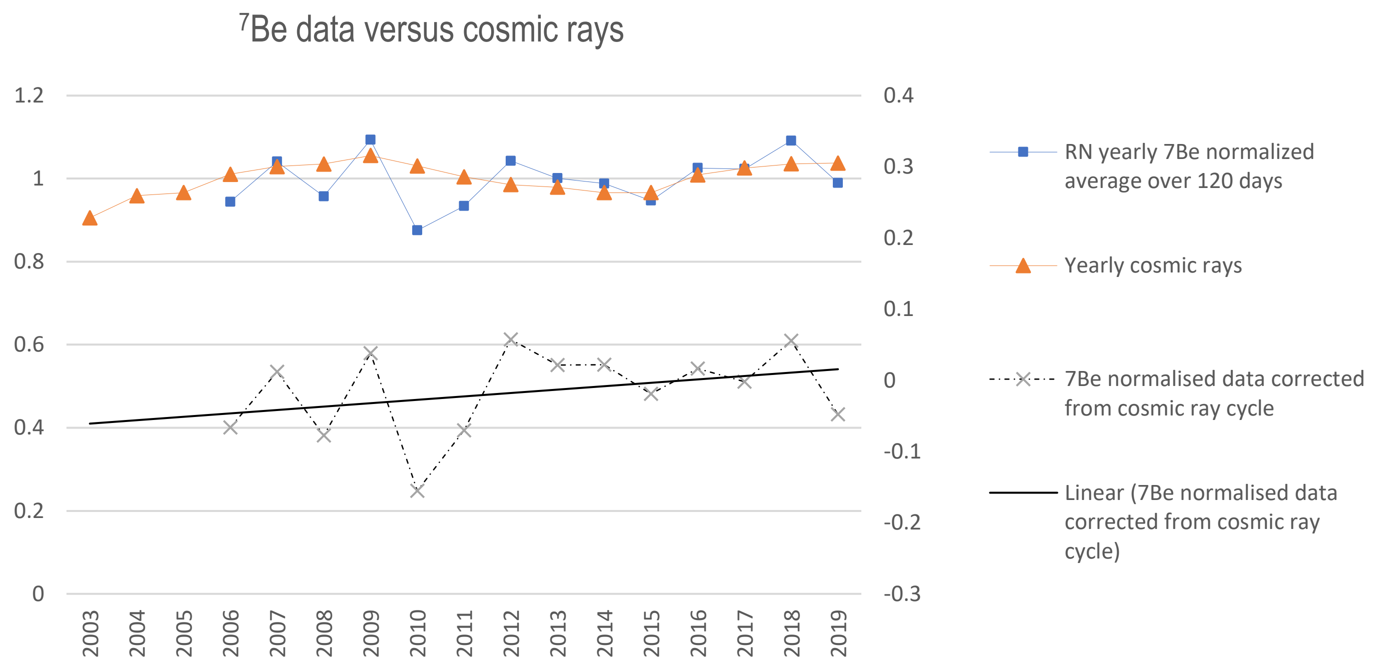

^{7}Be data versus cosmic rays
1.2
1
0.8
0.6
0.4
0.2
0
0.4
0.3
0.2
0.1
0
-0.1
-0.2
-0.3
2003
2004
2005
2006
2007
2008
2009
2010
2011
2012
2013
2014
2015
2016
2017
2018
2019
RN yearly 7Be normalized average over 120 days
Yearly cosmic rays
7Be normalised data corrected from cosmic ray cycle
Linear (7Be normalised data corrected from cosmic ray cycle)

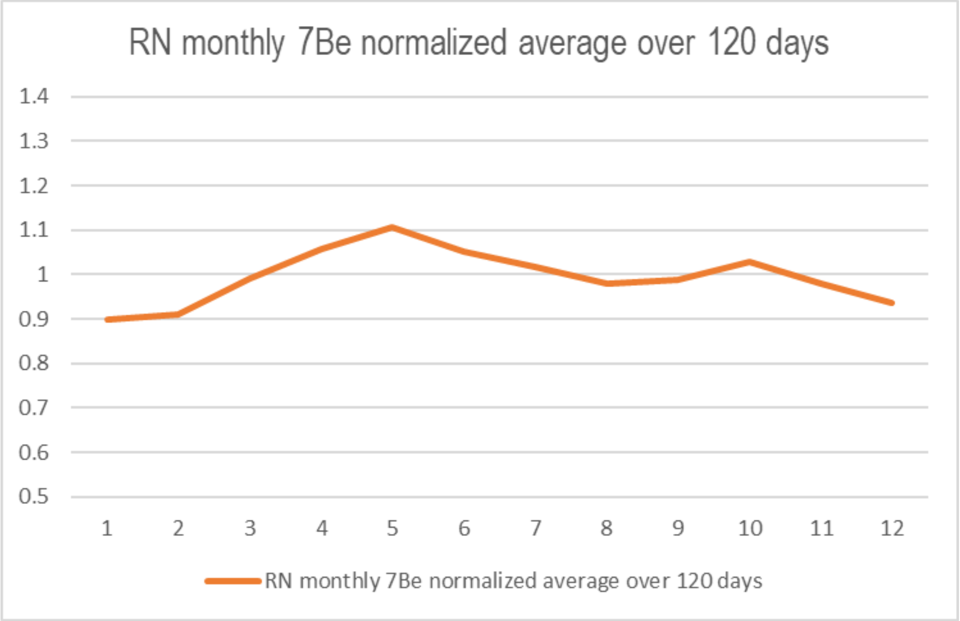

RN monthly 7Be normalized average over 120 days
1.4
1.3
1.2
1.1
1
0.9
0.8
0.7
0.6
0.5
1
2
3
4
5
6
7
8
9
10
11
12
RN monthly 7Be normalized average over 120 days

RN18

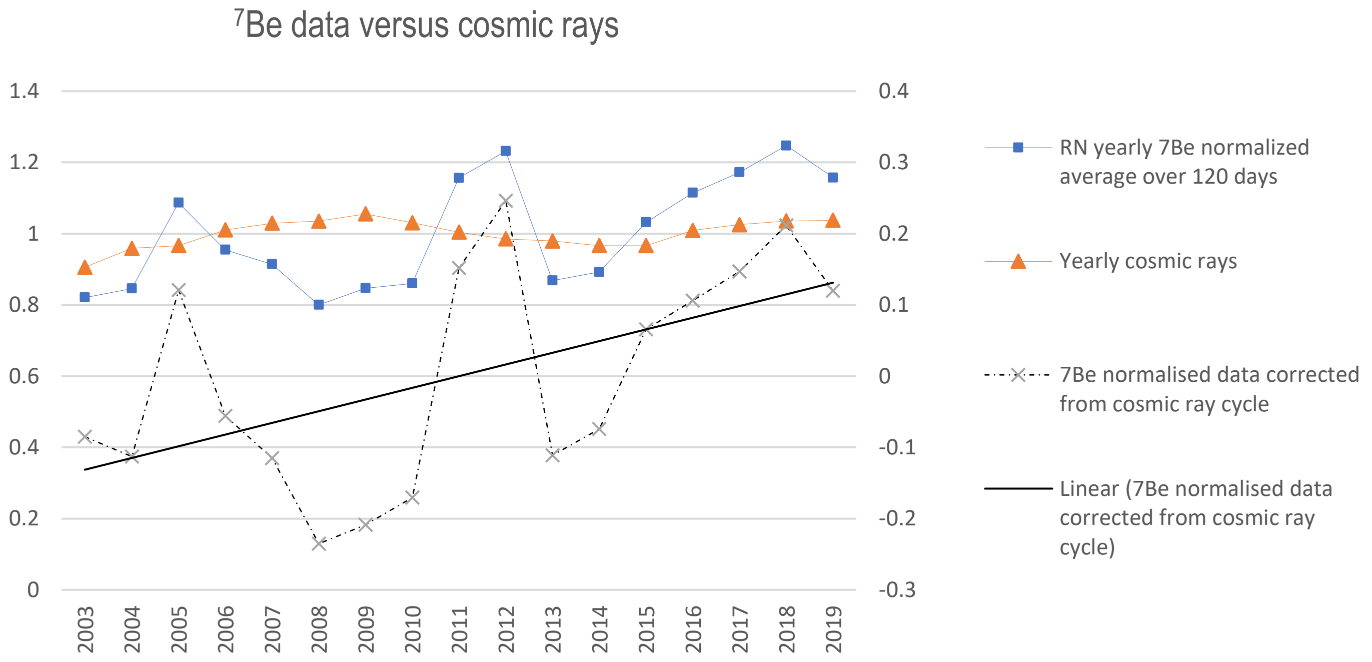

^{7}Be data versus cosmic rays
1.4
1.2
1
0.8
0.6
0.4
0.2
0
0.4
0.3
0.2
0.1
0
-0.1
-0.2
-0.3
2003
2004
2005
2006
2007
2008
2009
2010
2011
2012
2013
2014
2015
2016
2017
2018
2019
RN yearly 7Be normalized average over 120 days
Yearly cosmic rays
7Be normalised data corrected from cosmic ray cycle
Linear (7Be normalised data corrected from cosmic ray cycle)

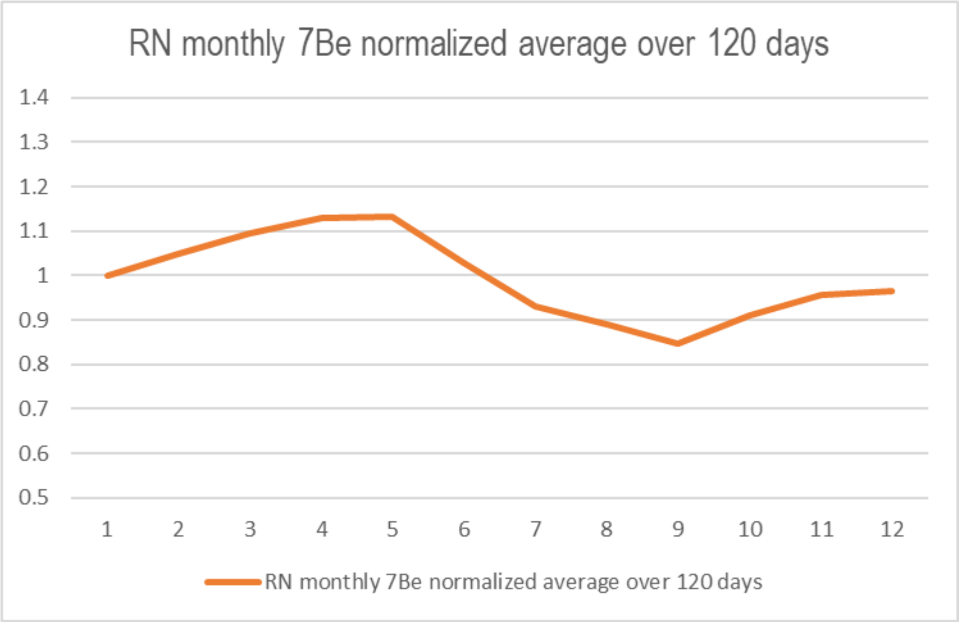

RN monthly 7Be normalized average over 120 days
1.4
1.3
1.2
1.1
1
0.9
0.8
0.7
0.6
0.5
1
2
3
4
5
6
7
8
9
10
11
12
RN monthly 7Be normalized average over 120 days

RN19

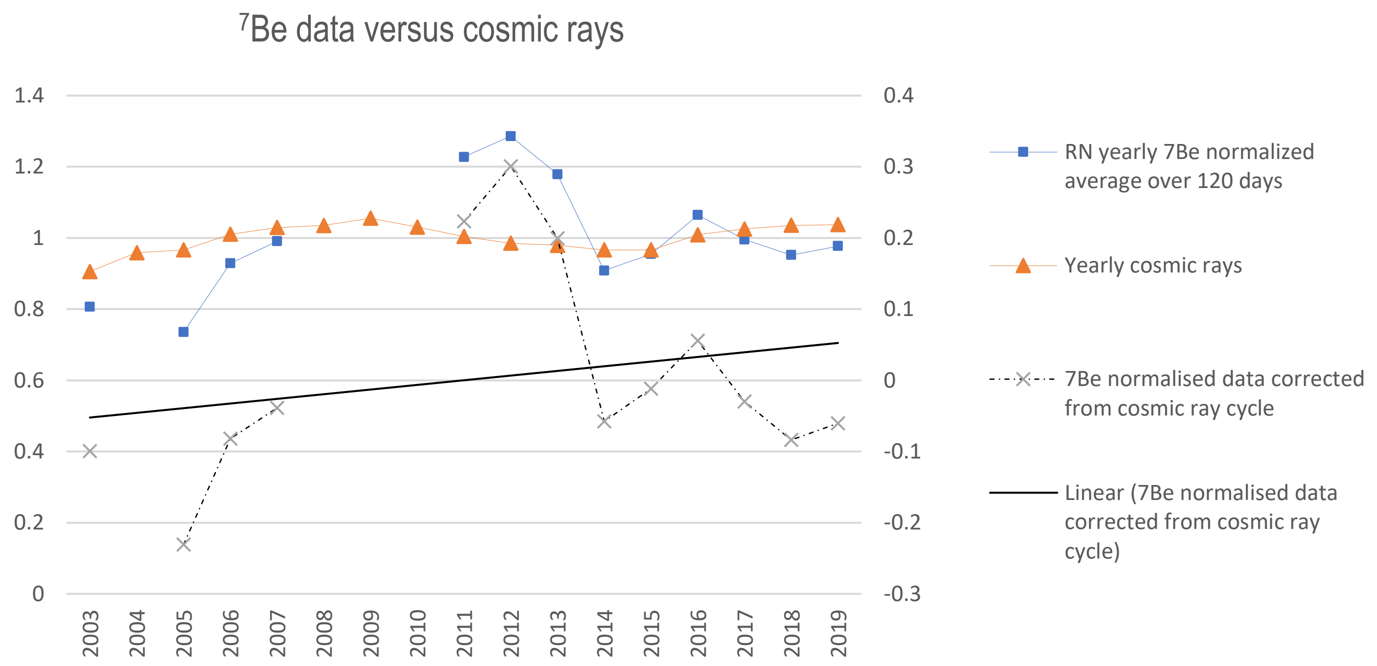

^{7}Be data versus cosmic rays
1.4
1.2
1
0.8
0.6
0.4
0.2
0
0.4
0.3
0.2
0.1
0
-0.1
-0.2
-0.3
2003
2004
2005
2006
2007
2008
2009
2010
2011
2012
2013
2014
2015
2016
2017
2018
2019
RN yearly 7Be normalized average over 120 days
Yearly cosmic rays
7Be normalised data corrected from cosmic ray cycle
Linear (7Be normalised data corrected from cosmic ray cycle)

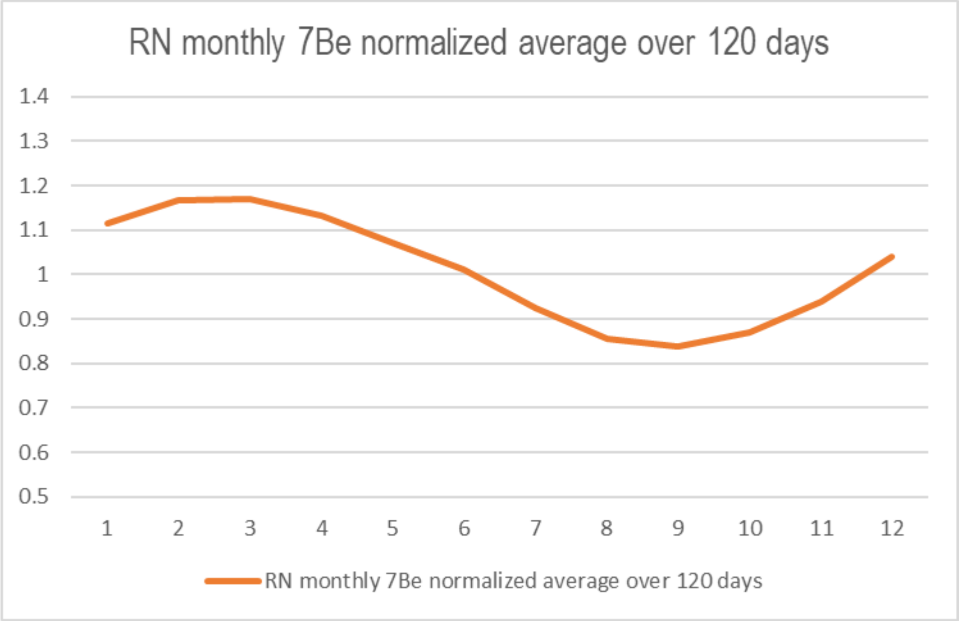

RN monthly 7Be normalized average over 120 days
1.4
1.3
1.2
1.1
1
0.9
0.8
0.7
0.6
0.5
1
2
3
4
5
6
7
8
9
10
11
12
RN monthly 7Be normalized average over 120 days

RN23

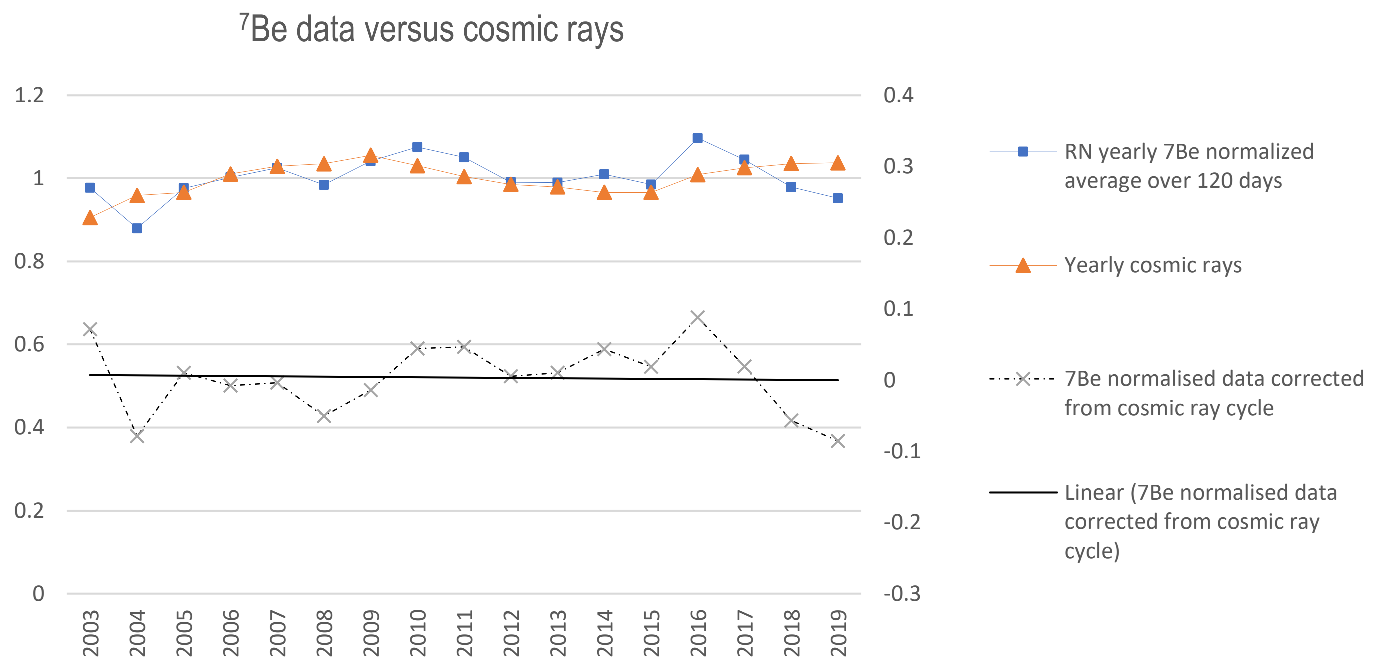

^{7}Be data versus cosmic rays
1.2
1
0.8
0.6
0.4
0.2
0
0.4
0.3
0.2
0.1
0
-0.1
-0.2
-0.3
2003
2004
2005
2006
2007
2008
2009
2010
2011
2012
2013
2014
2015
2016
2017
2018
2019
RN yearly 7Be normalized average over 120 days
Yearly cosmic rays
7Be normalised data corrected from cosmic ray cycle
Linear (7Be normalised data corrected from cosmic ray cycle)

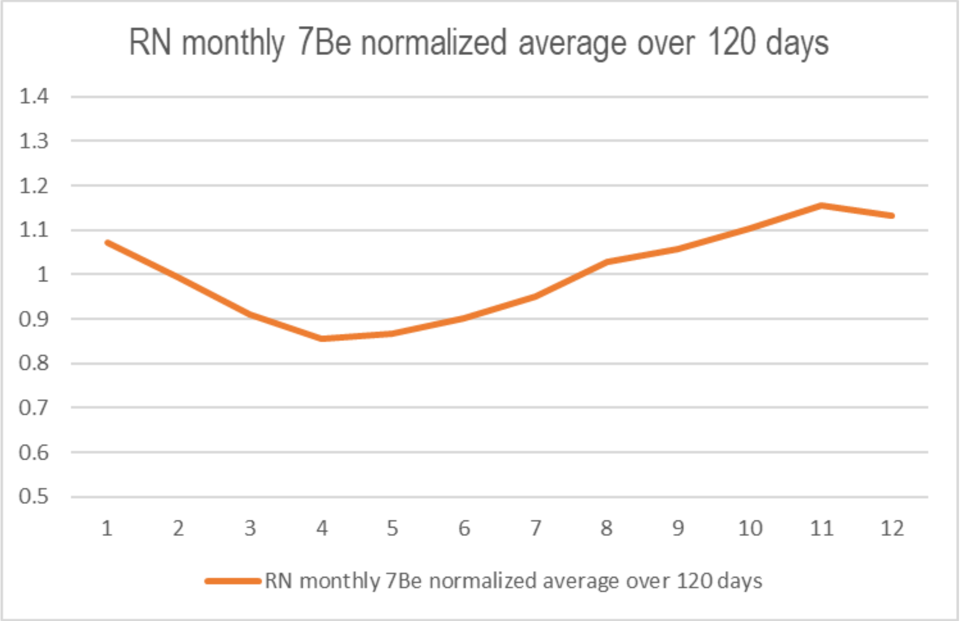

RN monthly 7Be normalized average over 120 days
1.4
1.3
1.2
1.1
1
0.9
0.8
0.7
0.6
0.5
1
2
3
4
5
6
7
8
9
10
11
12
RN monthly 7Be normalized average over 120 days

RN26

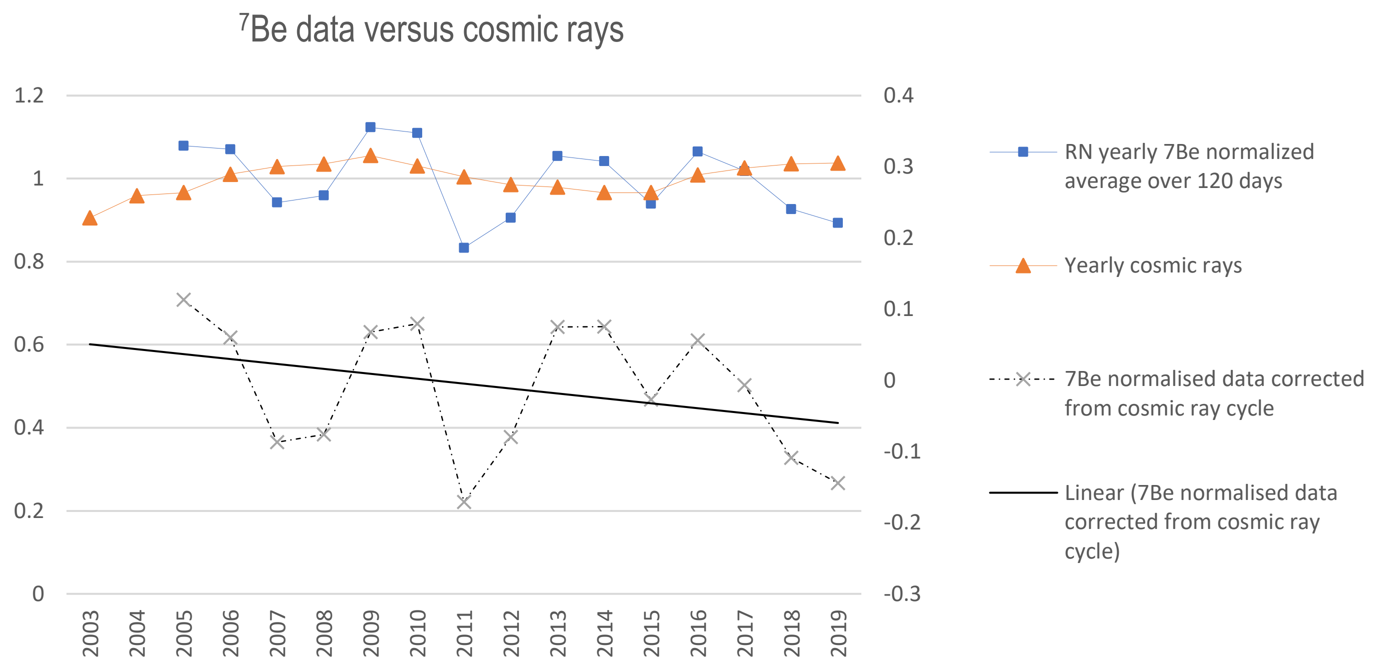

^{7}Be data versus cosmic rays
1.2
1
0.8
0.6
0.4
0.2
0
0.4
0.3
0.2
0.1
0
-0.1
-0.2
-0.3
2003
2004
2005
2006
2007
2008
2009
2010
2011
2012
2013
2014
2015
2016
2017
2018
2019
RN yearly 7Be normalized average over 120 days
Yearly cosmic rays
7Be normalised data corrected from cosmic ray cycle
Linear (7Be normalised data corrected from cosmic ray cycle)

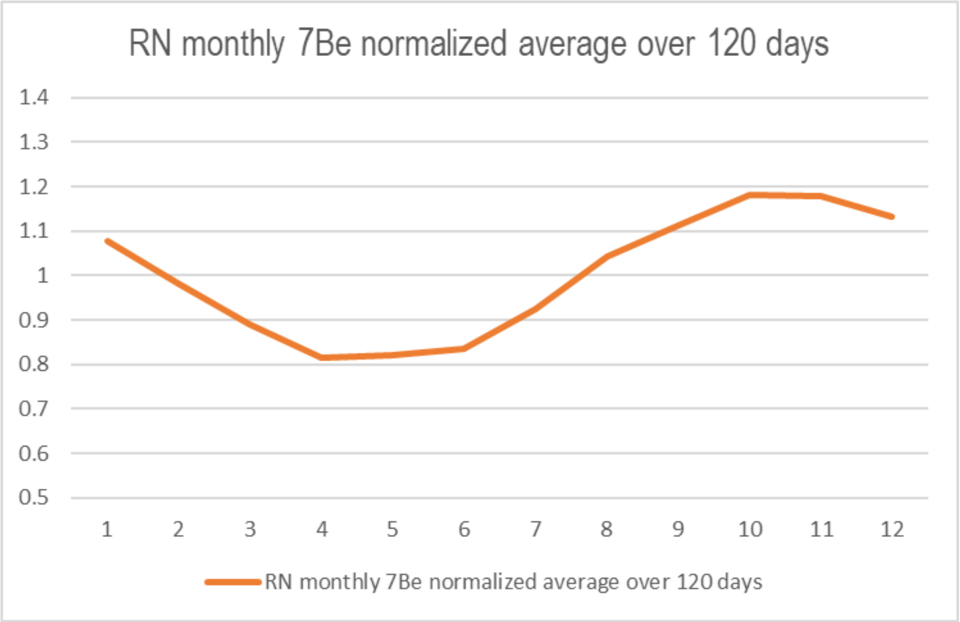

RN monthly 7Be normalized average over 120 days
1.4
1.3
1.2
1.1
1
0.9
0.8
0.7
0.6
0.5
1
2
3
4
5
6
7
8
9
10
11
12
RN monthly 7Be normalized average over 120 days

RN27

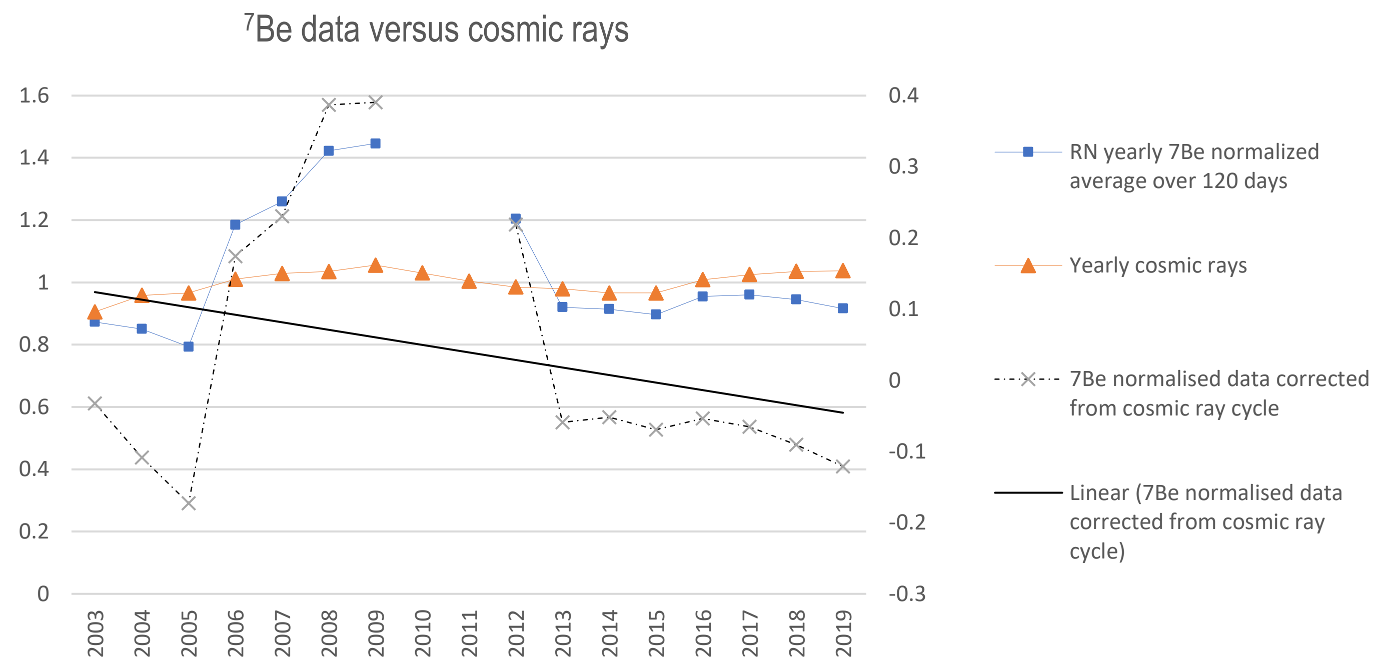

^{7}Be data versus cosmic rays
1.6
1.4
1.2
1
0.8
0.6
0.4
0.2
0
0.4
0.3
0.2
0.1
0
-0.1
-0.2
-0.3
2003
2004
2005
2006
2007
2008
2009
2010
2011
2012
2013
2014
2015
2016
2017
2018
2019
RN yearly 7Be normalized average over 120 days
Yearly cosmic rays
7Be normalised data corrected from cosmic ray cycle
Linear (7Be normalised data corrected from cosmic ray cycle)

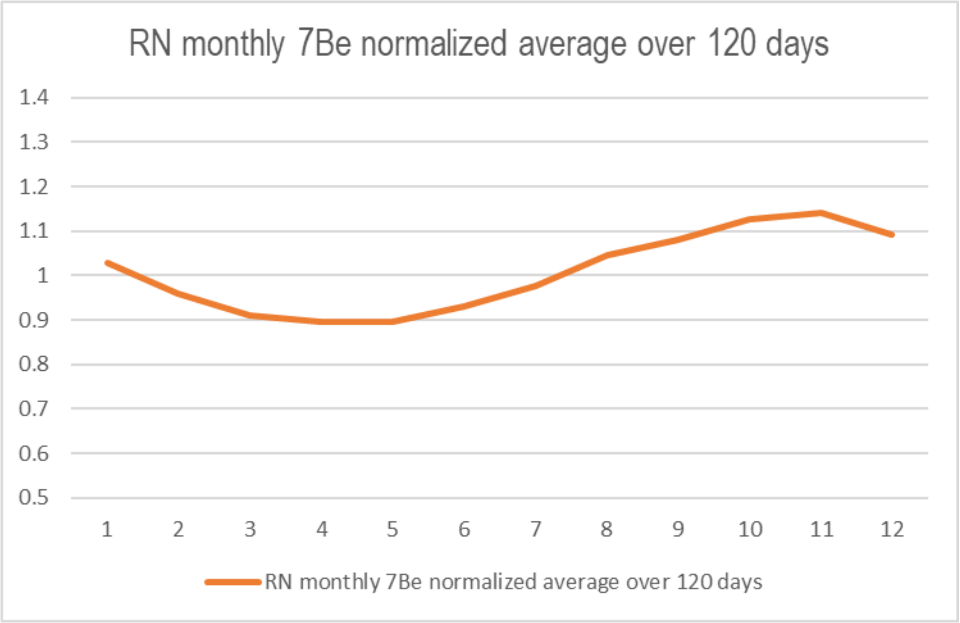

RN monthly 7Be normalized average over 120 days
1.4
1.3
1.2
1.1
1
0.9
0.8
0.7
0.6
0.5
1
2
3
4
5
6
7
8
9
10
11
12
RN monthly 7Be normalized average over 120 days

RN28

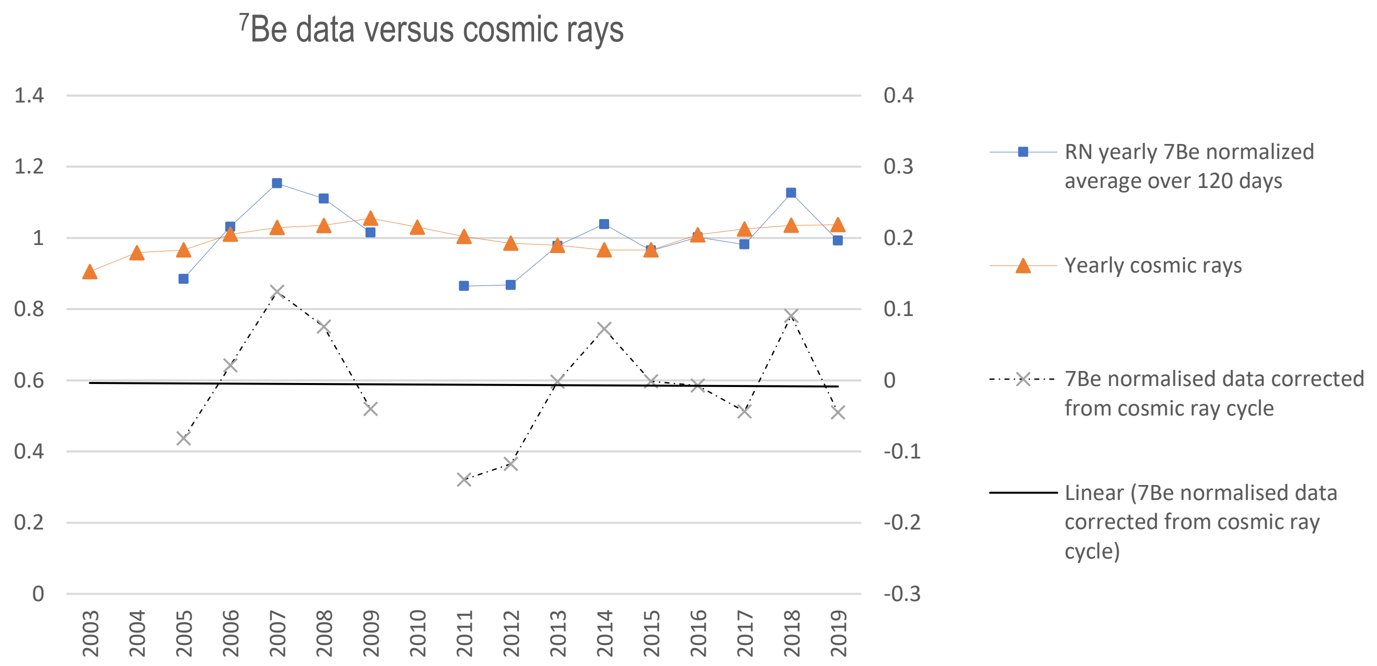

^{7}Be data versus cosmic rays
1.4
1.2
1
0.8
0.6
0.4
0.2
0
0.4
0.3
0.2
0.1
0
-0.1
-0.2
-0.3
2003
2004
2005
2006
2007
2008
2009
2010
2011
2012
2013
2014
2015
2016
2017
2018
2019
RN yearly 7Be normalized average over 120 days
Yearly cosmic rays
7Be normalised data corrected from cosmic ray cycle
Linear (7Be normalised data corrected from cosmic ray cycle)

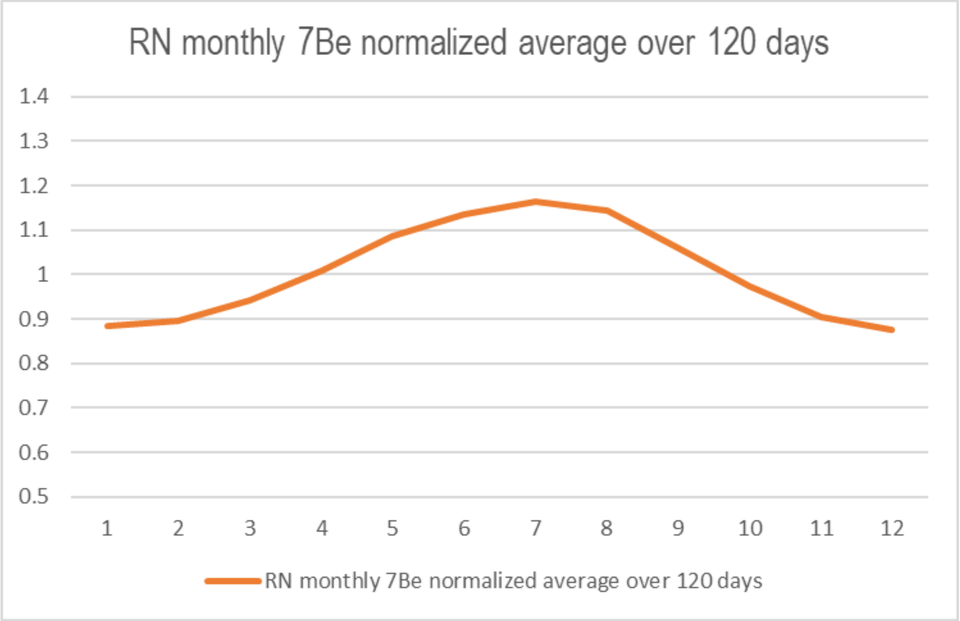

RN monthly 7Be normalized average over 120 days
1.4
1.3
1.2
1.1
1
0.9
0.8
0.7
0.6
0.5
1
2
3
4
5
6
7
8
9
10
11
12
RN monthly 7Be normalized average over 120 days

RN29

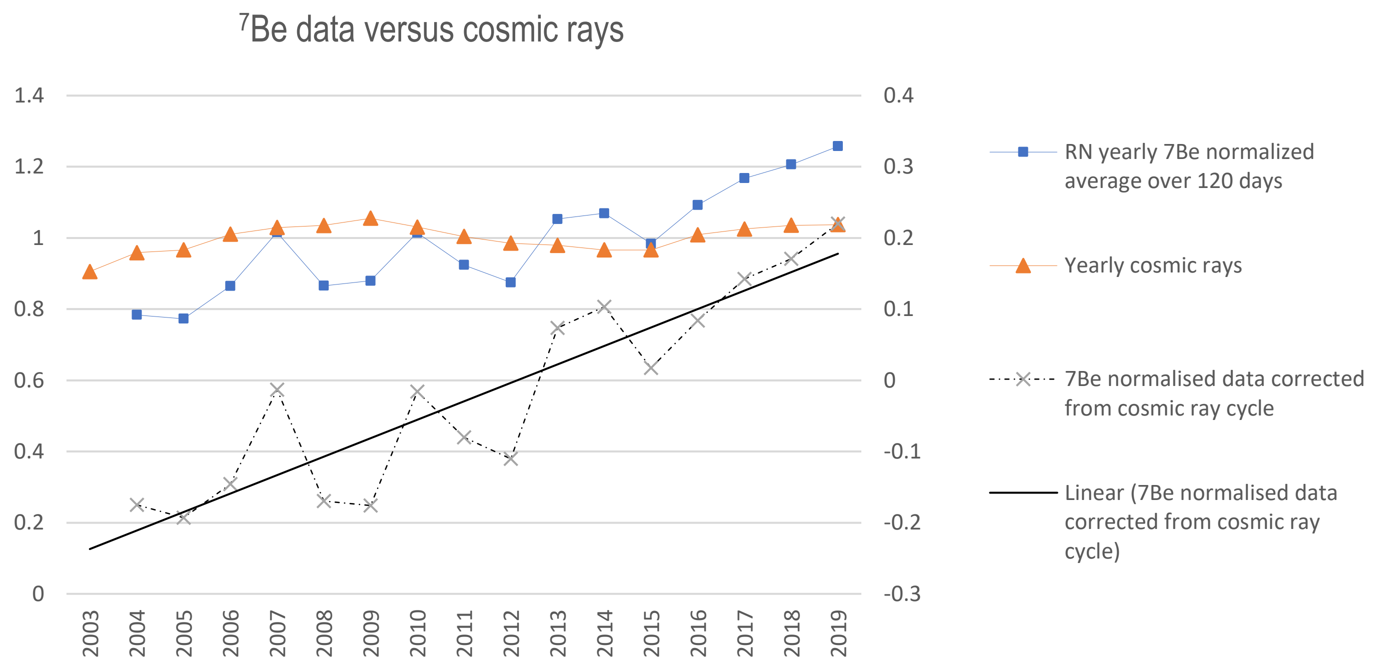

7Be data versus cosmic rays
1.4
1.2
1
0.8
0.6
0.4
0.2
0
0.4
0.3
0.2
0.1
0
-0.1
-0.2
-0.3
2003
2004
2005
2006
2007
2008
2009
2010
2011
2012
2013
2014
2015
2016
2017
2018
2019
RN yearly 7Be normalized average over 120 days
Yearly cosmic rays
7Be normalised data corrected from cosmic ray cycle
Linear (7Be normalised data corrected from cosmic ray cycle)

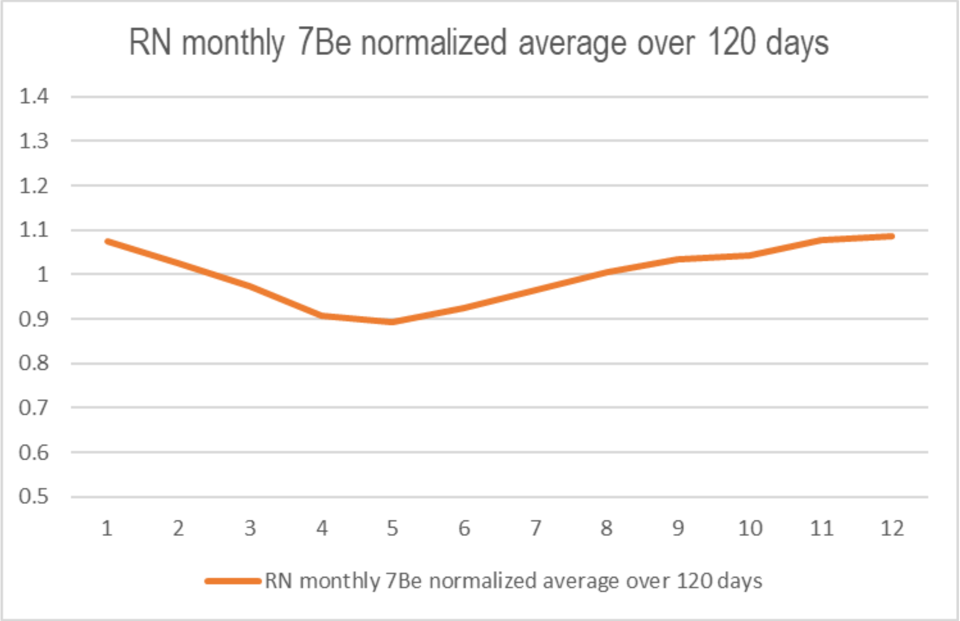

RN monthly 7Be normalized average over 120 days
1.4
1.3
1.2
1.1
1
0.9
0.8
0.7
0.6
0.5
1
2
3
4
5
6
7
8
9
10
11
12
RN monthly 7Be normalized average over 120 days

RN30

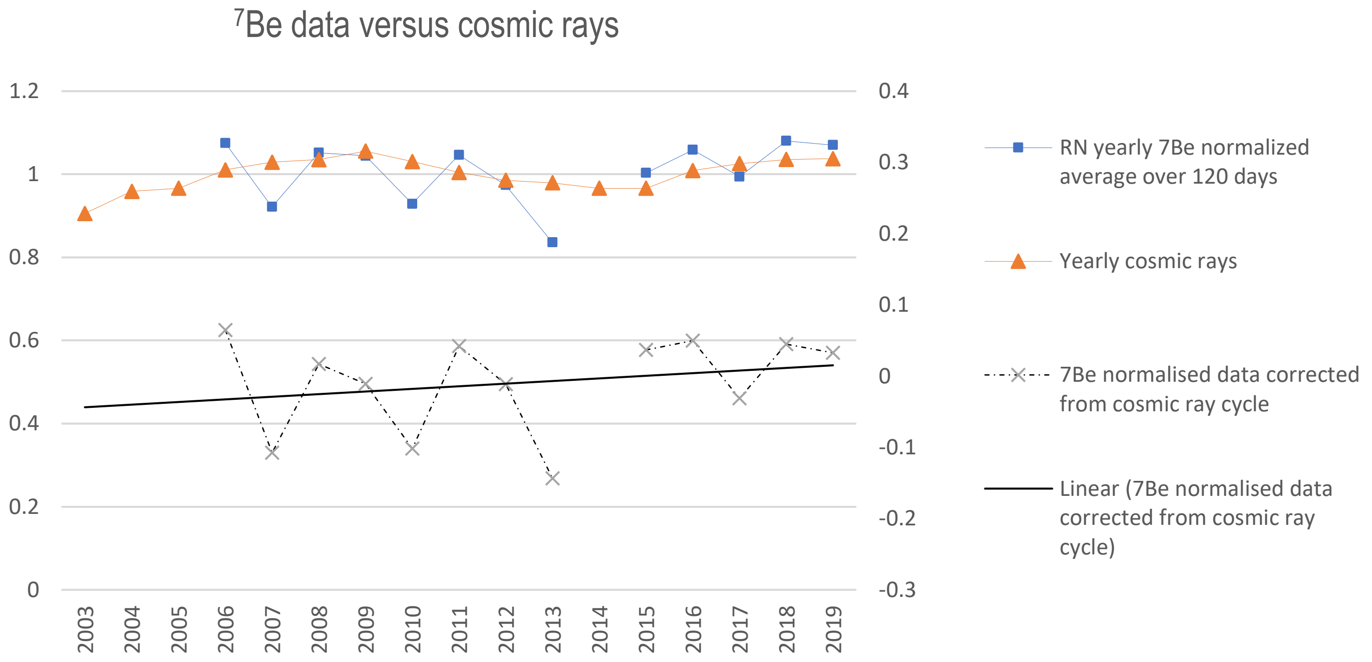
^{7}Be data versus cosmic rays
1.2
1
0.8
0.6
0.4
0.2
0
0.4
0.3
0.2
0.1
0
-0.1
-0.2
-0.3
2003
2004
2005
2006
2007
2008
2009
2010
2011
2012
2013
2014
2015
2016
2017
2018
2019
RN yearly 7Be normalized average over 120 days
Yearly cosmic rays
7Be normalised data corrected from cosmic ray cycle
Linear (7Be normalised data corrected from cosmic ray cycle)

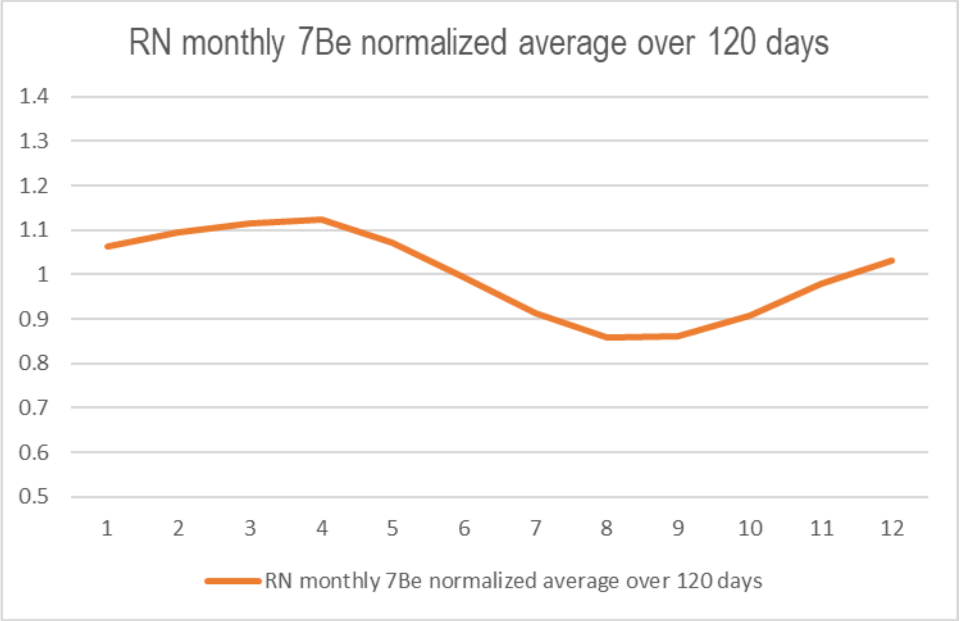
RN monthly 7Be normalized average over 120 days
1.4
1.3
1.2
1.1
1
0.9
0.8
0.7
0.6
0.5
1
2
3
4
5
6
7
8
9
10
11
12
RN monthly 7Be normalized average over 120 days

RN31

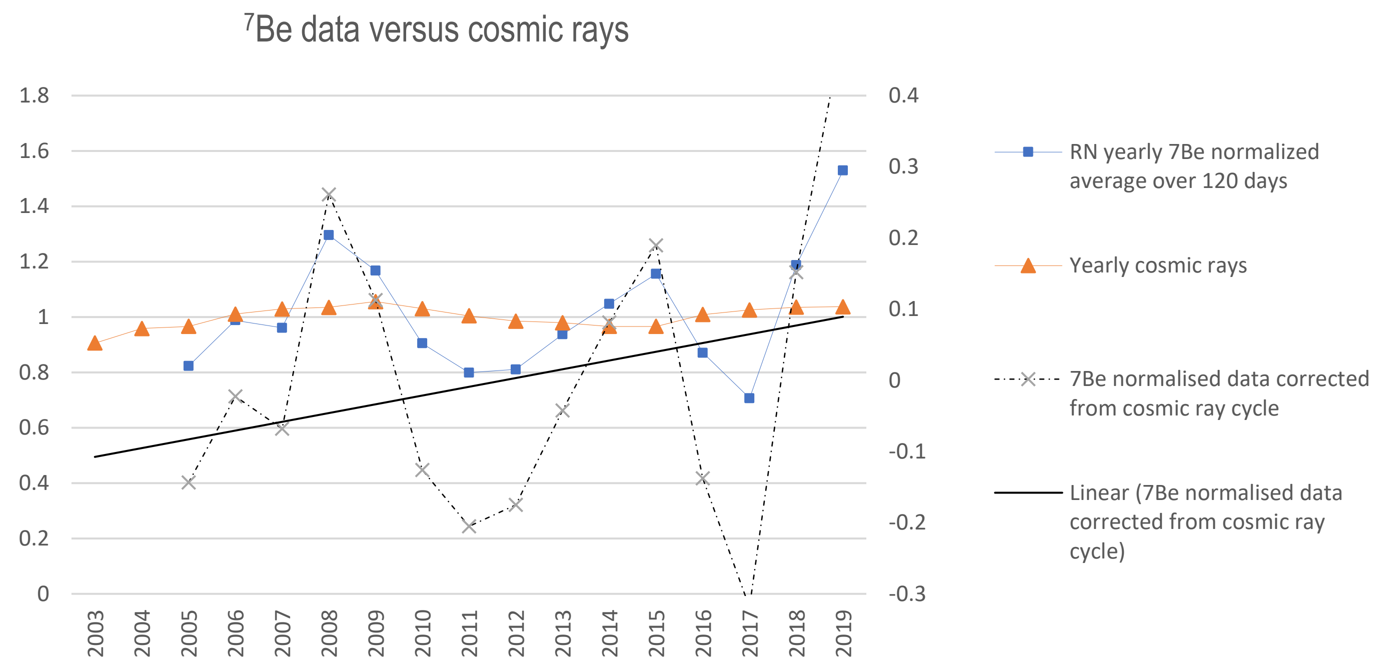

^{7}Be data versus cosmic rays
1.8
1.6
1.4
1.2
1
0.8
0.6
0.4
0.2
0
0.4
0.3
0.2
0.1
0
-0.1
-0.2
-0.3
2003 2004 2005 2006 2007 2008 2009 2010 2011 2012 2013 2014 2015 2016 2017 2018 2019
RN yearly 7Be normalized average over 120 days
Yearly cosmic rays
7Be normalised data corrected from cosmic ray cycle
Linear (7Be normalised data corrected from cosmic ray cycle)

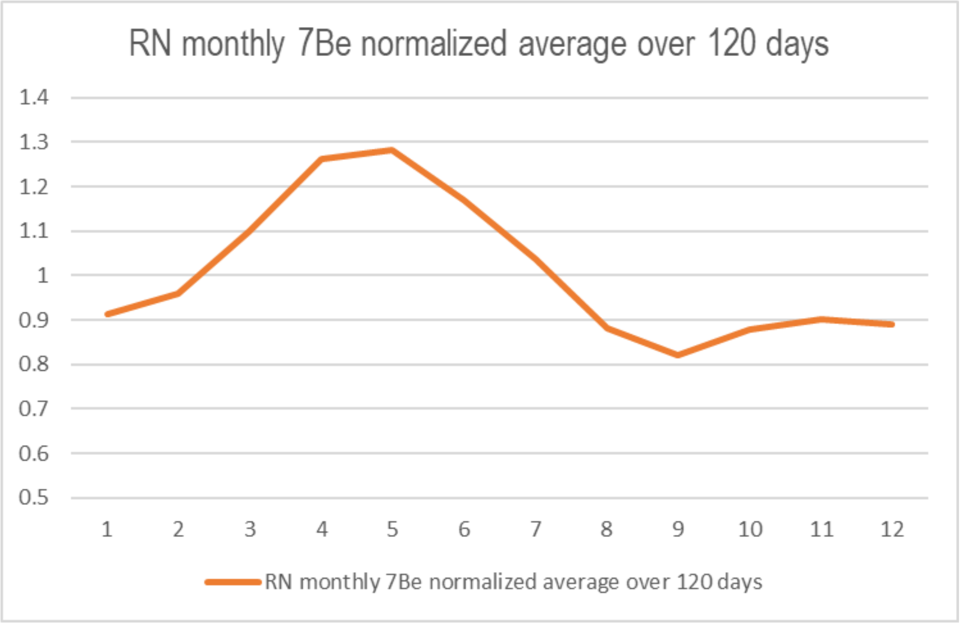

RN monthly 7Be normalized average over 120 days
1.4
1.3
1.2
1.1
1
0.9
0.8
0.7
0.6
0.5
1 2 3 4 5 6 7 8 9 10 11 12
RN monthly 7Be normalized average over 120 days

RN33

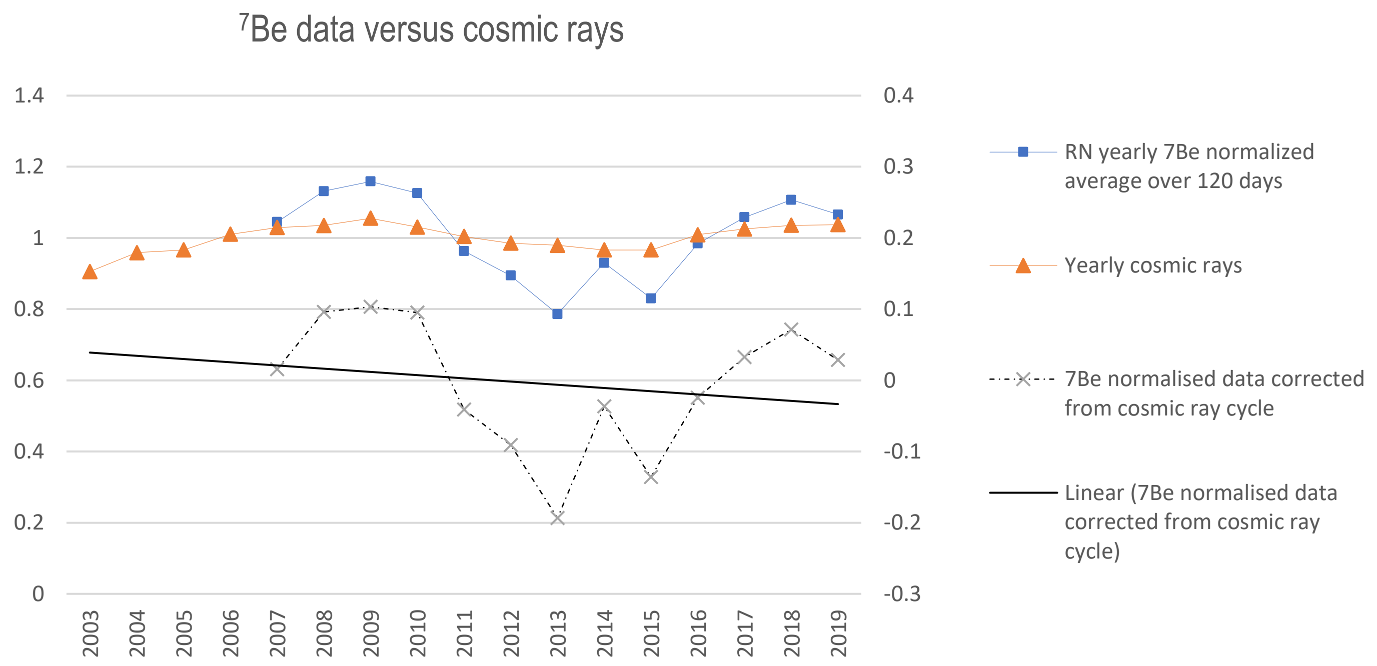

^{7}Be data versus cosmic rays
1.4
1.2
1
0.8
0.6
0.4
0.2
0
0.4
0.3
0.2
0.1
0
-0.1
-0.2
-0.3
2003
2004
2005
2006
2007
2008
2009
2010
2011
2012
2013
2014
2015
2016
2017
2018
2019
RN yearly 7Be normalized average over 120 days
Yearly cosmic rays
7Be normalised data corrected from cosmic ray cycle
Linear (7Be normalised data corrected from cosmic ray cycle)

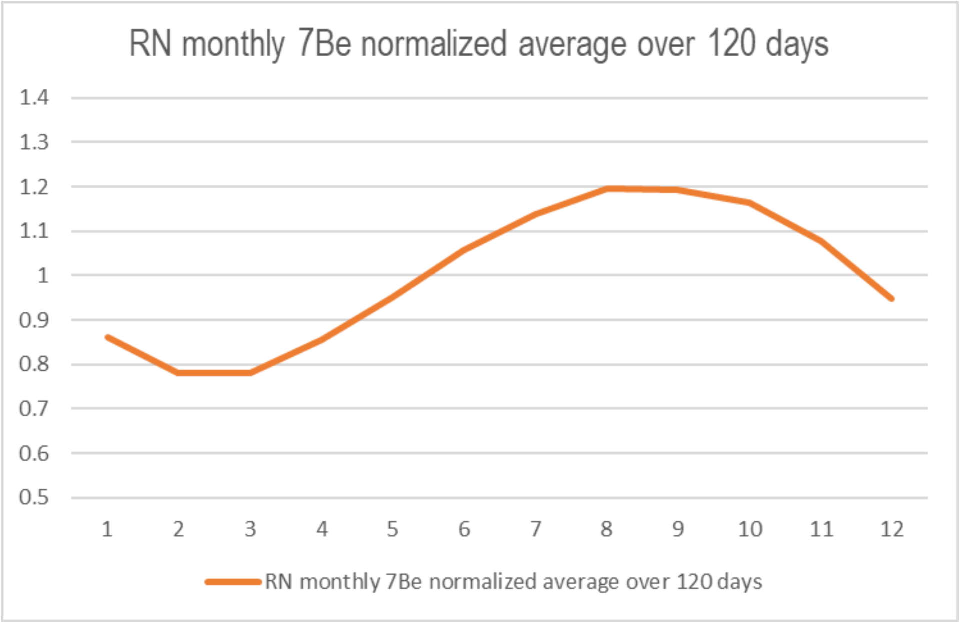

RN monthly 7Be normalized average over 120 days
1.4
1.3
1.2
1.1
1
0.9
0.8
0.7
0.6
0.5
1
2
3
4
5
6
7
8
9
10
11
12
RN monthly 7Be normalized average over 120 days

RN34

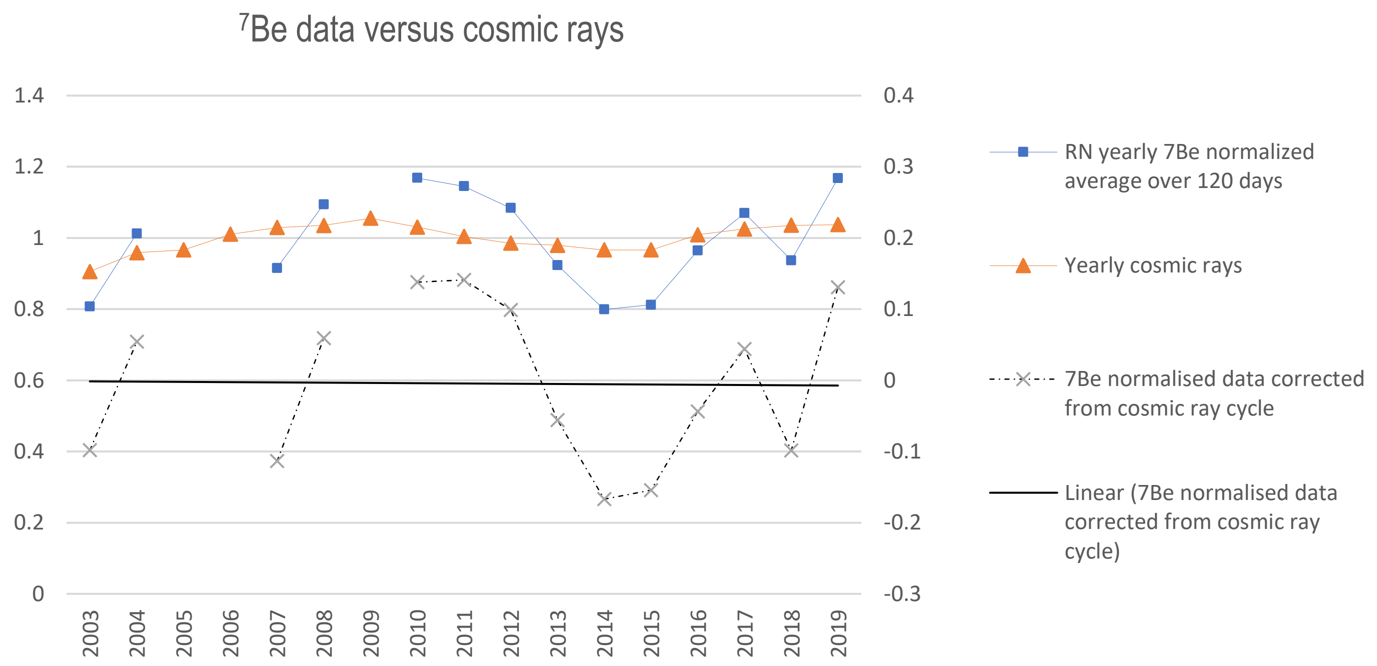
^{7}Be data versus cosmic rays
1.4
1.2
1
0.8
0.6
0.4
0.2
0
0.4
0.3
0.2
0.1
0
-0.1
-0.2
-0.3
2003
2004
2005
2006
2007
2008
2009
2010
2011
2012
2013
2014
2015
2016
2017
2018
2019
RN yearly 7Be normalized average over 120 days
Yearly cosmic rays
7Be normalised data corrected from cosmic ray cycle
Linear (7Be normalised data corrected from cosmic ray cycle)

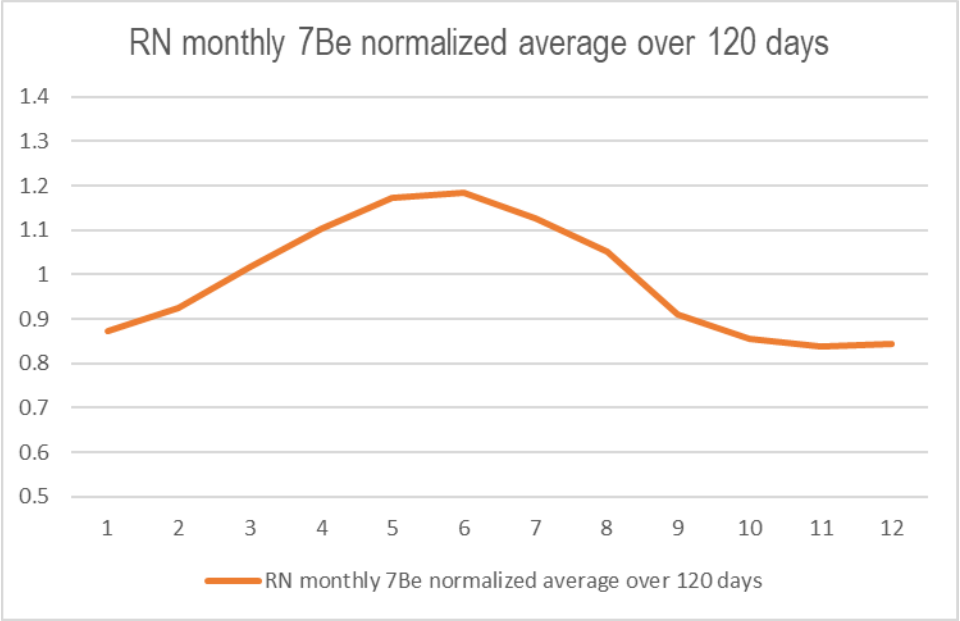
RN monthly 7Be normalized average over 120 days
1.4
1.3
1.2
1.1
1
0.9
0.8
0.7
0.6
0.5
1
2
3
4
5
6
7
8
9
10
11
12
RN monthly 7Be normalized average over 120 days

RN37

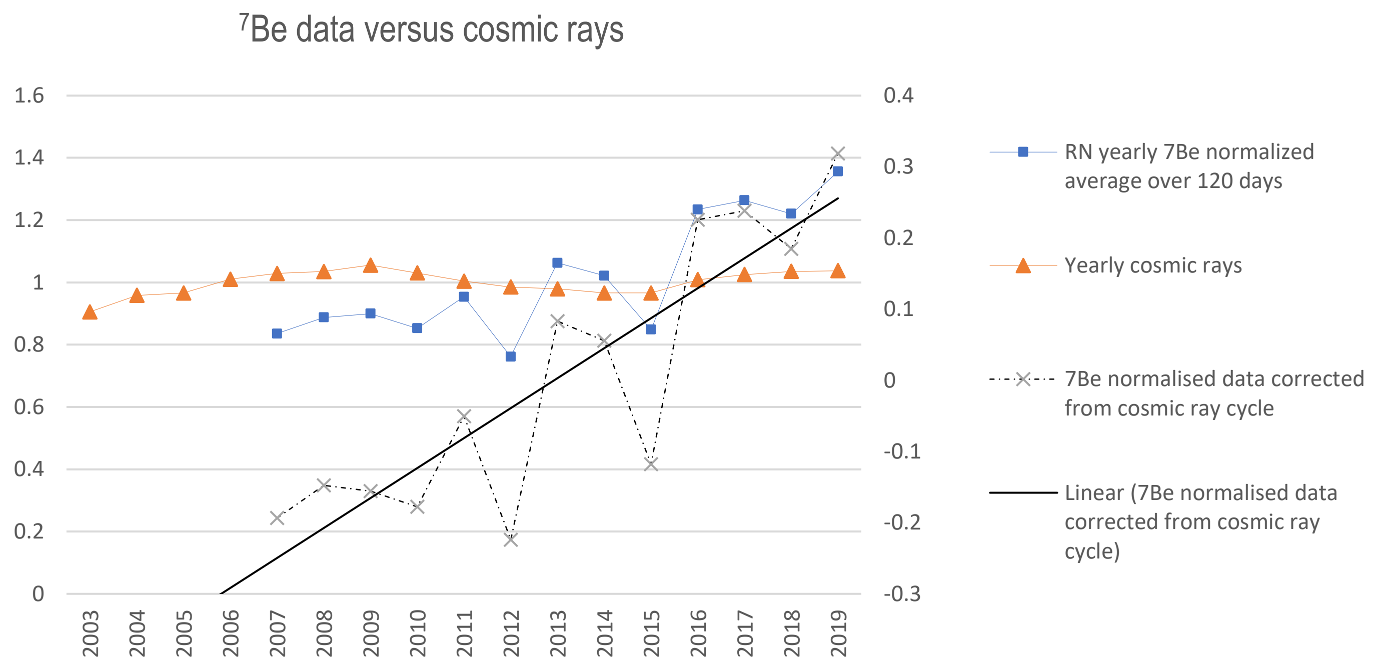

^{7}Be data versus cosmic rays
1.6
1.4
1.2
1
0.8
0.6
0.4
0.2
0
0.4
0.3
0.2
0.1
0
-0.1
-0.2
-0.3
2003
2004
2005
2006
2007
2008
2009
2010
2011
2012
2013
2014
2015
2016
2017
2018
2019
RN yearly 7Be normalized average over 120 days
Yearly cosmic rays
7Be normalised data corrected from cosmic ray cycle
Linear (7Be normalised data corrected from cosmic ray cycle)

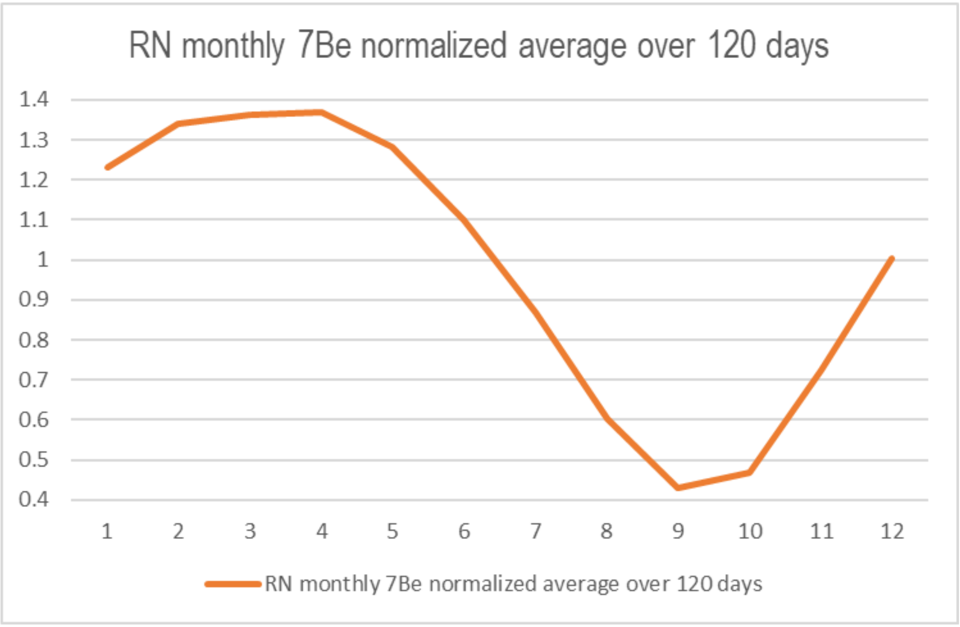

RN monthly 7Be normalized average over 120 days
1.4
1.3
1.2
1.1
1
0.9
0.8
0.7
0.6
0.5
0.4
1
2
3
4
5
6
7
8
9
10
11
12
RN monthly 7Be normalized average over 120 days

RN38

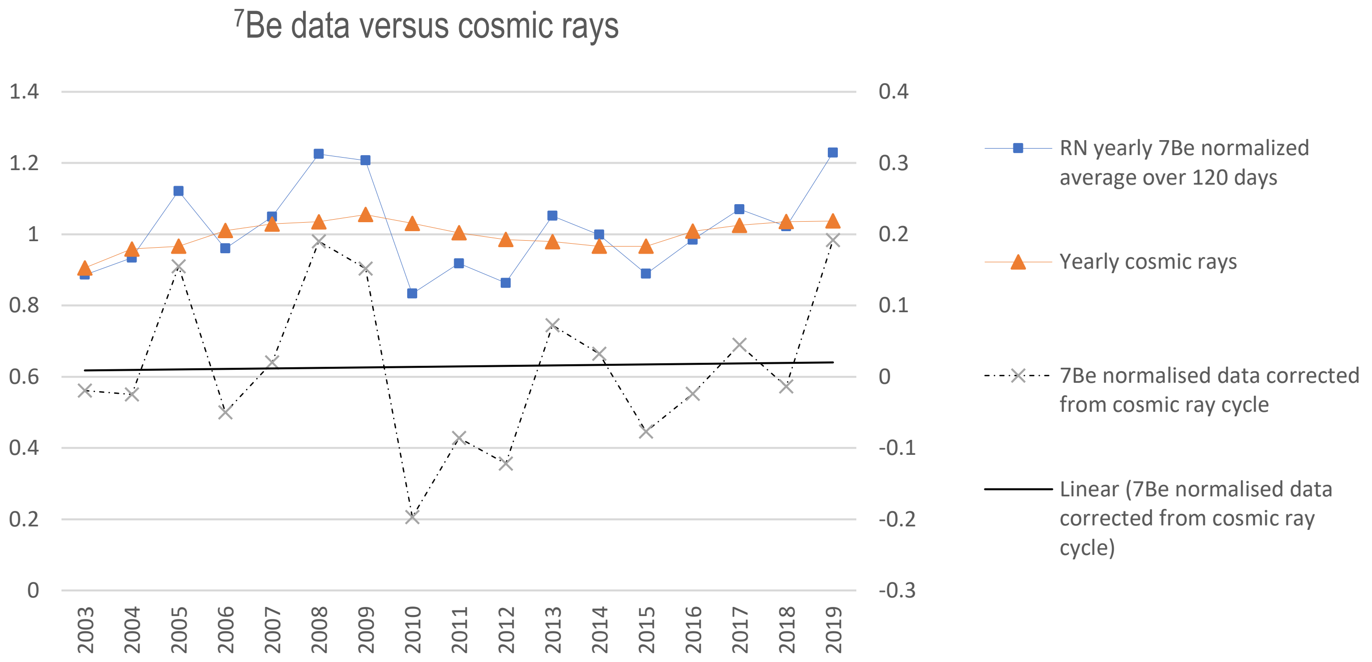

^{7}Be data versus cosmic rays
1.4
1.2
1
0.8
0.6
0.4
0.2
0
0.4
0.3
0.2
0.1
0
-0.1
-0.2
-0.3
2003
2004
2005
2006
2007
2008
2009
2010
2011
2012
2013
2014
2015
2016
2017
2018
2019
RN yearly 7Be normalized average over 120 days
Yearly cosmic rays
7Be normalised data corrected from cosmic ray cycle
Linear (7Be normalised data corrected from cosmic ray cycle)

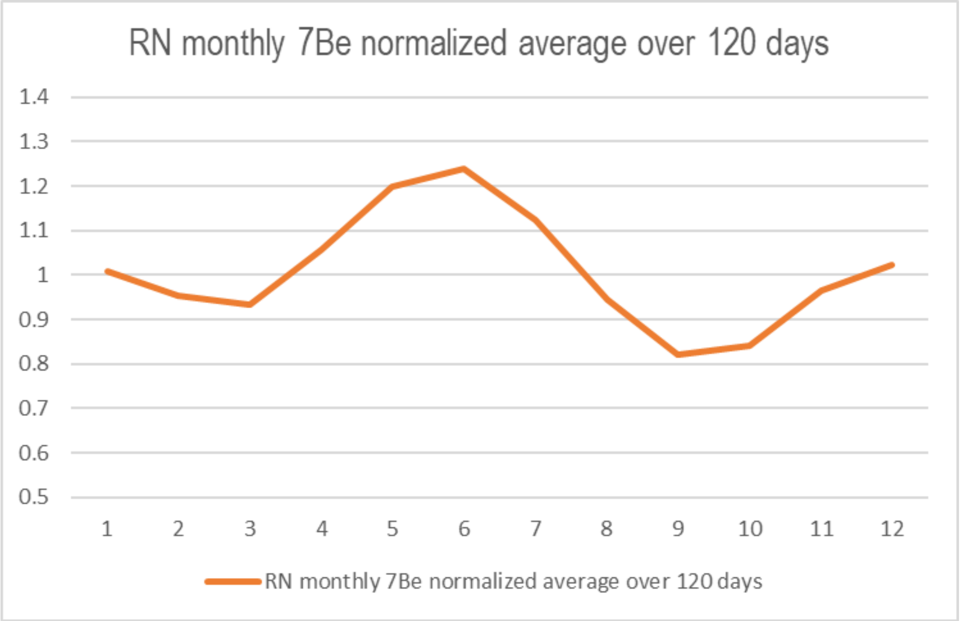

RN monthly 7Be normalized average over 120 days
1.4
1.3
1.2
1.1
1
0.9
0.8
0.7
0.6
0.5
1
2
3
4
5
6
7
8
9
10
11
12
RN monthly 7Be normalized average over 120 days

RN39

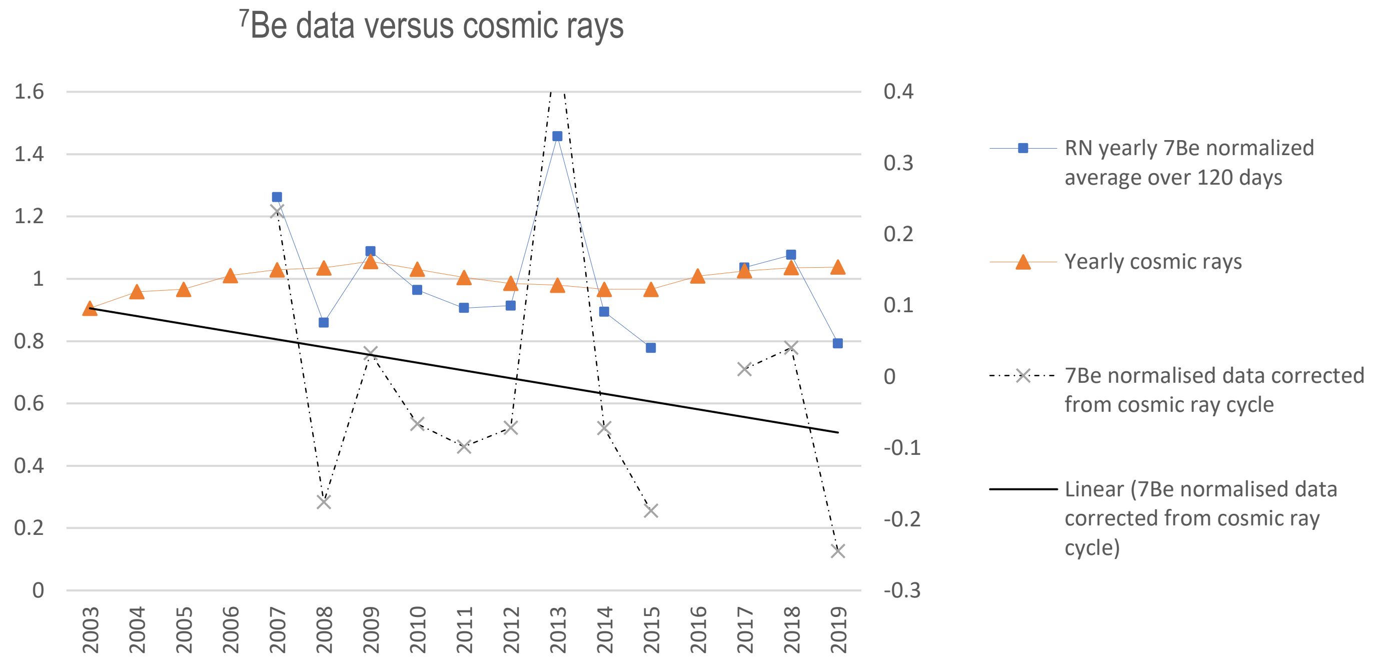

^{7}Be data versus cosmic rays
1.6
1.4
1.2
1
0.8
0.6
0.4
0.2
0
0.4
0.3
0.2
0.1
0
-0.1
-0.2
-0.3
2003
2004
2005
2006
2007
2008
2009
2010
2011
2012
2013
2014
2015
2016
2017
2018
2019
RN yearly 7Be normalized average over 120 days
Yearly cosmic rays
7Be normalised data corrected from cosmic ray cycle
Linear (7Be normalised data corrected from cosmic ray cycle)

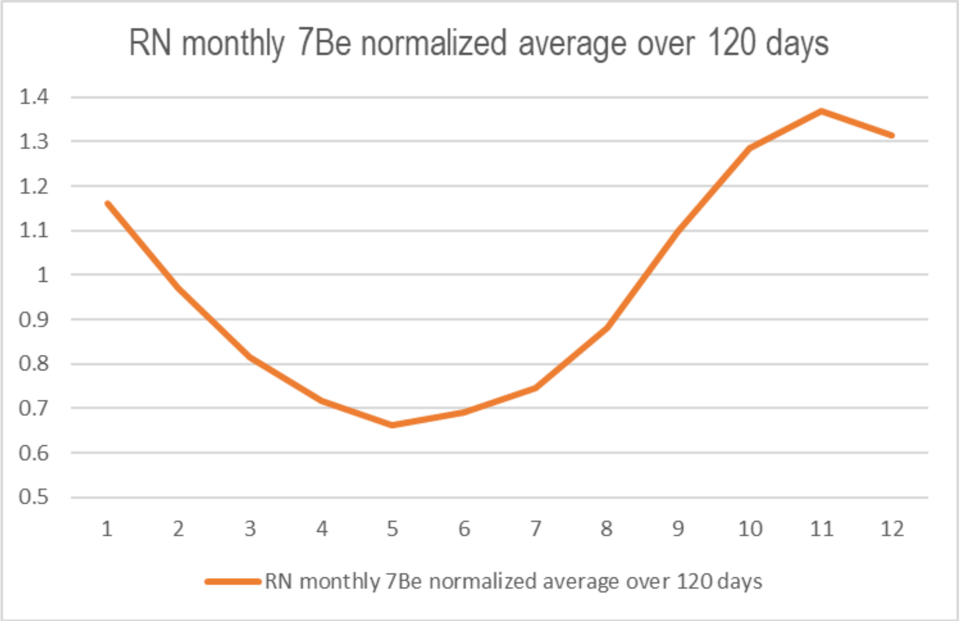

RN monthly 7Be normalized average over 120 days
1.4
1.3
1.2
1.1
1
0.9
0.8
0.7
0.6
0.5
1
2
3
4
5
6
7
8
9
10
11
12
RN monthly 7Be normalized average over 120 days

RN40

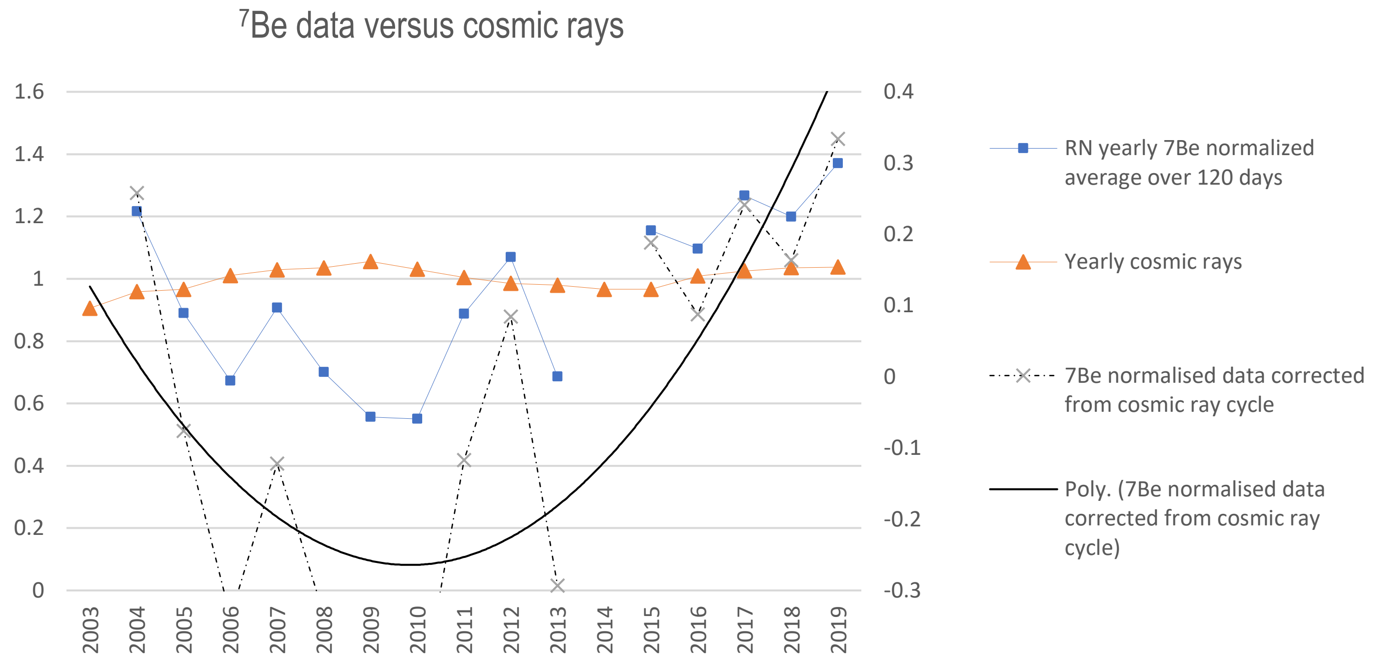

^{7}Be data versus cosmic rays
1.6
1.4
1.2
1
0.8
0.6
0.4
0.2
0
0.4
0.3
0.2
0.1
0
-0.1
-0.2
-0.3
2003
2004
2005
2006
2007
2008
2009
2010
2011
2012
2013
2014
2015
2016
2017
2018
2019
RN yearly 7Be normalized average over 120 days
Yearly cosmic rays
7Be normalised data corrected from cosmic ray cycle
Poly. (7Be normalised data corrected from cosmic ray cycle)

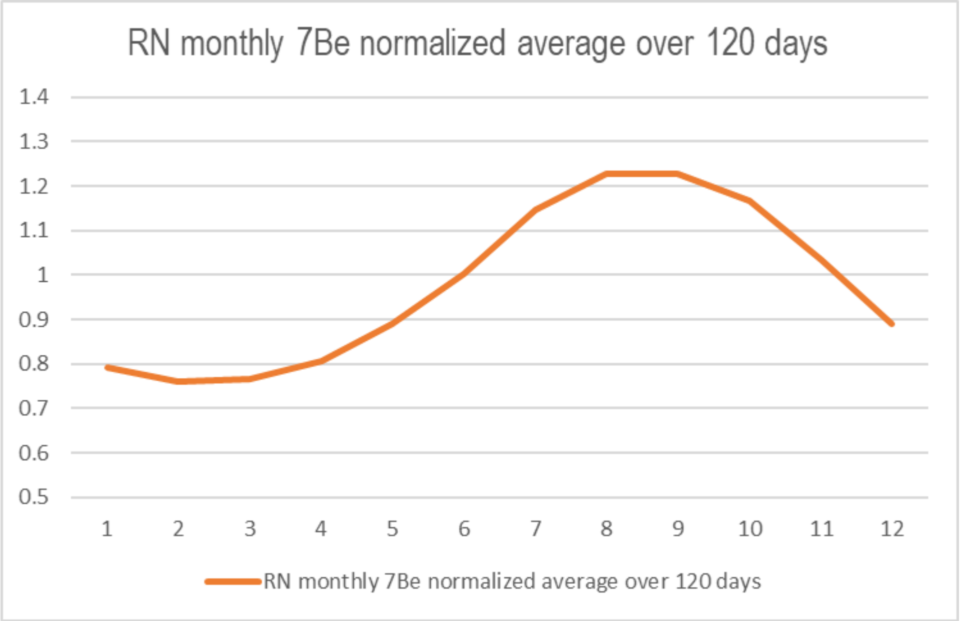

RN monthly 7Be normalized average over 120 days
1.4
1.3
1.2
1.1
1
0.9
0.8
0.7
0.6
0.5
1
2
3
4
5
6
7
8
9
10
11
12
RN monthly 7Be normalized average over 120 days

RN42

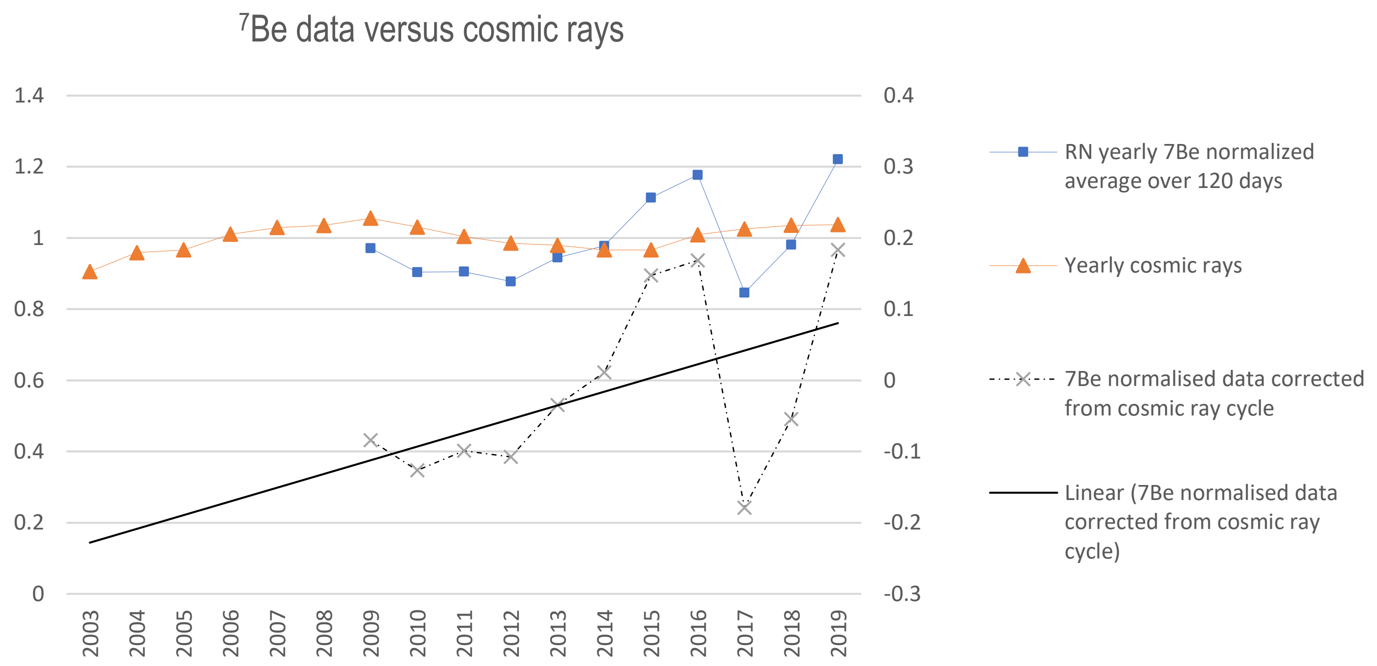

^{7}Be data versus cosmic rays
1.4
1.2
1
0.8
0.6
0.4
0.2
0
0.4
0.3
0.2
0.1
0
-0.1
-0.2
-0.3
2003
2004
2005
2006
2007
2008
2009
2010
2011
2012
2013
2014
2015
2016
2017
2018
2019
RN yearly 7Be normalized average over 120 days
Yearly cosmic rays
7Be normalised data corrected from cosmic ray cycle
Linear (7Be normalised data corrected from cosmic ray cycle)

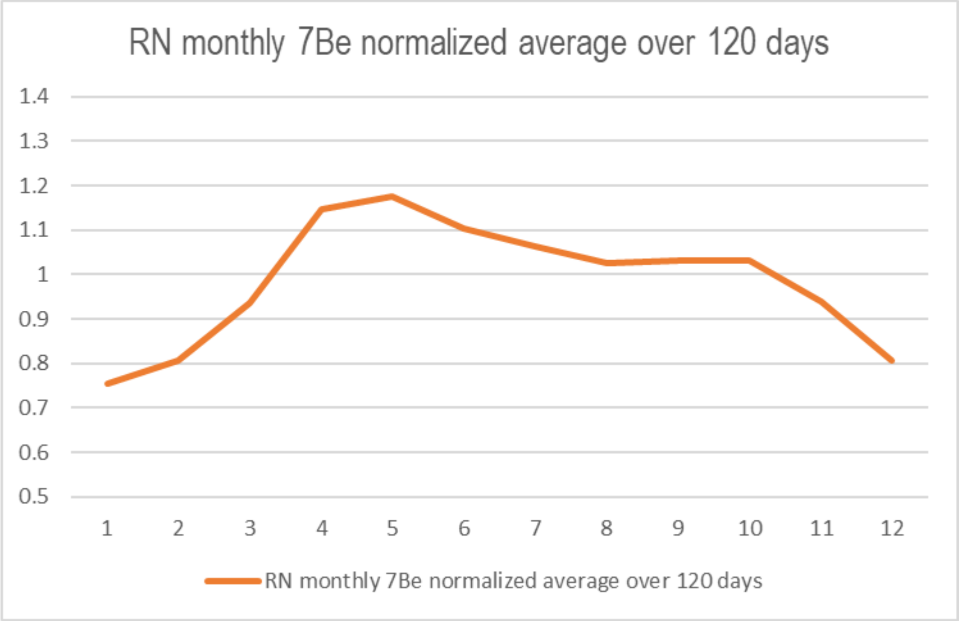

RN monthly 7Be normalized average over 120 days
1.4
1.3
1.2
1.1
1
0.9
0.8
0.7
0.6
0.5
1
2
3
4
5
6
7
8
9
10
11
12
RN monthly 7Be normalized average over 120 days

RN43

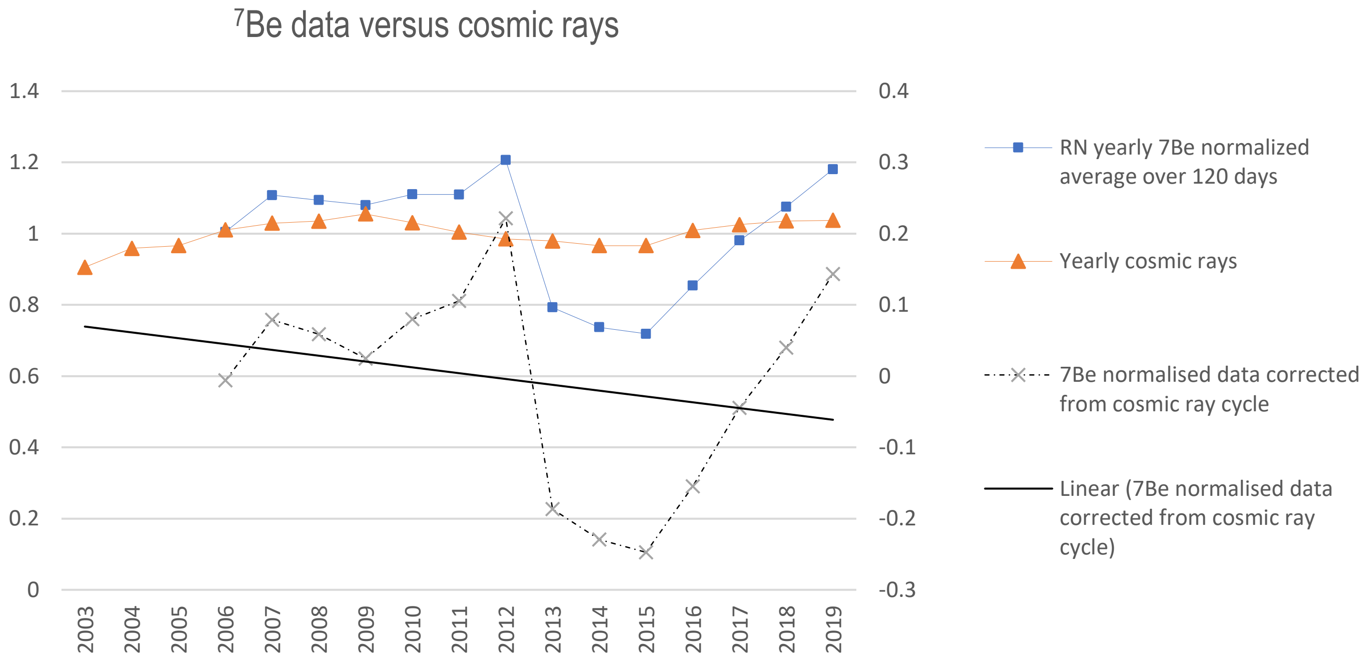

7Be data versus cosmic rays
1.4
1.2
1
0.8
0.6
0.4
0.2
0
0.4
0.3
0.2
0.1
0
-0.1
-0.2
-0.3
2003
2004
2005
2006
2007
2008
2009
2010
2011
2012
2013
2014
2015
2016
2017
2018
2019
RN yearly 7Be normalized average over 120 days
Yearly cosmic rays
7Be normalised data corrected from cosmic ray cycle
Linear (7Be normalised data corrected from cosmic ray cycle)

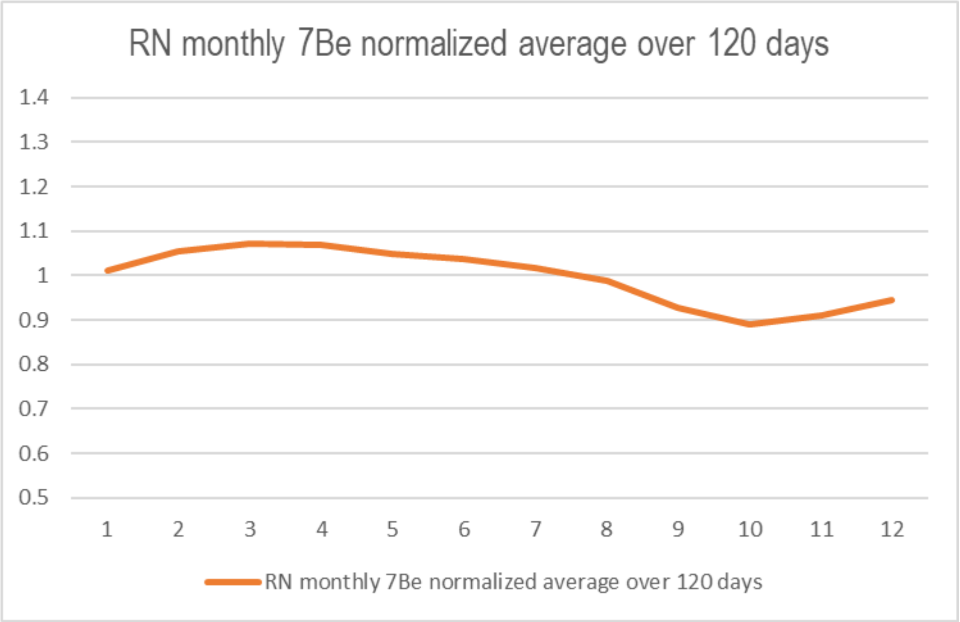

RN monthly 7Be normalized average over 120 days
1.4
1.3
1.2
1.1
1
0.9
0.8
0.7
0.6
0.5
1
2
3
4
5
6
7
8
9
10
11
12
RN monthly 7Be normalized average over 120 days

RN44

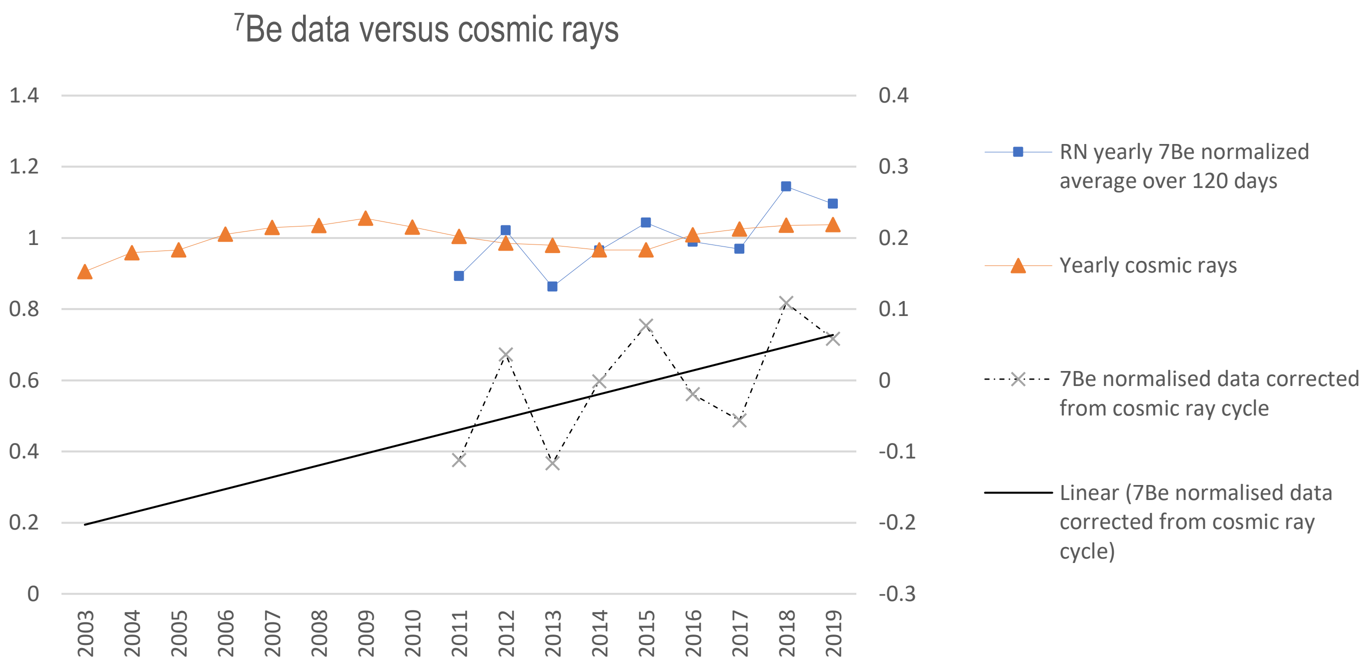
7Be data versus cosmic rays
1.4
1.2
1
0.8
0.6
0.4
0.2
0
0.4
0.3
0.2
0.1
0
-0.1
-0.2
-0.3
2003
2004
2005
2006
2007
2008
2009
2010
2011
2012
2013
2014
2015
2016
2017
2018
2019
RN yearly 7Be normalized average over 120 days
Yearly cosmic rays
7Be normalised data corrected from cosmic ray cycle
Linear (7Be normalised data corrected from cosmic ray cycle)

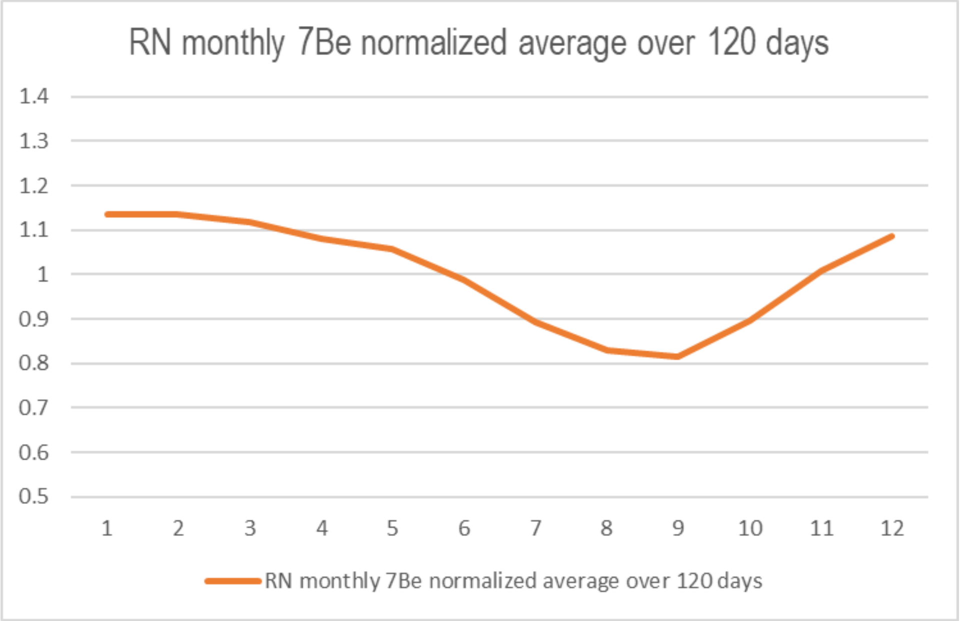
RN monthly 7Be normalized average over 120 days
1.4
1.3
1.2
1.1
1
0.9
0.8
0.7
0.6
0.5
1
2
3
4
5
6
7
8
9
10
11
12
RN monthly 7Be normalized average over 120 days

RN45

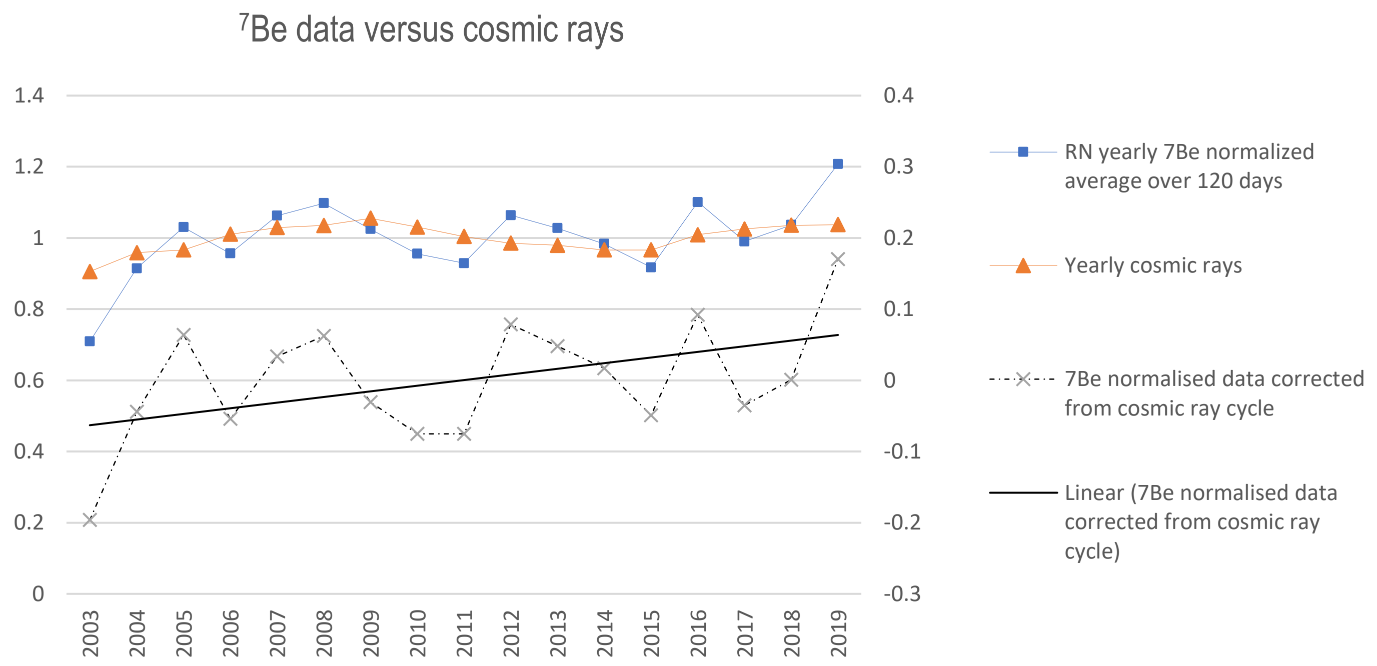

7Be data versus cosmic rays
1.4
1.2
1
0.8
0.6
0.4
0.2
0
0.4
0.3
0.2
0.1
0
-0.1
-0.2
-0.3
2003
2004
2005
2006
2007
2008
2009
2010
2011
2012
2013
2014
2015
2016
2017
2018
2019
RN yearly 7Be normalized average over 120 days
Yearly cosmic rays
7Be normalised data corrected from cosmic ray cycle
Linear (7Be normalised data corrected from cosmic ray cycle)

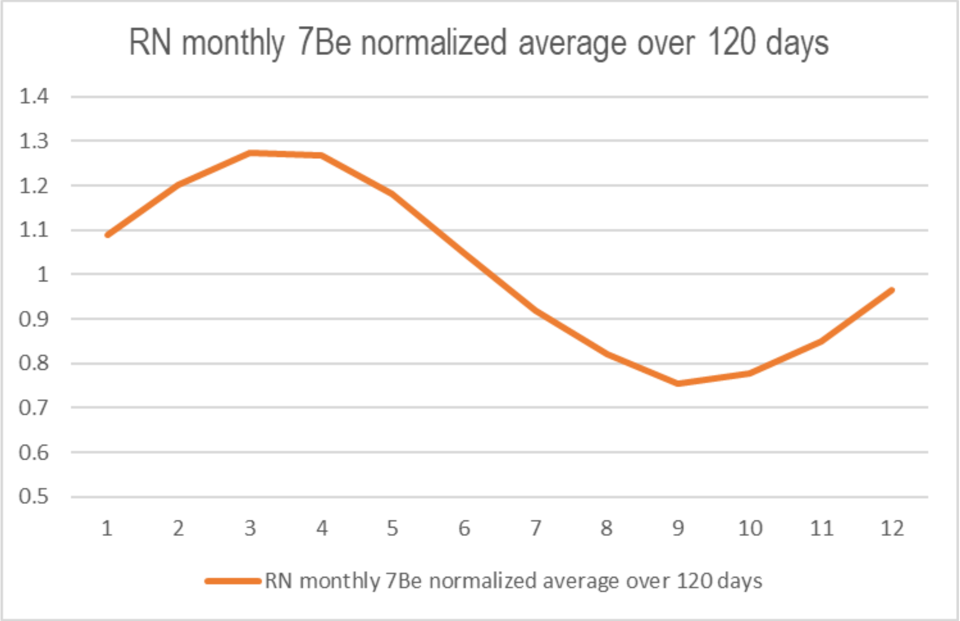

RN monthly 7Be normalized average over 120 days
1.4
1.3
1.2
1.1
1
0.9
0.8
0.7
0.6
0.5
1
2
3
4
5
6
7
8
9
10
11
12
RN monthly 7Be normalized average over 120 days

RN46

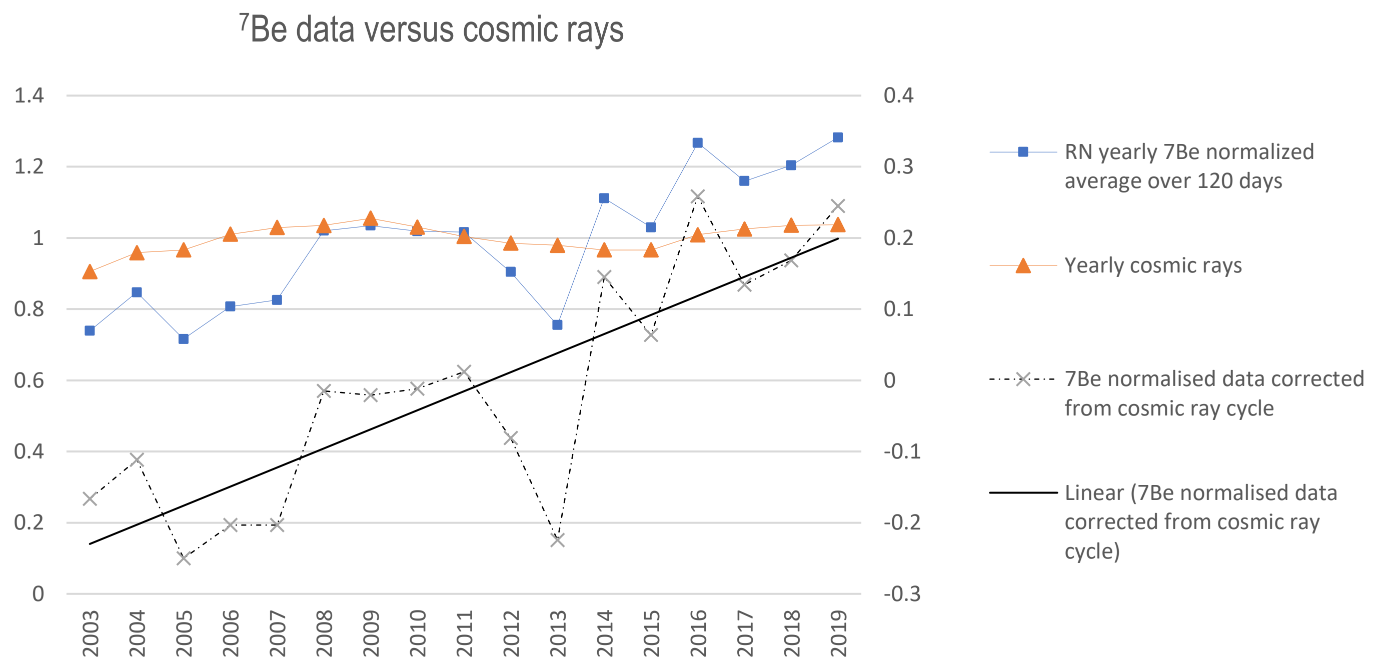

^{7}Be data versus cosmic rays
1.4
1.2
1
0.8
0.6
0.4
0.2
0
0.4
0.3
0.2
0.1
0
-0.1
-0.2
-0.3
2003
2004
2005
2006
2007
2008
2009
2010
2011
2012
2013
2014
2015
2016
2017
2018
2019
RN yearly 7Be normalized average over 120 days
Yearly cosmic rays
7Be normalised data corrected from cosmic ray cycle
Linear (7Be normalised data corrected from cosmic ray cycle)

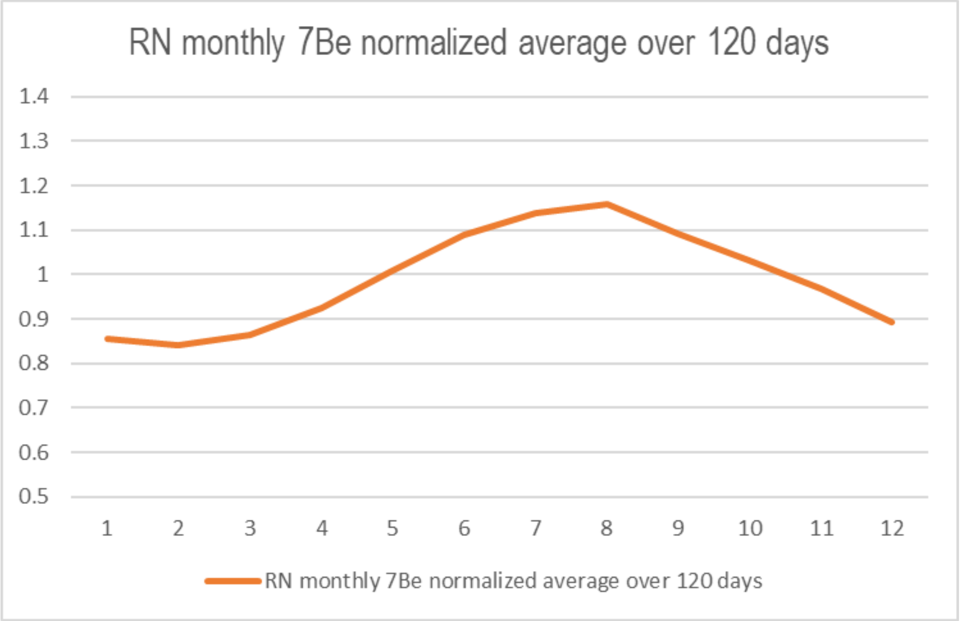

RN monthly 7Be normalized average over 120 days
1.4
1.3
1.2
1.1
1
0.9
0.8
0.7
0.6
0.5
1
2
3
4
5
6
7
8
9
10
11
12
RN monthly 7Be normalized average over 120 days

RN47

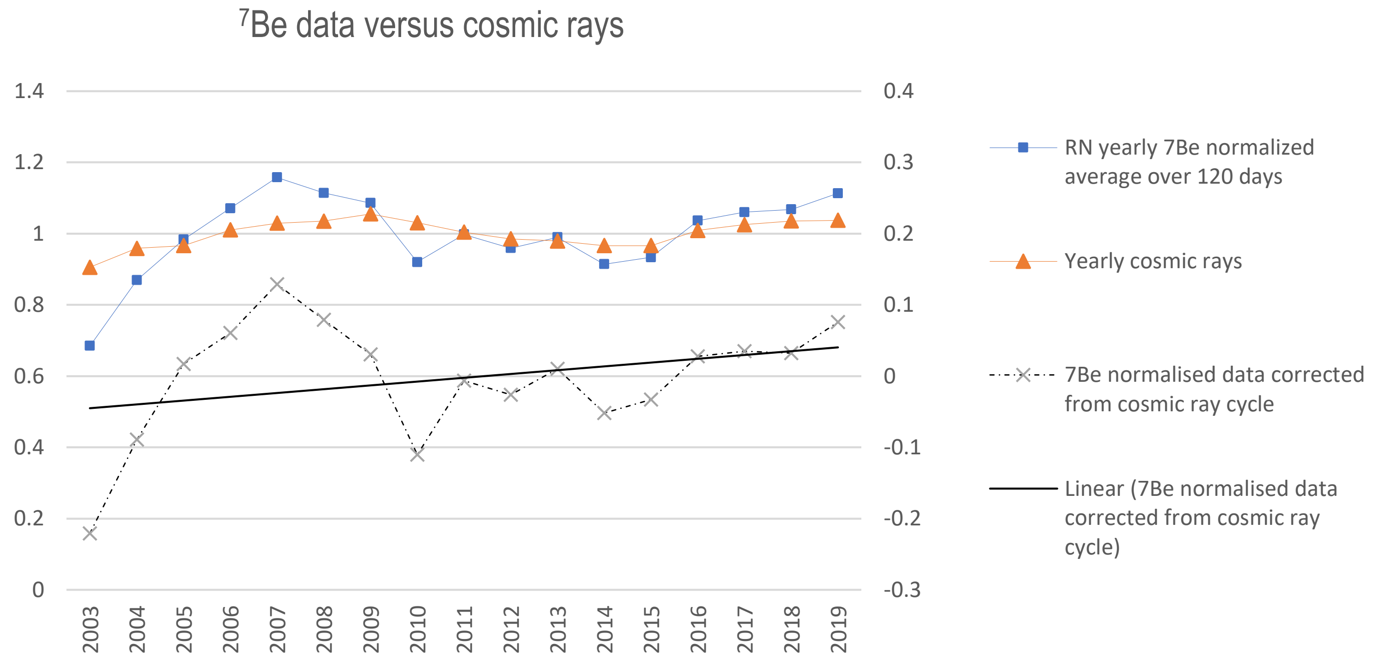

^{7}Be data versus cosmic rays
1.4
1.2
1
0.8
0.6
0.4
0.2
0
0.4
0.3
0.2
0.1
0
-0.1
-0.2
-0.3
2003
2004
2005
2006
2007
2008
2009
2010
2011
2012
2013
2014
2015
2016
2017
2018
2019
RN yearly 7Be normalized average over 120 days
Yearly cosmic rays
7Be normalised data corrected from cosmic ray cycle
Linear (7Be normalised data corrected from cosmic ray cycle)

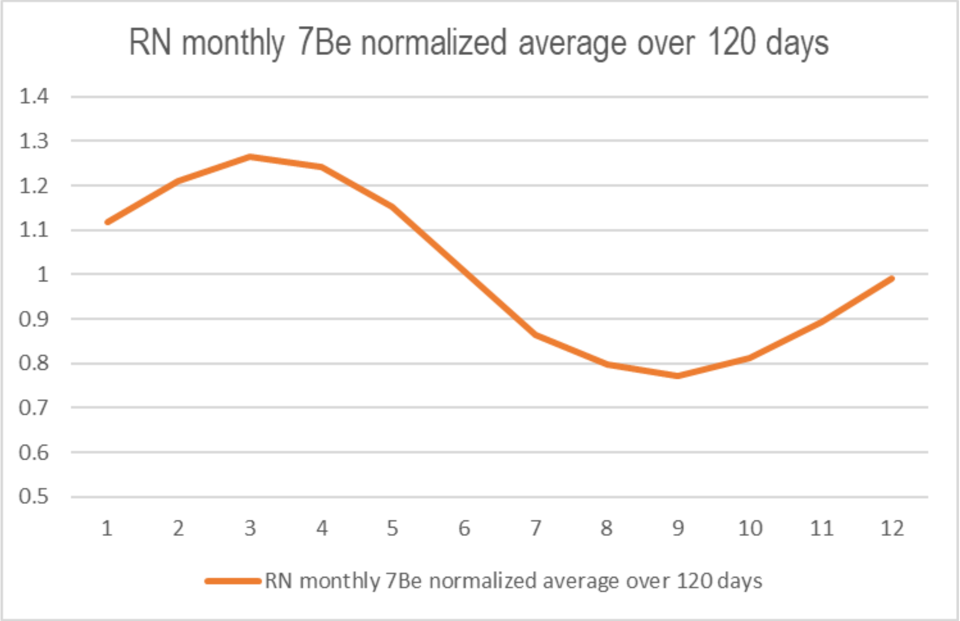

RN monthly 7Be normalized average over 120 days
1.4
1.3
1.2
1.1
1
0.9
0.8
0.7
0.6
0.5
1
2
3
4
5
6
7
8
9
10
11
12
RN monthly 7Be normalized average over 120 days

RN49

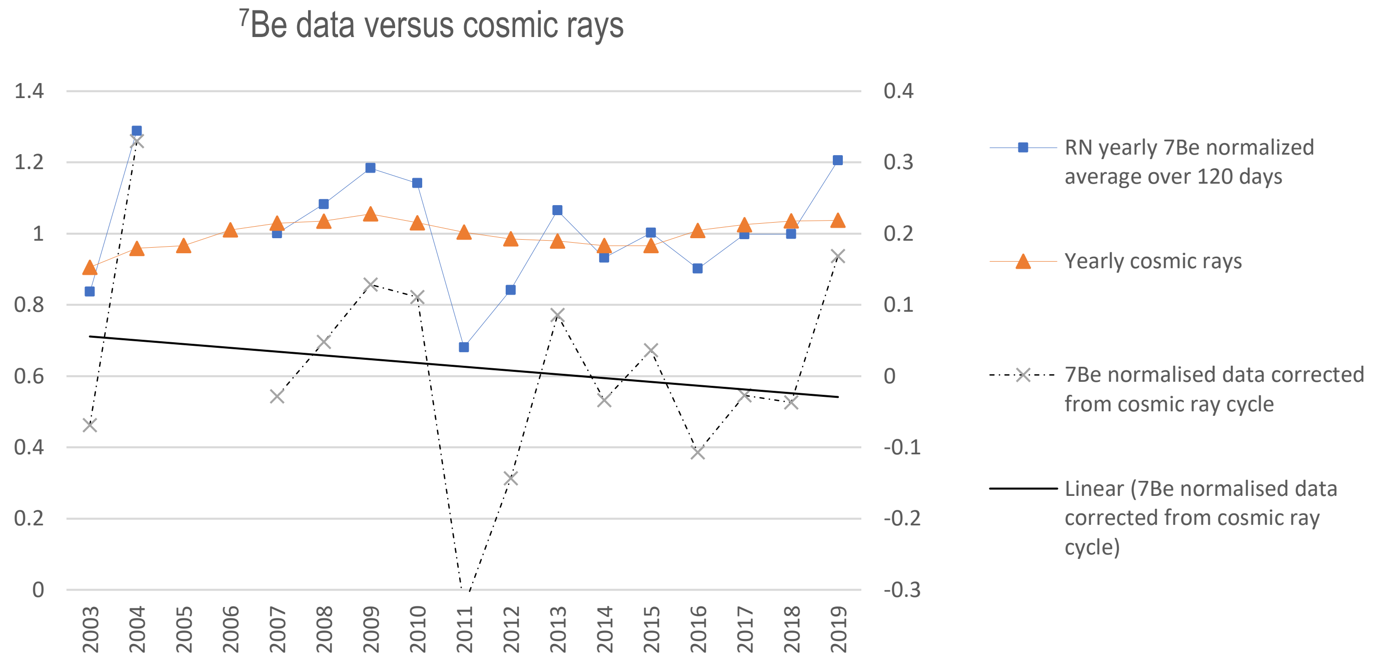

^{7}Be data versus cosmic rays
1.4
1.2
1
0.8
0.6
0.4
0.2
0
0.4
0.3
0.2
0.1
0
-0.1
-0.2
-0.3
2003
2004
2005
2006
2007
2008
2009
2010
2011
2012
2013
2014
2015
2016
2017
2018
2019
RN yearly 7Be normalized average over 120 days
Yearly cosmic rays
7Be normalised data corrected from cosmic ray cycle
Linear (7Be normalised data corrected from cosmic ray cycle)

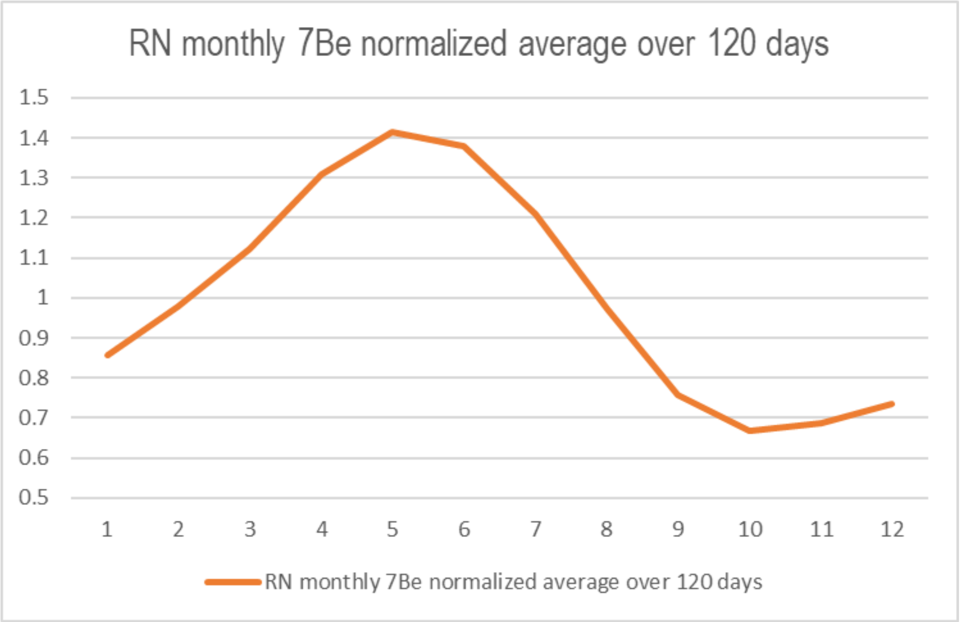

RN monthly 7Be normalized average over 120 days
1.5
1.4
1.3
1.2
1.1
1
0.9
0.8
0.7
0.6
0.5
1
2
3
4
5
6
7
8
9
10
11
12
RN monthly 7Be normalized average over 120 days

RN50

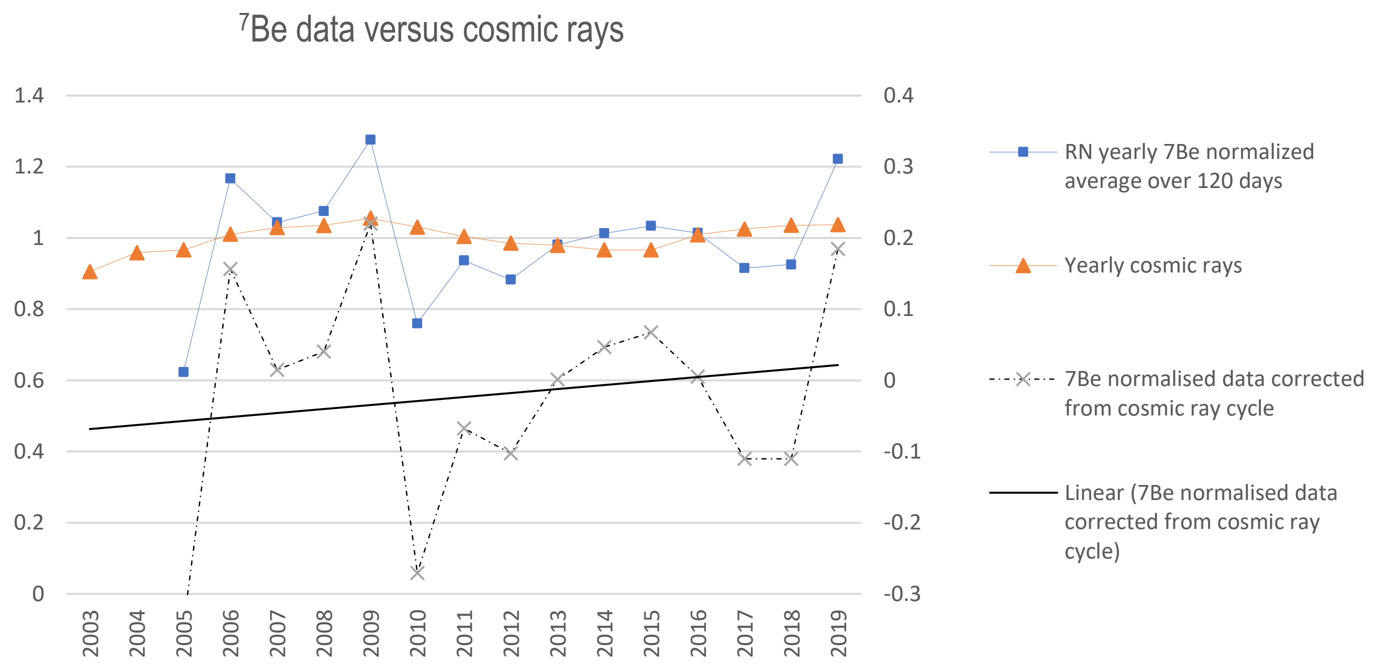

^{7}Be data versus cosmic rays
1.4
1.2
1
0.8
0.6
0.4
0.2
0
0.4
0.3
0.2
0.1
0
-0.1
-0.2
-0.3
2003
2004
2005
2006
2007
2008
2009
2010
2011
2012
2013
2014
2015
2016
2017
2018
2019
RN yearly 7Be normalized average over 120 days
Yearly cosmic rays
7Be normalised data corrected from cosmic ray cycle
Linear (7Be normalised data corrected from cosmic ray cycle)

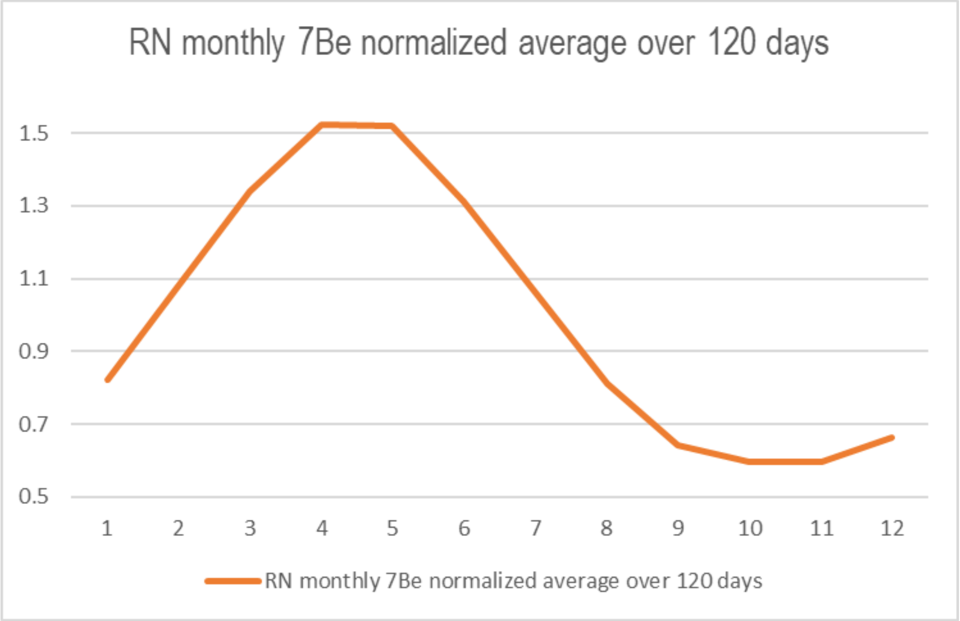

RN monthly 7Be normalized average over 120 days
1.5
1.3
1.1
0.9
0.7
0.5
1
2
3
4
5
6
7
8
9
10
11
12
RN monthly 7Be normalized average over 120 days

RN51

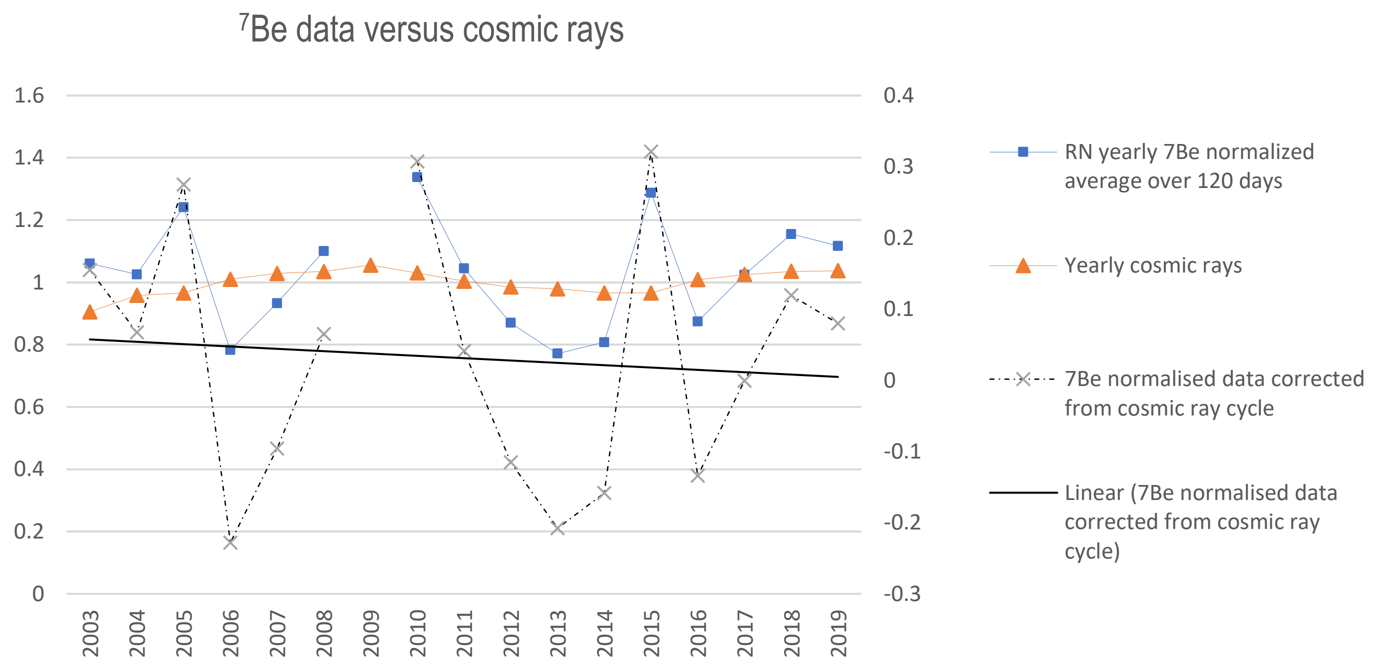

^{7}Be data versus cosmic rays
1.6
1.4
1.2
1
0.8
0.6
0.4
0.2
0
0.4
0.3
0.2
0.1
0
-0.1
-0.2
-0.3
2003
2004
2005
2006
2007
2008
2009
2010
2011
2012
2013
2014
2015
2016
2017
2018
2019
RN yearly 7Be normalized average over 120 days
Yearly cosmic rays
7Be normalised data corrected from cosmic ray cycle
Linear (7Be normalised data corrected from cosmic ray cycle)

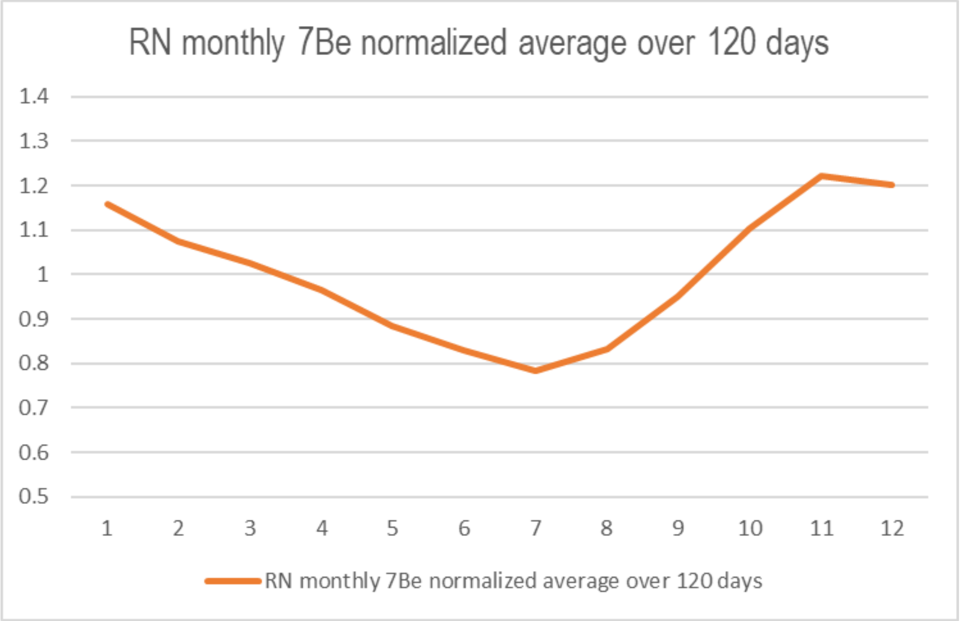

RN monthly 7Be normalized average over 120 days
1.4
1.3
1.2
1.1
1
0.9
0.8
0.7
0.6
0.5
1
2
3
4
5
6
7
8
9
10
11
12
RN monthly 7Be normalized average over 120 days

RN52

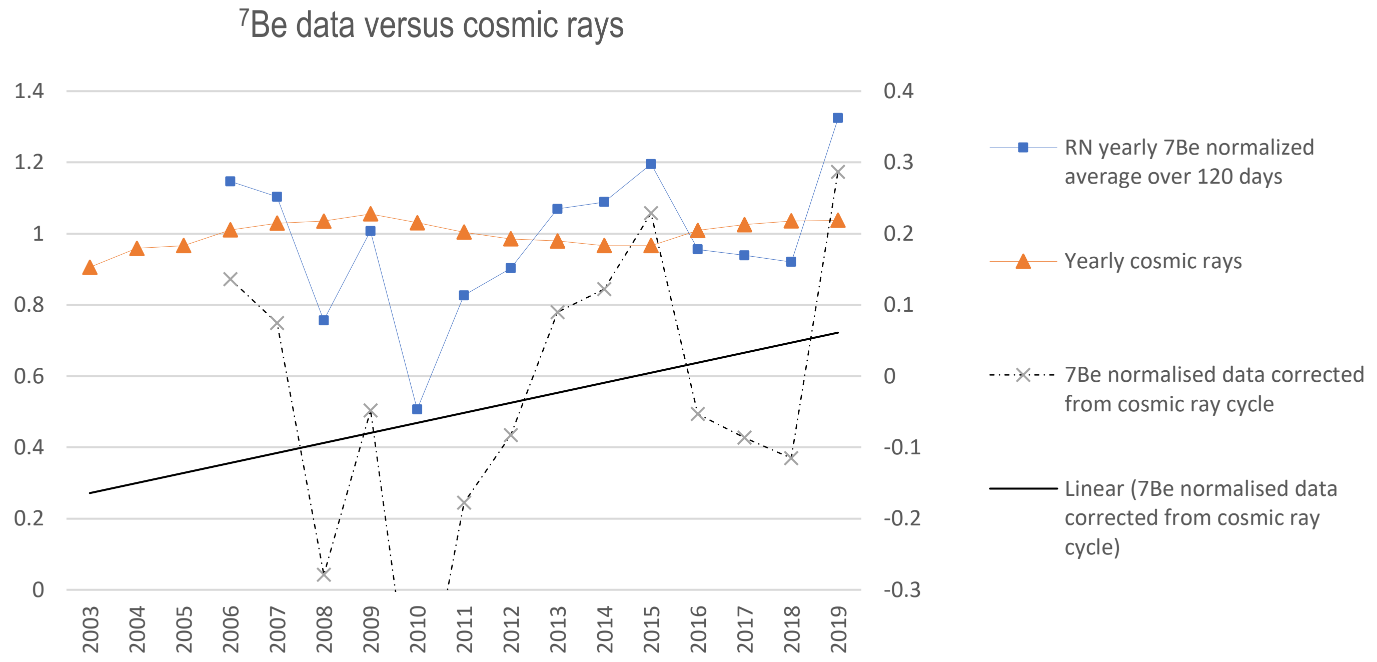

^{7}Be data versus cosmic rays
1.4
1.2
1
0.8
0.6
0.4
0.2
0
0.4
0.3
0.2
0.1
0
-0.1
-0.2
-0.3
2003
2004
2005
2006
2007
2008
2009
2010
2011
2012
2013
2014
2015
2016
2017
2018
2019
RN yearly 7Be normalized average over 120 days
Yearly cosmic rays
7Be normalised data corrected from cosmic ray cycle
Linear (7Be normalised data corrected from cosmic ray cycle)

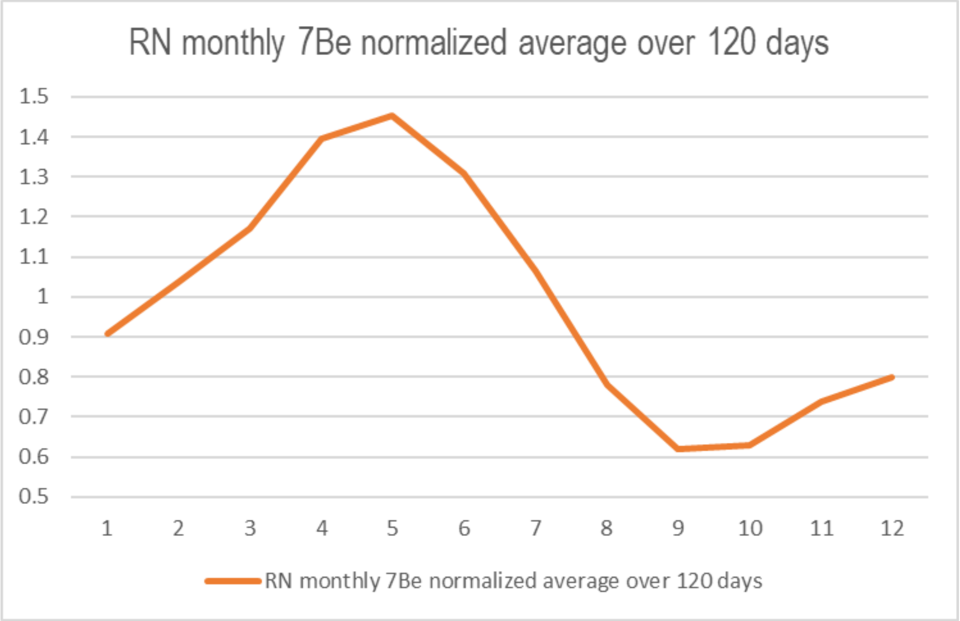

RN monthly 7Be normalized average over 120 days
1.5
1.4
1.3
1.2
1.1
1
0.9
0.8
0.7
0.6
0.5
1
2
3
4
5
6
7
8
9
10
11
12
RN monthly 7Be normalized average over 120 days

RN53

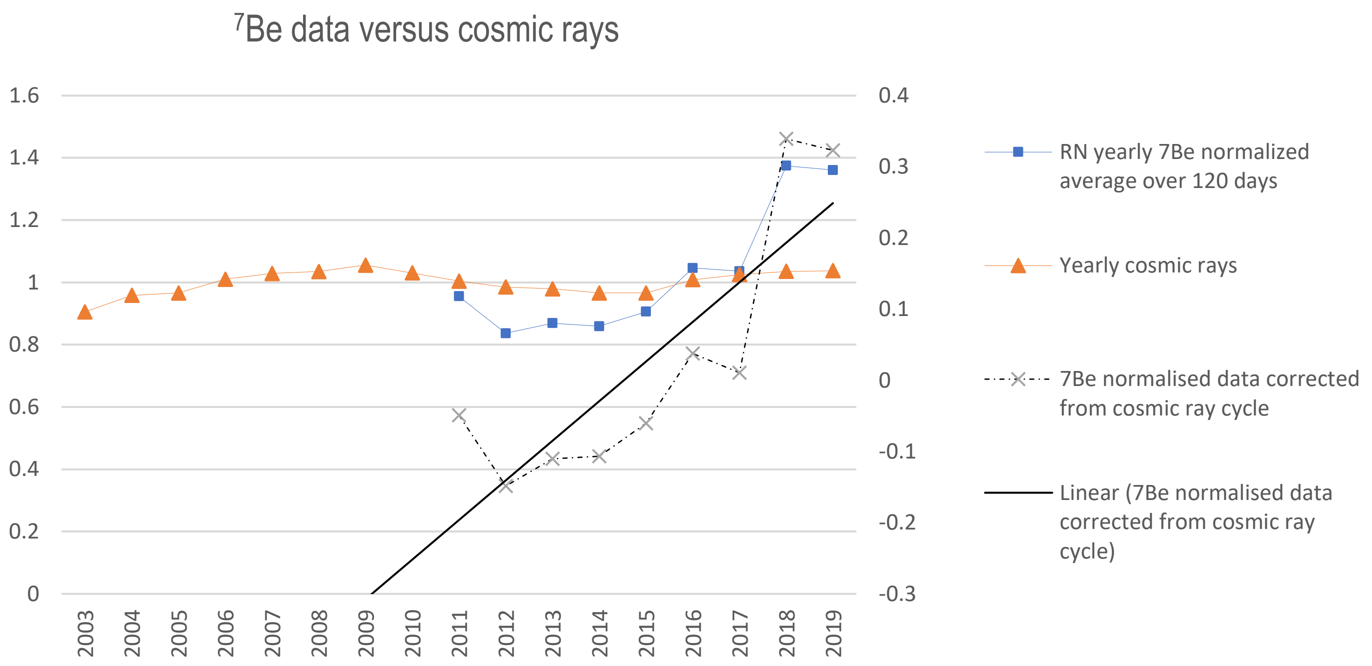

^{7}Be data versus cosmic rays
1.6
1.4
1.2
1
0.8
0.6
0.4
0.2
0
0.4
0.3
0.2
0.1
0
-0.1
-0.2
-0.3
2003
2004
2005
2006
2007
2008
2009
2010
2011
2012
2013
2014
2015
2016
2017
2018
2019
RN yearly 7Be normalized average over 120 days
Yearly cosmic rays
7Be normalised data corrected from cosmic ray cycle
Linear (7Be normalised data corrected from cosmic ray cycle)

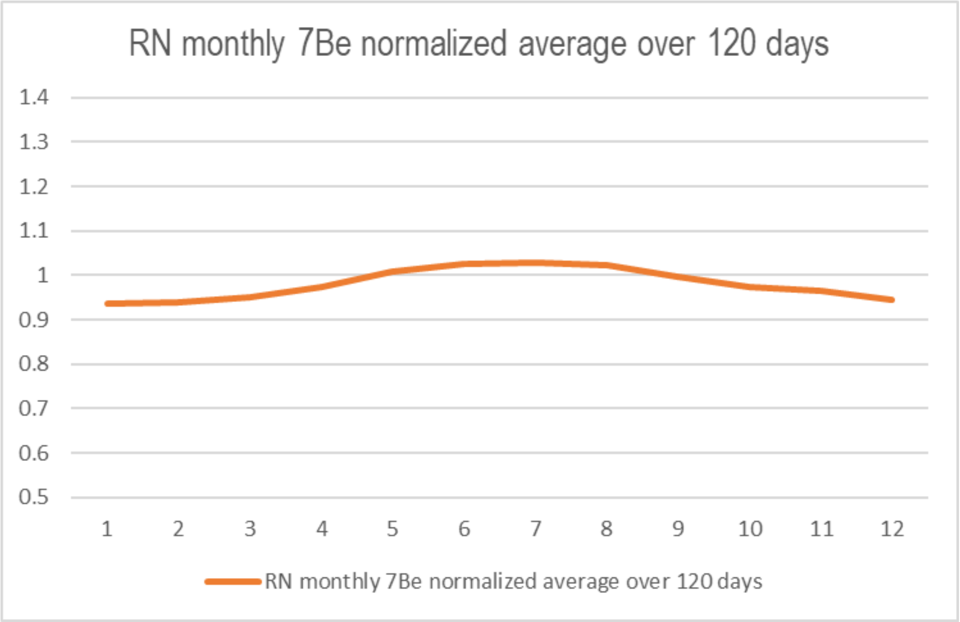

RN monthly 7Be normalized average over 120 days
1.4
1.3
1.2
1.1
1
0.9
0.8
0.7
0.6
0.5
1
2
3
4
5
6
7
8
9
10
11
12
RN monthly 7Be normalized average over 120 days

RN54

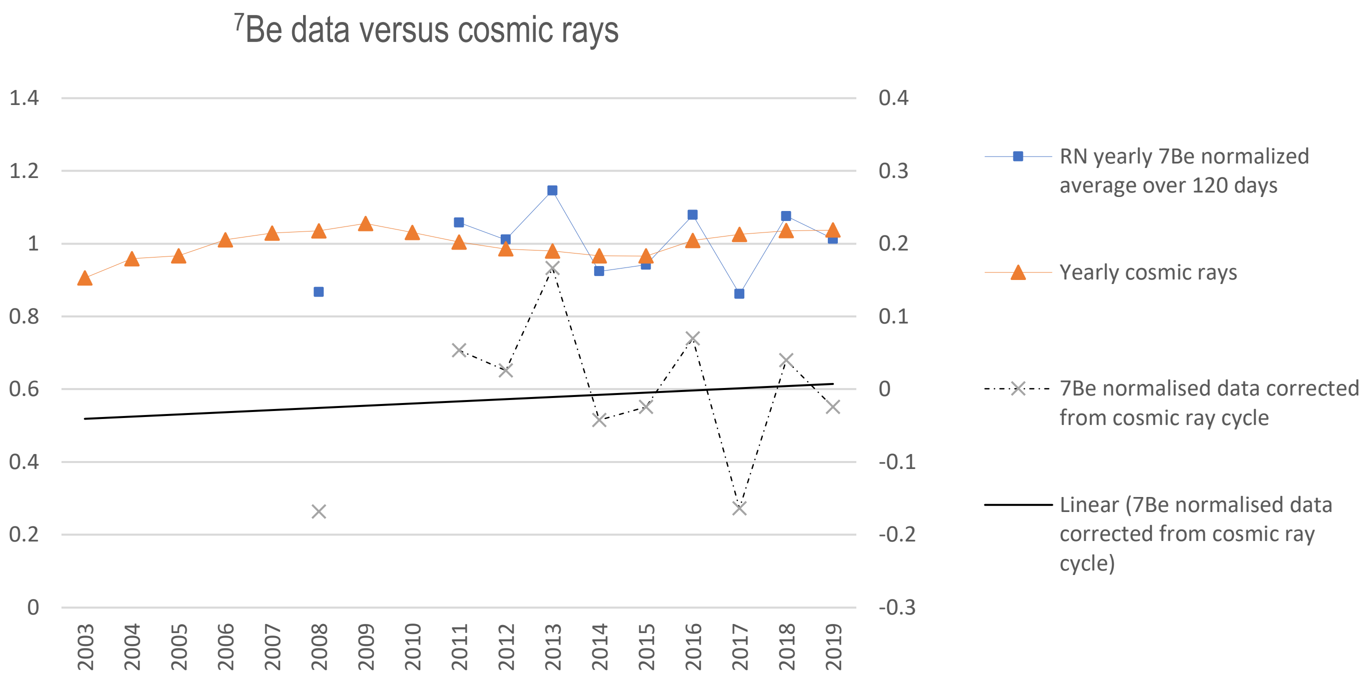

^{7}Be data versus cosmic rays
1.4
1.2
1
0.8
0.6
0.4
0.2
0
0.4
0.3
0.2
0.1
0
-0.1
-0.2
-0.3
2003
2004
2005
2006
2007
2008
2009
2010
2011
2012
2013
2014
2015
2016
2017
2018
2019
RN yearly 7Be normalized average over 120 days
Yearly cosmic rays
7Be normalised data corrected from cosmic ray cycle
Linear (7Be normalised data corrected from cosmic ray cycle)

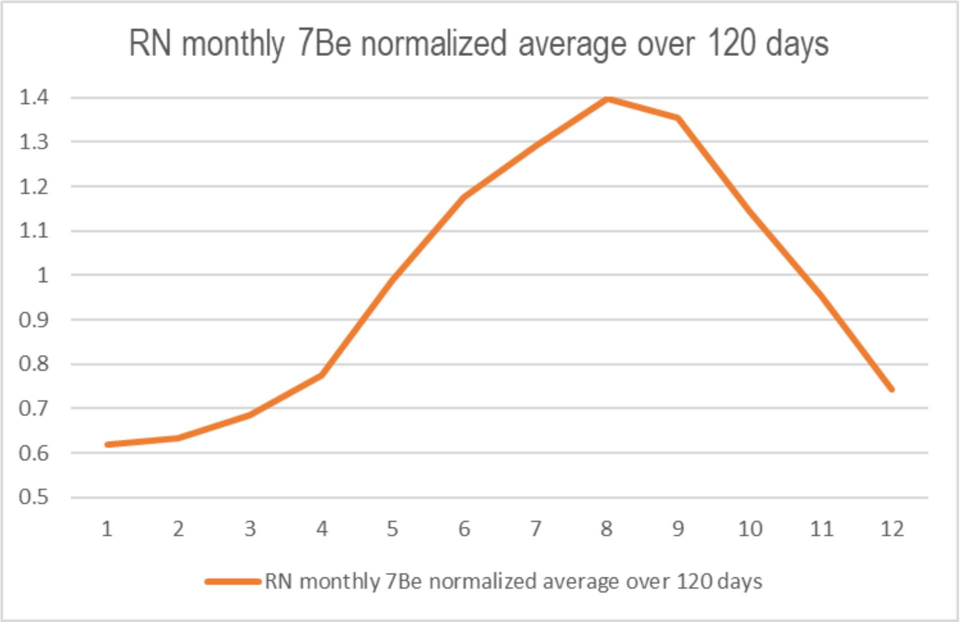

RN monthly 7Be normalized average over 120 days
1.4
1.3
1.2
1.1
1
0.9
0.8
0.7
0.6
0.5
1
2
3
4
5
6
7
8
9
10
11
12
RN monthly 7Be normalized average over 120 days

RN58

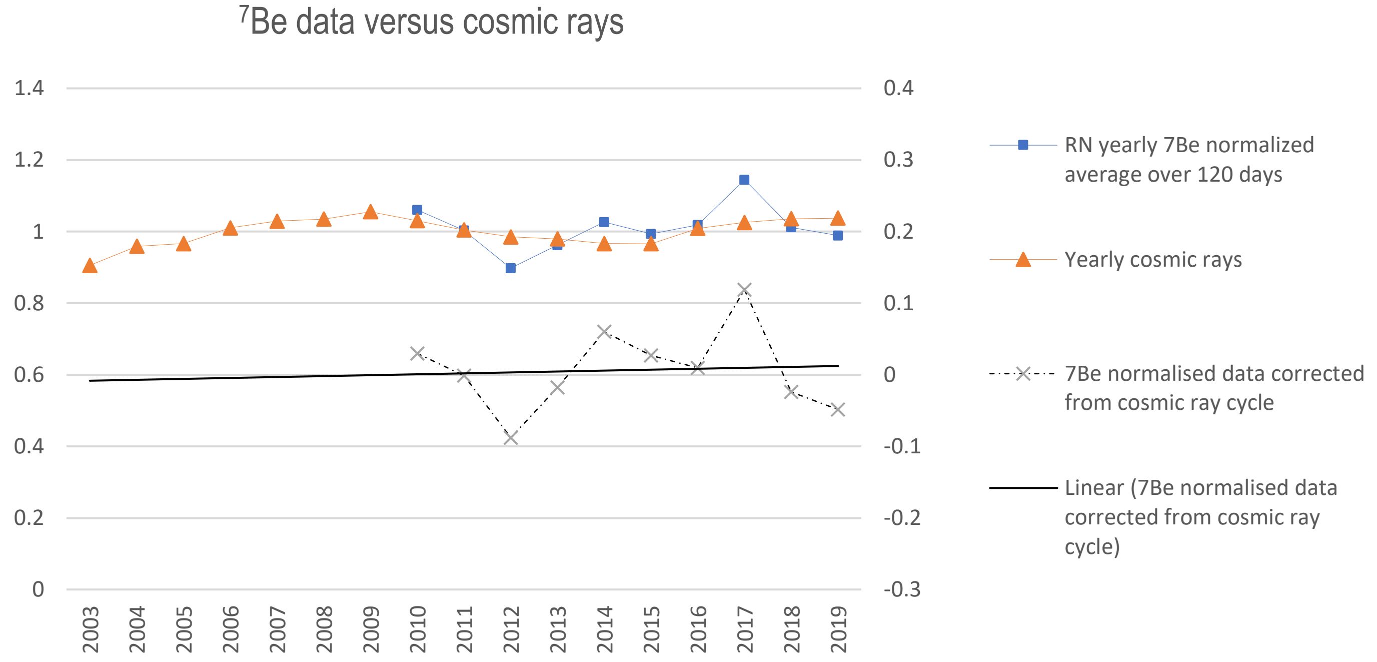

^{7}Be data versus cosmic rays
1.4
1.2
1
0.8
0.6
0.4
0.2
0
0.4
0.3
0.2
0.1
0
-0.1
-0.2
-0.3
2003
2004
2005
2006
2007
2008
2009
2010
2011
2012
2013
2014
2015
2016
2017
2018
2019
RN yearly 7Be normalized average over 120 days
Yearly cosmic rays
7Be normalised data corrected from cosmic ray cycle
Linear (7Be normalised data corrected from cosmic ray cycle)

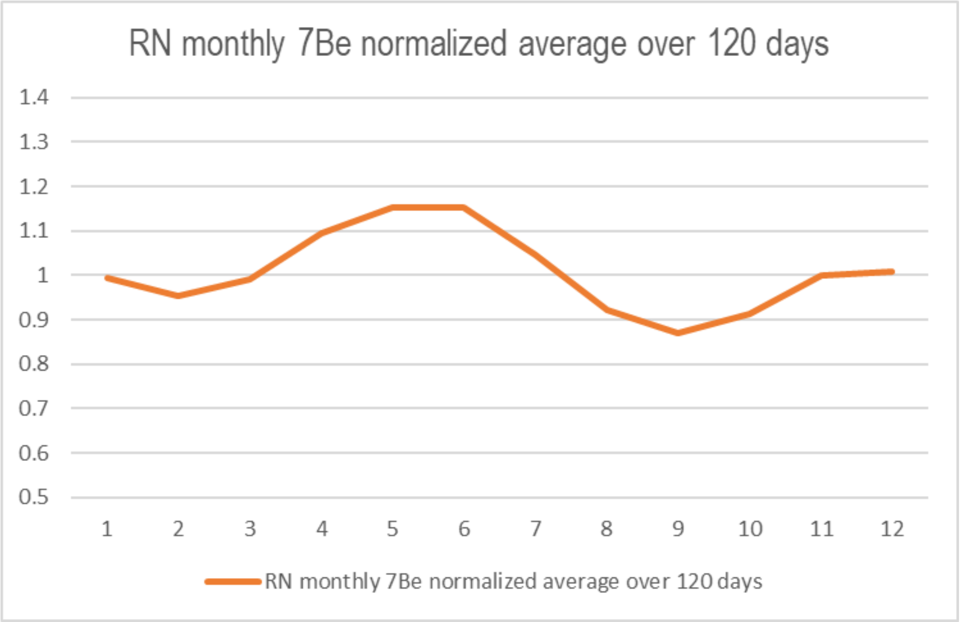

RN monthly 7Be normalized average over 120 days
1.4
1.3
1.2
1.1
1
0.9
0.8
0.7
0.6
0.5
1
2
3
4
5
6
7
8
9
10
11
12
RN monthly 7Be normalized average over 120 days

RN59

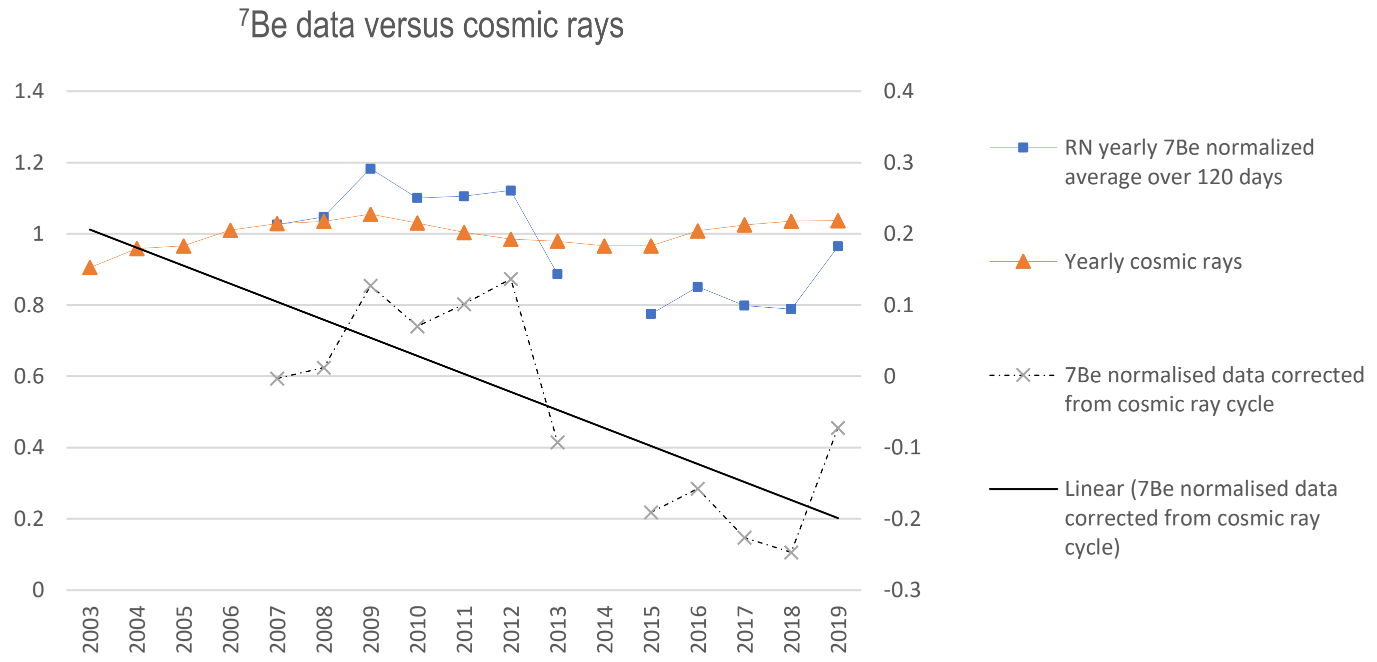
7Be data versus cosmic rays
1.4
1.2
1
0.8
0.6
0.4
0.2
0
0.4
0.3
0.2
0.1
0
-0.1
-0.2
-0.3
2003
2004
2005
2006
2007
2008
2009
2010
2011
2012
2013
2014
2015
2016
2017
2018
2019
RN yearly 7Be normalized average over 120 days
Yearly cosmic rays
7Be normalised data corrected from cosmic ray cycle
Linear (7Be normalised data corrected from cosmic ray cycle)

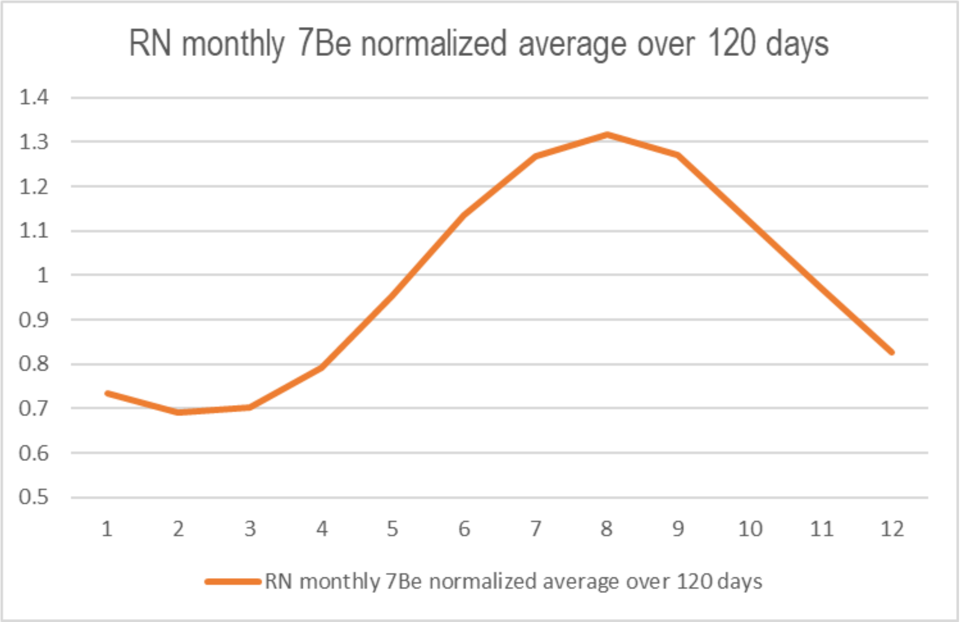
RN monthly 7Be normalized average over 120 days
1.4
1.3
1.2
1.1
1
0.9
0.8
0.7
0.6
0.5
1
2
3
4
5
6
7
8
9
10
11
12
RN monthly 7Be normalized average over 120 days

RN60

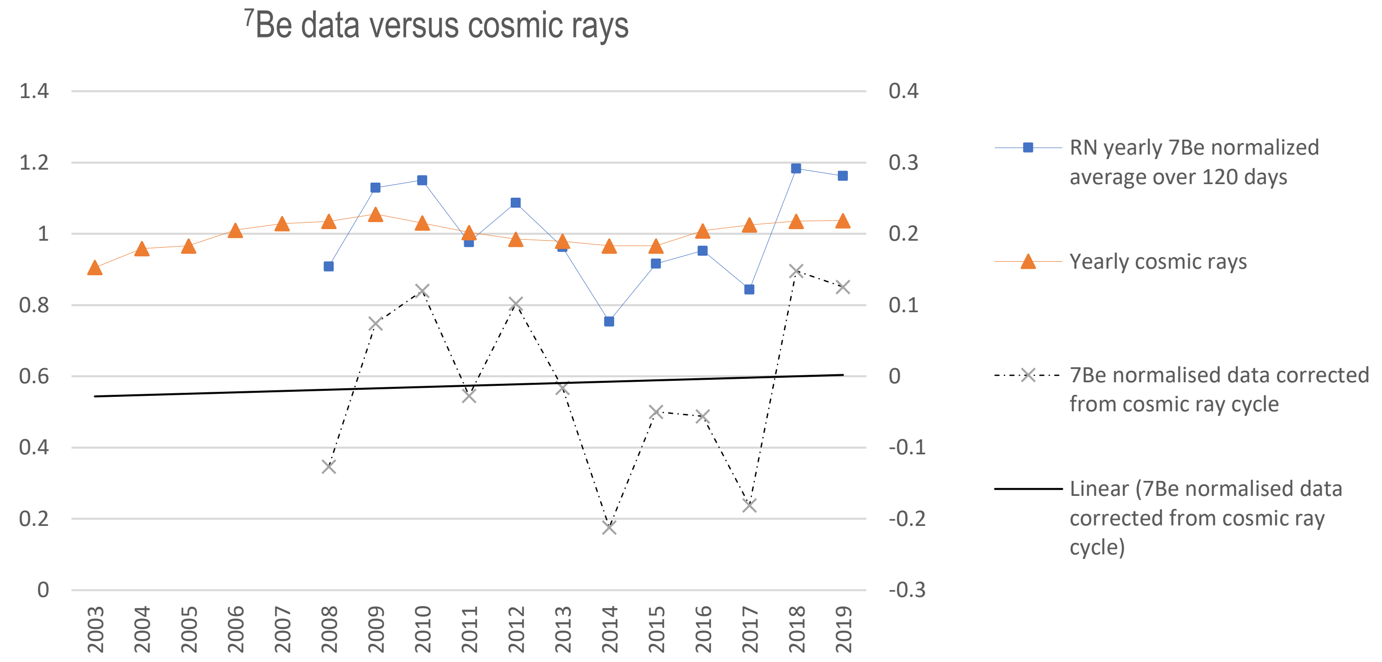

7Be data versus cosmic rays
1.4
1.2
1
0.8
0.6
0.4
0.2
0
0.4
0.3
0.2
0.1
0
-0.1
-0.2
-0.3
2003
2004
2005
2006
2007
2008
2009
2010
2011
2012
2013
2014
2015
2016
2017
2018
2019
RN yearly 7Be normalized average over 120 days
Yearly cosmic rays
7Be normalised data corrected from cosmic ray cycle
Linear (7Be normalised data corrected from cosmic ray cycle)

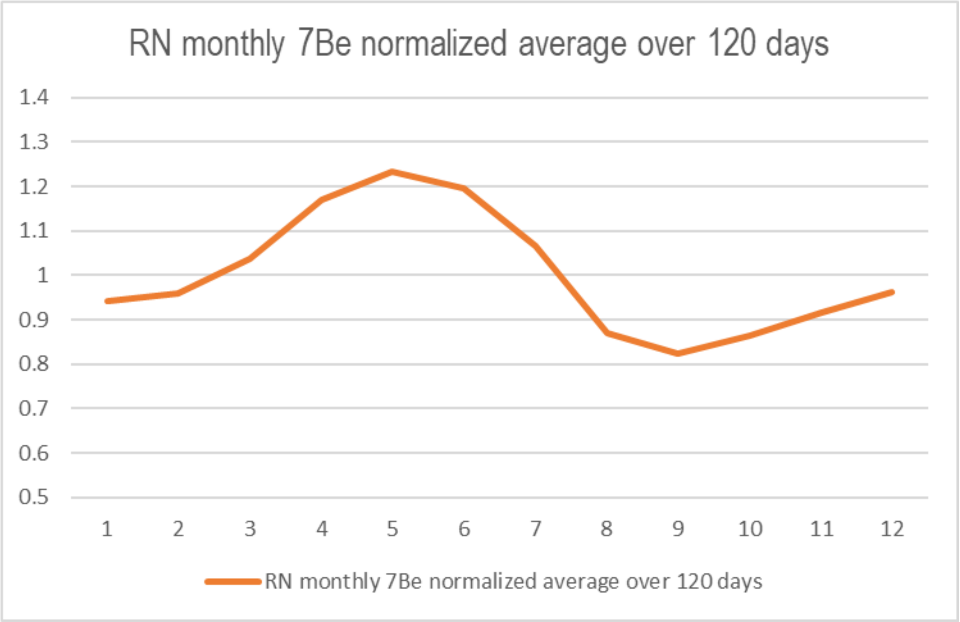

RN monthly 7Be normalized average over 120 days
1.4
1.3
1.2
1.1
1
0.9
0.8
0.7
0.6
0.5
1
2
3
4
5
6
7
8
9
10
11
12
RN monthly 7Be normalized average over 120 days

RN61

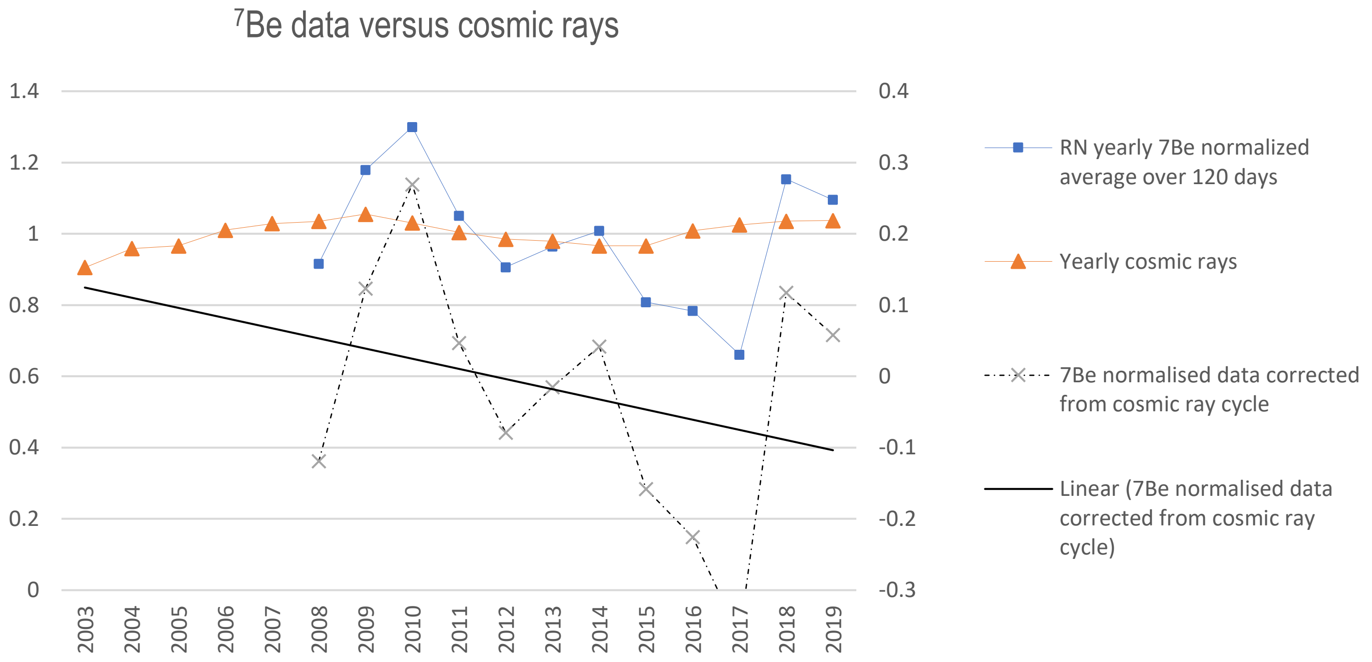

7Be data versus cosmic rays
1.4
1.2
1
0.8
0.6
0.4
0.2
0
0.4
0.3
0.2
0.1
0
-0.1
-0.2
-0.3
2003
2004
2005
2006
2007
2008
2009
2010
2011
2012
2013
2014
2015
2016
2017
2018
2019
RN yearly 7Be normalized average over 120 days
Yearly cosmic rays
7Be normalised data corrected from cosmic ray cycle
Linear (7Be normalised data corrected from cosmic ray cycle)

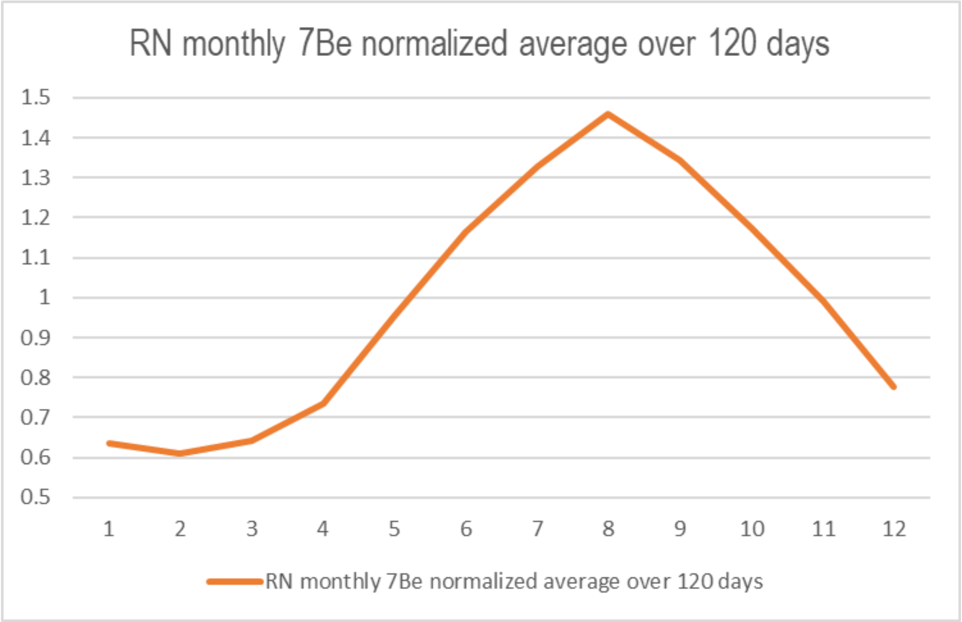

RN monthly 7Be normalized average over 120 days
1.5
1.4
1.3
1.2
1.1
1
0.9
0.8
0.7
0.6
0.5
1
2
3
4
5
6
7
8
9
10
11
12
RN monthly 7Be normalized average over 120 days

RN63

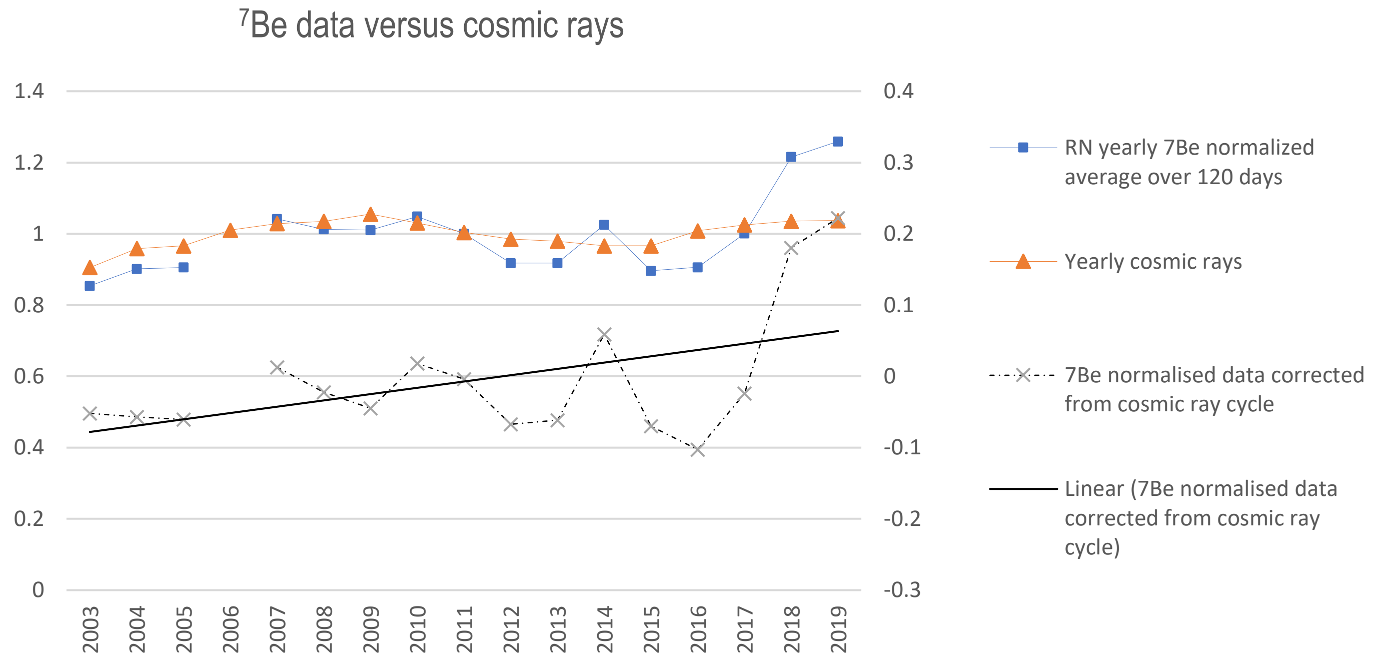
^{7}Be data versus cosmic rays
1.4
1.2
1
0.8
0.6
0.4
0.2
0
0.4
0.3
0.2
0.1
0
-0.1
-0.2
-0.3
2003
2004
2005
2006
2007
2008
2009
2010
2011
2012
2013
2014
2015
2016
2017
2018
2019
RN yearly 7Be normalized average over 120 days
Yearly cosmic rays
7Be normalised data corrected from cosmic ray cycle
Linear (7Be normalised data corrected from cosmic ray cycle)

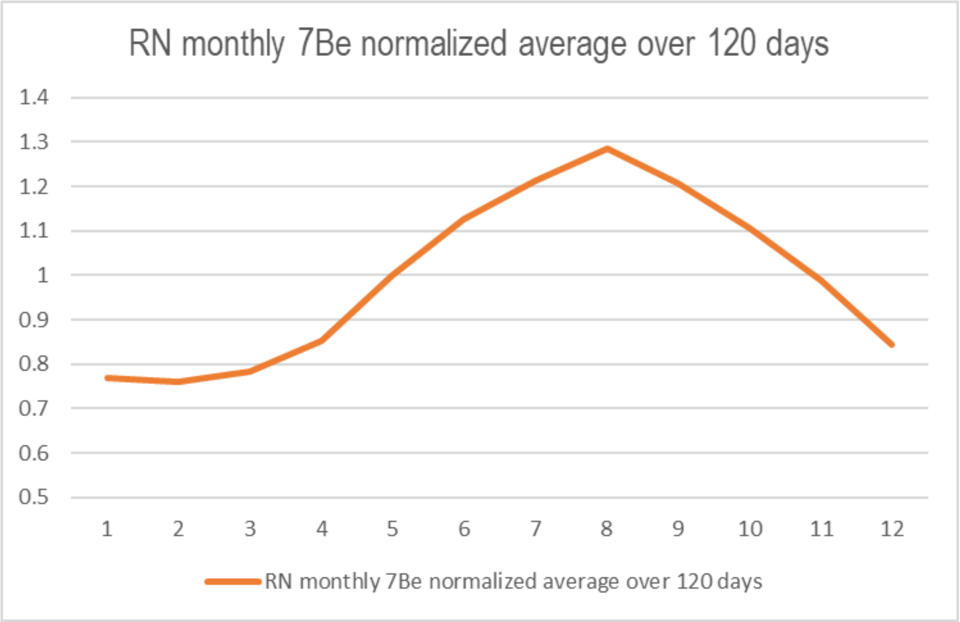
RN monthly 7Be normalized average over 120 days
1.4
1.3
1.2
1.1
1
0.9
0.8
0.7
0.6
0.5
1
2
3
4
5
6
7
8
9
10
11
12
RN monthly 7Be normalized average over 120 days

RN64

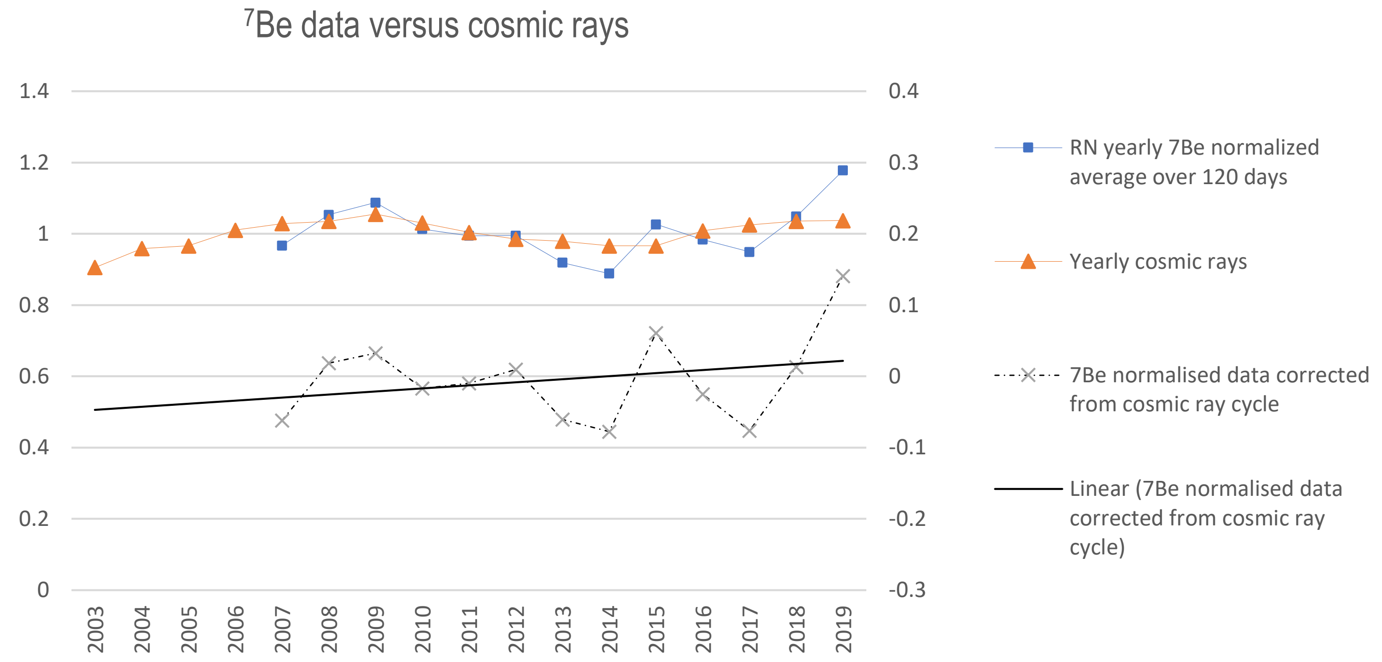

^{7}Be data versus cosmic rays
1.4
1.2
1
0.8
0.6
0.4
0.2
0
0.4
0.3
0.2
0.1
0
-0.1
-0.2
-0.3
2003
2004
2005
2006
2007
2008
2009
2010
2011
2012
2013
2014
2015
2016
2017
2018
2019
RN yearly 7Be normalized average over 120 days
Yearly cosmic rays
7Be normalised data corrected from cosmic ray cycle
Linear (7Be normalised data corrected from cosmic ray cycle)

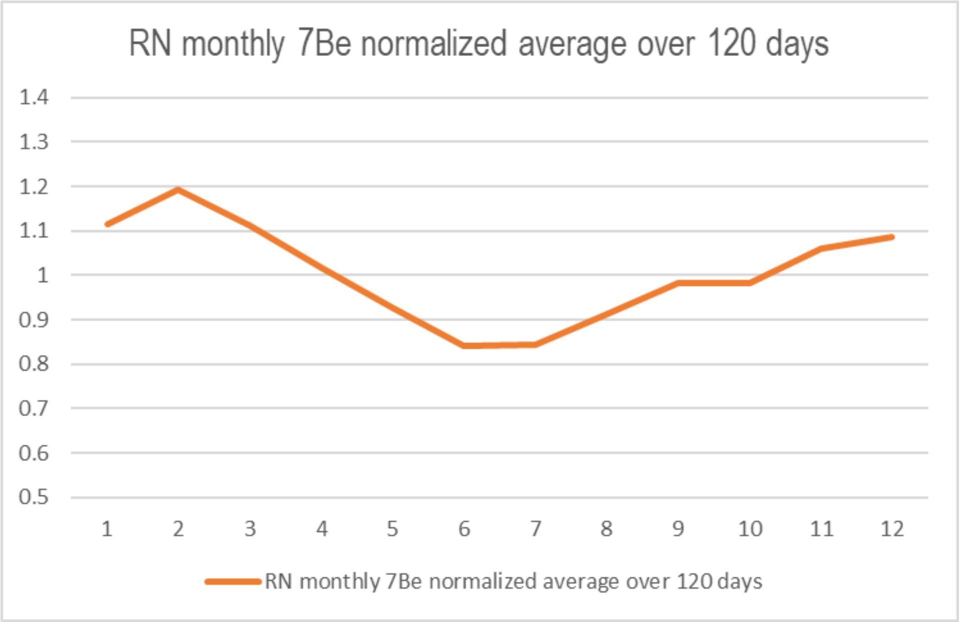

RN monthly 7Be normalized average over 120 days
1.4
1.3
1.2
1.1
1
0.9
0.8
0.7
0.6
0.5
1
2
3
4
5
6
7
8
9
10
11
12
RN monthly 7Be normalized average over 120 days

RN66

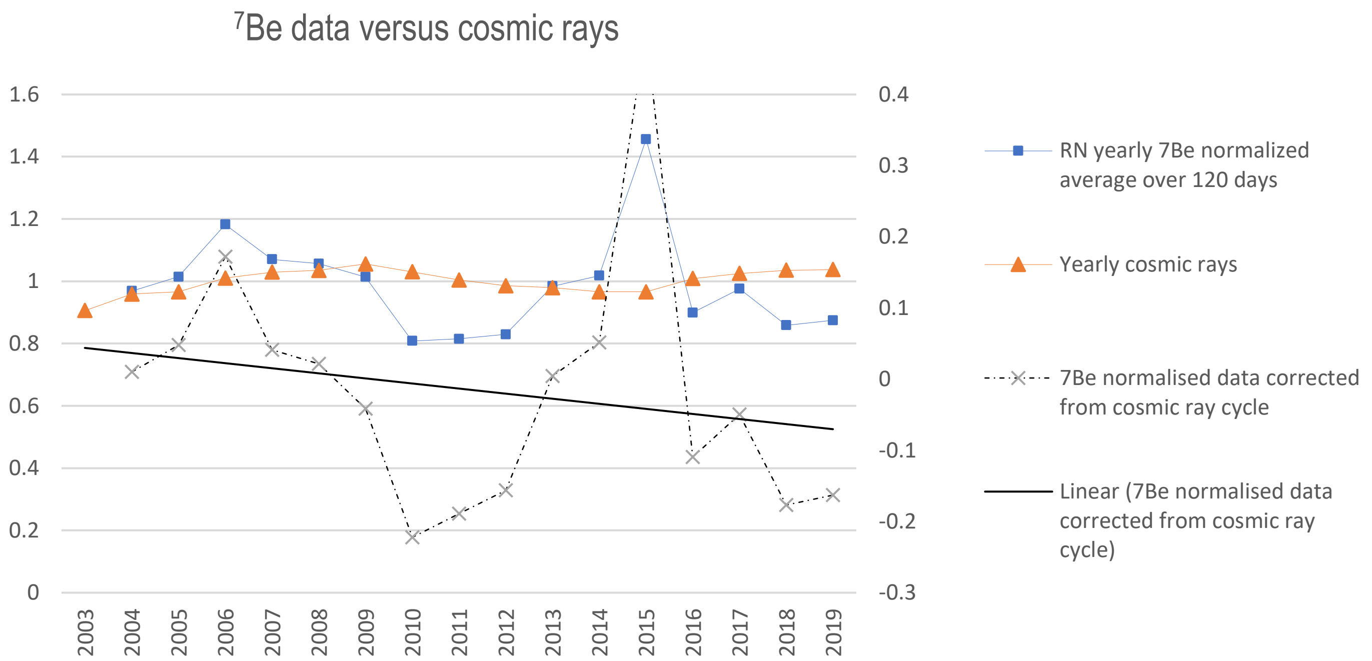

^{7}Be data versus cosmic rays
1.6
1.4
1.2
1
0.8
0.6
0.4
0.2
0
0.4
0.3
0.2
0.1
0
-0.1
-0.2
-0.3
2003
2004
2005
2006
2007
2008
2009
2010
2011
2012
2013
2014
2015
2016
2017
2018
2019
RN yearly 7Be normalized average over 120 days
Yearly cosmic rays
7Be normalised data corrected from cosmic ray cycle
Linear (7Be normalised data corrected from cosmic ray cycle)

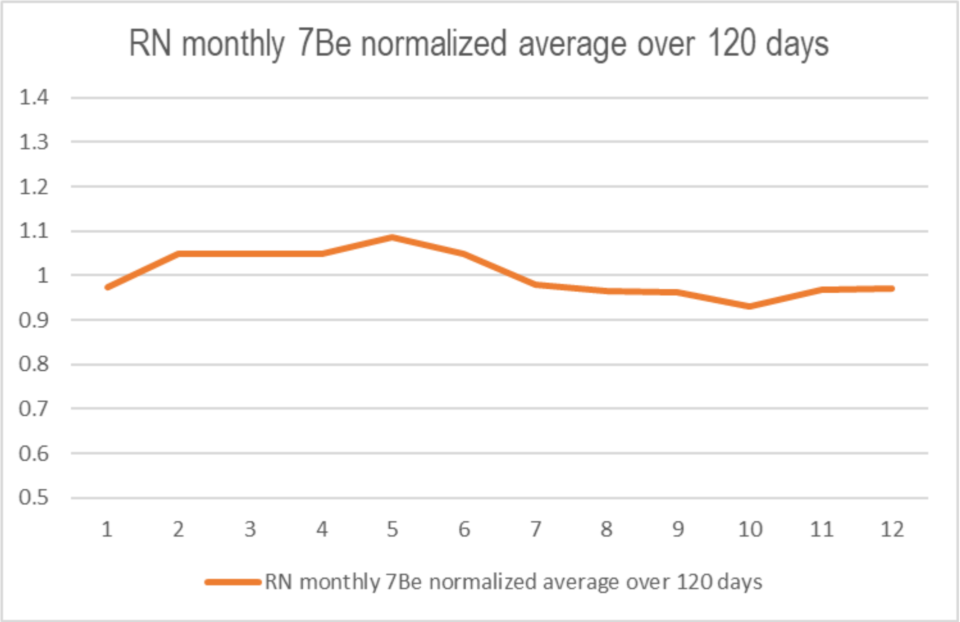

RN monthly 7Be normalized average over 120 days
1.4
1.3
1.2
1.1
1
0.9
0.8
0.7
0.6
0.5
1
2
3
4
5
6
7
8
9
10
11
12
RN monthly 7Be normalized average over 120 days

RN67

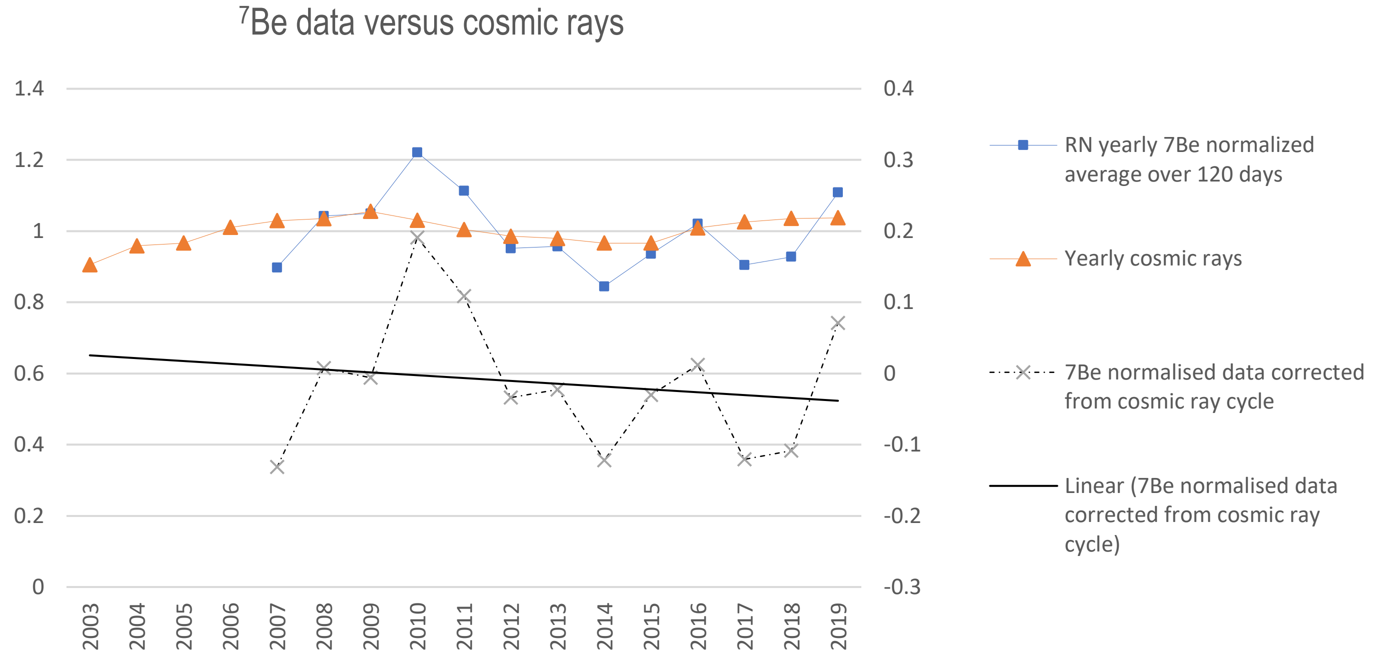

^{7}Be data versus cosmic rays
1.4
1.2
1
0.8
0.6
0.4
0.2
0
0.4
0.3
0.2
0.1
0
-0.1
-0.2
-0.3
2003
2004
2005
2006
2007
2008
2009
2010
2011
2012
2013
2014
2015
2016
2017
2018
2019
RN yearly 7Be normalized average over 120 days
Yearly cosmic rays
7Be normalised data corrected from cosmic ray cycle
Linear (7Be normalised data corrected from cosmic ray cycle)

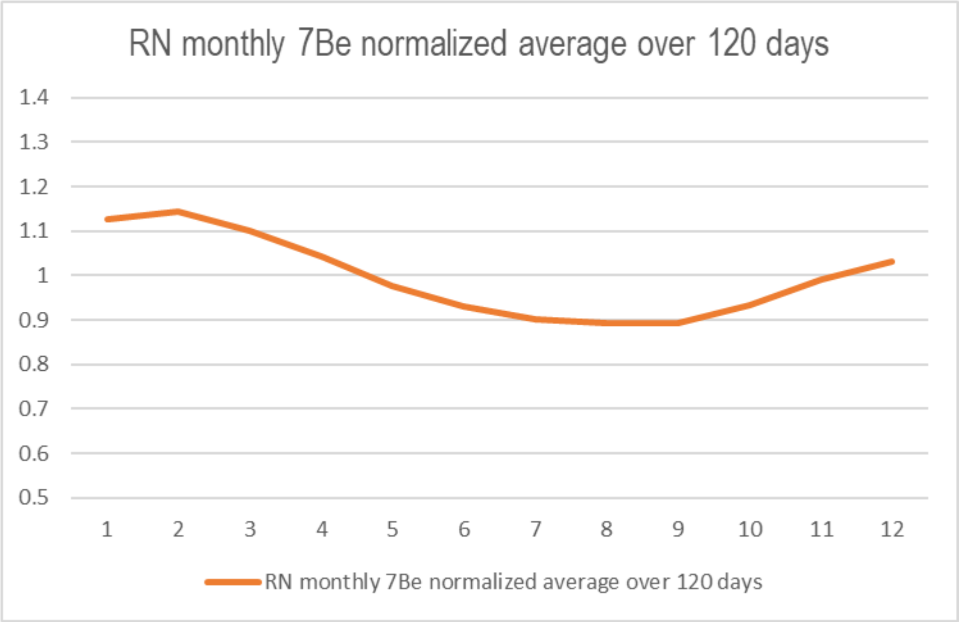

RN monthly 7Be normalized average over 120 days
1.4
1.3
1.2
1.1
1
0.9
0.8
0.7
0.6
0.5
1
2
3
4
5
6
7
8
9
10
11
12
RN monthly 7Be normalized average over 120 days

RN68

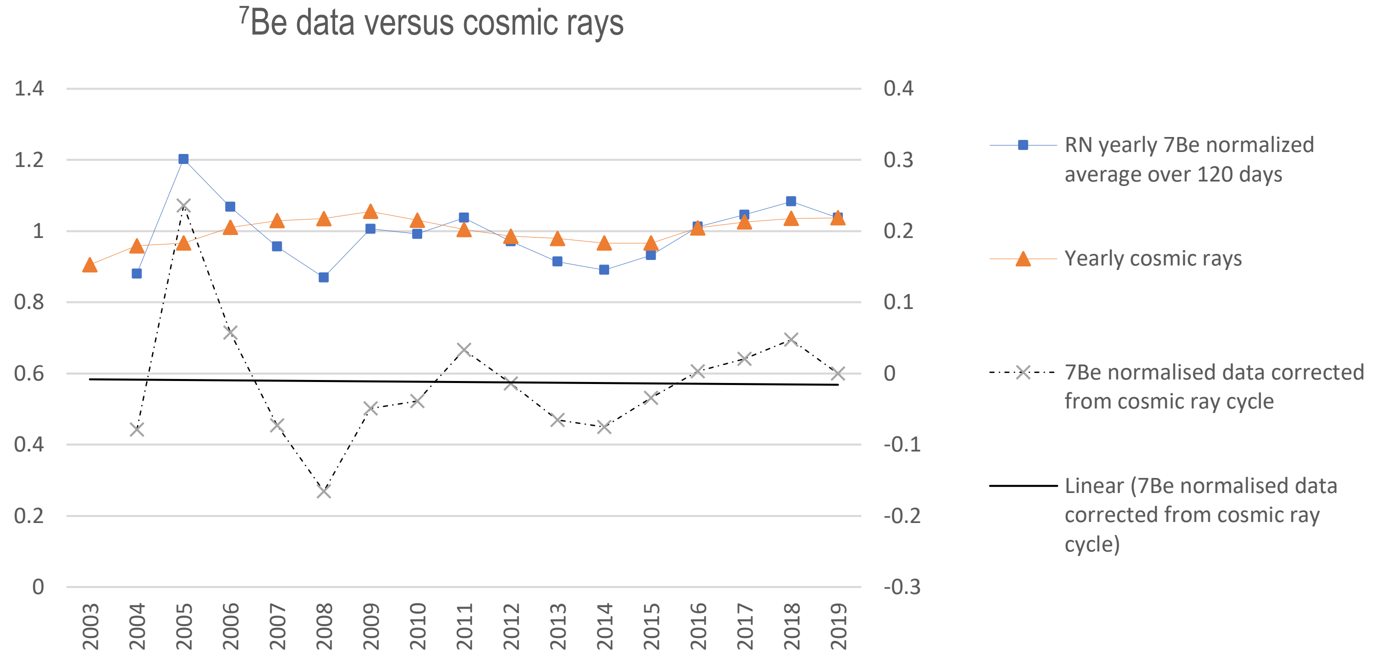

^{7}Be data versus cosmic rays
1.4
1.2
1
0.8
0.6
0.4
0.2
0
0.4
0.3
0.2
0.1
0
-0.1
-0.2
-0.3
2003 2004 2005 2006 2007 2008 2009 2010 2011 2012 2013 2014 2015 2016 2017 2018 2019
RN yearly 7Be normalized average over 120 days
Yearly cosmic rays
7Be normalised data corrected from cosmic ray cycle
Linear (7Be normalised data corrected from cosmic ray cycle)

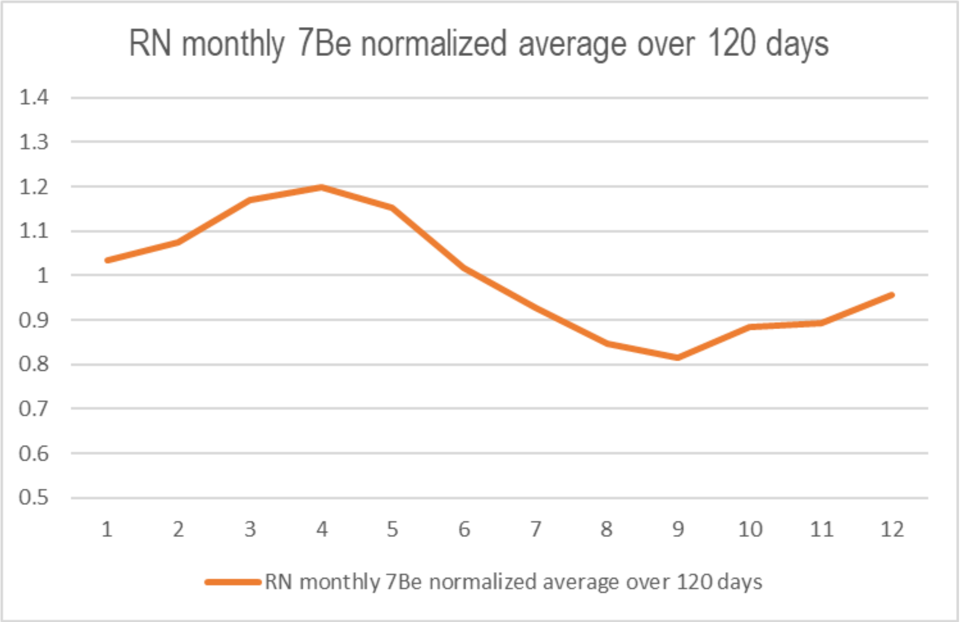

RN monthly 7Be normalized average over 120 days
1.4
1.3
1.2
1.1
1
0.9
0.8
0.7
0.6
0.5
1 2 3 4 5 6 7 8 9 10 11 12
RN monthly 7Be normalized average over 120 days

RN70

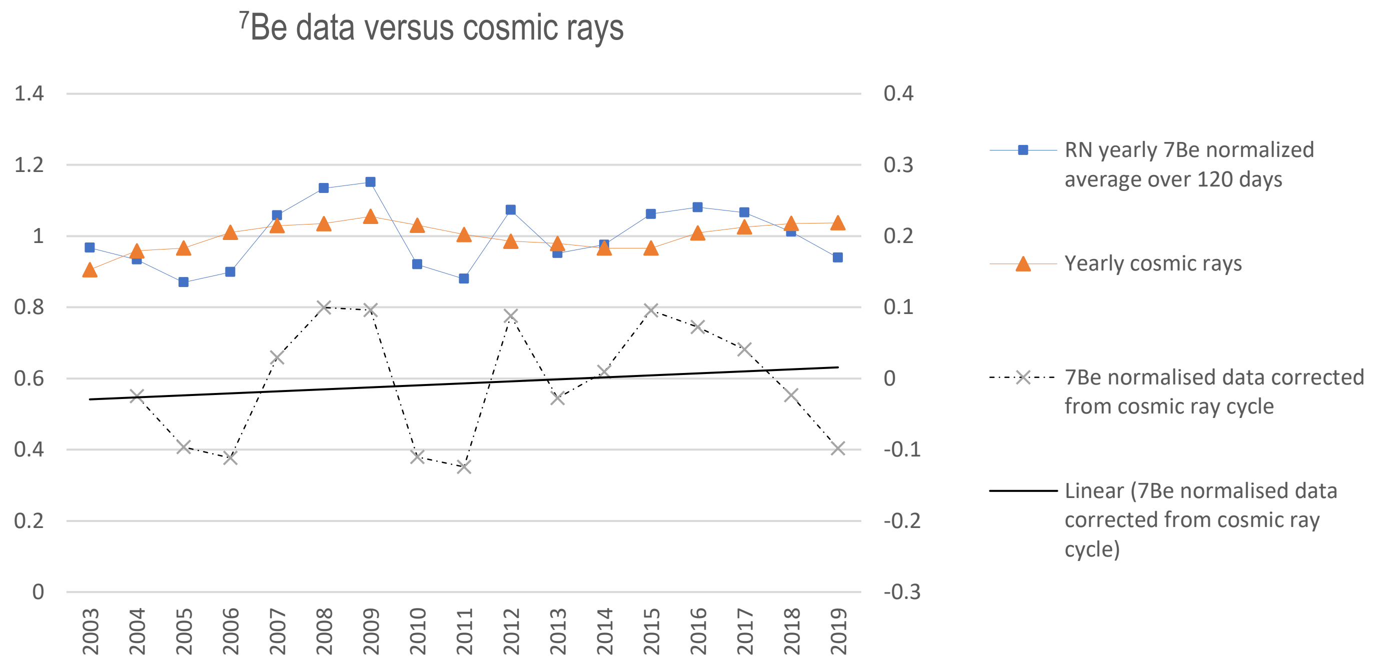

^{7}Be data versus cosmic rays
1.4
1.2
1
0.8
0.6
0.4
0.2
0
0.4
0.3
0.2
0.1
0
-0.1
-0.2
-0.3
2003
2004
2005
2006
2007
2008
2009
2010
2011
2012
2013
2014
2015
2016
2017
2018
2019
RN yearly 7Be normalized average over 120 days
Yearly cosmic rays
7Be normalised data corrected from cosmic ray cycle
Linear (7Be normalised data corrected from cosmic ray cycle)

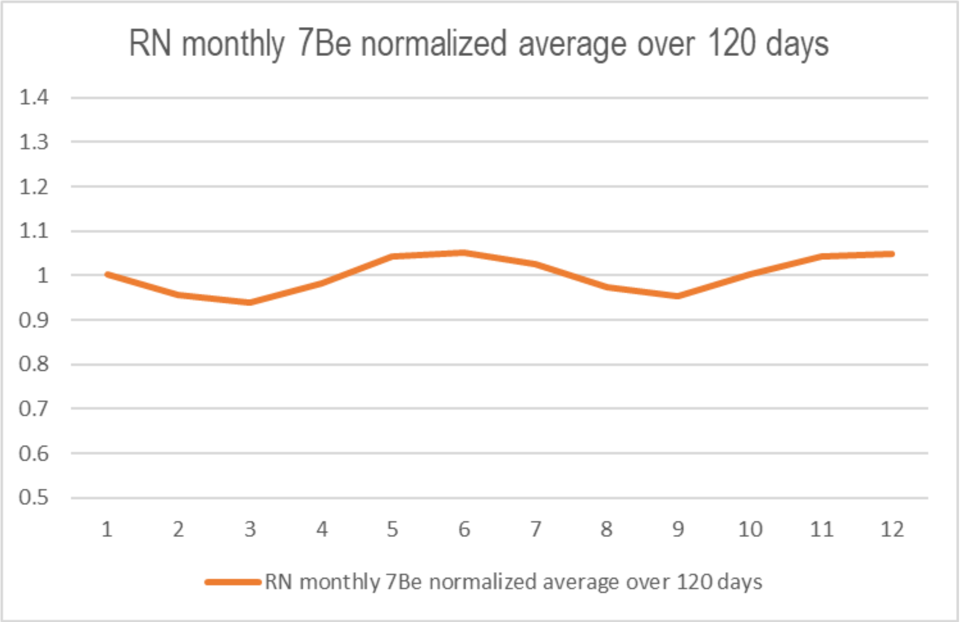

RN monthly 7Be normalized average over 120 days
1.4
1.3
1.2
1.1
1
0.9
0.8
0.7
0.6
0.5
1
2
3
4
5
6
7
8
9
10
11
12
RN monthly 7Be normalized average over 120 days

RN71

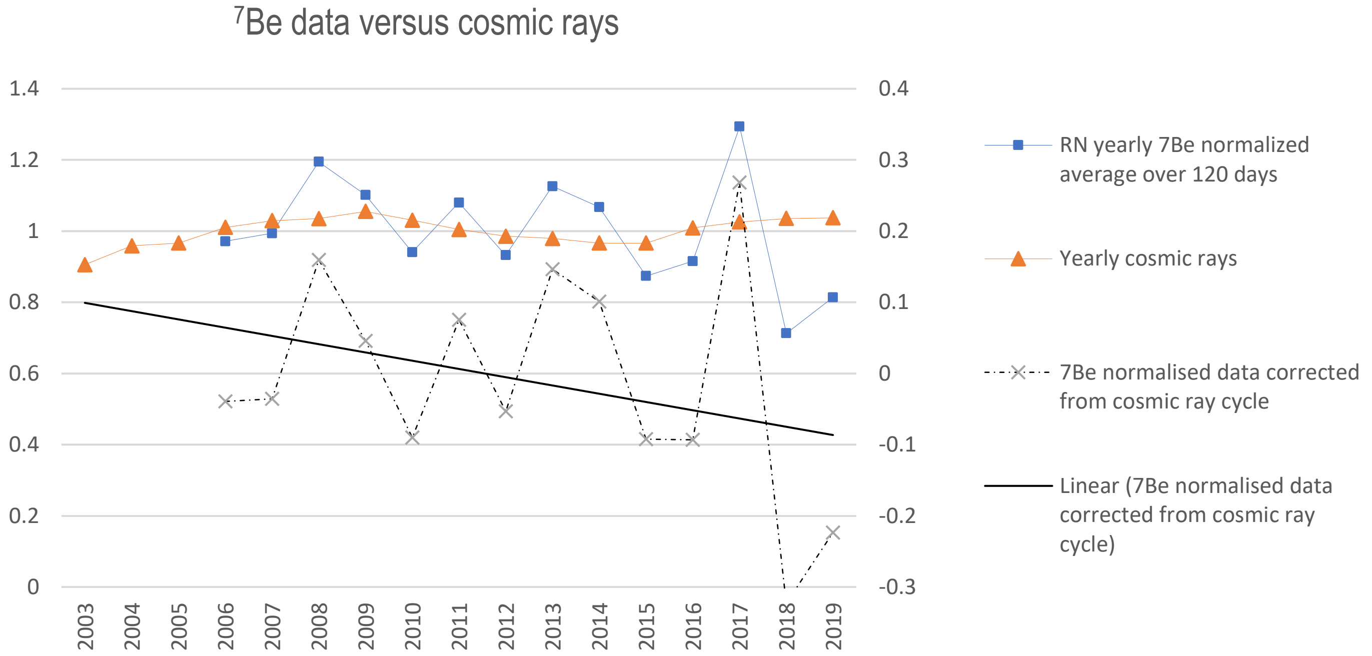

^{7}Be data versus cosmic rays
1.4
1.2
1
0.8
0.6
0.4
0.2
0
0.4
0.3
0.2
0.1
0
-0.1
-0.2
-0.3
2003
2004
2005
2006
2007
2008
2009
2010
2011
2012
2013
2014
2015
2016
2017
2018
2019
RN yearly 7Be normalized average over 120 days
Yearly cosmic rays
7Be normalised data corrected from cosmic ray cycle
Linear (7Be normalised data corrected from cosmic ray cycle)

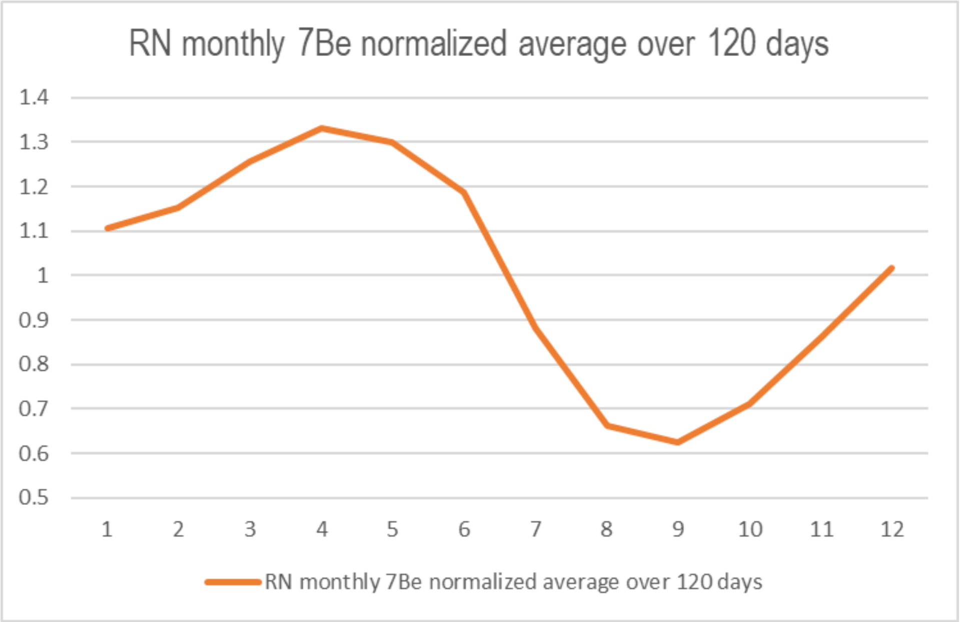

RN monthly 7Be normalized average over 120 days
1.4
1.3
1.2
1.1
1
0.9
0.8
0.7
0.6
0.5
1
2
3
4
5
6
7
8
9
10
11
12
RN monthly 7Be normalized average over 120 days

RN72

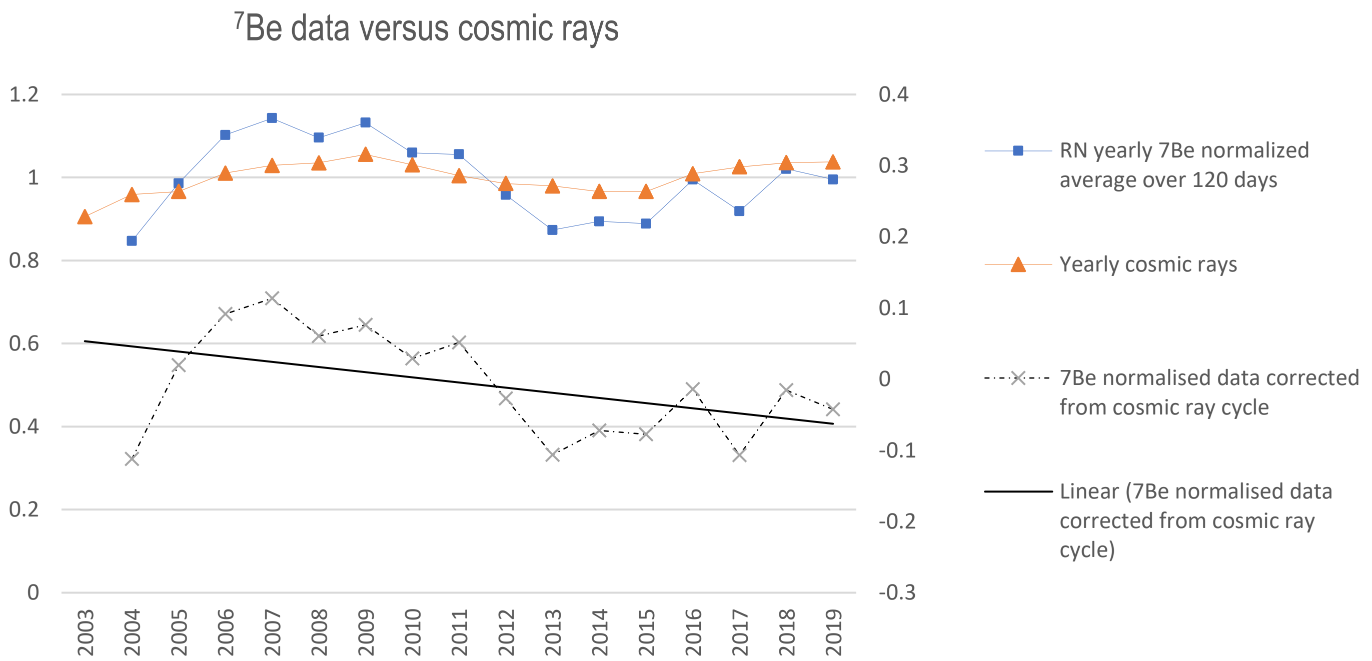

^{7}Be data versus cosmic rays
1.2
1
0.8
0.6
0.4
0.2
0
0.4
0.3
0.2
0.1
0
-0.1
-0.2
-0.3
2003
2004
2005
2006
2007
2008
2009
2010
2011
2012
2013
2014
2015
2016
2017
2018
2019
RN yearly 7Be normalized average over 120 days
Yearly cosmic rays
7Be normalised data corrected from cosmic ray cycle
Linear (7Be normalised data corrected from cosmic ray cycle)

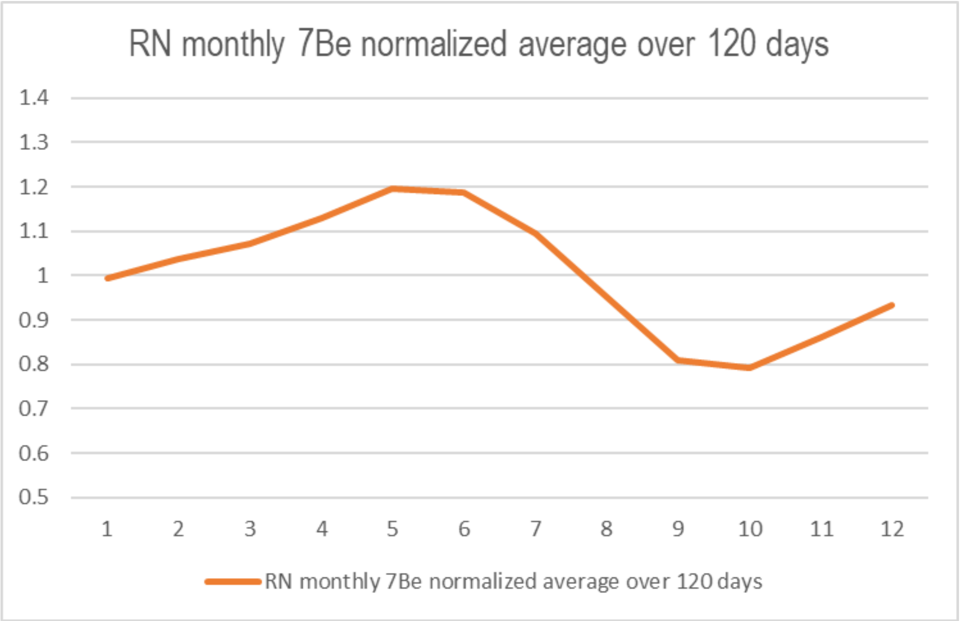

RN monthly 7Be normalized average over 120 days
1.4
1.3
1.2
1.1
1
0.9
0.8
0.7
0.6
0.5
1
2
3
4
5
6
7
8
9
10
11
12
RN monthly 7Be normalized average over 120 days

RN73

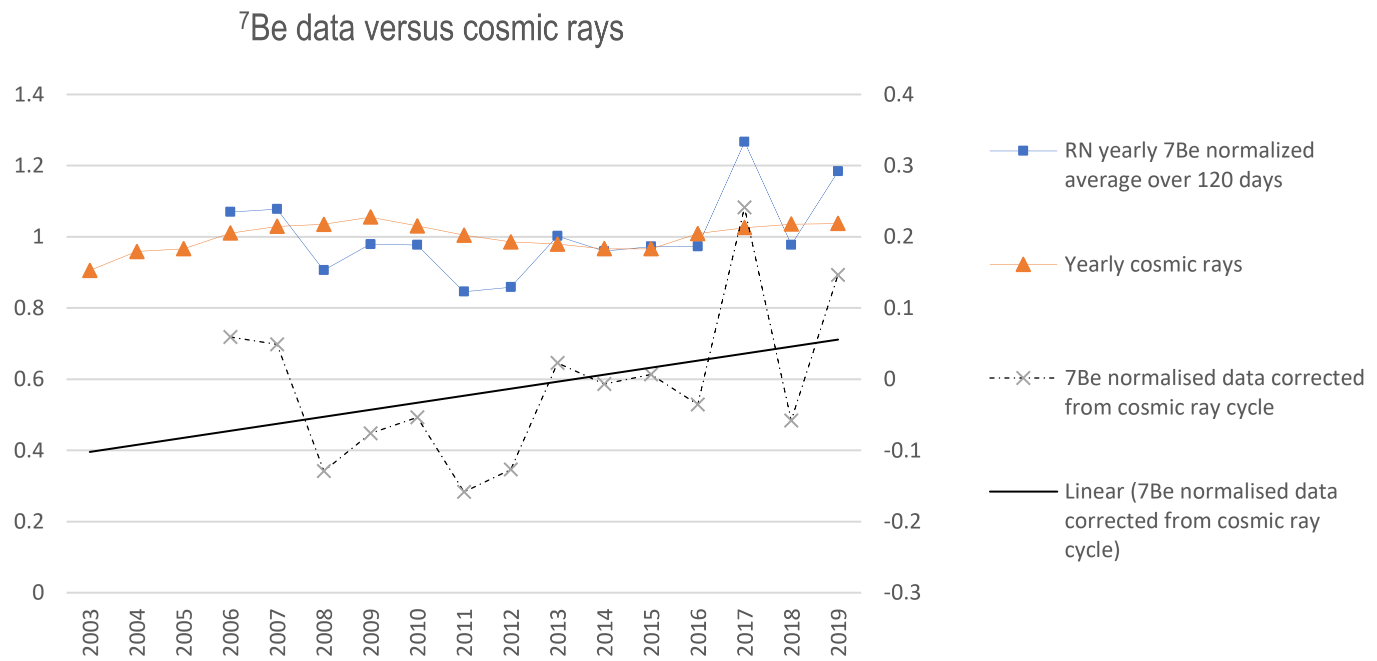

^{7}Be data versus cosmic rays
1.4
1.2
1
0.8
0.6
0.4
0.2
0
0.4
0.3
0.2
0.1
0
-0.1
-0.2
-0.3
2003
2004
2005
2006
2007
2008
2009
2010
2011
2012
2013
2014
2015
2016
2017
2018
2019
RN yearly 7Be normalized average over 120 days
Yearly cosmic rays
7Be normalised data corrected from cosmic ray cycle
Linear (7Be normalised data corrected from cosmic ray cycle)

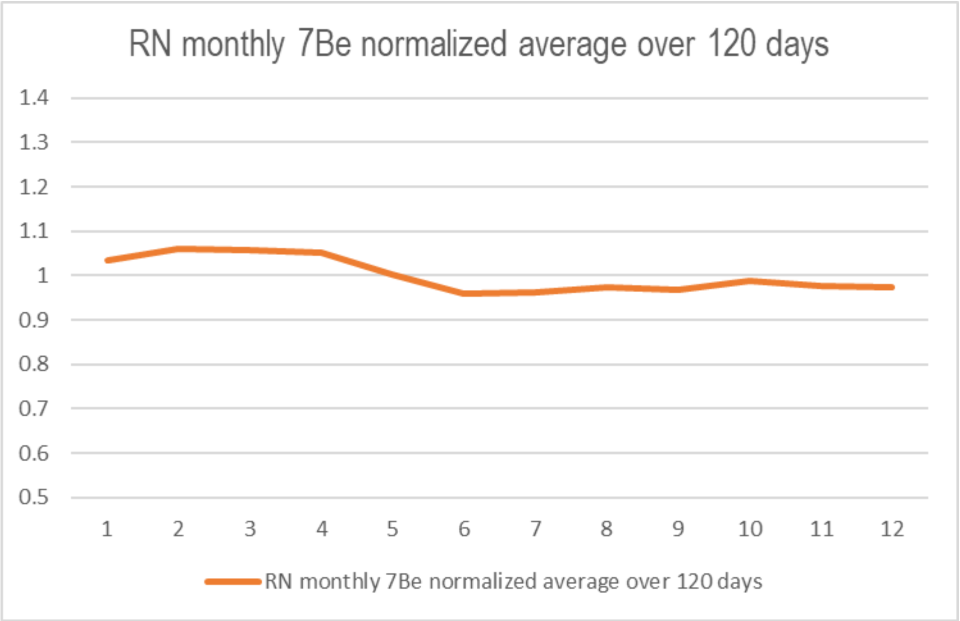

RN monthly 7Be normalized average over 120 days
1.4
1.3
1.2
1.1
1
0.9
0.8
0.7
0.6
0.5
1
2
3
4
5
6
7
8
9
10
11
12
RN monthly 7Be normalized average over 120 days

RN74

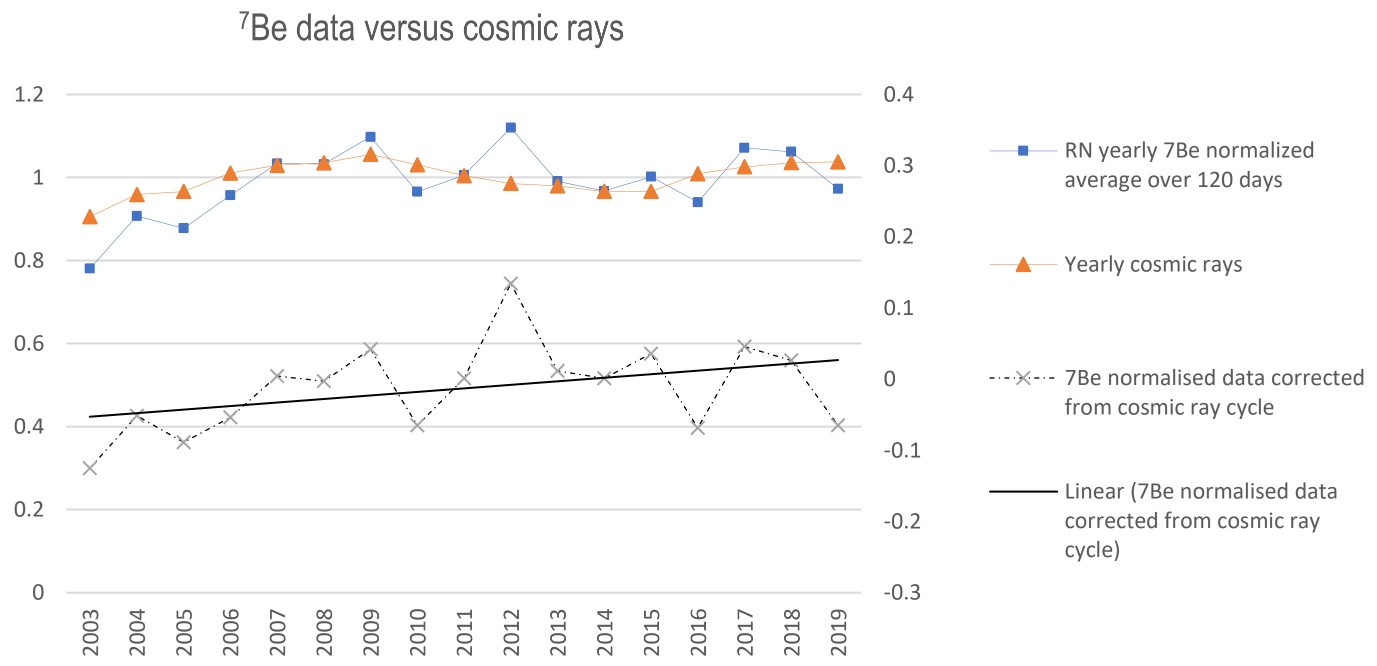

^{7}Be data versus cosmic rays
1.2
1
0.8
0.6
0.4
0.2
0
0.4
0.3
0.2
0.1
0
-0.1
-0.2
-0.3
2003
2004
2005
2006
2007
2008
2009
2010
2011
2012
2013
2014
2015
2016
2017
2018
2019
RN yearly 7Be normalized average over 120 days
Yearly cosmic rays
7Be normalised data corrected from cosmic ray cycle
Linear (7Be normalised data corrected from cosmic ray cycle)

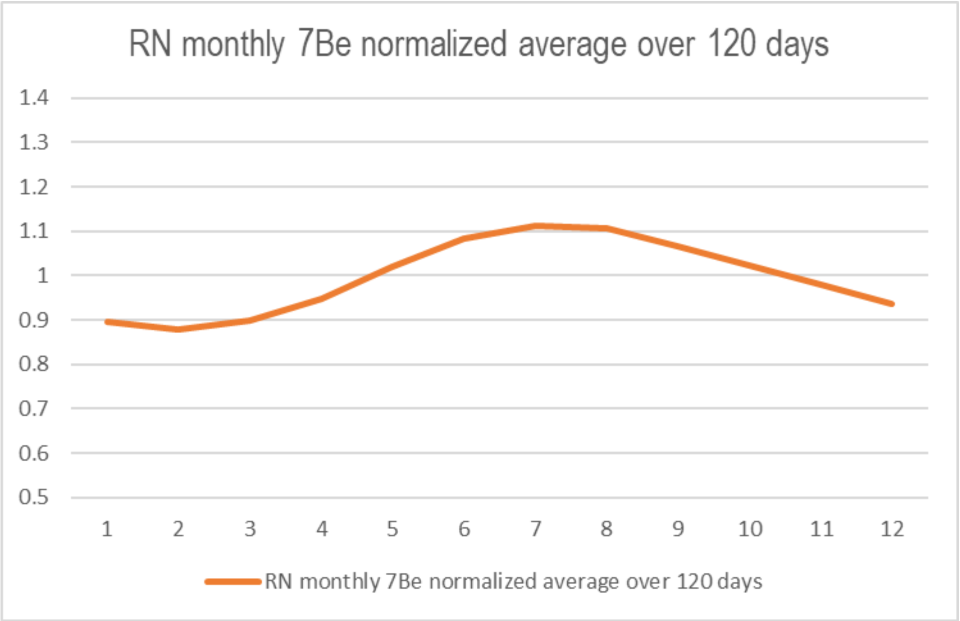

RN monthly 7Be normalized average over 120 days
1.4
1.3
1.2
1.1
1
0.9
0.8
0.7
0.6
0.5
1
2
3
4
5
6
7
8
9
10
11
12
RN monthly 7Be normalized average over 120 days

RN75

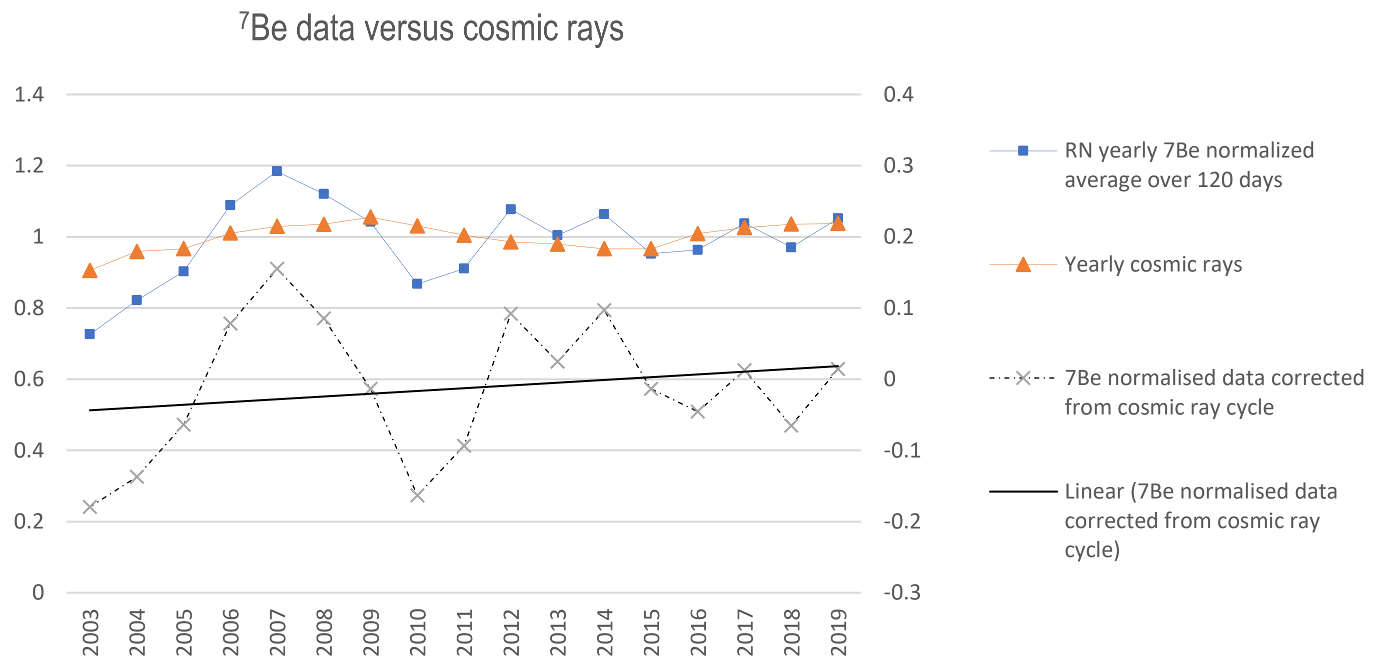

⁷Be data versus cosmic rays
1.4
1.2
1
0.8
0.6
0.4
0.2
0
0.4
0.3
0.2
0.1
0
-0.1
-0.2
-0.3
2003
2004
2005
2006
2007
2008
2009
2010
2011
2012
2013
2014
2015
2016
2017
2018
2019
RN yearly 7Be normalized average over 120 days
Yearly cosmic rays
7Be normalised data corrected from cosmic ray cycle
Linear (7Be normalised data corrected from cosmic ray cycle)

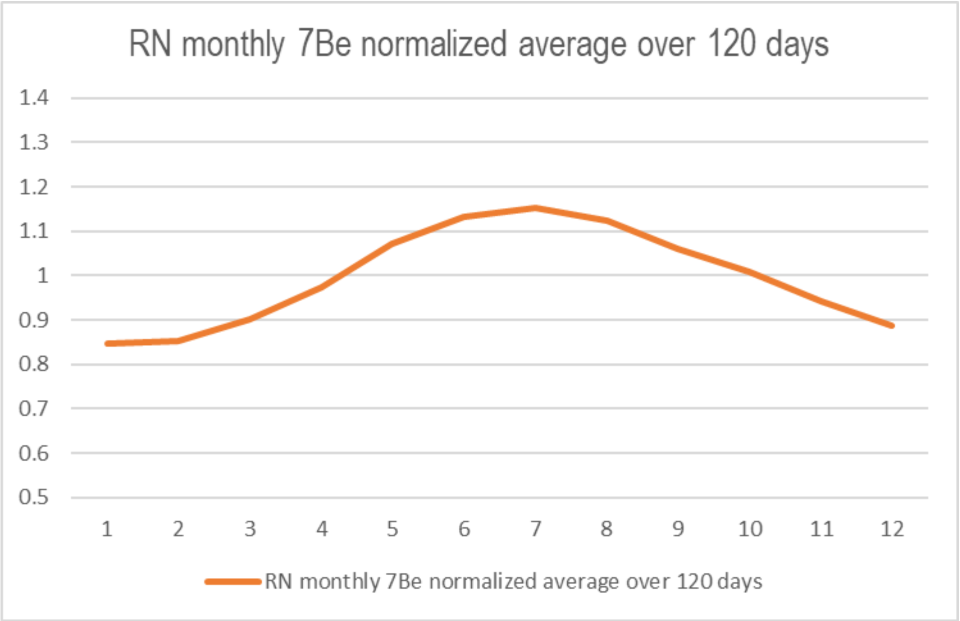

RN monthly 7Be normalized average over 120 days
1.4
1.3
1.2
1.1
1
0.9
0.8
0.7
0.6
0.5
1
2
3
4
5
6
7
8
9
10
11
12
RN monthly 7Be normalized average over 120 days

RN76

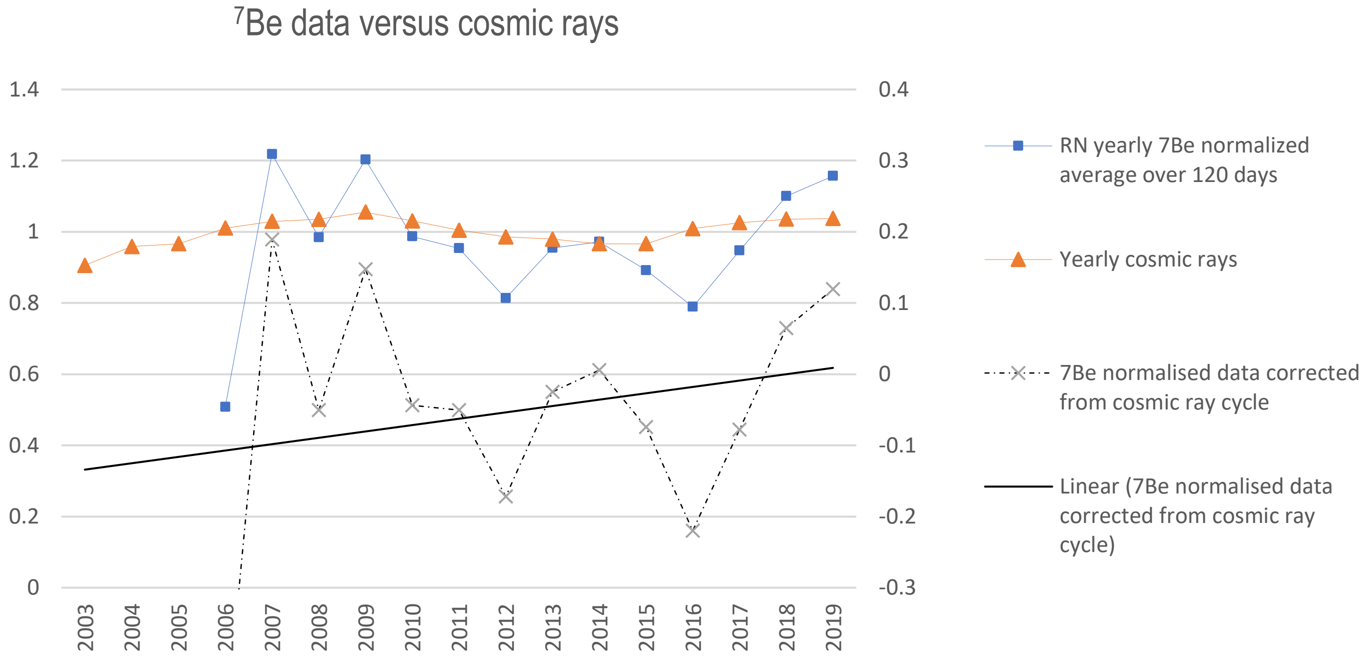

^{7}Be data versus cosmic rays
1.4
1.2
1
0.8
0.6
0.4
0.2
0
0.4
0.3
0.2
0.1
0
-0.1
-0.2
-0.3
2003
2004
2005
2006
2007
2008
2009
2010
2011
2012
2013
2014
2015
2016
2017
2018
2019
RN yearly 7Be normalized average over 120 days
Yearly cosmic rays
7Be normalised data corrected from cosmic ray cycle
Linear (7Be normalised data corrected from cosmic ray cycle)

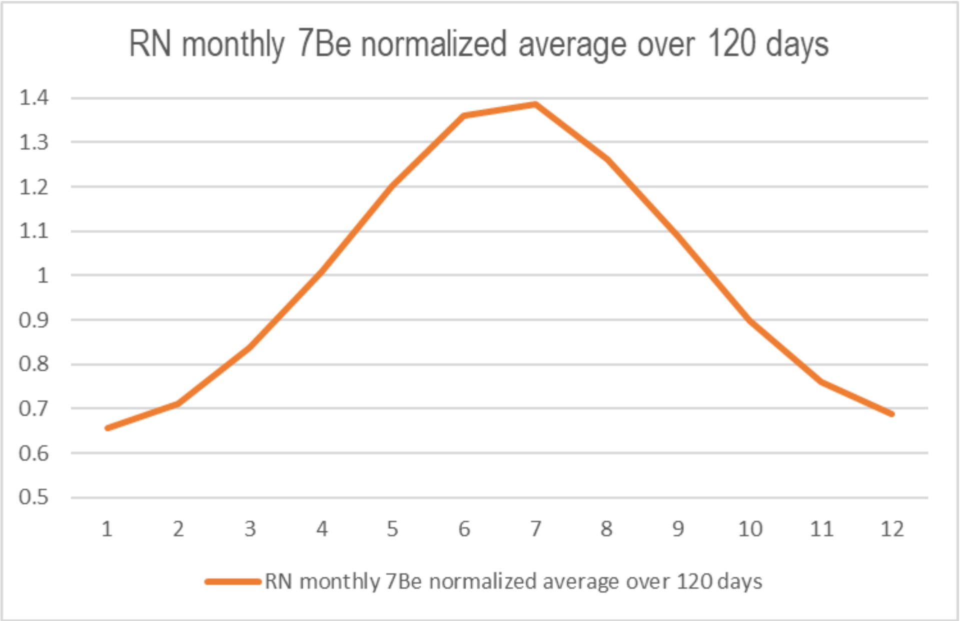

RN monthly 7Be normalized average over 120 days
1.4
1.3
1.2
1.1
1
0.9
0.8
0.7
0.6
0.5
1
2
3
4
5
6
7
8
9
10
11
12
RN monthly 7Be normalized average over 120 days

RN77

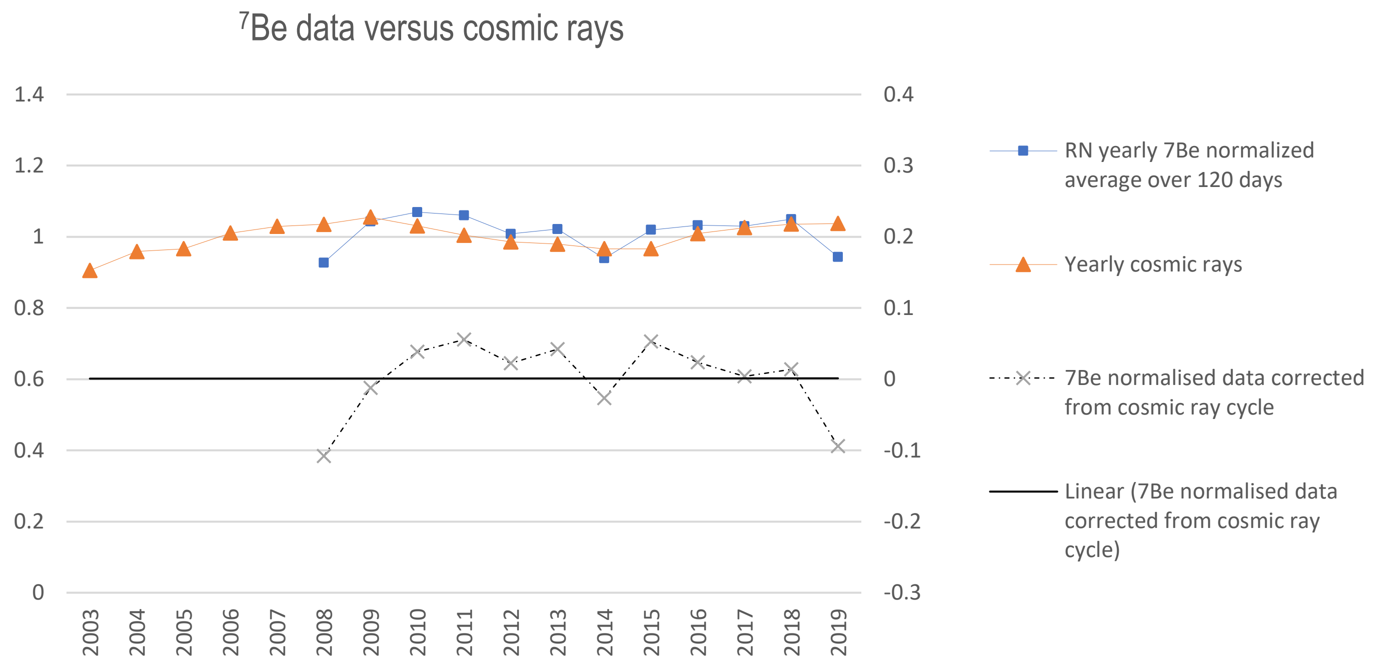

^{7}Be data versus cosmic rays
1.4
1.2
1
0.8
0.6
0.4
0.2
0
0.4
0.3
0.2
0.1
0
-0.1
-0.2
-0.3
2003
2004
2005
2006
2007
2008
2009
2010
2011
2012
2013
2014
2015
2016
2017
2018
2019
RN yearly 7Be normalized average over 120 days
Yearly cosmic rays
7Be normalised data corrected from cosmic ray cycle
Linear (7Be normalised data corrected from cosmic ray cycle)

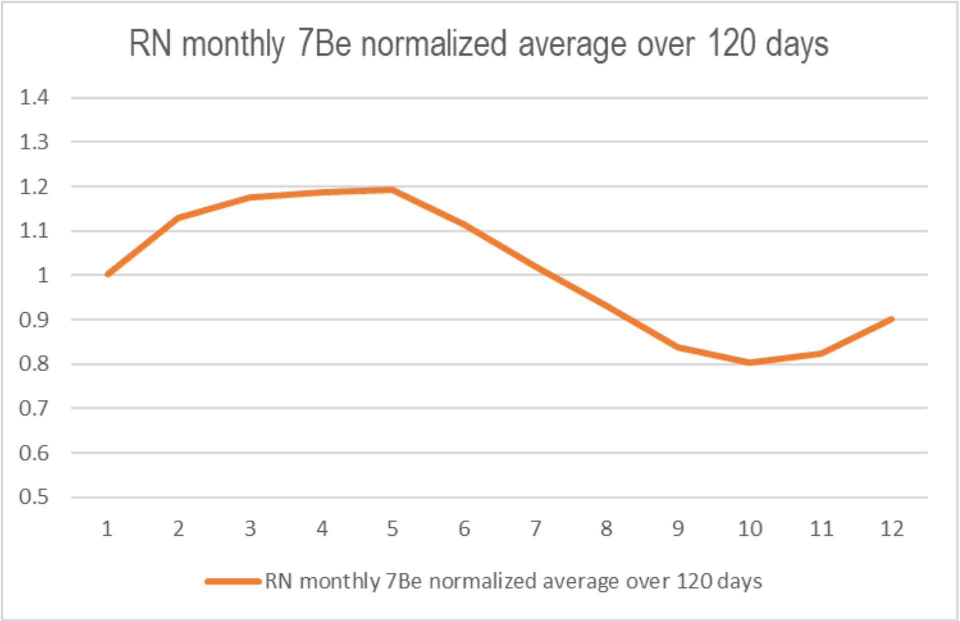

RN monthly 7Be normalized average over 120 days
1.4
1.3
1.2
1.1
1
0.9
0.8
0.7
0.6
0.5
1
2
3
4
5
6
7
8
9
10
11
12
RN monthly 7Be normalized average over 120 days

RN78

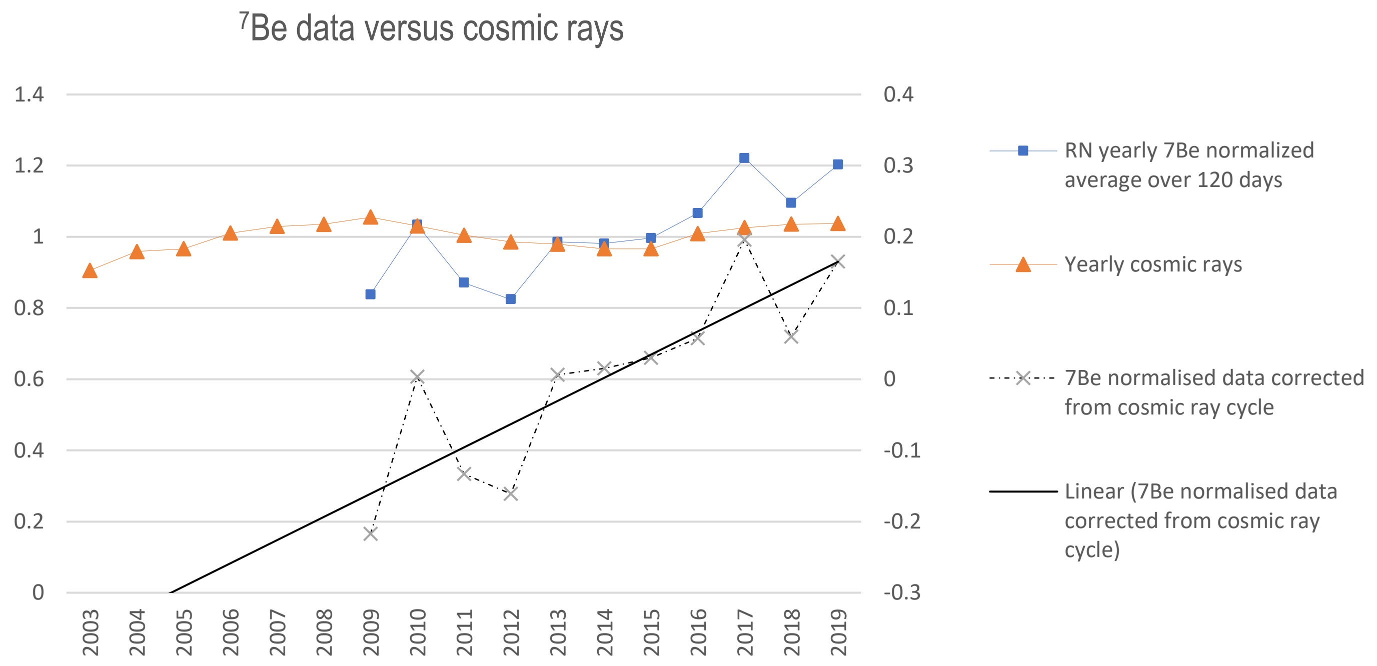

^{7}Be data versus cosmic rays
1.4
1.2
1
0.8
0.6
0.4
0.2
0
0.4
0.3
0.2
0.1
0
-0.1
-0.2
-0.3
2003
2004
2005
2006
2007
2008
2009
2010
2011
2012
2013
2014
2015
2016
2017
2018
2019
RN yearly 7Be normalized average over 120 days
Yearly cosmic rays
7Be normalised data corrected from cosmic ray cycle
Linear (7Be normalised data corrected from cosmic ray cycle)

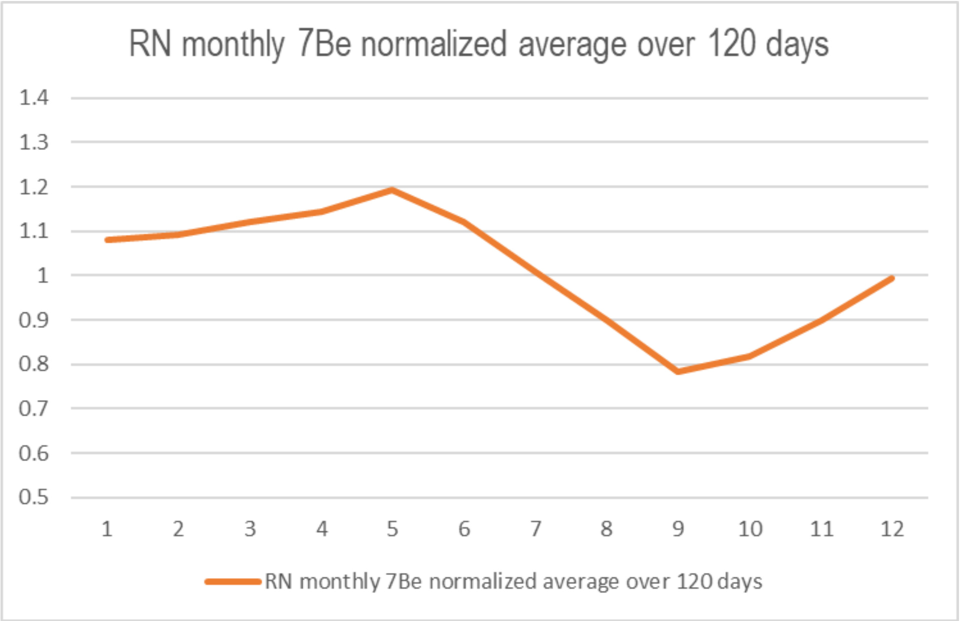

RN monthly 7Be normalized average over 120 days
1.4
1.3
1.2
1.1
1
0.9
0.8
0.7
0.6
0.5
1
2
3
4
5
6
7
8
9
10
11
12
RN monthly 7Be normalized average over 120 days

RN79

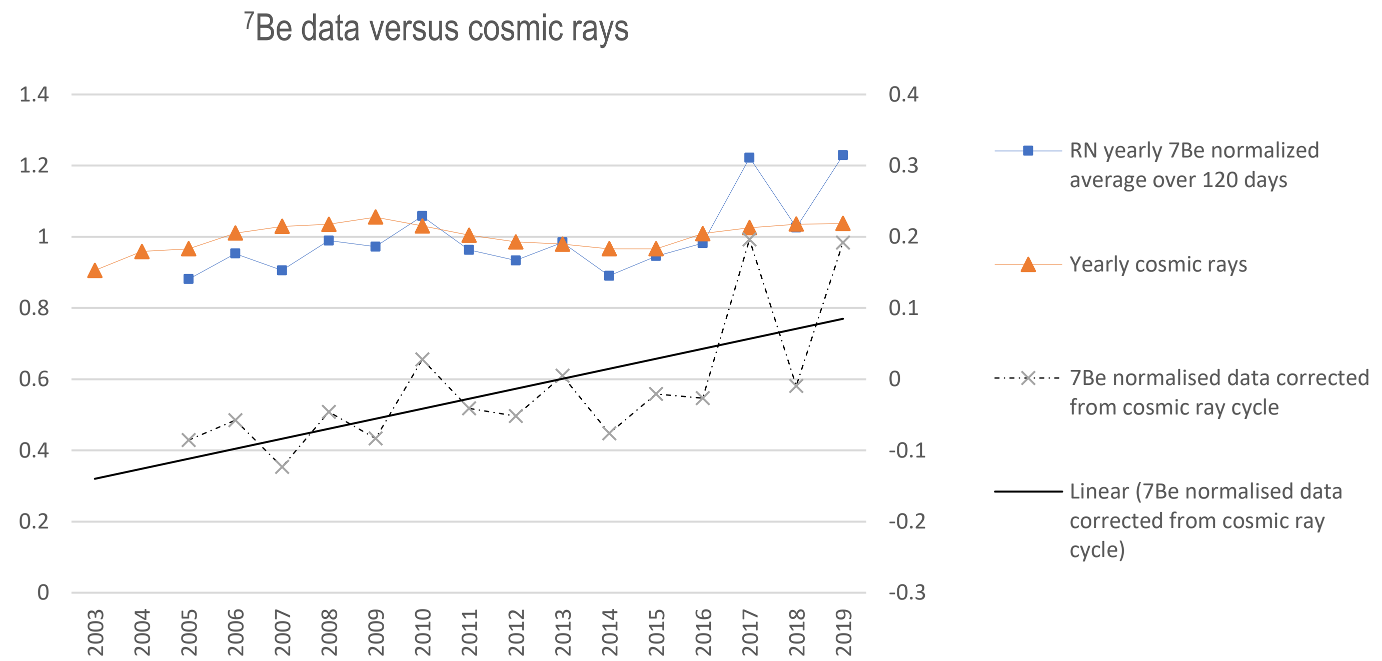

^{7}Be data versus cosmic rays
1.4
1.2
1
0.8
0.6
0.4
0.2
0
0.4
0.3
0.2
0.1
0
-0.1
-0.2
-0.3
2003
2004
2005
2006
2007
2008
2009
2010
2011
2012
2013
2014
2015
2016
2017
2018
2019
RN yearly 7Be normalized average over 120 days
Yearly cosmic rays
7Be normalised data corrected from cosmic ray cycle
Linear (7Be normalised data corrected from cosmic ray cycle)

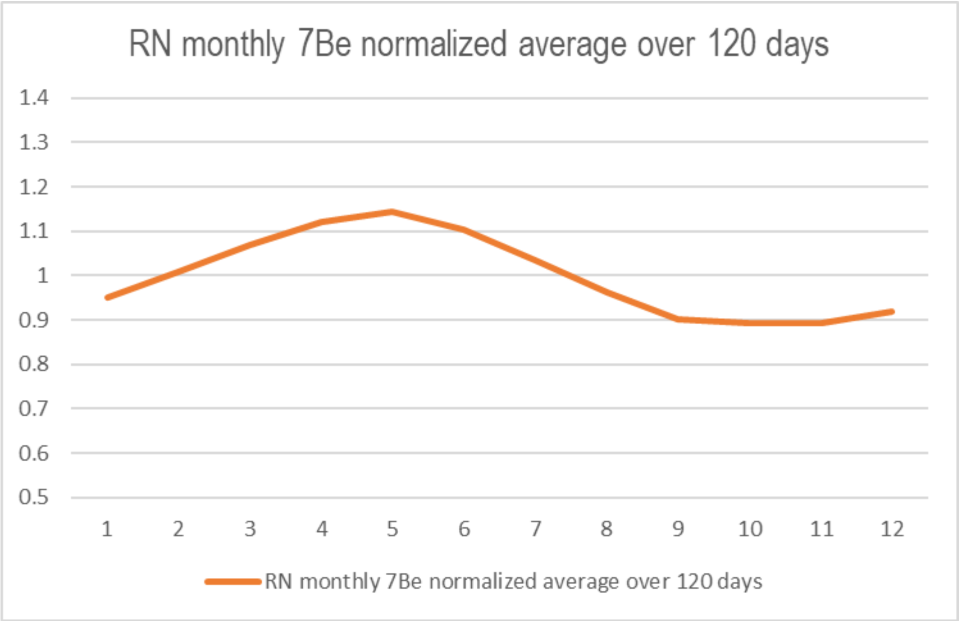

RN monthly 7Be normalized average over 120 days
1.4
1.3
1.2
1.1
1
0.9
0.8
0.7
0.6
0.5
1
2
3
4
5
6
7
8
9
10
11
12
RN monthly 7Be normalized average over 120 days

RN80

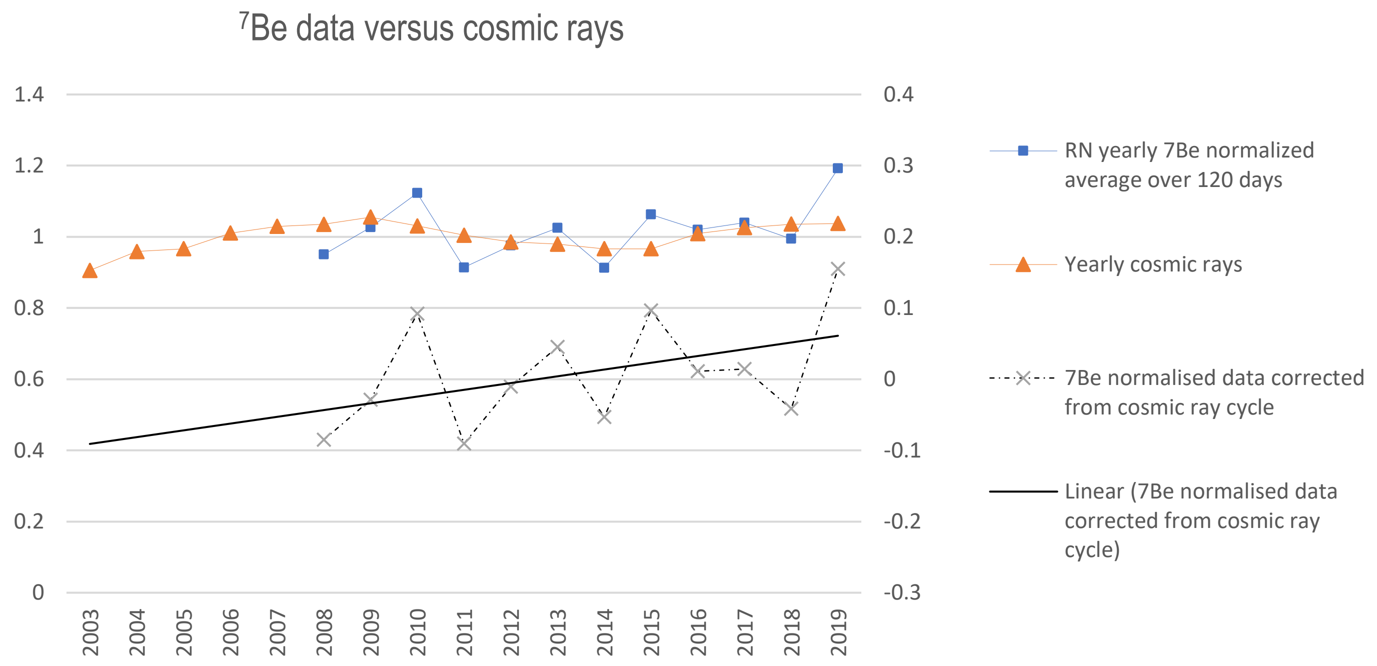
^{7}Be data versus cosmic rays
1.4
1.2
1
0.8
0.6
0.4
0.2
0
0.4
0.3
0.2
0.1
0
-0.1
-0.2
-0.3
2003
2004
2005
2006
2007
2008
2009
2010
2011
2012
2013
2014
2015
2016
2017
2018
2019
RN yearly 7Be normalized average over 120 days
Yearly cosmic rays
7Be normalised data corrected from cosmic ray cycle
Linear (7Be normalised data corrected from cosmic ray cycle)

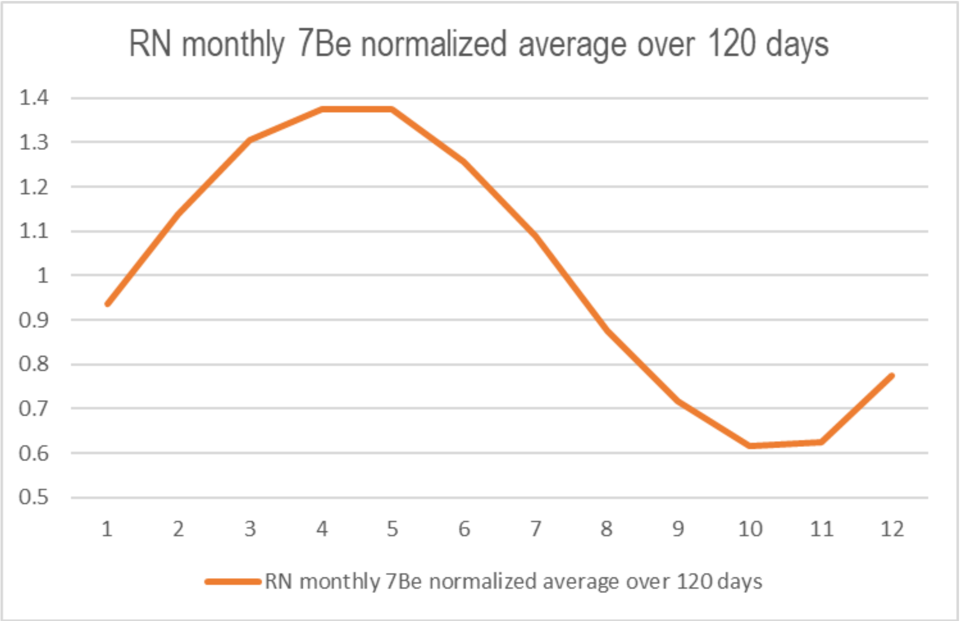
RN monthly 7Be normalized average over 120 days
1.4
1.3
1.2
1.1
1
0.9
0.8
0.7
0.6
0.5
1
2
3
4
5
6
7
8
9
10
11
12
RN monthly 7Be normalized average over 120 days
