# Supplementary material for: Radioisotopes demonstrate changes in global atmospheric circulation possibly caused by global warming
Source: Sci Rep. 2020 Jul 1;10:10695. doi: 10.1038/s41598-020-66541-5 (PMC7329870; doi:10.1038/s41598-020-66541-5)
Supplement: Supplementary file 7 — Supplementary Information Guide. [file 41598_2020_66541_MOESM7_ESM.docx]

SI Guide

**Radioisotopes demonstrate changes in global atmospheric circulation possibly caused by global warming**

**Authors:** Lucrezia Terzi*, Gerhard Wotawa, Michael Schoeppner, Martin Kalinowski, Paul R.J. Saey, Philipp Steinmann, Lan Luan and Paul W. Staten.

1. Extended data file

1_Extended_data_file_.pdf (1.9 MB)

Extended data file contains detailed description of IMS stations and post processing values of beryllium-7 data. Percentage values of beryllium-7 yearly growth versus the overall average; map of the International Monitoring System; reconstruction of downward branch of the Hadley circulation through beryllium-7 concentrations; surface chart of sodium-22/beryllium-7 ratio from Swiss stations; beryllium-7 growth versus overall average and the increase in the n. of days required per year to reach the maximum threshold; global surface temperature changes from 1880 to 2019; beryllium-7 growth trend at all 62 stations in 2003, 2011 and 2019; connection between CO_2_ emissions to climate change patterns and the use of beryllium-7 as a proxy; examples of different IMS station locations where tropopause profile based on GPS-RO data are compared to beryllium-7 activity concentrations.

1. Tables

Supplementary dataset

2_supplementary_dataset.xlsx (4.7 MB)

Normalised averaged of beryllium-7 concentrations including cosmic ray correction from 2003 to 2019. Daily and Yearly beryllium-7 values in worksheet 1 and 2 and Cosmic rays values listed in worksheet 3.

1. Figures

Beryllium-7 maps

3_Beryllium-7_map_slides.pdf (5.8 MB)

Global maps displaying the interpolation of beryllium-7 activity concentrations from 2003 to 2019.

Beryllium-7 trends

4_Beryllium-7_trend_charts.pdf (1.4 MB)

For each IMS station the seasonal and interannual trend of beryllium-7 is displayed.

1. Movies

Supplementary Movie 1

How nuclear monitoring links global warming to extreme weather.mp4 (23.6 MB)

Abstract video describes the correlation between beryllium-7 activity concentrations with extreme weather events.

Supplementary Movie 2

3_Berylium-7_map_slides.mp4 (7.7 MB)

Slide animation of beryllium-7 maps from 2003 to 2019. The sequence highlights the interannual variability of beryllium-7 records interpolated into a global map as an expression of tropopause height change.
